# Trichinella spiralis-induced mastocytosis and erythropoiesis are simultaneously supported by a bipotent mast cell/erythrocyte precursor cell

**DOI:** 10.1371/journal.ppat.1008579

**Published:** 2020-05-18

**Authors:** Juan M. Inclan-Rico, Christina M. Hernandez, Everett K. Henry, Hannah G. Federman, Chandler B. Sy, John J. Ponessa, Alexander D. Lemenze, Nathanael Joseph, Patricia Soteropoulos, Aimee M. Beaulieu, George S. Yap, Mark C. Siracusa

**Affiliations:** 1 Center for Immunity and Inflammation, New Jersey Medical School, Rutgers-The State University of New Jersey, Newark, New Jersey, United States of America; 2 Department of Medicine, New Jersey Medical School, Rutgers-The State University of New Jersey, Newark, New Jersey, United States of America; 3 The Department of Pathology, Immunology and Laboratory Medicine, New Jersey Medical School, Rutgers-The State University of New Jersey, Newark, New Jersey, United States of America; 4 The Genomics Center, New Jersey Medical School, Rutgers-The State University of New Jersey, Newark, New Jersey, United States of America; 5 Department of Microbiology, Biochemistry and Molecular Genetics, New Jersey Medical School, Rutgers-The State University of New Jersey, Newark, New Jersey, United States of America; University of Manchester, UNITED KINGDOM

## Abstract

Anti-helminth responses require robust type 2 cytokine production that simultaneously promotes worm expulsion and initiates the resolution of helminth-induced wounds and hemorrhaging. However, how infection-induced changes in hematopoiesis contribute to these seemingly distinct processes remains unknown. Recent studies have suggested the existence of a hematopoietic progenitor with dual mast cell-erythrocyte potential. Nonetheless, whether and how these progenitors contribute to host protection during an active infection remains to be defined. Here, we employed single cell RNA-sequencing and identified that the metabolic enzyme, carbonic anhydrase (Car) 1 marks a predefined bone marrow-resident hematopoietic progenitor cell (HPC) population. Next, we generated a Car1-reporter mouse model and found that Car1-GFP positive progenitors represent bipotent mast cell/erythrocyte precursors. Finally, we show that Car1-expressing HPCs simultaneously support mast cell and erythrocyte responses during *Trichinella spiralis* infection. Collectively, these data suggest that mast cell/erythrocyte precursors are mobilized to promote type 2 cytokine responses and alleviate helminth-induced blood loss, developmentally linking these processes. Collectively, these studies reveal unappreciated hematopoietic events initiated by the host to combat helminth parasites and provide insight into the evolutionary pressure that may have shaped the developmental relationship between mast cells and erythrocytes.

## Author summary

Helminth parasites infect approximately 2 billion people and represent a significant public health concern. Helminths undertake complex developmental life cycles through multiple organs and as a result cause substantial tissue damage. To combat this, mammals have evolved mechanisms to initiate balanced immune responses that promote inflammation needed to seclude parasites in granulomas, reduce parasitic burdens and mitigate the consequences of helminth-induced wounds. Despite their clinical importance, the mechanisms that regulate these events remain poorly defined. Here we have uncovered a unique progenitor cell that supports both proinflammatory mast cell responses and red blood cell development, thereby simultaneously initiating both of these host-protective responses. Collectively, these studies reveal unappreciated events initiated by the host to combat pathogens that infect billions of individuals worldwide.

## Introduction

It is estimated that close to one third of the world’s population is infected with one or more parasitic helminths, making them among the most prevalent pathogens worldwide[1, 2]. Although helminth infections rarely result in mortality, they represent a substantial cause of debilitating morbidities. For example, children infected with helminths often suffer from developmental and cognitive issues thought to be caused by infection-induced malnutrition and anemia[2]. Helminths have infected humans for millennia and as a result have coevolved and developed sophisticated relationships with their mammalian hosts. These relationships are reflected by the complex life cycles of helminths that require their passage through several host tissues. While the completion of these life cycles allows the parasites to reach their reproductive stages, they are detrimental to the host and result in the substantial wounding of affected organs. Therefore, to promote protection the host must initiate highly regulated forms of inflammation that are strong enough to expel the worms but measured in scope to allow for the healing of helminth-affected tissues in order to prevent additional hemorrhaging and blood loss.

Host-protective responses to helminths are highly dependent on the initiation of type 2 cytokine-mediated inflammation. While type 2 cytokine production is necessary to seclude the parasites in granulomas and to expel the worms, type 2 inflammation also initiates critical healing and tissue remodeling events required to mitigate infection-induced wounding[3–5]. It is well established that many terminally differentiated immune cells participate in these processes including T helper type 2 cells (T_H_2), group 2 innate lymphocytes (ILC2s), eosinophils, basophils, mast cells and M2 macrophages[6]. In addition, emerging studies have also demonstrated that specialized hematopoietic progenitor cell (HPC) populations are rapidly mobilized from the bone marrow, enter the periphery and also support protective immunity to helminths[7–10]. Despite these advances, the cellular and molecular events that regulate helminth-mobilized HPCs and their exact contributions to host-protective responses remain unknown.

*Trichinella spiralis* is one of several species of *Trichinella* that are reported to infect a broad range of mammals and other hosts, including birds and reptiles[11]. *T*. *spiralis* is contracted by the consumption of infected tissue and undergoes a life cycle that includes an acute intestinal phase where infectious larvae mature into adults, and a long-lived muscle phase where newborn larvae encyst in skeletal muscle[11]. Our previous work recently identified a unique HPC population that enters the periphery following a *T*. *spiralis* challenge and promotes mast cell responses that contribute to worm clearance[12]. Functional and transcriptional profiling of *T*. *spiralis*-mobilized HPCs revealed that their enhanced mast cell potential is associated with their elevated expression of the metabolic enzymes carbonic anhydrase (Car)1 and 2. Critically, our previous studies also demonstrated that Car enzyme inhibition was sufficient to prevent *Trichinella*-induced mast cell responses, type 2 cytokine-mediated inflammation and worm clearance[12]. Despite these advances, whether these HPCs acquire Car enzyme expression after entering the periphery, or Car-enzyme expressing cells are bone marrow-resident at steady-state remains unknown. Moreover, the full developmental capacity and contributions of these specialized HPCs to both worm clearance and the mitigation of helminth-induced wounding remains to be defined.

Here we performed unbiased single cell RNA-sequencing (RNA-seq) analysis of bone marrow-resident progenitor cells from naïve mice and determined that specific cell clusters were defined by their high expression of carbonic anhydrase 1 and 2. To further evaluate Car enzyme-expressing HPCs, we generated a Car1-green fluorescent protein (GFP) reporter mouse model that allowed us to evaluate this unique cell population at both steady-state and in the context of a *Trichinella spiralis* infection. Using a combination of discovery-based and classical reductionist approaches we were able to determine that Car enzyme-expressing progenitors are mobilized following infection and possess dual mast cell and erythrocyte potential. Further, adoptive transfer studies demonstrate that the dual potential of Car-expressing cells allows them to support mast cell and erythrocyte development following a *T*. *spiralis* infection.

Emerging studies investigating the relationships of distinct cell lineages indicate that mast cell and erythrocyte development are highly related[13–15]. Despite these advances, the surface markers and molecular signatures of mast cell/erythrocyte progenitors remain to be fully defined. Additionally, the benefits provided by having these distinct cell types developmentally linked remains unknown. Importantly, Car1 enzyme expression is a shared feature of both mast cell and erythrocyte lineage commitment[12, 16], and the studies presented here identify Car1 as a defining feature of a mast cell/erythrocyte progenitor population. Although additional studies are needed to determine how these mast cell/erythrocyte progenitors act in the context of other parasitic helminth infections, it is possible that these bipotent cells might be capable of promoting host-protective responses needed to expel parasites while simultaneously supporting recovery from infection-induced anemia. Collectively, these data provide insight into the role that mast cell/erythrocyte progenitors play in the context of infection and highlight some of the possible benefits that may result from having these distinct lineages developmentally linked. Finally, these studies identify Car1 as a potential therapeutic target for the treatment of mast cell- and erythrocyte-associated disorders.

## Results

### Car1 expression is a defining feature of specific progenitor cell populations

**Fig 1:**
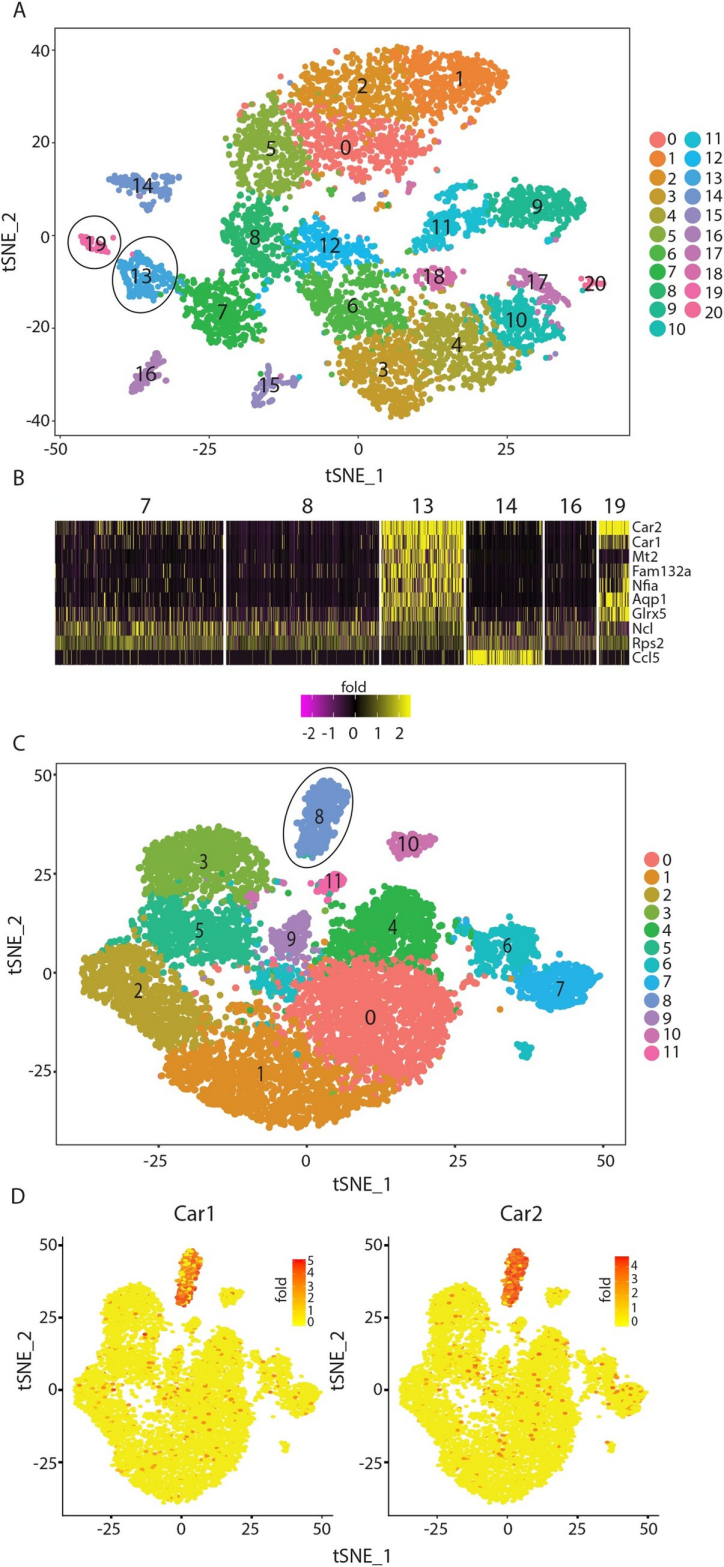
Carbonic anhydrase 1 and 2 are highly expressed by specific subsets of progenitor cells. (**A**), t-distributed stochastic neighbor embedding (TSNE) plot illustrating defined clusters of cells generated by single cell RNA-seq of live, CD45+ CD19- CD3- Ly6G- bone marrow-resident cells. (**B**), Heat map illustrating the top 10 marker genes for cluster 13 and their expression levels in other relevant clusters. (**C**), TSNE plots from bone marrow samples downloaded from NCBI (GSE108097). (**D**), TSNE plots illustrating expression of Car1 and Car2 across clusters. (A), Generated by pooling bone-marrow cells from 3 individual mice.

Our previous studies revealed that Car1 and Car2 are highly expressed by mast cell progenitors that enter the periphery, expand in number and promote immunity to *Trichinella spiralis*[12]. Further, these studies demonstrated that pharmacologically or genetically interrupting Car1 activity is sufficient to prevent mast cell development. Collectively, these data suggest that Car1 operates as a positive regulator of mast cell development and mast cell-mediated immunity to *T*. *spiralis*[12]. Despite these advances, whether Car enzyme expression is acquired post-infection as the progenitor cells are mobilized or whether there are predefined subsets of Car1-expressing progenitors that exist in the bone marrow at steady-state remains unknown. To further address this question, we took an unbiased discovery-based approach and performed single cell RNA-sequencing on bone marrow-resident cells to determine if Car1 and/or Car2 are signature genes that define subsets of naïve progenitor populations. In order to further enrich for progenitor cells, we excluded mature lymphocytes and neutrophils from our single cell input population. Single cell RNA-sequencing analysis of bone marrow-resident cells revealed the presence of 20 distinct cell clusters using the Seurat workflow (**Fig 1A**). This approach entails cell-to-cell normalization, scaling, dimensional clustering and finally gene marker identification per cluster[17]. Importantly, the generated gene lists identify the defining genes for each cluster and illustrate the expression of genes associated with mature cell populations, such as basophils, that are identified by expression of the genes encoding the mast cell protease (Mcpt) 8 and the high affinity IgE receptor FcεRIα that were enriched in cluster 16[18] (**S1 Fig, S1 Table**). Critically, these data also illustrate that *Car1* and *Car2* were genes significantly associated with clusters 13 and 19, respectively (**Fig 1A and 1B), (S1 Fig**). Interestingly, cluster 19 was also defined by the erythrocyte-associated hemoglobulin chains (*Hbb-bt*, *Hba-a2*, *Hba-a1* and *Hbb-bs*) (**S1 Fig, S1 Table**)[19]. In contrast, cluster 13 expressed lower levels of erythrocyte-associated genes but expressed higher levels of metallothionein 2 (*Mt2*), which has previously been associated with activation of basophils and mast cells (**S1 Fig, S1 Table**) [20]. To further confirm our observation, we evaluated publicly available single cell RNA-seq data generated using an alternative Microwell-Seq platform[21]. Analysis of data available for bone marrow-resident cells revealed the presence of 11 distinct clusters (**Fig 1C**). Further, when the defining genes distinguishing those clusters were evaluated, *Car1* and *Car2* were among the top genes that defined cluster 8 (**Fig 1C and 1D**). Collectively, these unbiased discovery-based studies suggest that *Car1* and *Car2* are genes that define specific subsets of bone marrow-resident progenitor cells that may play important host-protective roles following a helminth challenge.

### Car1-expressing progenitor cells expand in the periphery post-*Trichinella* infection

**Fig 2:**
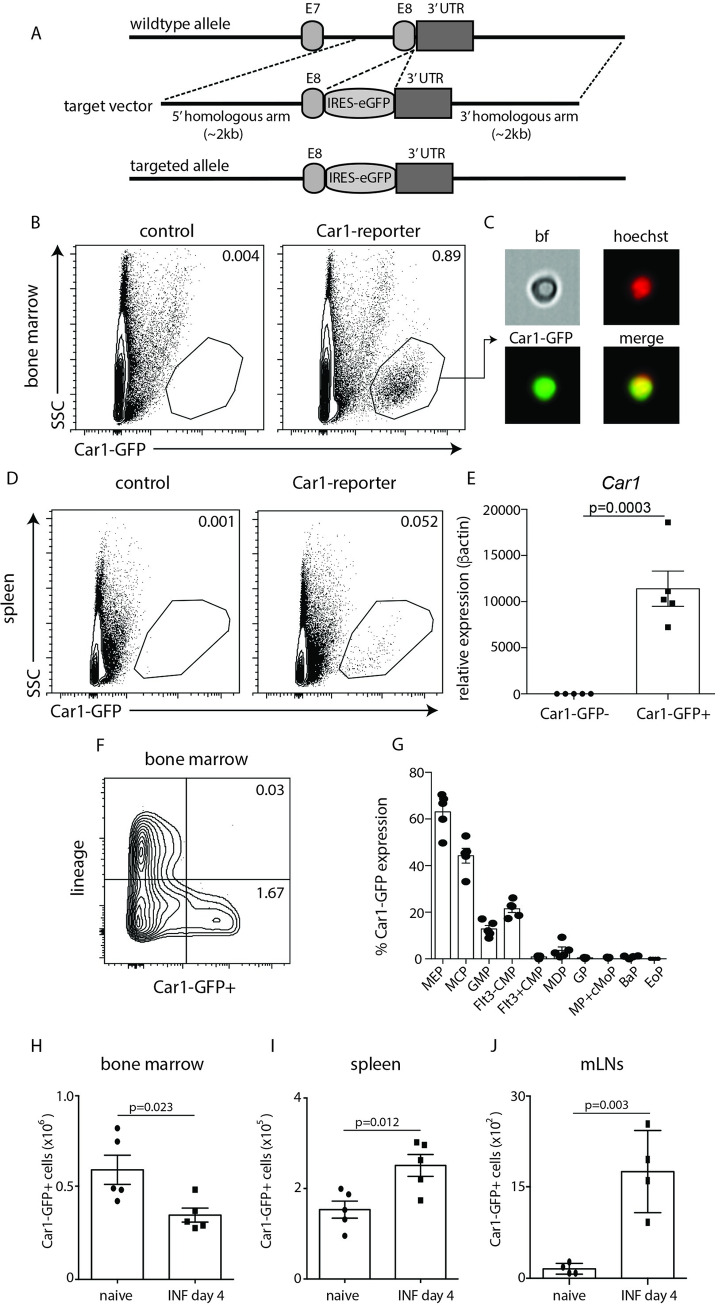
Car1-expressing HPCs are found in the gut-associated lymphoid tissue post-*Trichinella* infection. (**A**), Schematic illustrating targeting strategy and placement of IRES following exon 8 of Car1. Flow cytometric analysis of (**B**) bone marrow-resident cells or (**D**) splenic cells from control or Car1-GFP^+/+^ mice illustrating Car1-GFP expression. (**C**), AMNIS imaging flow cytometry illustrating Car1-GFP expression from bone marrow-resident cells. (**E**), *Car1* expression levels within sort-purified lineage- GFP- and lineage- GFP+ cells from the bone marrow were evaluated by RT-PCR. (**F**), Car1-GFP expression within lineage- and lineage+ compartments in the bone marrow was determined by flow cytometric analysis. (**G**), Car1-GFP expression by bone marrow-resident MEPs, MCPs, Flt3-CMPs, Flt3+CMPs MDPs, GPs, MP+cMoPs, BaPs and EoPs was evaluted. Car1-GFP+ cells were quantified in the (**H**) bone marrow, (**I**) spleen and (**J**) mesenteric lymph nodes (MLNs) on day 4 post-Trichinella infection. (A, B, D-J), Representative of at least 3 separate experiments. (C), Representative picture taken from a single experiment analyzing thousands of cells bf = bright field.

Unlike some carbonic anhydrase family members that are expressed on the surface of cells, Car1 and Car2 reside in the cytoplasm, which precludes their use for sort-purification strategies[22]. To address this issue, we generated mice that contain an internal ribosome entry site (IRES) and green fluorescent protein (GFP) tag immediately following the eighth and final exon of *Car1* (**Fig 2A**) via CRISPR/Cas9 technology. Car1 was chosen based on our previous work demonstrating that it appears to play a more important role in mast cell development than Car2[12]. This mouse model allows for the expression of Car1 to be tracked by GFP expression without interrupting its enzymatic functions. Conventional and AMNIS-based image flow cytometric analysis demonstrated that Car1-expressing cells could be found in the bone marrow and spleen of Car1-GFP reporter mice when compared to littermate controls (**Fig 2B–2D**). First, we sort-purified Car1-GFP+ cells to confirm that *Car1* gene expression was enriched in the GFP+ compartment. The relative expression levels of *Car1* was found to average 2.9 in the Car1-GFP- compartment and 11,398 in the Car1-GFP+ compartment (**Fig 2E**). Next, we stained for surface markers associated with mature cell lineages and demonstrated that the vast majority of Car1-expressing cells were lineage negative in both the bone marrow and the spleen (**Fig 2F, S2A Fig**). Further, flow cytometric analysis revealed that Car1-GFP+ cells express several markers that have been previously associated with hematopoietic progenitors (**S2B Fig**), suggesting they represented hematopoietic stem/progenitor cells. To further determine the identity of Car1-GFP positive cells we gated on markers known to traditionally define various subsets of hematopoietic progenitors and evaluated Car1-GFP expression within those gated populations. The most apparent Car1-GFP expression was found in compartments traditionally described as megakaryocyte-erythroid progenitors (MEPs) and mast cell progenitors (MCPs) with lower expression levels present in granulocyte/macrophage progenitors (GMPs) and FMS-like tyrosine kinase 3 (FLT3) negative common myeloid progenitors (CMPs). Little to no expression was detected in other progenitor compartments including basophil progenitors (BaPs) and eosinophil progenitors (EoPs) (**Fig 2G**). Collectively, these data demonstrate that Car1 expression marks previously unappreciated heterogeneity within bone marrow-resident progenitor cell populations that is not detected by traditional surface staining. Next, we monitored the presence of Car1-GFP+ cell numbers following a *T*. *spiralis* challenge and found that they were significantly decreased in the bone marrow on day 4 post-infection (**Fig 2H**). Importantly, the decreased presence of Car1-expressing cells in the bone marrow was associated with an increased presence of Car1-GFP+ cells in both the spleen and gut-associated lymphoid tissue on day 4 post-*Trichinella* (**Fig 2I and 2J**). Consistent with a heightened proliferative state, significantly more Car1-GFP+ cells were also found to be BrdU positive following infection (**S3A–S3C Fig**). These findings are consistent with our previous studies showing that Car1-expressing progenitor populations expand in the periphery and promote protective immunity to *T*. *spiralis*[12].

### Car1-expressing progenitor cell responses are associated with *Trichinella*-induced anemia and mastocytosis

Analysis of Car1-expressing cells suggests that they represent progenitors that fall within compartments of various developmental potential, with their prevalence being most pronounced in the MCP and MEP compartments (**Fig 2G**). These data are consistent with previous reports illustrating that Car enzymes are highly expressed during erythrocyte development as well as our previous studies identifying Car1 as a critical regulator of mast cell lineage commitment and the development of mast cell-dependent immunity to *T*. *spiralis*. [12, 16]. Further, these findings provoke the hypothesis that Car1-expressing progenitor cells may possess the potential to support both mast cell and erythrocyte development following a helminth challenge.

**Fig 3:**
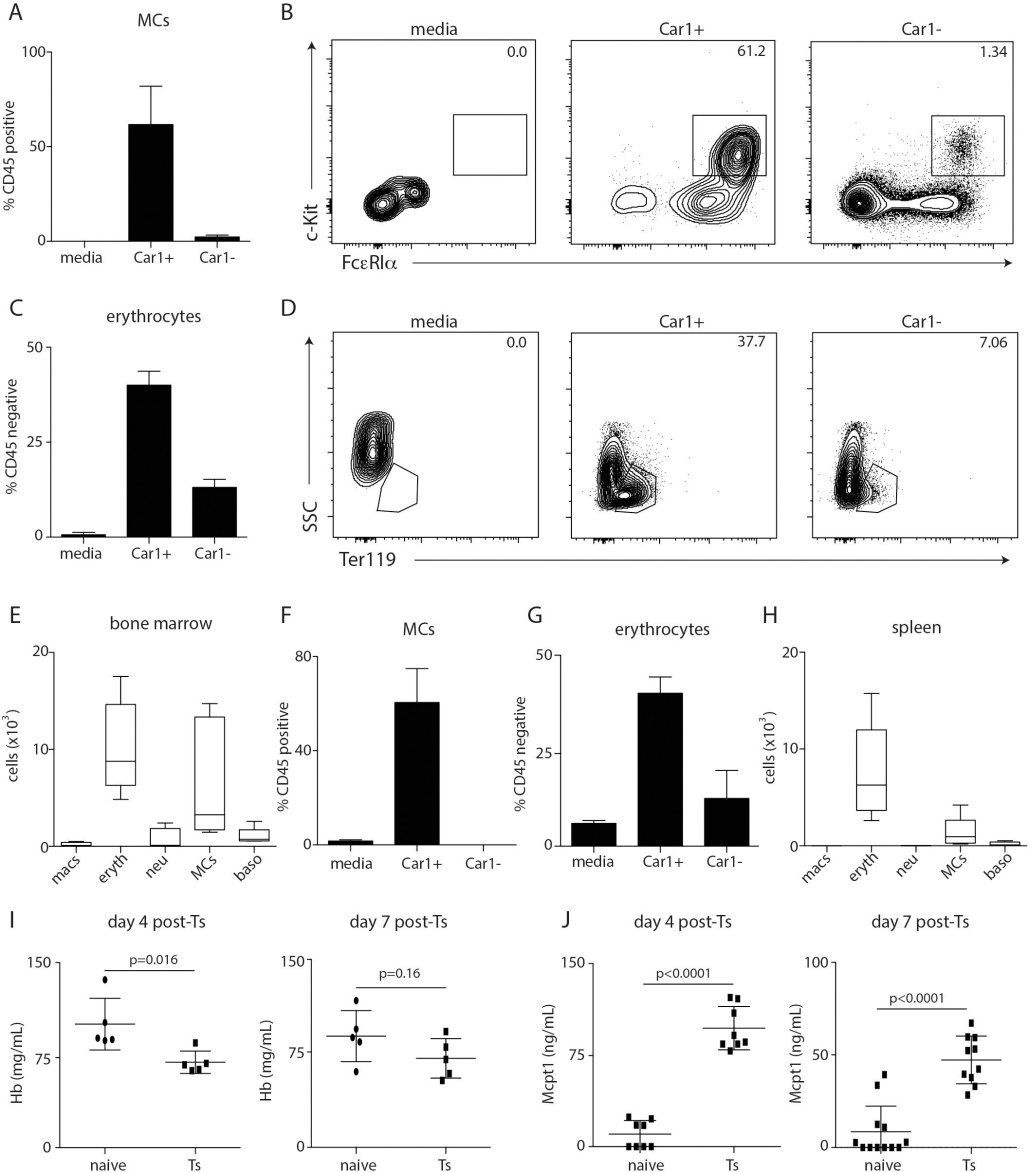
Car1-expressing HPC responses correlate with *Trichinella*-induced anemia and mast cell responses. Lineage negative Car1+ or lineage negative Car1- cells were sort-purified from the bone marrow of mice and seeded into MethoCult with hematopoietic cytokines. The percentages of (**A,B**) mast cells (MCs) and (**C,D**) erythrocytes were evaluated by flow cytometric analysis post-culture. (**E**), The number of macrophages (macs), erythrocytes (eryth), neutrophils (neu), MCs, and basophils (basos) were determined post-culture. Car1-GFP+ or Car1-GFP- cells were sort-purified from the spleens of mice and seeded into MethoCult. The percentage of (**F**) MCs and (**G**) erthrocytes were evaluated by flow cytometric analysis post-culture. (**H**), The number of macs, eryth, neu, MCs, and basos were determined post-culture. (**I**), Hemoglobin (Hb) levels were quantified on days 4 and 7 post-*Trichinella* infection. (**J**), Serum levels of mast cell protease 1 (Mcpt1) were quantified on days 4 and 7 post-*Trichinella spiralis* (Ts) infection. (A-J), Representative of at least 3 separate experiments. (E,H), Illustrate data pooled from 2 separate experiments.

To further evaluate their developmental capacity, we sort-purified Car1-expressing cells from the bone marrow of naïve mice and cultured them using methylcellulose conditions that are permissive for granulocyte, macrophage and erythrocyte lineages[23, 24]. Importantly, sort-purified lineage negative Car1-expressing cells were found to be highly enriched for mast cell (**Fig 3A and 3B**) and erythrocyte development (**Fig 3C and 3D**) compared to sort-purified lineage negative Car1 negative cells. Further, the majority of progeny from Car1-positive cells isolated from the bone marrow were found to be erythrocytes and mast cells with very few macrophages, neutrophils, or basophils detected post-culture (**Fig 3E**). This was in contrast to Car1-GFP negative cultures that exhibited robust macrophage and neutrophil development under the same conditions (**S4A and S4B Fig**). Similar results were also seen when Car1-expressing cells in the spleen were evaluated (**Fig 3F–3H, S4C and S4D Fig**). These data are consistent with Car1 expression being present in MCP and MEP compartments and further suggest that these cells might support mast cell and erythrocyte development post-*Trichinella* infection (**Fig 2G**). To further assess this possibility, we evaluated whether Car1-expressing progenitor cell responses post-*Trichinella* were associated with recovery from infection-induced anemia and mast cell activation. While no signs of anemia were evident on day 2 post-infection (**S4E Fig)**, infected mice exhibited significantly reduced hemoglobin levels by day 4 post-infection that returned to baseline by day 7 post-infection (**Fig 3I**). Further, Trichinella-induced mast cell responses, as determined by serum levels of Mcpt1, were evident by day 4 post-infection and persisted through day 7 post-infection (**Fig 3J**). These important data illustrate that the increased pool of Car1-expressing GFP positive cells in the periphery (**Fig 2I and 2J)** is associated with recovery from *Trichinella*-induced blood loss and the initiation of infection-induced mastocytosis.

### Expression of c-Kit and integrin β7 identify developmentally distinct subsets of Car1-expressing cells

**Fig 4:**
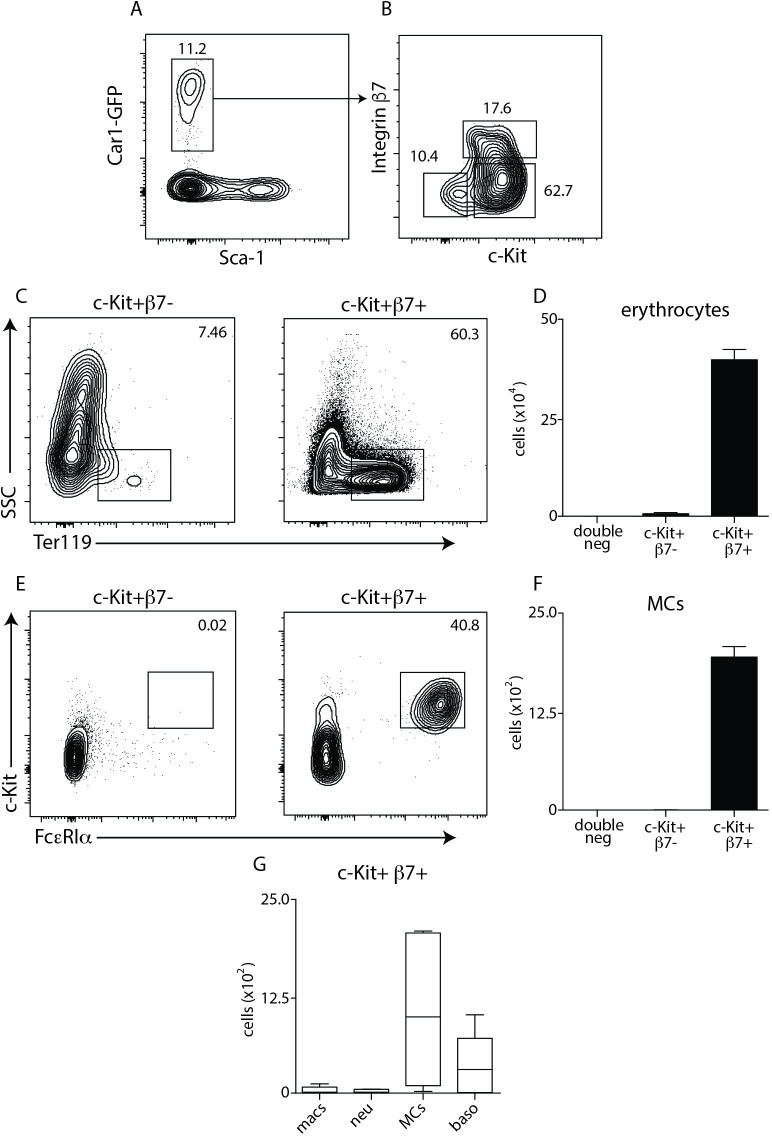
Surface expression of c-Kit and Integrin β7 identify functionally distinct subsets of Car1+ cells. (**A,B**), Flow cytometric analysis illustrating expression of c-Kit and integrin β7 on Car1+ cells from the bone marrow. Car1-GFP+ c-Kit+ β7- or Car1-GFP+ c-Kit+ β7+ cells were sort-purified from the bone marrow of mice and seeded into MethoCult and the percentages and total numbers of (**C,D**) erythrocytes and (**E,F**) mast cells (MCs) were evaluated by flow cytometric analysis post-culture. (**G**), The number of macrophages (macs), neutrophils (neu), MCs and basophils (baso) were quantified from Car1-GFP+ c-Kit+ β7+ seeded cultures. (A-G), Representative of at least 3 separate experiments. (G), Illustrates data pooled from 2 separate experiments.

Mast cell progenitors are reported to increase in the periphery following a helminth challenge where they contribute to mast cell-mediated immunity[7, 10, 12]. Our data suggest that in addition to supporting protective immunity, these progenitor cells may also possess the capacity to alleviate helminth-induced anemia. Traditionally, progenitors with mast cell potential have been identified by their expression of c-Kit and integrin β7. Therefore, we sought to test whether Car1-GFP+ cells expressed these well-described surface markers and to determine if mast cell and erythrocyte potential could be separated within the Car1-GFP+ compartment[25, 26]. While no Car1-GFP+ c-Kit- β7+ cells were identified, three readily identifiable Car1-positive populations were defined by c-Kit and integrin β7 expression: (Car1-GFP+ c-Kit- β7-), (Car1-GFP+ c-Kit+ β7-) and (Car1-GFP+ c-Kit+ β7+) cells (**Fig 4A and 4B**).

To evaluate the developmental capacity of these various populations we sort-purified them and cultured them in the same methylcellulose conditions described above. While Car1-GFP+ c-Kit- β7- cells failed to give rise to progeny of any cell type, Car1-GFP+ c-Kit+ β7- cells showed a slight capacity to develop into erythrocytes (**Fig 4C and 4D**) but not mast cells (**Fig 4E and 4F**). In contrast, Car1-GFP+ c-Kit+, β7+ cells showed substantial erythrocyte (**Fig 4C and 4D**) and mast cell (**Fig 4E and 4F**) potential. Further, when the progeny of Car1-GFP+ c-Kit+ β7+ were evaluated for the presence of other lineages, little to no macrophage and neutrophil development was detected (**Fig 4G**). Again, this is in contrast to Car1-GFP- cultures under the same conditions that showed robust development of macrophages and neutrophils (**S5A and S5B Fig**). Although basophils were consistently identified in cultures seeded with Car1-GFP+ c-Kit+ β7+ cells, they were found at low levels compared to mast cells (**Fig 4G**). These data suggest that Car1-GFP+ c-Kit+ β7+ cells are highly enriched for mast cell and erythrocyte potential that can support host protective responses to helminths.

### CD24a defines heterogeneity within Car1-expressing cells

**Fig 5:**
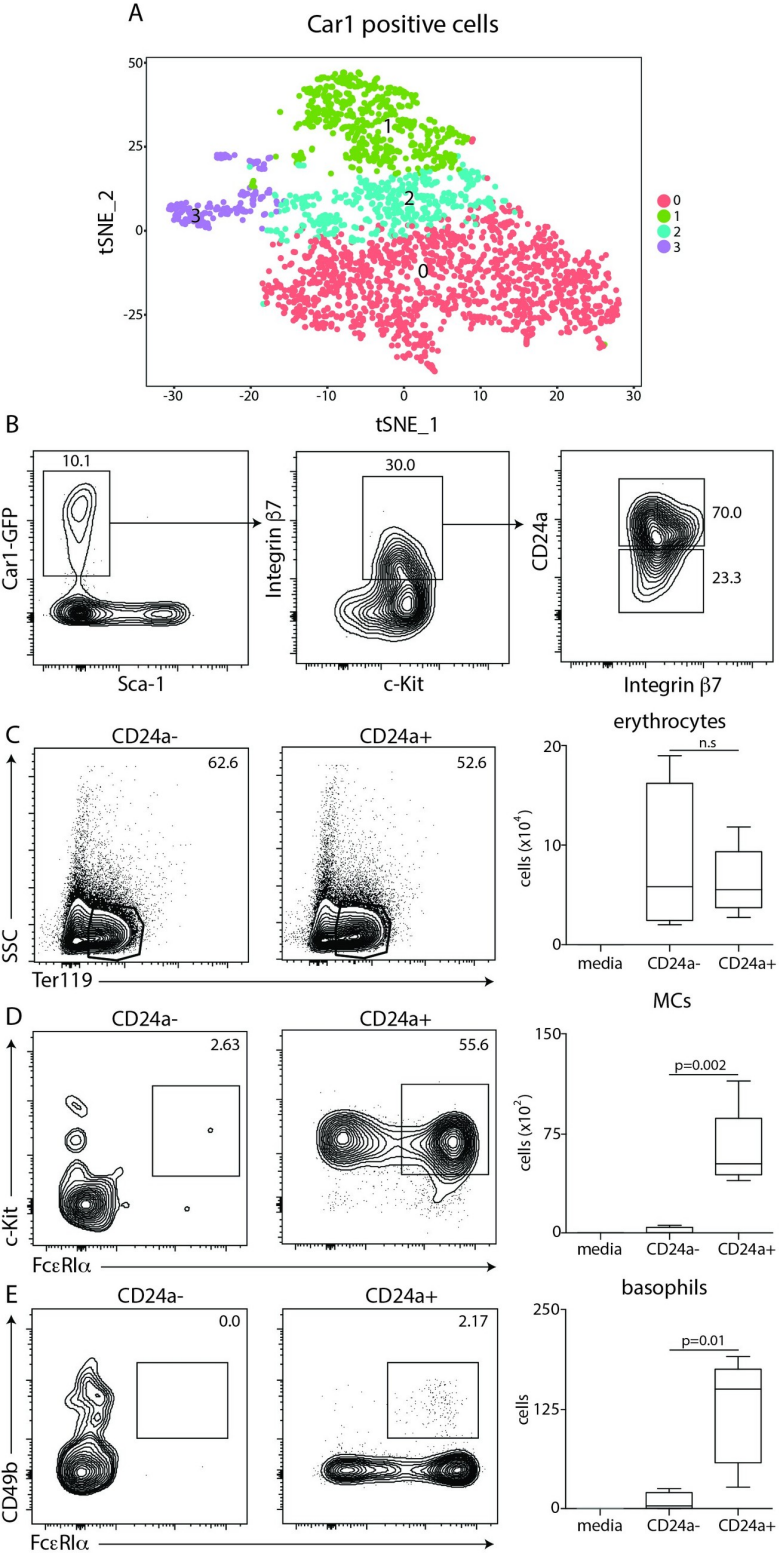
CD24a expression distinguishes erythrocyte potential from mast cell-erythrocyte potential within Car1-GFP positive cells. (**A**), TSNE plot illustrating defined clusters of cells generated by single cell RNA-seq of live CD45+ Car1-GFP+ cells sort-purified from the bone marrow of naïve mice. (**B**), Plots illustrating gating strategy used to fractionate bone marrow-resident Car1-GFP+ cells. Car1-GFP+ c-Kit+ β7+ CD24a- or Car1-GFP+ c-Kit+ β7+ CD24a+ cells were sort-purified from the bone marrow of mice and seeded into MethoCult and the percentage and total numbers of (**C**) erythrocytes, (**D**) mast cells (MCs) and (**E**) basophils were evaluated by flow cytometric analysis post-culture. (B-E), Results are representative of at least 3 separate experiments. Statistical analysis performed using a Student’s t-test.

Although our flow cytometric analysis revealed heterogeneity within the Car1-expressing compartment (**Fig 4A and 4B**), we sought to further identify strategies that might allow us to separate mast cell and erythrocyte potential within this compartment. Therefore, to gain further resolution and evaluate heterogeneity with the Car1-GFP+ compartment, we performed single cell RNA-sequencing on Car1-GFP+ cells. Single cell analysis of Car1-GFP+ cells revealed the presence of 4 distinct clusters (**Fig 5A**). When heat maps were generated to determine the genes that define each cell cluster, it was found that CD24a was a defining gene of cluster 1 (**S6A Fig**). In addition to *CD24a*, cluster 1 was also defined by expression of the erythrocyte-associated genes heme binding protein 1 (*Hebp1*) and the hemoglobin chains Hba-a1 and Hbb-bt, suggesting that cluster 1 was likely to contain erythrocyte potential (**S6B Fig**). Although cluster 1 expressed other genes including glycophorin A (*Gypa*), cathespin E (*Ctse*) and cyclin D3 (*Ccnd3*), none of the identified genes were strong indicators of mast cell potential. However, the identification of CD24a (**S6A Fig** red arrow) as a marker of heterogeneity within the Car1-GFP+ compartment provided the opportunity to further separate Car1-expressing cells in order to test their developmental capacity. To evaluate this possibility, we first determined if CD24a surface expression could be detected on Car1-GFP+ cells by flow cytometric analysis. Critically, CD24a was found to be expressed on approximately 50% of Car1-expressing cells, suggesting that CD24a can be used for sort-purification strategies and may allow us to more definitely determine the developmental potential of these cellular subsets and how they may operate post-helminth infection (**S6B Fig**). Importantly, the patterns of c-Kit, β7 and CD24a within the Car1-GFP+ compartment also remained consistent following a *T*. *spiralis* challenge (**S6C Fig**).

### CD24a expression separates cells with erythrocyte potential from cells with erythrocyte/mast cell potential

Our previous data demonstrated that Car1-GFP+ c-Kit+ β7+ cells were highly enriched for mast cell and erythrocyte potential (**Fig 4C–4F**). Therefore, we first sought to determine if we could use CD24a to further separate this compartment. Flow cytometric analysis demonstrated that approximately 70% of Car1-GFP+ c-Kit+ β7+ cells expressed CD24a (**Fig 5B**). To further evaluate the developmental potential of these distinct populations we sort-purified Car1-GFP+ c-Kit+ β7+ CD24a- and Car1-GFP+ c-Kit+ β7+ CD24a+ cells and evaluated their ability to generate erythrocytes and/or mast cells using our *in vitro* culture system. While CD24a- cells showed a substantial capacity to generate erythrocytes (**Fig 5C**), very few mast cells could be detected post-culture (**Fig 5D**). In contrast, CD24a+ cells demonstrated the capacity to generate substantial erythrocyte and mast cell populations (**Fig 5C and 5D**). Our previous cultures demonstrated that Car1-GFP+ c-Kit+ β7+ cells were also capable of generating small but readily detectable CD49b+ FcεRIα+ basophil populations (**Fig 4G**). Consistent with these data Car1-GFP+ c-Kit+ β7+ cells possessed a very limited but reproducible capacity to generate basophils while other cell lineages were not consistently detected (**Fig 5E**). Collectively, these data suggest that lineage negative Car1-GFP+ c-Kit+ β7+ CD24a+ cells may represent unique progenitor cells with erythrocyte, mast cell and limited basophil potential.

### CD24a+ Car1-expressing cells are potent mast cell and erythrocyte precursors

**Fig 6:**
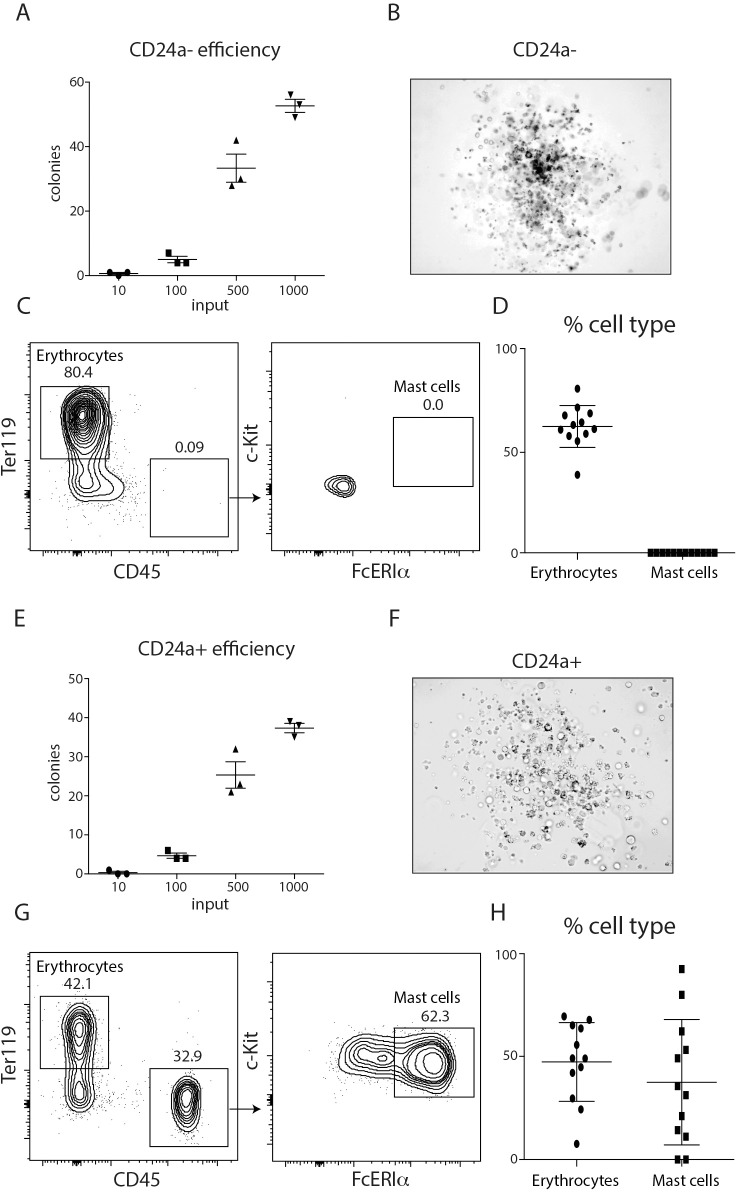
Car1+ c-Kit+ β7+ CD24a+ cells are mast cell-erythrocyte progenitors. (**A**), Car1-GFP+ c-Kit+ β7+ CD24a- cells were sort-purified, seeded into MethoCult and the number of colonies were quantified post-culture. (**B**), Representative colony from Car1-GFP+ c-Kit+ β7+ CD24a- seeded cultures. (**C**), Single colonies were isolated from Car1-GFP+ c-Kit+ β7+ CD24a- seeded cultures and the presence of erythrocytes and mast cells were determined by flow cytometric analysis. (**D**), The values for the gated cell populations illustrated in (**C**) were plotted for each colony isolated. (**E**), Car1-GFP+ c-Kit+ β7+ CD24a+ cells were sort-purified, seeded into MethoCult and the number of colonies were quantified post-culture. (**F**), Representative colony from Car1-GFP+ c-Kit+ β7+ CD24a+ seeded cultures. (**G**), Single colonies were isolated from Car1-GFP+ c-Kit+ β7+ CD24a+ seeded cultures and the presence of erythrocytes and mast cells were determined by flow cytometric analysis. (**H**), The values for the gated cell populations illustrated in (**G**) were plotted for each colony isolated. (A-G), Representative of at least 3 separate experiments. At least 10 single colonies were isolated per experiment.

To further evaluate the developmental capacity of Car1-GFP+ c-Kit+ β7+ CD24a- and CD24a+ cells and their potential roles following a helminth infection, they were sort-purified and plated in permissive media at various dilutions to evaluate their plating efficiencies and the makeup of individual colonies. When Car1-GFP+ c-Kit+ β7+ CD24a- populations were cultured, approximately 30–40 colonies were obtained from 500 input cells (**Fig 6A**) and their appearance was consistent with that of erythrocytes (**Fig 6B**). Flow cytometric analysis of individual colonies further confirmed the presence of Ter119-expressing cells in individual colonies (**Fig 6C**). Consistent with our previous analysis, very few CD45+ cells were detected and no mast cells were identified in any of the individual colonies isolated from cultures of CD24a- cells (**Fig 6C and 6D**). When Car1-GFP+ c-Kit+ β7+ CD24a+ populations were cultured, approximately 20–30 colonies were obtained from 500 input cells (**Fig 6E**), and their appearance was consistent with the presence of mixed cell types (**Fig 6F**). Similar to CD24a- input populations, flow cytometric analysis of individual colonies confirmed the presence of Ter119-expressing cells in cultures seeded with CD24a+ cells (**Fig 6G**). However, in contrast to CD24a- cultures, individual colonies isolated from cultures of CD24a+ cells contained a high percentage of CD45+ mast cells (**Fig 6G and 6H**). Importantly, solitary mast cell potential that was uncoupled from erythrocyte potential was not detected. These data further support the notion that Car1-GFP+ c-Kit+ β7+ CD24a- cells represent committed erythrocyte precursors while Car1-GFP+ c-Kit+ β7+ CD24a+ cells possess both mast cell and erythrocyte potential. Collectively, these studies suggest that Car1-expressing progenitor cells are capable of promoting host protection to *Trichinella* by supporting protective mast cell responses and erythropoiesis to alleviate infection-induced anemia.

### Car1-expressing HPCs support mast cell and erythrocyte responses post-*Trichinella* infection

**Fig 7:**
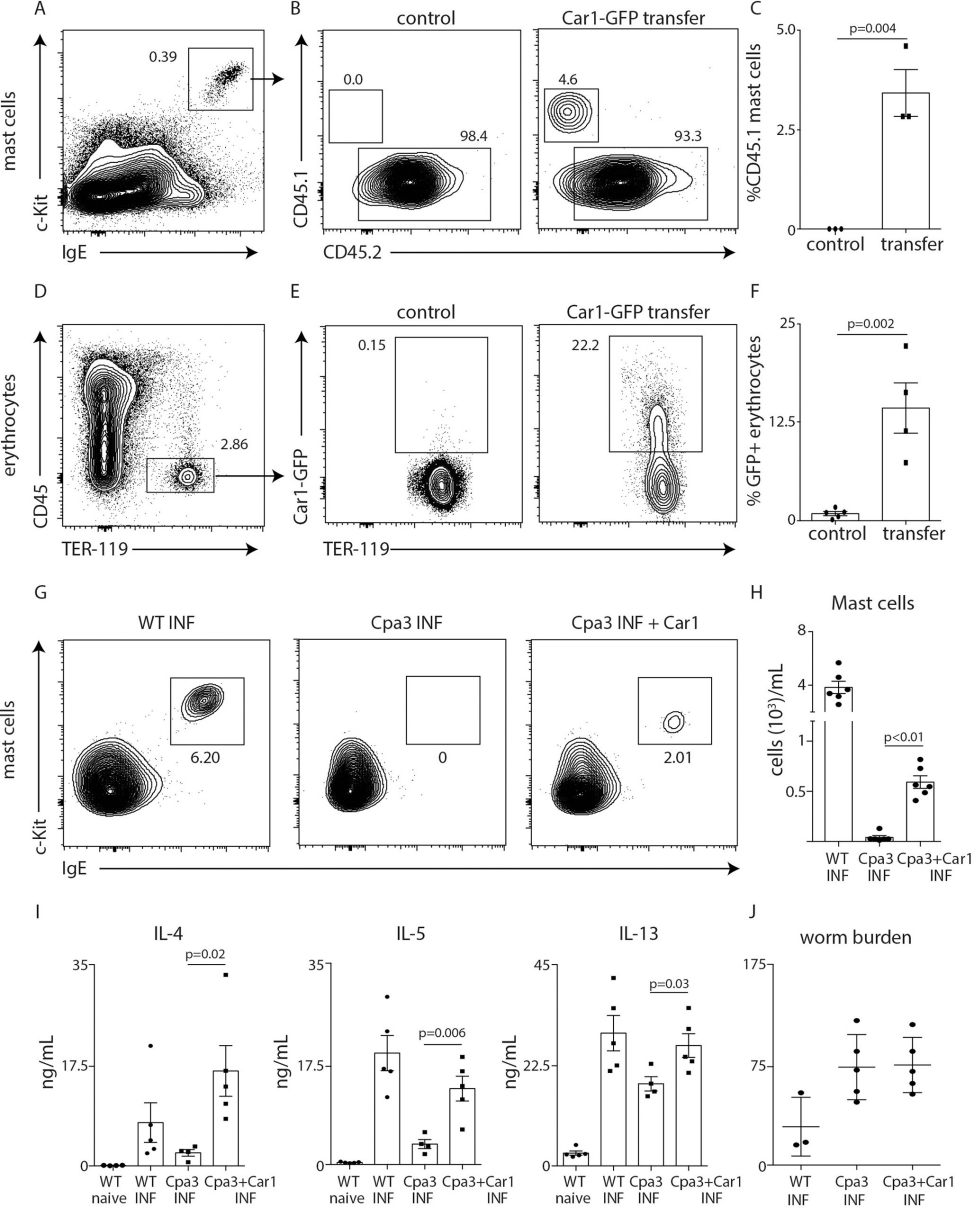
Car1-GFP+ HPCs support mast cell and erythrocyte development post-*Trichinella* infection. Car1-GFP+ cells were sort-purified from the bone marrow of naïve CD45.1 congenic mice and adoptively transferred (i.p.) into CD45.2 hosts on day 4 and 5 post-infection. On day 10 post-infection (**A**) peritoneal mast cells were identified as CD45+ c-Kit+ IgE+ and (**B**) the percent of CD45.1 and CD45.2 mast cells were determined in control mice and mice receiving cell transfers. (**D**), Erythrocytes in the peritoneal exudate identified as CD45- TER119+ and (**E**) the percent of erythrocytes expressing Car1-GFP were determined in control mice and mice receiving cell transfers. (**C, F**), The percentage of identified cells were quantified. WT and Cpa3-Cre mice were infected with *T*. *Spiralis*. Groups of Cpa3-Cre mice received transfers of Car1-GFP+ cells post-infection and (**G, H**) peritoneal mast cells, (**I**) IL-4, IL-5 and IL-13 levels of stimulated mLNs (**J**) and worm burdens were determined. (A,D,G), Representative plots illustrating gating strategies. (A-F) Are representative of at least 3 separate experiments. (G,H), Results are representative of 2 separate experiments comprised of 10 biological replicates total. Statistical analysis performed using a Student’s t-test.

To further evaluate the role that Car1-expressing HPCs play following a helminth infection, we transferred CD45.1+ Car1-GFP+ cells from naïve mice into *Trichinella*-infected CD45.2+ hosts and investigated their ability to contribute to mast cell and erythrocyte development. Consistent with their ability to support mast cell responses post-infection, CD45.1+ cells were readily identified within the c-Kit+ FcεRIα+ mast cell gate (**Fig 7A–7C**). Although CD45 expression is lost during the maturation process of erythrocytes[27], we were able to detect Car1-GFP expression within the CD45- Ter119+ erythrocyte compartment (**Fig 7D–7F**). Collectively, these data confirm our *in vitro* assays and demonstrate that Car1-GFP+ HPCs support mast cell and erythrocyte developmental in the context of a *Trichinella* infection. Next, we sought to evaluate if Car1-GFP+ cells are sufficient to promote type 2 cytokine responses. To test this, we infected Cpa3-Cre mice, which lack mast cells[28], with *Trichinella* and adoptively transferred Car1-GFP+ cells. As expected, transfer of Car1-GFP+ cells resulted in a small but detectable mast cell population as compared to non-transferred Cpa3-Cre mice (**Fig 7G and 7H**). Critically, while Cpa3-Cre mice exhibited significantly reduced IL-4, IL-5 and IL-13 production post-*Trichinella* infection, those mice receiving Car1-GFP+ cells exhibited significantly increased type 2 cytokine production. Despite partially restored mast cell responses and fully restored type 2 cytokine production, mice receiving Car1-GFP+ cells had equivalent worm burdens at the timepoint evaluated (**Fig 7I and 7J**). It is possible that more frequent cell transfers are required to fully restore mast cell responses and protective immunity. However, these data demonstrate that Car1-expressing progenitors are sufficient to promote type 2 cytokine responses in addition to promoting erythrocyte development.

## Discussion

Host-protective responses to helminth parasites require the initiation of distinct pathways that simultaneously promote worm clearance and the resolution of helminth-induced wounding. While it has long been appreciated that type 2 cytokines initiate these important events through the activation of immune cells, such as M2 macrophages, our understanding of the hematopoietic pathways that support these processes remains poorly understood. Here we have defined a small population of carbonic anhydrase 1 (Car1)-expressing HPCs that exists at steady-state and increase their presence in the periphery following a *T*. *spiralis* infection. By using a combination of discovery-based and reductionist approaches we have determined that Car1-expressing HPCs are comprised of an erythrocyte progenitor and a dual mast cell/erythrocyte progenitor that can be distinguished by its expression of Car1, c-Kit, integrin β7 and CD24a. Importantly, we show that Car1-expressing HPC responses are associated with a recovery from infection-induced anemia and the initiation of mastocytosis. Finally, adoptive transfer studies demonstrate that Car1-expressing HPCs support both mast cell and erythrocyte development post-*Trichinella* infection and provide substantial insight into the hematopoietic pathways that promote host-protective responses to parasitic worms.

As new technologies and techniques are developed, our ability to understand hematopoiesis and lineage commitment events continues to advance. Single cell RNA-sequencing platforms now allow for a more thorough evaluation of heterogeneity within previously defined HPC populations[29]. As a result, emerging studies are beginning to reshape traditional models of the hematopoietic tree and are identifying previously unappreciated developmental similarities between distinct lineages. For example, recent human and murine studies suggest that mast cells and erythrocytes share common developmental pathways[13–15]. Despite these advances, the contributions of mast cell/erythrocyte progenitors to infection-induced inflammation remain poorly defined.

The studies presented above suggest a beneficial role for Car1-expressing HPCs in the context of a T. *spiralis* infection. Mast cells are well recognized for their ability to promote parasite clearance, and anemia is a common feature of helminth infections[2, 25]. Therefore, a progenitor cell with a dual capacity to generate mast cells and erythrocytes may be ideally suited to maintain these cell types under homeostatic conditions and also promote host protective responses to a broad range of infections. Therefore, it will be important to evaluate the relative contributions of Car1-expressing HPCs in promoting mast cell/erythrocyte development at baseline and in response to other helminths in order to fully evaluate the biologic functions of these distinct cells. Further, given that many of the studies presented here evaluated the developmental capacity of these cells at the steady-state, it will also be important to determine how these developmental patterns change in response to diverse inflammatory settings.

In addition to the host protective responses mentioned above, it will also be interesting to evaluate the role of Car1-expressing progenitors in models of allergic inflammation or dysregulated mast cell development. Interestingly, recent human studies have highlighted an association between allergic disease and anemia[30–32]. Further, it is well established that patients suffering from mastocytosis also present with severe anemia [33, 34]. Although several plausible explanations have been proposed to explain these associations, including the possibility that mast cell-derived effector molecules kill erythrocytes, it is possible that they might be the result of a defective development branch point that forces the overabundance of mast cells to the detriment of erythrocytes. The identification of Car1 as a reliable marker for mast cell/erythrocyte progenitors provides an exciting opportunity to study these important pathways.

It will also be interesting to further dissect the importance of Car1 enzymatic function in the lineage commitment of these distinct cells. Carbonic anhydrases are a family of metabolic enzymes that are known to regulate pH and CO_2_ homeostasis and are most commonly referenced for their highly efficient metabolic properties[35]. However, emerging studies suggest that the expression patterns of specific Car family members represent the defining features of several lineages. For example, our work and that of others suggest that Car1 and Car2 are important components of erythrocyte and mast cell development[12, 16]. Additional studies have also demonstrated that Car4 represents the lineage-identifying gene of alveolar macrophages[36]. Finally, a recent report has defined a role for Car6b in epigenetically controlling proinflammatory cytokine production and bacterial clearance by macrophages[37]. Collectively, these studies strongly suggest that Car enzyme family members possess unique properties in addition to their known metabolic functions. Therefore, gaining a better understanding of their expression patterns and unique functions may be of great therapeutic interest.

In summary, better defining the developmental origin of mast cells and erythrocytes is of great scientific and clinical relevance. Mast cells are of ancient origin and their presence has been remarkably conserved throughout evolution, an indicator of their critical importance[25]. Mast cells are strategically positioned at barrier surfaces and have been described as sentinels of the immune system capable of quickly promoting inflammation when barrier integrity is compromised[25]. In most instances, like those seen in the context of a helminth infection, the disruption of a barrier surface is the result of wounding that is associated with bleeding. In the studies presented above we have described a unique progenitor cell population that is specialized to support mast cell responses while simultaneously mitigating the effects of blood loss. Although important studies are needed to determine how these HPCs operate in the context of other forms of inflammation, this work may inform important therapeutic strategies to address diverse clinical situations by targeting Car1.

## Materials and methods

### Mice

C57BL(6) WT mice (stock #000664) and Cpa3-Cre mice (stock #026828) were purchased from The Jackson Laboratory. Mice were maintained in specific pathogen–free facilities at the Rutgers New Jersey Medical School. Car1-GFP reporter mice on a C57BL(6) background were created by Biocytogen (Worcester, MA). All protocols were approved by the Rutgers Institutional Animal Care and Use Committee (IACUC) protocol number 17025.

### *Trichinella spiralis* infections, blood hemoglobin analysis, cell transfers, and BrdU incorporation

Methods for maintenance, recovery, infection and isolation of *Trichinella spiralis* larvae were performed as previously described [38]. Mice were infected with 500 *Trichinella* muscle larvae by oral gavage; mice were sacrificed at 4, 7 or 10 days post-infection. At necropsy, single cell suspensions of bone marrow, mLNs and spleens were prepared for flow cytometric analysis. Blood hemoglobin (Hb) levels were monitored as previously published [39]. Briefly, 2μl of tail blood was placed in 500μl Drabkin’s Solution (Sigma-Aldrich D5941), in duplicate. 100μl of the diluted blood was plated into a 96-well plate and read at a wavelength of 540nm in a microplate reader. Hb concentrations were then calculated along a standard curve of human Hb according to Sigma-Aldrich’s product information.

For cell transfers, bone-marrow cell suspensions were prepared from naïve CD45.1+ Car1-GFP reporter mice and Lin- Car1-GFP expressing cells were sort-purified using a FACSAria II or FACSAria Fusion flow cytometer (BD). Then, 1.0–3.0 x 10^5 cells were adoptively transferred i.p. into CD45.2 WT or Cpa3-Cre host mice that were infected with *T*.*spiralis* 4 and 5 days before the cell transfer. 10 days post-*Trichinella* infection, peritoneal exudate cells were isolated as previously described [40] and mast cells and erythrocytes populations were evaluated by flow cytometry. mLNs cell suspensions were prepared and restimulated with anti-CD3 and anti-CD28 antibodies for 48 hours and supernantant levels of IL-4, IL-5, and IL-13 were evaluated by ELISA.

Cell proliferation was evaluated via BrdU-incorporation analysis following manufacturer's instructions (BD Pharmigen, 552598). Briefly, naive or *T*. *spiralis*-infected Car1-GFP reporter mice were administered a single injection of 1mg BrdU i.p. on day 3 post-infection. Bone marrow and spleen were collected 24 hours post-injection and the percentage and total number of Car1-GFP+ BrdU+ cells were evaluated via flow cytometric analysis.

### Flow cytometry and cell sorting

Cells were stained with anti-mouse fluorescently labeled conjugated antibodies: I-A/I-E (M5/114.15.2), CD115 (AFS98) and CD127 (A7R34) from Biolegend; or Sca-1 (D7), CD34 (RAM34), FcεRI (MAR-1), CD16/32 (93), CD11c (N418), CD135 (Flt3, A2F10.1), Ly6C (HK1.4), and CD45 (104) from eBioscience; or Ter119 (TER-119), FcεRI (MAR-1), CD5 (53–7.3) and γδTCR (eBioGL3) from Invitrogen; or CD49b (DX5), c-Kit (2B8), CD125 (T21), Integrin β7 (FIB504), CD3 (145-2C11), CD19 (1D3), CD11b (MI/70), NK1.1 (PK136), Ly6G (1A8), CD45 (30-F11), CD11b (M1/70), CD24a (M169) and Ly6G (1A8) from BD Biosciences. Live cells were distinguished by staining with DAPI (Molecular Probes). The lineage marker staining consisted of CD3, CD19, CD11b, CD11c, NK1.1, γδTCR, CD5, Ly6G and Ter119. Hematopoietic progenitors were characterized as follows: MCPs (Lin- Sca1- c-Kit+ Integrin β7+), MEPs (Lin- Sca1- c-Kit+ CD34- CD16/32-), Flt3+ or Flt3- CMPs (Lin- Sca1- c-Kit+ CD34+ CD16/32- CD115lo), MDPs (Lin- Sca1- c-Kit+ CD34+ CD16/32- Flt3+ CD115hi), GMPs (Lin- Sca1- c-Kit+ CD34+ CD16/32hi Ly6C-CD115lo), GPs (Lin- Sca1- c-Kit+ CD34+ CD16/32hi Ly6C+CD115lo), MP+ cMoP (Lin- Sca1- c-Kit+ CD34+ CD16/32hi Ly6C+CD115hi), BaPs (Lin- Sca1- c-Kit+ CD34+ CD16/32hi FcER+), and EoPs (Lin- Sca1- CD34+ c-Kit- CD125+). Samples were acquired on a LSR II or LSR FORTESSA X-20 flow cytometer (BD) and analyzed using FlowJo software (v10.4.1 Tree Star). Cell sorting was performed using a FACSAria II or FACSAria Fusion flow cytometer (BD). Car1-GFP+ cells were sort purified as CD45+ Lin- Sca-1- and subsequently on Integrin β7, c-Kit and CD24a expression when indicated. Live cells were distinguished by staining with DAPI (Molecular Probes). Car1-GFP cellular distribution was determined using AMNIS Imagestream^X^ MarkII. Hoescht was used to fluorescently label DNA prior to AMNIS Imagestream^X^ MarkII analysis.

### Isolation and processing of immune cells for flow cytometry, cell sorting and single cell RNA-sequencing

Bone marrow (BM) cells from the femur and tibia or splenocytes were isolated and red blood cells were lysed. Prior to sorting, flow cytometry or single cell RNA-sequencing, cells were enriched using MagniSort Streptavidin Negative Selection Beads kit (Invitrogen). Briefly, after red blood cell lysis, BM cells and splenocytes were washed with Magnisort wash buffer and then incubated with anti-mouse biotinylated antibodies: CD11b (M1/70) and TCRβ (H57-597) from eBioscience; CD19 (eBio1D3 (1D3)) from Invitrogen; CD8b (53–5.8) from Biolegend. The cells were then incubated with magnetic beads conjugated to streptavidin and placed within a magnetic field. The unlabeled cells (in the supernatant) were collected, washed with FACS buffer and stained with the appropriate fluorescent antibody cocktail.

### Methocult cultures

Sort-purified bone marrow or splenic cells (1 x 10^2^–10 x 10^3^) were added to MethoCult (M3434)[23, 24] supplemented with Penicillin/Streptomycin (Gibco). Mast cell (CD45+ c-Kit+ FcεRIα+), erythrocyte (CD45- Ter119+), macrophage (CD45+ CD11b+ CD115+), basophil (CD45+ CD49b+ FcεRIα+) and neutrophil (CD45+ CD11b+ Ly6G+) populations were evaluated on day 7 post-culture using flow cytometry.

### Single cell RNA-sequencing

Bone marrow-resident cells were sort-purified on live CD45+ CD3- CD19- Ly6G- or on live CD45+ Car1+ cells from three biological replicates and pooled. 10,000 sort-purified cells were then processed using the 10X Genomics Chromium Controller. Cell suspensions were loaded onto the Chromium Single Cell A Chip for cell lysis and barcoding. RNA from individual cells was reverse transcribed and sequencing libraries prepared using the Chromium Single Cell 3’ Library Kit v2 following the manufacturers protocol. Samples were sequenced using an Illumina NextSeq 550 with standard 10X Genomics Configuration (26bpx98bp). After sequencing, raw bcl files were processed using the cellranger mkfastq command for sample demultiplexing and conversion to.fastq files, followed by cellranger count for cell barcode and UMI deconvolution as well as mapping to the respective reference genome. Processed digital gene expression matrices were imported into R studio for analysis using the Seurat package. Samples were aligned along common sources of variation and compared using canonical correlation analysis to identify unique clusters of cells within the samples. Marker genes for each sample and cluster were identified and used for generation of downstream plots within the Seurat package. All packages are maintained to be best in class and are regularly updated to their most recent release. These data have been uploaded into NCBI (GSE3105).

### Publicly available data analysis

Data for the bone marrow samples were downloaded from NCBI (GSE108097). In total, the mapped digital gene expression files from three bone marrow samples were downloaded for analysis, GSM2906396, GSM2906399, and GSM2906400. Digital gene expression matrices were imported into R studio for analysis using the Seurat package. Samples were aligned along common sources of variation and compared using canonical correlation analysis to identify unique clusters of cells within the samples. Marker genes for each sample and cluster were identified and used for generation of downstream plots within the Seurat package. All packages are maintained to be best in class and are regularly updated to their most recent release.

### Ethics statement

The studies performed were governed by protocol 17025 as approved by the IACUC committee of New Jersey Medical School animal studies were compliant with all applicable provisions established by the Animal Welfare Act and the Public Health Services (PHS) Policy on the Humane Care and Use of Laboratory Animals.

### Statistics

Results are shown as mean ± SD. Statistical analysis was performed using Student’s t-tests in Prism (version 6.07; GraphPad Software).

## Supporting information

S1 Fig Heat map illustrating the top marker genes defining the 20 distinct clusters identified by single cell RNA-seq analysis of bone marrow resident cells.
(TIF)

S2 Fig (**A**), Car1-GFP expression within lineage- and lineage+ compartments in the spleen was determined by flow cytometric analysis. (**B**), Expression levels of progenitor-associated markers on Car1-GFP+ cells in the bone marrow. Results are representative of 3 separate experiments.
(TIF)

S3 Fig (**A**), Car1-GFP reporter mice were infected with *T*. *spiralis* and treated with BrdU and the (**B,C**) percentage and total Car1-GFP+ cells in the spleen were determined on day 4 post infection. Results are representative of 2 separate experiments comprised of 10 biological replicates total. Statistical analysis performed using a Student’s t-test.
(TIF)

S4 Fig Car1-GFP+ or Car1-GFP- cells were sort-purified from the bone marrow of mice and seeded into MethoCult and the numbers of (**A**) macrophages and (**B**) neutrophils were evaluated by flow cytometric analysis post-culture. (**C,D**), Car1+ or Car1- cells were sort-purified from the spleens of mice and seeded into MethoCult with hematopoietic cytokines. Representative plots illustrating the percentage of mast cells (MCs) and erythrocytes identified by flow cytometric analysis post-culture. (**E**), Hemoglobin (Hb) levels were quantified on day 2 post-*Trichinella* infection. Results are representative of at least 3 separate experiments.
(TIF)

S5 Fig Car1-GFP+ c-Kit+ β7+, Car1-GFP+ c-Kit- β7-, Car1-GFP+ c-Kit+ β7-, or Car1-GFP- c-Kit+ β7+ cells were sort-purified from the bone marrow of mice and seeded into MethoCult and the total numbers of (**A**) macrophages and (**B**) neutrophils were evaluated by flow cytometric analysis post-culture. Results are representative of at least 3 separate experiments.
(TIF)

S6 Fig (**A**), Heat map illustrating the top marker genes defining the 4 distinct clusters identified by single cell RNA-seq analysis of bone marrow resident GFP+ cells. (**B**), Bone marrow resident Car1-GFP+ cells were evaluated for CD24a expression. (**C**), Expression patterns of lineage markers, c-Kit, integrin β7 and CD24a were evaluated on bone marrow-resident Car1-GFP+ cells 7 days post-*T*. *spiralis* infection.
(TIF)

S1 Table Markers defining each of the 20 clusters identified in Fig 1A. (PDF)

## References

[1] Schistosomiasis and soil-transmitted helminthiases: number of people treated in 2015. Wkly Epidemiol Rec. 2016;91(49–50):585–95. . 27934297

[2] Jourdan PM, Lamberton PHL, Fenwick A, Addiss DG. Soil-transmitted helminth infections. Lancet. 2017 10.1016/S0140-6736(17)31930-X . 28882382

[3] Gause WC, Wynn TA, Allen JE. Type 2 immunity and wound healing: evolutionary refinement of adaptive immunity by helminths. Nature reviews Immunology. 2013;13(8):607–14. 10.1038/nri3476 23827958 PMC3789590

[4] Gieseck Iii RL, Wilson MS, Wynn TA. Type 2 immunity in tissue repair and fibrosis. Nature Reviews Immunology. 2017;18:62 10.1038/nri.2017.90 28853443

[5] Allen JE, Wynn TA. Evolution of Th2 immunity: a rapid repair response to tissue destructive pathogens. PLoS Pathog. 2011;7(5):e1002003 Epub 2011/05/19. 10.1371/journal.ppat.1002003 21589896 PMC3093361

[6] Lloyd CM, Snelgrove RJ. Type 2 immunity: Expanding our view. Science immunology. 2018;3(25). Epub 2018/07/08. 10.1126/sciimmunol.aat1604 . 29980619

[7] Siracusa MC, Saenz SA, Wojno ED, Kim BS, Osborne LC, Ziegler CG, et al Thymic stromal lymphopoietin-mediated extramedullary hematopoiesis promotes allergic inflammation. Immunity. 2013;39(6):1158–70. 10.1016/j.immuni.2013.09.016 24332033 PMC3959827

[8] Saenz SA, Siracusa MC, Perrigoue JG, Spencer SP, Urban JF Jr., Tocker JE, et al IL25 elicits a multipotent progenitor cell population that promotes T(H)2 cytokine responses. Nature. 2010;464(7293):1362–6. 10.1038/nature08901 20200520 PMC2861732

[9] Hui CC, McNagny KM, Denburg JA, Siracusa MC. In situ hematopoiesis: a regulator of TH2 cytokine-mediated immunity and inflammation at mucosal surfaces. Mucosal Immunol. 2015;8(4):701–11. 10.1038/mi.2015.17 . 25783967

[10] Liu AY, Dwyer DF, Jones TG, Bankova LG, Shen S, Katz HR, et al Mast Cells Recruited to Mesenteric Lymph Nodes during Helminth Infection Remain Hypogranular and Produce IL-4 and IL-6. The Journal of Immunology. 2013;190(4):1758–66. 10.4049/jimmunol.1202567 23319739 PMC3563837

[11] Wakelin D. Trichinella spiralis: immunity, ecology, and evolution. The Journal of parasitology. 1993;79(4):488–94. Epub 1993/08/01. . 8331470

[12] Henry EK, Sy CB, Inclan-Rico JM, Espinosa V, Ghanny SS, Dwyer DF, et al Carbonic anhydrase enzymes regulate mast cell-mediated inflammation. The Journal of experimental medicine. 2016;213(9):1663–73. 10.1084/jem.20151739 27526715 PMC4995079

[13] Tusi BK, Wolock SL, Weinreb C, Hwang Y, Hidalgo D, Zilionis R, et al Population snapshots predict early haematopoietic and erythroid hierarchies. Nature. 2018;555:54 10.1038/nature25741 https://www.nature.com/articles/nature25741 - supplementary-information. 29466336 PMC5899604

[14] Zheng S, Papalexi E, Butler A, Stephenson W, Satija R. Molecular transitions in early progenitors during human cord blood hematopoiesis. Molecular systems biology. 2018;14(3):e8041 Epub 2018/03/17. 10.15252/msb.20178041 29545397 PMC5852373

[15] Li Z, Liu S, Xu J, Zhang X, Han D, Liu J, et al Adult Connective Tissue-Resident Mast Cells Originate from Late Erythro-Myeloid Progenitors. Immunity. 2018;49(4):640–53 e5. Epub 2018/10/18. 10.1016/j.immuni.2018.09.023 . 30332630

[16] Villeval JL, Testa U, Vinci G, Tonthat H, Bettaieb A, Titeux M, et al Carbonic anhydrase I is an early specific marker of normal human erythroid differentiation. Blood. 1985;66(5):1162–70. Epub 1985/11/01. . 3931725

[17] Butler A, Hoffman P, Smibert P, Papalexi E, Satija R. Integrating single-cell transcriptomic data across different conditions, technologies, and species. Nature Biotechnology. 2018;36(5):411–20. 10.1038/nbt.4096 29608179 PMC6700744

[18] Sullivan BM, Liang HE, Bando JK, Wu D, Cheng LE, McKerrow JK, et al Genetic analysis of basophil function in vivo. Nature immunology. 2011;12(6):527–35. Epub 2011/05/10. 10.1038/ni.2036 21552267 PMC3271435

[19] Schechter AN. Hemoglobin research and the origins of molecular medicine. Blood. 2008;112(10):3927–38. Epub 2008/11/08. 10.1182/blood-2008-04-078188 18988877 PMC2581994

[20] Nishida K, Uchida R. Role of Zinc Signaling in the Regulation of Mast Cell-, Basophil-, and T Cell-Mediated Allergic Responses. Journal of immunology research. 2018;2018:5749120 Epub 2019/01/01. 10.1155/2018/5749120 30596108 PMC6286780

[21] Han X, Wang R, Zhou Y, Fei L, Sun H, Lai S, et al Mapping the Mouse Cell Atlas by Microwell-Seq. Cell. 2018;172(5):1091–107 e17. Epub 2018/02/24. 10.1016/j.cell.2018.02.001 . 29474909

[22] Innocenti A, Scozzafava A, Parkkila S, Puccetti L, De Simone G, Supuran CT. Investigations of the esterase, phosphatase, and sulfatase activities of the cytosolic mammalian carbonic anhydrase isoforms I, II, and XIII with 4-nitrophenyl esters as substrates. Bioorg Med Chem Lett. 2008;18(7):2267–71. Epub 2008/03/21. 10.1016/j.bmcl.2008.03.012 . 18353640

[23] Sata M, Saiura A, Kunisato A, Tojo A, Okada S, Tokuhisa T, et al Hematopoietic stem cells differentiate into vascular cells that participate in the pathogenesis of atherosclerosis. Nature medicine. 2002;8:403 10.1038/nm0402-403 11927948

[24] Visnjic D, Kalajzic Z, Rowe DW, Katavic V, Lorenzo J, Aguila HL. Hematopoiesis is severely altered in mice with an induced osteoblast deficiency. Blood. 2004;103(9):3258–64. Epub 2004/01/17. 10.1182/blood-2003-11-4011 . 14726388

[25] Voehringer D. Protective and pathological roles of mast cells and basophils. Nature reviews Immunology. 2013;13(5):362–75. Epub 2013/04/06. 10.1038/nri3427 . 23558889

[26] Dahlin JS, Hallgren J. Mast cell progenitors: origin, development and migration to tissues. Mol Immunol. 2015;63(1):9–17. Epub 2014/03/07. 10.1016/j.molimm.2014.01.018 . 24598075

[27] Mikkola HK, Fujiwara Y, Schlaeger TM, Traver D, Orkin SH. Expression of CD41 marks the initiation of definitive hematopoiesis in the mouse embryo. Blood. 2003;101(2):508–16. Epub 2002/10/24. 10.1182/blood-2002-06-1699 . 12393529

[28] Lilla JN, Chen C-C, Mukai K, BenBarak MJ, Franco CB, Kalesnikoff J, et al Reduced mast cell and basophil numbers and function in Cpa3-Cre; Mcl-1fl/fl mice. Blood. 2011;118(26):6930–8. Epub 10/14. 10.1182/blood-2011-03-343962 . 22001390 PMC3245213

[29] Wang Y, Navin NE. Advances and applications of single-cell sequencing technologies. Mol Cell. 2015;58(4):598–609. Epub 2015/05/23. 10.1016/j.molcel.2015.05.005 26000845 PMC4441954

[30] Drury KE, Schaeffer M, Silverberg JI. Association Between Atopic Disease and Anemia in US Children. JAMA pediatrics. 2016;170(1):29–34. Epub 2015/12/01. 10.1001/jamapediatrics.2015.3065 . 26619045

[31] Nwaru BI, Hayes H, Gambling L, Craig LC, Allan K, Prabhu N, et al An exploratory study of the associations between maternal iron status in pregnancy and childhood wheeze and atopy. The British journal of nutrition. 2014;112(12):2018–27. Epub 2014/10/25. 10.1017/S0007114514003122 . 25342229

[32] Bener A, Ehlayel M, Hamid Q. The impact of anemia and hemoglobin level as a risk factor for asthma and allergic diseases. Indian Journal of Allergy, Asthma and Immunology. 2015;29(2):72–8. 10.4103/0972-6691.178271

[33] Lewis RA. Mastocytosis. J Allergy Clin Immunol. 1984;74(6):755–65. Epub 1984/12/01. 10.1016/0091-6749(84)90172-6 . 6389650

[34] Jawhar M, Schwaab J, Alvarez-Twose I, Shoumariyeh K, Naumann N, Lubke J, et al MARS: Mutation-Adjusted Risk Score for Advanced Systemic Mastocytosis. Journal of clinical oncology: official journal of the American Society of Clinical Oncology. 2019:Jco1900640 Epub 2019/09/12. 10.1200/jco.19.00640 . 31509472 PMC6823885

[35] Supuran CT. Carbonic anhydrases: novel therapeutic applications for inhibitors and activators. Nat Rev Drug Discov. 2008;7(2):168–81. Epub 2008/01/03. 10.1038/nrd2467 . 18167490

[36] Lavin Y, Winter D, Blecher-Gonen R, David E, Keren-Shaul H, Merad M, et al Tissue-resident macrophage enhancer landscapes are shaped by the local microenvironment. Cell. 2014;159(6):1312–26. Epub 2014/12/07. 10.1016/j.cell.2014.11.018 25480296 PMC4437213

[37] Xu J, Xu X, Wang B, Ma Y, Zhang L, Xu H, et al Nuclear carbonic anhydrase 6B associates with PRMT5 to epigenetically promote IL-12 expression in innate response. Proceedings of the National Academy of Sciences of the United States of America. 2017;114(32):8620–5. Epub 2017/07/26. 10.1073/pnas.1700917114 28739930 PMC5559001

[38] Urban JF Jr., Schopf L, Morris SC, Orekhova T, Madden KB, Betts CJ, et al Stat6 signaling promotes protective immunity against Trichinella spiralis through a mast cell- and T cell-dependent mechanism. J Immunol. 2000;164(4):2046–52. Epub 2000/02/05. 10.4049/jimmunol.164.4.2046 . 10657657

[39] Moreau R, Tshikudi Malu D, Dumais M, Dalko E, Gaudreault V, Roméro H, et al Alterations in bone and erythropoiesis in hemolytic anemia: comparative study in bled, phenylhydrazine-treated and Plasmodium-infected mice. PloS one. 2012;7(9):e46101–e. Epub 09/28. 10.1371/journal.pone.0046101 . 23029401 PMC3461039

[40] Ray A, Dittel BN. Isolation of mouse peritoneal cavity cells. Journal of visualized experiments: JoVE. 2010;(35):1488 10.3791/1488 . 20110936 PMC3152216

