# Supplementary figures and images for: Trichinella spiralis-induced mastocytosis and erythropoiesis are simultaneously supported by a bipotent mast cell/erythrocyte precursor cell
Source: PLoS Pathog. 2020 May 18;16(5):e1008579. doi: 10.1371/journal.ppat.1008579 (PMC7259795; doi:10.1371/journal.ppat.1008579)

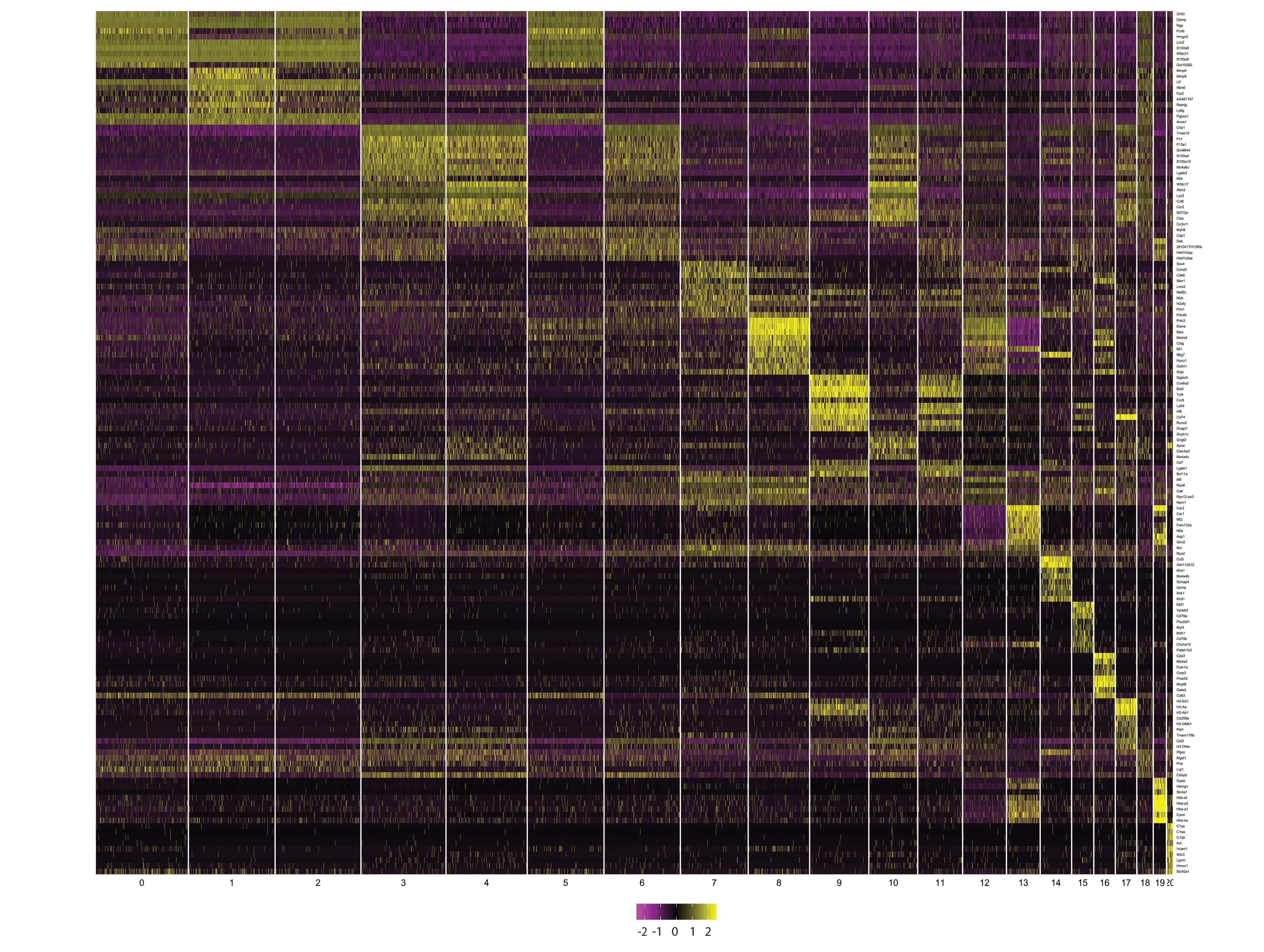

Supplement: S1 Fig — Heat map illustrating the top marker genes defining the 20 distinct clusters identified by single cell RNA-seq analysis of bone marrow resident cells. (TIF) [file ppat.1008579.s001.tif]

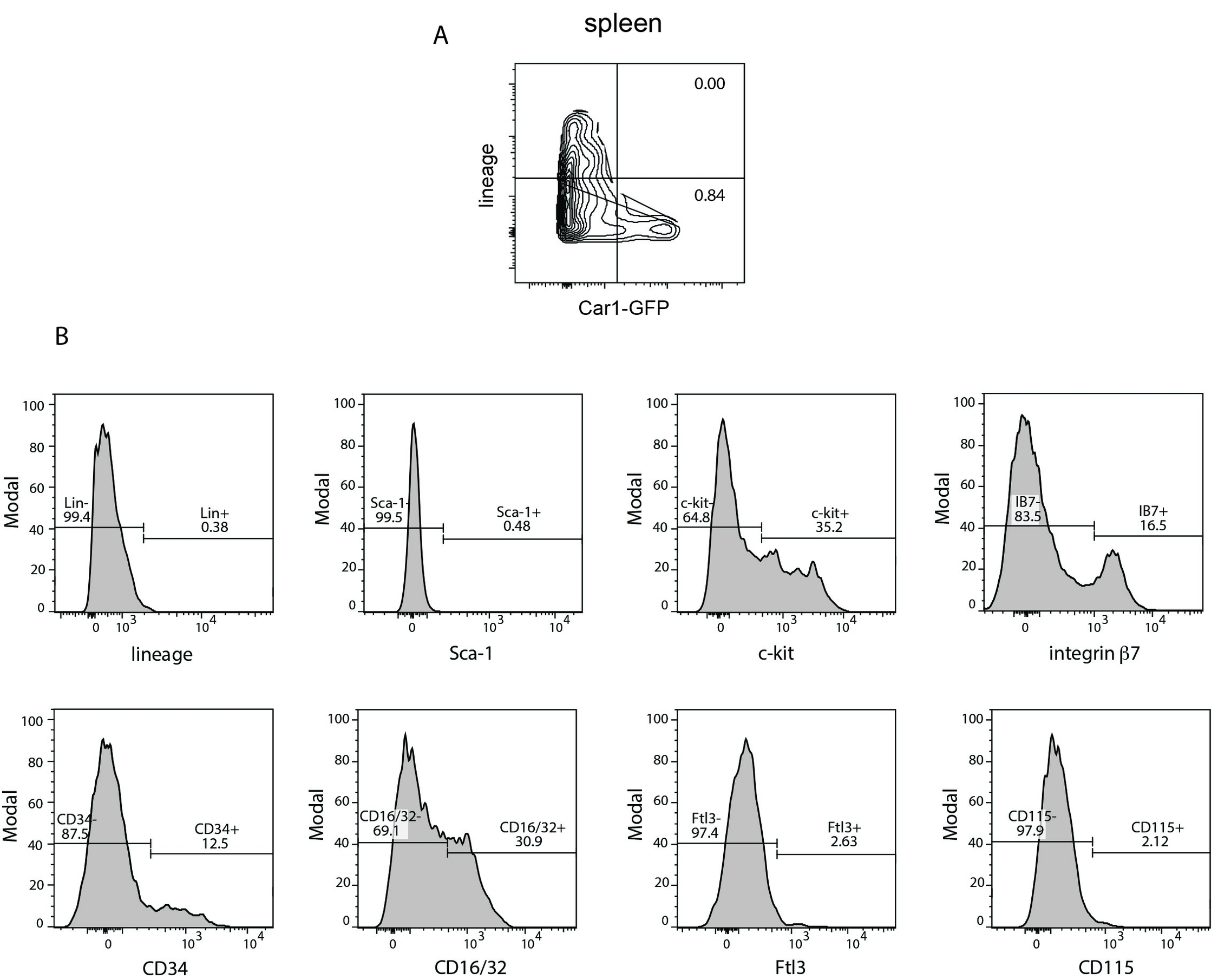

Supplement: S2 Fig — (A), Car1-GFP expression within lineage- and lineage+ compartments in the spleen was determined by flow cytometric analysis. (B), Expression levels of progenitor-associated markers on Car1-GFP+ cells in the bone marrow. Results are representative of 3 separate experiments. (TIF) [file ppat.1008579.s002.tif]

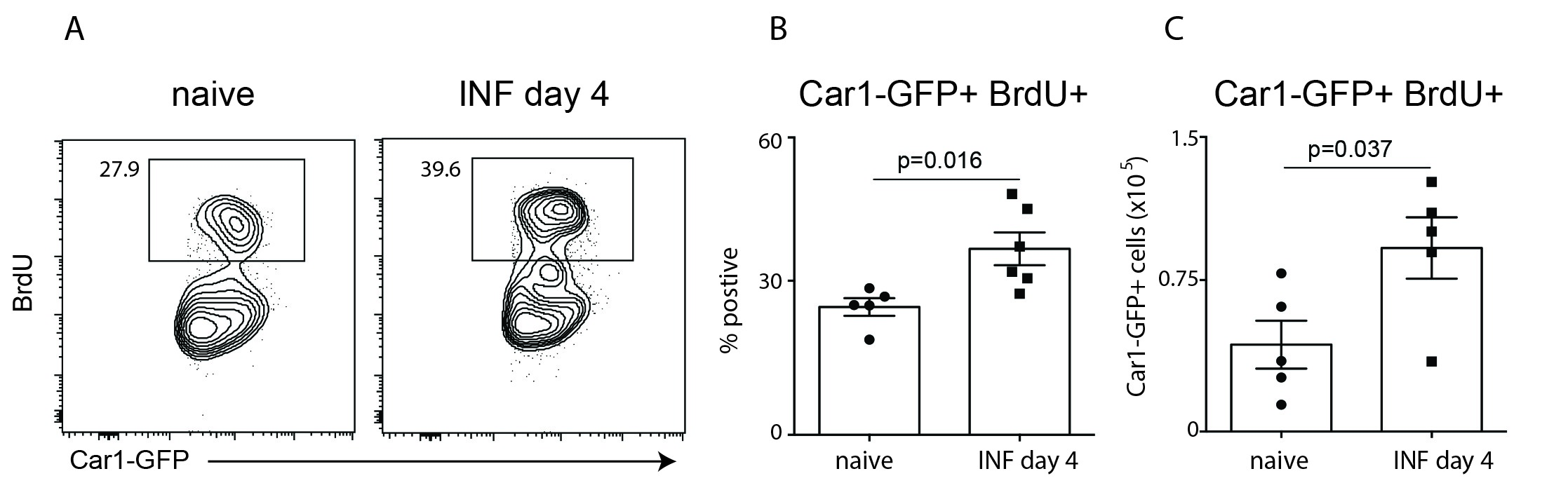

Supplement: S3 Fig — (A), Car1-GFP reporter mice were infected with T. spiralis and treated with BrdU and the (B,C) percentage and total Car1-GFP+ cells in the spleen were determined on day 4 post infection. Results are representative of 2 separate experiments comprised of 10 biological replicates total. Statistical analysis performed using a Student’s t-test. (TIF) [file ppat.1008579.s003.tif]

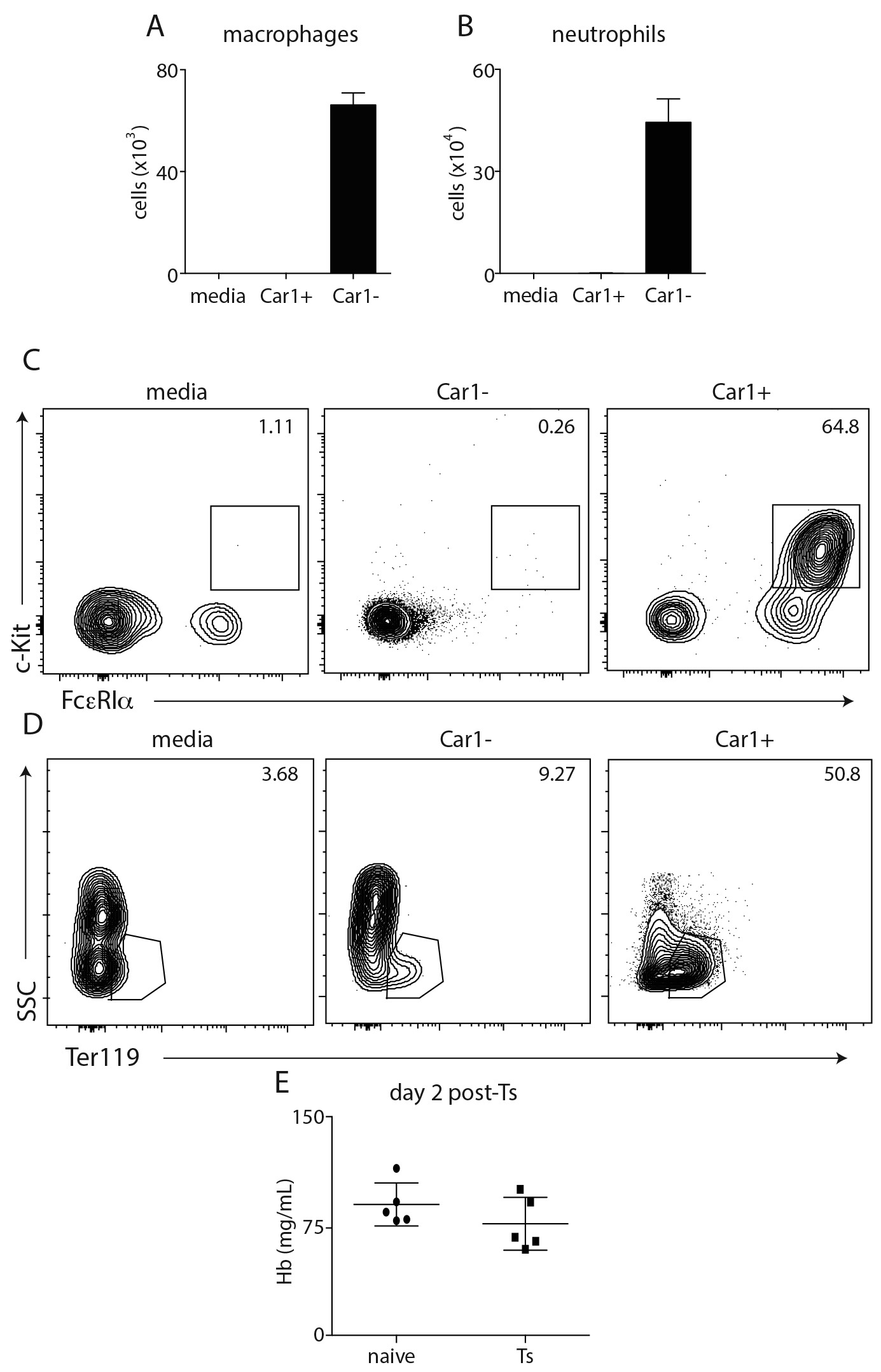

Supplement: S4 Fig — Car1-GFP+ or Car1-GFP- cells were sort-purified from the bone marrow of mice and seeded into MethoCult and the numbers of (A) macrophages and (B) neutrophils were evaluated by flow cytometric analysis post-culture. (C,D), Car1+ or Car1- cells were sort-purified from the spleens of mice and seeded into MethoCult with hematopoietic cytokines. Representative plots illustrating the percentage of mast cells (MCs) and erythrocytes identified by flow cytometric analysis post-culture. (E), Hemoglobin (Hb) levels were quantified on day 2 post-Trichinella infection. Results are representative of at least 3 separate experiments. (TIF) [file ppat.1008579.s004.tif]

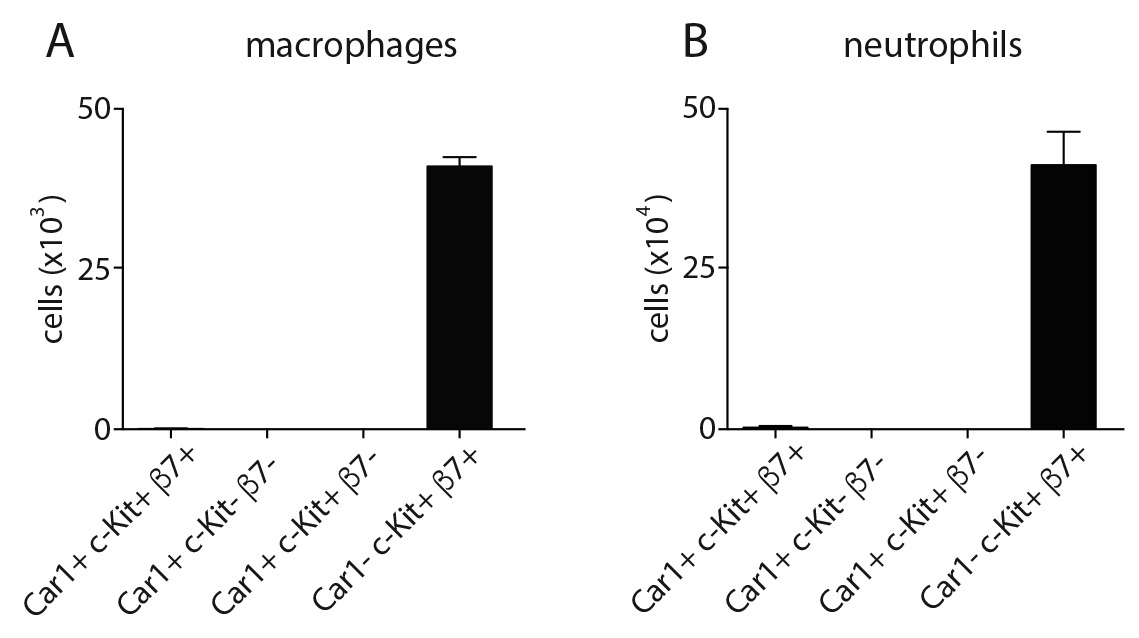

Supplement: S5 Fig — Car1-GFP+ c-Kit+ β7+, Car1-GFP+ c-Kit- β7-, Car1-GFP+ c-Kit+ β7-, or Car1-GFP- c-Kit+ β7+ cells were sort-purified from the bone marrow of mice and seeded into MethoCult and the total numbers of (A) macrophages and (B) neutrophils were evaluated by flow cytometric analysis post-culture. Results are representative of at least 3 separate experiments. (TIF) [file ppat.1008579.s005.tif]

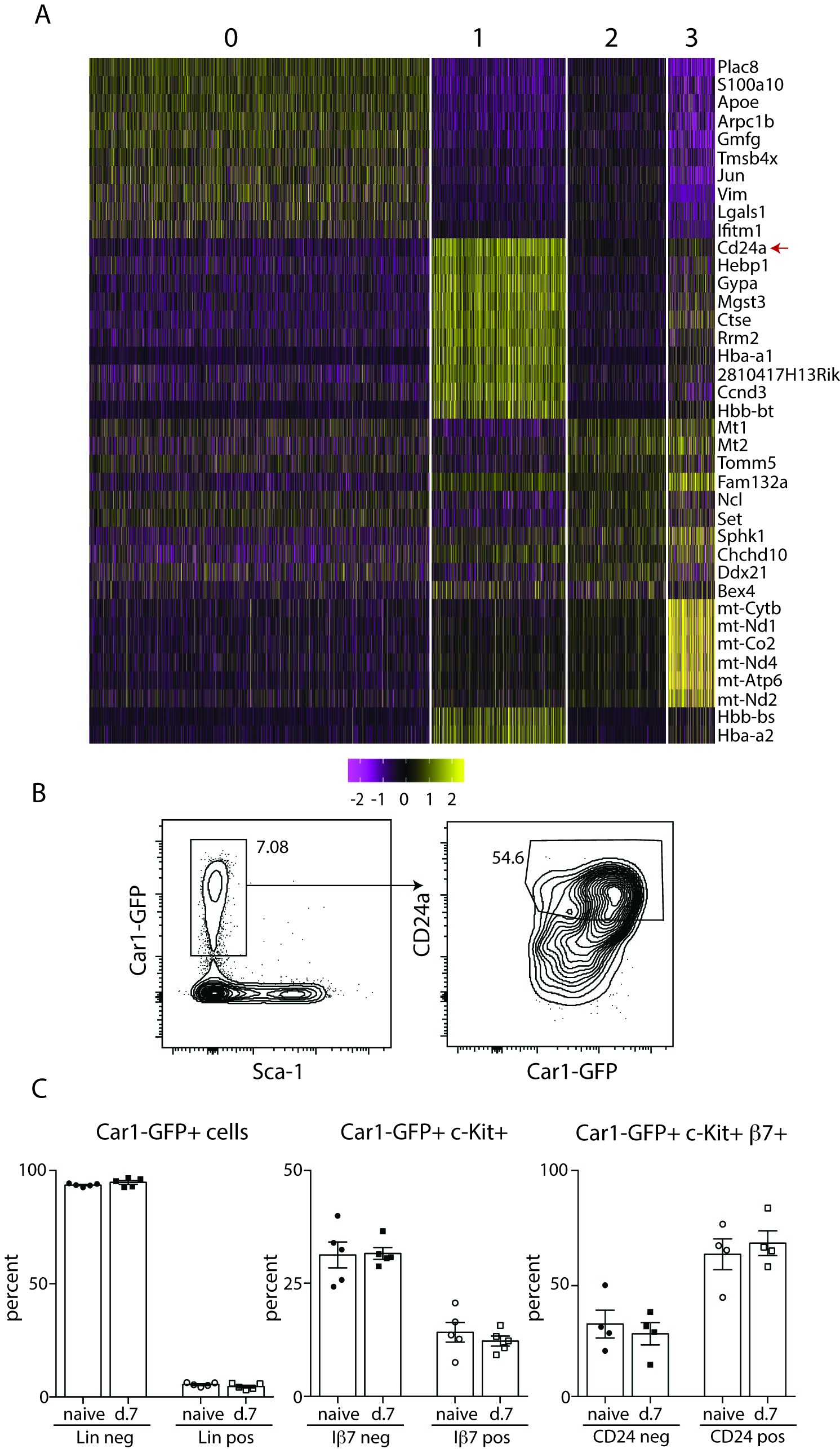

Supplement: S6 Fig — (A), Heat map illustrating the top marker genes defining the 4 distinct clusters identified by single cell RNA-seq analysis of bone marrow resident GFP+ cells. (B), Bone marrow resident Car1-GFP+ cells were evaluated for CD24a expression. (C), Expression patterns of lineage markers, c-Kit, integrin β7 and CD24a were evaluated on bone marrow-resident Car1-GFP+ cells 7 days post-T. spiralis infection. (TIF) [file ppat.1008579.s006.tif]
