# Supplementary material for: Trichinella spiralis-induced mastocytosis and erythropoiesis are simultaneously supported by a bipotent mast cell/erythrocyte precursor cell
Source: PLoS Pathog. 2020 May 18;16(5):e1008579. doi: 10.1371/journal.ppat.1008579 (PMC7259795; doi:10.1371/journal.ppat.1008579)
Supplement: S1 Table — (PDF) [file ppat.1008579.s007.pdf]

p value	Av_log FC	pct.1	pct.2	p_val_adj	cluster	gene symbol	Predicted cell type
2.16E-222	1.211418752	0.996	0.51	2.85E-218	0	Chil3	Neutrophil
1.94E-213	1.267768556	1	0.924	2.56E-209	0	Camp	
5.58E-195	1.222154003	1	0.835	7.36E-191	0	Ngp	
9.51E-186	1.176517108	0.687	0.162	1.25E-181	0	Fcnb	
2.16E-183	1.065804766	0.966	0.551	2.85E-179	0	Hmgn2	
3.11E-172	0.99968864	1	0.516	4.10E-168	0	Lcn2	
1.78E-165	0.940500031	0.961	0.398	2.35E-161	0	Pglyrp1	
8.51E-165	1.099093172	1	0.999	1.12E-160	0	S100a8	
1.68E-163	0.97260825	1	0.595	2.22E-159	0	Wfdc21	
3.48E-161	0.927008053	0.991	0.498	4.59E-157	0	Ltf	
6.57E-161	1.099898095	1	0.994	8.66E-157	0	S100a9	
4.16E-134	-0.901990192	0.948	0.957	5.49E-130	0	Rps27	
3.22E-131	0.965445249	0.679	0.218	4.25E-127	0	Gm10282	
5.40E-128	-1.301794883	0.748	0.869	7.12E-124	0	Rpl35	
6.54E-126	-1.103082612	0.863	0.905	8.63E-122	0	Rps28	
8.70E-126	-0.97838138	0.966	0.955	1.15E-121	0	Rpl41	
1.69E-122	-1.09546059	0.806	0.881	2.23E-118	0	Rpl18a	
2.44E-120	-1.190330703	0.762	0.86	3.22E-116	0	Rpl13	
1.71E-119	0.833005161	0.62	0.175	2.25E-115	0	Cd177	
4.23E-119	-1.02797304	0.819	0.887	5.58E-115	0	Rps14	
8.15E-116	-0.983918779	0.851	0.907	1.08E-111	0	Rpl37	
8.31E-112	0.888600704	0.542	0.147	1.10E-107	0	Cebpe	
8.21E-109	-1.148041284	0.757	0.843	1.08E-104	0	Rps18	
1.76E-108	-0.845327973	0.924	0.94	2.33E-104	0	Rpl37a	
4.24E-107	-0.710538715	0.996	0.974	5.59E-103	0	Rps29	
5.50E-107	-1.215206774	0.636	0.799	7.25E-103	0	Rps5	
1.34E-102	-0.945762127	0.877	0.898	1.77E-98	0	Rps19	
3.73E-99	-1.068957037	0.693	0.82	4.92E-95	0	Rpl13a	
7.19E-99	-1.094885079	0.67	0.806	9.48E-95	0	Rps11	

1.23E-98	-1.325127207	0.425	0.722	1.62E-94	0	Rpl10
6.21E-97	-1.209288782	0.625	0.779	8.19E-93	0	Rpl36
1.03E-95	0.619507796	0.97	0.761	1.36E-91	0	Hmgb2
1.16E-93	0.655875943	0.847	0.389	1.53E-89	0	Anxa1
5.78E-93	-0.95195122	0.831	0.871	7.63E-89	0	Rpl32
7.31E-92	-1.246702651	0.54	0.74	9.64E-88	0	Rplp1
1.96E-90	-1.22623236	0.504	0.731	2.59E-86	0	Rplp0
2.20E-90	-1.961981353	0.563	0.757	2.90E-86	0	Tmsb10
2.54E-90	-0.907470428	0.776	0.851	3.34E-86	0	Rps27rt
1.48E-89	-1.01346154	0.655	0.793	1.95E-85	0	Rpl26
1.64E-87	-0.991071357	0.714	0.82	2.16E-83	0	Rps23
3.55E-85	-1.121624842	0.552	0.742	4.68E-81	0	Rps4x
1.70E-84	-0.870333654	0.801	0.854	2.24E-80	0	Rpl38
5.67E-84	-0.963599543	0.794	0.84	7.47E-80	0	Rps8
1.54E-82	-0.987807108	0.67	0.782	2.03E-78	0	Rps3a1
3.58E-82	0.691401102	0.504	0.155	4.72E-78	0	Adpgk
1.63E-80	-0.743177549	0.849	0.875	2.15E-76	0	Rps9
1.99E-79	-1.054231293	0.568	0.736	2.62E-75	0	Rpl27a
9.95E-79	-0.990027203	0.673	0.786	1.31E-74	0	Rpl39
1.65E-77	-1.178774749	0.481	0.694	2.18E-73	0	Gm2000
1.08E-76	-1.001208488	0.623	0.759	1.42E-72	0	Rps6
2.17E-76	-1.920514062	0.384	0.661	2.86E-72	0	Crip1
1.18E-75	-0.850199517	0.758	0.812	1.56E-71	0	Rps16
4.77E-74	-0.824817997	0.755	0.825	6.30E-70	0	Rpl23a
9.34E-73	-1.12325883	0.453	0.679	1.23E-68	0	Eef1a1
1.02E-72	-0.69400177	0.858	0.879	1.34E-68	0	Rps27a
1.30E-72	-0.953467526	0.634	0.765	1.71E-68	0	Gm10076
5.22E-72	-1.018862023	0.602	0.736	6.89E-68	0	Rps26
2.02E-71	-0.802476715	0.739	0.811	2.66E-67	0	Rpl34
3.14E-71	0.547485748	0.936	0.639	4.14E-67	0	Arhgdib
6.04E-71	-1.115892777	0.517	0.702	7.97E-67	0	Rpl14

5.16E-70	0.650169227	0.382	0.107	6.81E-66	0	Syne1
1.84E-69	-0.878115511	0.655	0.773	2.43E-65	0	Rpl17
3.52E-69	-1.798575028	0.156	0.508	4.64E-65	0	Lgals1
6.60E-69	-0.932746596	0.574	0.736	8.71E-65	0	Rpl19
5.96E-68	-1.228534291	0.51	0.701	7.87E-64	0	Plac8
5.95E-67	0.625263022	0.318	0.08	7.85E-63	0	Fam101b
7.89E-67	-0.848417463	0.723	0.783	1.04E-62	0	Rplp2
1.03E-65	0.616367847	0.428	0.133	1.36E-61	0	Cd9
2.00E-65	-1.181035922	0.368	0.622	2.64E-61	0	Rpl3
3.01E-65	-0.946520612	0.6	0.733	3.97E-61	0	Rpl6
9.19E-65	-0.869314282	0.659	0.753	1.21E-60	0	Rpl24
1.60E-64	0.647898756	0.291	0.072	2.11E-60	0	Gca
2.01E-64	-0.816453153	0.686	0.776	2.66E-60	0	Rpl21
1.25E-63	-1.042352898	0.472	0.663	1.65E-59	0	Gm10263
1.52E-62	-0.952784771	0.549	0.705	2.00E-58	0	Rps7
1.60E-62	-1.444792174	0.156	0.499	2.11E-58	0	Ly6e
3.19E-62	0.63033009	0.364	0.108	4.21E-58	0	Trem3
1.13E-61	-0.83223059	0.663	0.757	1.49E-57	0	Rps15a
1.43E-61	0.584639303	0.741	0.363	1.88E-57	0	Dstn
5.84E-61	-0.731414096	0.753	0.81	7.71E-57	0	Rps24
1.75E-60	0.733294825	0.396	0.132	2.30E-56	0	Ube2c
1.85E-60	-0.815971599	0.801	0.823	2.44E-56	0	Ftl1
5.71E-59	-0.602540312	0.872	0.872	7.53E-55	0	Rpl35a
4.61E-57	0.509803516	0.302	0.081	6.08E-53	0	Lrg1
2.62E-56	-0.761616437	0.698	0.767	3.46E-52	0	Rps3
2.98E-56	-1.267933365	0.183	0.494	3.93E-52	0	Rpl10-ps3
1.83E-55	-1.019186962	0.474	0.66	2.42E-51	0	Rpl36a
2.77E-54	0.44313168	0.718	0.353	3.65E-50	0	Hist1h2ap
3.22E-54	0.51264858	0.787	0.423	4.25E-50	0	Hp
3.94E-54	-1.069616699	0.389	0.597	5.19E-50	0	Rpl15
6.45E-54	-0.505093945	0.929	0.918	8.51E-50	0	Fau

1.68E-53	-0.841754562	0.602	0.716	2.21E-49	0	Sub1
1.00E-52	0.654046063	0.306	0.094	1.32E-48	0	Spc25
1.20E-52	0.557581595	0.803	0.447	1.59E-48	0	F630028O10Rik
2.34E-52	-0.948638476	0.547	0.67	3.09E-48	0	Rps12
3.22E-52	-0.473310325	0.961	0.954	4.25E-48	0	mt-Co3
3.75E-52	-0.820562915	0.567	0.691	4.95E-48	0	Rpl27
3.86E-52	-0.850357981	0.519	0.685	5.09E-48	0	Rpl23a-ps3
9.10E-52	-0.898374459	0.52	0.66	1.20E-47	0	Rpl8
1.18E-51	0.588118515	0.497	0.203	1.56E-47	0	Cpne3
1.55E-51	0.582973453	0.472	0.187	2.04E-47	0	Serpinb1a
2.48E-51	-1.218250706	0.215	0.494	3.27E-47	0	Gm8730
2.84E-51	-0.624401335	0.854	0.86	3.74E-47	0	mt-Atp6
5.30E-51	0.503912234	0.382	0.128	6.99E-47	0	Id2
5.48E-51	-0.772154156	0.703	0.745	7.23E-47	0	Rpl23
1.31E-50	-0.826556707	0.623	0.717	1.72E-46	0	Rpl31
2.26E-50	-0.802774374	0.654	0.723	2.98E-46	0	Rpl11
3.73E-50	-1.220009145	0.178	0.465	4.92E-46	0	Gm10073
7.41E-50	-1.025052598	0.396	0.593	9.77E-46	0	Rpl10a
1.11E-49	-0.880563085	0.508	0.657	1.47E-45	0	Rpl18
3.67E-48	-0.698836559	0.698	0.75	4.84E-44	0	Rpl9
4.04E-48	-0.790936216	0.586	0.702	5.33E-44	0	Rps13
3.14E-47	0.5752311	0.362	0.129	4.14E-43	0	Lta4h
1.41E-46	-0.676804107	0.689	0.75	1.86E-42	0	Rps15
4.23E-46	-1.087209062	0.384	0.571	5.58E-42	0	Rps2
6.61E-46	0.620614707	0.373	0.141	8.72E-42	0	Nusap1
1.01E-45	-0.912100787	0.437	0.605	1.33E-41	0	Wdr89
1.46E-45	0.434967079	0.488	0.198	1.92E-41	0	Cd63
1.95E-45	-0.73759513	0.666	0.714	2.57E-41	0	Rpl28
1.11E-44	-0.694587953	0.67	0.733	1.47E-40	0	Rps25
3.69E-44	-1.074701808	0.242	0.492	4.86E-40	0	Rps18-ps3
4.07E-44	-0.898188132	0.437	0.605	5.37E-40	0	Rpl4

1.39E-43	-0.704281034	0.602	0.717	1.84E-39	0	Tpt1
8.12E-43	0.536811188	0.456	0.19	1.07E-38	0	C3
2.37E-42	-1.149157427	0.185	0.451	3.13E-38	0	Prdx1
2.46E-42	-1.104719991	0.286	0.503	3.25E-38	0	Rpl12
2.97E-42	-0.960563383	0.341	0.544	3.92E-38	0	Rpl22
3.26E-42	0.439081254	0.625	0.306	4.31E-38	0	2810417H13Rik
3.65E-42	0.526723215	0.291	0.095	4.82E-38	0	Mgst2
3.77E-42	0.545295491	0.655	0.358	4.97E-38	0	1810037I17Rik
5.32E-42	-0.745769685	0.613	0.69	7.02E-38	0	Rpsa
7.76E-42	0.512857072	0.405	0.16	1.02E-37	0	Ckap4
7.82E-42	-1.229864131	0.368	0.56	1.03E-37	0	Psap
3.10E-41	-0.706717394	0.679	0.725	4.09E-37	0	Rps17
5.22E-41	-0.955816697	0.519	0.654	6.89E-37	0	Fth1
5.94E-40	-0.686107431	0.705	0.777	7.83E-36	0	B2m
7.82E-40	-1.110490784	0.174	0.425	1.03E-35	0	Rpl36-ps3
1.06E-39	-0.830588903	0.483	0.633	1.40E-35	0	mt-Nd1
1.25E-39	0.479273913	0.252	0.077	1.65E-35	0	Actn1
1.56E-39	-1.43406073	0.083	0.34	2.06E-35	0	Ms4a6c
1.81E-39	0.345783739	0.925	0.661	2.39E-35	0	Cyba
4.77E-39	-0.726099024	0.581	0.665	6.29E-35	0	Rps10
8.92E-39	-0.671533649	0.616	0.712	1.18E-34	0	mt-Cytb
1.16E-38	0.397973795	0.519	0.233	1.53E-34	0	Lmo4
2.33E-38	-1.230520945	0.352	0.549	3.07E-34	0	S100a6
8.01E-38	0.364769006	0.886	0.622	1.06E-33	0	Prdx5
1.80E-37	0.388631536	0.522	0.239	2.38E-33	0	Ncf1
4.43E-37	0.460669147	0.295	0.103	5.85E-33	0	Arrb2
2.36E-36	-0.774920934	0.506	0.618	3.11E-32	0	Rps21
1.22E-35	0.419280328	0.52	0.241	1.61E-31	0	Clec4a2
1.41E-35	-0.983679409	0.234	0.454	1.86E-31	0	Rpl13-ps3
1.45E-35	-1.407687739	0.057	0.295	1.92E-31	0	Ccr2
2.73E-35	-0.538689802	1	0.997	3.60E-31	0	Malat1

3.57E-35	-0.70752639	0.529	0.641	4.71E-31	0	Uba52
5.01E-35	-0.699024976	0.6	0.668	6.60E-31	0	Rps20
1.01E-34	0.431230574	0.583	0.299	1.33E-30	0	Scp2
1.40E-34	-1.023062558	0.222	0.438	1.84E-30	0	Hsp90ab1
1.57E-34	-0.688962212	0.528	0.648	2.08E-30	0	Sec61g
1.38E-33	0.464178338	0.268	0.097	1.82E-29	0	Cenpf
1.46E-33	0.414197073	0.691	0.398	1.93E-29	0	Tkt
3.63E-33	0.345915531	0.794	0.473	4.79E-29	0	Cybb
7.99E-33	-0.698884498	0.517	0.62	1.05E-28	0	Gm11808
2.90E-32	-0.898052344	0.24	0.453	3.82E-28	0	Gm9493
4.63E-32	-0.854663116	0.282	0.482	6.11E-28	0	Rpl30
5.13E-32	0.380474868	0.565	0.295	6.76E-28	0	Top2a
6.32E-32	0.47553769	0.437	0.209	8.33E-28	0	Smc2
1.05E-31	-1.203097274	0.062	0.283	1.39E-27	0	S100a10
3.76E-31	-1.012248158	0.19	0.401	4.96E-27	0	Npm1
5.56E-31	-1.555390152	0.167	0.377	7.34E-27	0	Ifi27l2a
5.57E-31	-0.909972368	0.295	0.473	7.34E-27	0	Rps12-ps3
7.60E-31	-1.233875268	0.098	0.313	1.00E-26	0	Pdcd4
7.65E-31	0.439486966	0.382	0.174	1.01E-26	0	Rrm2
2.54E-30	-0.482393416	0.78	0.792	3.35E-26	0	Gm9843
5.21E-30	0.297395381	0.975	0.805	6.88E-26	0	H2afz
1.76E-29	-1.181799918	0.066	0.276	2.32E-25	0	Gm9844
4.52E-29	-0.840318883	0.329	0.489	5.97E-25	0	Rps26-ps1
4.85E-29	-0.822573513	0.314	0.485	6.40E-25	0	Rpl29
5.12E-29	-0.311704929	0.988	0.978	6.76E-25	0	mt-Co1
5.37E-29	-0.904716355	0.561	0.639	7.08E-25	0	Cst3
2.92E-28	-1.119103066	0.052	0.254	3.85E-24	0	Zeb2
3.37E-28	-0.830423396	0.256	0.448	4.44E-24	0	Rpl5
1.80E-27	0.362258808	0.487	0.248	2.37E-23	0	Gpi1
2.03E-27	0.452877066	0.403	0.199	2.68E-23	0	Mki67
3.64E-27	-0.694628207	0.423	0.559	4.80E-23	0	Rpl7

4.43E-27	0.335029673	0.261	0.1	5.85E-23	0	Ets1
7.36E-27	-0.784242703	0.332	0.488	9.71E-23	0	Rpl27-ps3
1.42E-26	0.426299005	0.256	0.1	1.88E-22	0	Pygl
1.67E-26	-0.810168638	0.277	0.454	2.21E-22	0	Gnb2l1
1.84E-26	0.363215024	0.606	0.343	2.43E-22	0	Gsr
3.31E-26	-0.749969132	0.462	0.588	4.36E-22	0	H2-D1
4.65E-26	-0.680597112	0.487	0.582	6.13E-22	0	Rpl36al
5.75E-26	0.37889876	0.327	0.145	7.59E-22	0	Ncf4
8.49E-26	0.469154361	0.364	0.178	1.12E-21	0	Tuba4a
8.88E-26	0.426795684	0.382	0.189	1.17E-21	0	Birc5
4.49E-25	-0.67081074	0.472	0.58	5.92E-21	0	mt-Nd4
1.14E-24	-0.919948109	0.11	0.301	1.51E-20	0	Eef1g
1.51E-24	-0.845267698	0.179	0.367	1.99E-20	0	Rpl6l
2.17E-24	-0.954684513	0.149	0.339	2.86E-20	0	Npc2
2.23E-24	-0.761945272	0.268	0.442	2.94E-20	0	Eef2
3.93E-24	-0.834930473	0.306	0.462	5.19E-20	0	Psma7
4.26E-23	0.312643718	0.48	0.255	5.62E-19	0	Aldh2
1.43E-22	-0.83946905	0.178	0.352	1.88E-18	0	Nop10
4.75E-22	-0.909410686	0.192	0.36	6.27E-18	0	H2afy
5.33E-22	0.335015986	0.694	0.448	7.03E-18	0	Txn1
4.01E-21	0.309914979	0.694	0.442	5.28E-17	0	Rac2
5.31E-21	-0.69623613	0.414	0.514	7.00E-17	0	Rpl22l1
6.39E-21	-0.785803587	0.147	0.321	8.43E-17	0	Polr2l
2.55E-20	0.332998227	0.311	0.151	3.37E-16	0	Tecr
7.18E-20	-0.782100243	0.131	0.298	9.47E-16	0	Gm10269
8.48E-20	-0.717588802	0.227	0.39	1.12E-15	0	Btf3
1.34E-19	-0.658202076	0.377	0.487	1.77E-15	0	Rps27l
2.98E-19	0.28794638	0.261	0.118	3.93E-15	0	Vasp
3.87E-19	0.310653716	0.291	0.139	5.11E-15	0	Glrx
1.51E-18	-0.732195291	0.171	0.332	1.99E-14	0	Eef1b2
1.84E-18	0.336010012	0.423	0.243	2.43E-14	0	Aprt

3.12E-18	-0.774069862	0.16	0.318	4.12E-14	0	mt-Nd2
6.36E-18	-0.859776903	0.107	0.257	8.39E-14	0	Calr
1.64E-17	-0.596884146	0.405	0.509	2.16E-13	0	Rpl7a
1.91E-17	0.384635918	0.334	0.184	2.52E-13	0	Smc4
2.41E-17	0.281812332	0.526	0.314	3.18E-13	0	Clec12a
2.45E-17	-0.621221903	0.336	0.459	3.23E-13	0	Rpl9-ps6
4.48E-17	-1.008458263	0.51	0.563	5.90E-13	0	Ifitm3
5.41E-17	0.288663373	0.465	0.276	7.14E-13	0	Ppp1cb
1.00E-16	0.261792233	0.272	0.131	1.33E-12	0	Gpsm3
1.15E-16	0.25830262	0.332	0.173	1.52E-12	0	Hcst
1.37E-16	-0.751086704	0.215	0.354	1.80E-12	0	Set
1.71E-16	0.27049247	0.508	0.309	2.25E-12	0	Cks2
1.72E-16	-0.562896369	0.485	0.551	2.27E-12	0	Hspa8
2.08E-16	0.289639906	0.321	0.172	2.74E-12	0	Lockd
2.80E-16	-0.812600789	0.126	0.267	3.69E-12	0	Ncl
3.11E-16	-0.751539565	0.119	0.264	4.10E-12	0	Psmb8
3.31E-16	0.304486283	0.359	0.2	4.36E-12	0	Rad21
4.63E-16	-0.748024841	0.211	0.347	6.11E-12	0	Hnrnpa1
5.77E-16	-0.584746968	0.44	0.514	7.61E-12	0	Sec61b
6.20E-16	0.296370163	0.302	0.159	8.18E-12	0	Tmed3
7.32E-16	-0.624305535	0.337	0.443	9.65E-12	0	Ybx1
7.93E-16	-0.827316843	0.121	0.257	1.05E-11	0	Fxyd5
8.96E-16	-0.696082922	0.199	0.333	1.18E-11	0	Gm10036
9.09E-16	0.335042625	0.288	0.151	1.20E-11	0	H2afx
1.03E-15	-0.76327963	0.233	0.37	1.36E-11	0	H2-K1
2.99E-15	0.264772921	0.32	0.171	3.94E-11	0	Ogfrl1
3.59E-15	-0.612738835	0.417	0.512	4.74E-11	0	Mbnl1
3.94E-15	-0.413795837	0.631	0.651	5.20E-11	0	Atp5l
5.03E-15	-0.778260023	0.199	0.328	6.64E-11	0	Lsp1
1.14E-14	-0.479868031	0.867	0.81	1.50E-10	0	Tyrobp
1.21E-14	-0.677202626	0.194	0.331	1.60E-10	0	Gm10116

2.04E-14	-0.58133009	0.366	0.459	2.68E-10	0	Naca
2.65E-14	-0.402898136	0.643	0.668	3.50E-10	0	Cox4i1
2.86E-14	0.268030963	0.552	0.362	3.78E-10	0	Lbr
2.91E-14	-0.544813849	0.391	0.491	3.83E-10	0	Uqcrh
4.16E-14	-0.665269011	0.215	0.346	5.49E-10	0	Pomp
9.90E-14	-0.630968937	0.206	0.337	1.31E-09	0	Eif3f
1.13E-13	-0.611989288	0.249	0.367	1.50E-09	0	Snrpf
2.40E-13	-0.635906565	0.204	0.325	3.17E-09	0	Atp5g1
2.43E-13	-0.377945581	0.627	0.657	3.21E-09	0	mt-Co2
1.03E-12	0.263689482	0.282	0.155	1.36E-08	0	Cited2
1.12E-12	0.278298929	0.371	0.228	1.48E-08	0	Rnaseh2c
1.79E-12	-0.74825426	0.373	0.445	2.36E-08	0	Lgals3
2.04E-12	-0.641842382	0.172	0.295	2.69E-08	0	Nsa2
2.54E-12	-0.605447934	0.217	0.334	3.35E-08	0	Slc25a5
2.55E-12	-0.610977998	0.234	0.351	3.36E-08	0	Hsp90b1
2.57E-12	-1.166907732	0.167	0.28	3.39E-08	0	Mpo
3.18E-12	-0.493806101	0.648	0.636	4.19E-08	0	Xist
3.64E-11	-0.551648671	0.368	0.433	4.80E-07	0	Serbp1
4.34E-11	-0.582483692	0.325	0.411	5.72E-07	0	Dbi
5.90E-11	-0.555865922	0.316	0.404	7.78E-07	0	mt-Atp8
9.36E-11	-0.473353047	0.503	0.539	1.23E-06	0	Mrpl33
1.95E-10	-0.366029742	0.597	0.603	2.57E-06	0	Atp5e
4.46E-10	-0.341333685	0.947	0.832	5.88E-06	0	Ptma
4.65E-10	-0.315459892	0.728	0.694	6.13E-06	0	Oaz1
1.20E-09	-0.564841002	0.194	0.293	1.58E-05	0	Cmtm7
2.72E-09	-0.429171922	0.417	0.484	3.59E-05	0	Morf4l1
4.61E-09	-0.541538322	0.295	0.38	6.09E-05	0	Srrm2
6.16E-09	-0.522572848	0.218	0.31	8.13E-05	0	Erh
8.22E-09	-0.512365976	0.245	0.335	0.000108429	0	Ndufa7
1.30E-08	-0.624752002	0.258	0.34	0.000171466	0	Notch2
2.30E-08	-0.530039896	0.208	0.299	0.000303211	0	Rsrp1

3.10E-08	-0.476810147	0.274	0.356	0.000408597	0	Cox7b
3.14E-08	-0.493411683	0.169	0.262	0.000414501	0	Eif3a
6.20E-08	-0.484208014	0.252	0.332	0.000818467	0	Ranbp1
8.80E-08	-0.431707012	0.423	0.454	0.001160965	0	Uqcr11
1.43E-07	-0.45706238	0.229	0.315	0.00188504	0	Canx
1.49E-07	-0.516508589	0.185	0.271	0.001961172	0	Prrc2c
1.59E-07	-0.547165258	0.185	0.265	0.002096297	0	Ucp2
1.60E-07	-0.562364677	0.226	0.3	0.002109829	0	Laptm5
2.48E-07	-0.385720776	0.526	0.519	0.003277106	0	Snrpg
3.25E-07	-0.466440757	0.242	0.321	0.004287562	0	Eif3k
3.51E-07	-0.451077992	0.384	0.436	0.004633336	0	Serinc3
3.54E-07	-0.909344778	0.21	0.279	0.004666497	0	Prtn3
7.51E-07	-0.457708116	0.206	0.284	0.009907016	0	Bola2
7.59E-07	-0.444895012	0.362	0.416	0.01001222	0	Atox1
1.10E-06	-0.452347787	0.229	0.305	0.014554505	0	Atp5g2
1.17E-06	-0.478749219	0.206	0.283	0.015442735	0	Arglu1
2.33E-06	-0.473143534	0.297	0.353	0.030727695	0	Ptpn18
2.62E-06	-0.440661407	0.21	0.285	0.034614381	0	mt-Nd4l
3.36E-06	-0.441505947	0.222	0.292	0.04433874	0	Eif4a1
6.06E-06	-0.393474743	0.341	0.39	0.079978938	0	Tmem258
6.23E-06	-0.404895759	0.185	0.259	0.082118702	0	Timm13
7.72E-06	-0.389912861	0.242	0.311	0.101855003	0	Hnrnpm
7.89E-06	-0.435323082	0.698	0.606	0.104037056	0	Ly6c2
8.42E-06	-0.460887113	0.284	0.346	0.111021442	0	Ifitm2
2.56E-05	-0.351918762	0.387	0.415	0.337906194	0	2010107E04Rik
2.57E-05	-0.510314839	0.201	0.26	0.338422151	0	Btg1
2.88E-05	-0.385078614	0.313	0.367	0.380536121	0	BC005537
3.22E-05	-0.379056545	0.366	0.394	0.425020977	0	Gm8186
3.66E-05	-1.136902331	0.199	0.252	0.482164497	0	Elane
6.13E-05	-0.444411842	0.325	0.367	0.808944344	0	Serp1
7.34E-05	-0.392442562	0.194	0.254	0.967562085	0	Rbm25

8.20E-05	-0.397590004	0.25	0.305	1	0	Ost4
8.90E-05	-0.320893277	0.462	0.476	1	0	H2afj
8.96E-05	-0.335477812	0.371	0.408	1	0	Ndufa13
9.10E-05	-0.356352788	0.377	0.409	1	0	Uqcrq
0.00010146	-0.36816904	0.265	0.317	1	0	Cycs
0.00014146	-0.309403633	0.444	0.452	1	0	Usmg5
0.00015996	-0.364248887	0.236	0.292	1	0	Clta
0.00019091	-0.307345061	0.38	0.411	1	0	Rbm39
0.00024535	-0.349795748	0.419	0.427	1	0	Hint1
0.00033116	-0.449945475	0.217	0.26	1	0	Anxa2
0.00057609	-0.262073217	0.627	0.568	1	0	Cox8a
0.00065431	-0.394727682	0.249	0.291	1	0	Psmb1
0.00069087	-0.35204683	0.282	0.322	1	0	Nedd8
0.00071197	-0.340599366	0.336	0.367	1	0	Son
0.00088077	-0.266070929	0.202	0.256	1	0	Ndufb2
0.00093308	-0.325611904	0.217	0.268	1	0	Gnas
0.00098654	-0.385039718	0.217	0.26	1	0	Atp5c1
0.00136556	-0.431997867	0.291	0.318	1	0	Ptprc
0.00140308	-0.327912823	0.375	0.394	1	0	Ywhaz
0.00147321	-0.286206598	0.417	0.429	1	0	Hnrnpk
0.00178138	-0.31781962	0.387	0.405	1	0	Pfdn5
0.00185756	-0.328678288	0.206	0.252	1	0	Eif3h
0.00192123	-0.322446616	0.222	0.266	1	0	Snrpd2
0.00211427	-0.339307451	0.474	0.448	1	0	Atpif1
0.00215867	-0.28187171	0.407	0.413	1	0	Chchd2
0.00262638	-0.261452182	0.561	0.516	1	0	Atp5j2
0.0027267	-0.363451469	0.334	0.358	1	0	Gm42418
0.0033252	-0.261695934	0.805	0.706	1	0	Fcer1g
0.00351476	-0.294847543	0.321	0.352	1	0	Ndufa6
0.00363377	-0.290297356	0.3	0.328	1	0	Hsp90aa1
0.00589287	-0.25379605	0.258	0.297	1	0	Nap1l1

0.00623433	-0.288252525	0.233	0.267	1	0	Snrpb	
0	2.07072349	0.597	0.072	0	1	Mmp9	**Neutrophil**
2.48E-291	2.23548758	0.588	0.078	3.27E-287	1	Mmp8	
1.36E-235	1.529574687	0.977	0.503	1.79E-231	1	Ltf	
6.44E-220	1.54976457	0.872	0.315	8.50E-216	1	Ifitm6	
1.52E-215	1.351701596	0.998	0.598	2.01E-211	1	Wfdc21	
3.96E-215	1.359220115	0.992	0.52	5.23E-211	1	Lcn2	
5.99E-211	1.25622012	1	0.994	7.91E-207	1	S100a9	
2.76E-207	-1.8732797	0.515	0.935	3.65E-203	1	Rps28	
8.83E-206	-1.287443647	0.84	0.988	1.17E-201	1	Rps29	
3.27E-198	-2.061092352	0.366	0.902	4.31E-194	1	Rpl35	
6.67E-197	-1.488448819	0.746	0.974	8.80E-193	1	Rpl41	
5.22E-192	1.204579335	1	0.999	6.89E-188	1	S100a8	
5.43E-186	1.287808841	0.929	0.405	7.16E-182	1	Pglyrp1	
1.37E-169	-1.736785955	0.368	0.877	1.81E-165	1	Rps18	
3.54E-168	-1.456812326	0.573	0.925	4.67E-164	1	Rps19	
5.14E-166	-1.69084374	0.391	0.882	6.78E-162	1	Ptma	
1.26E-163	-1.621906524	0.42	0.889	1.66E-159	1	Rpl13	
1.38E-159	1.118696926	0.998	0.836	1.82E-155	1	Ngp	
1.58E-158	-1.436534371	0.506	0.907	2.08E-154	1	Rpl18a	
6.14E-157	1.433583055	0.382	0.056	8.09E-153	1	Fpr2	
1.84E-152	-1.569016691	0.408	0.874	2.42E-148	1	Rps8	
5.80E-152	-1.155617104	0.719	0.958	7.65E-148	1	Rpl37a	
4.26E-151	-1.437268131	0.51	0.899	5.62E-147	1	Rpl32	
4.62E-150	1.275878873	0.828	0.394	6.09E-146	1	Anxa1	
2.69E-147	-1.672620181	0.292	0.819	3.55E-143	1	Rpl39	
8.98E-142	1.482306354	0.389	0.067	1.18E-137	1	AA467197	
6.53E-141	-0.96352817	0.786	0.969	8.62E-137	1	mt-Co3	
4.38E-140	-1.329799356	0.464	0.883	5.78E-136	1	Rpl38	
6.83E-139	-0.987197512	0.83	0.968	9.01E-135	1	Rps27	
5.80E-138	1.199861261	0.887	0.555	7.65E-134	1	S100a11	

7.48E-138	2.424450446	0.798	0.448	9.87E-134	1	Retnlg
4.12E-135	-1.919109487	0.141	0.722	5.43E-131	1	Gm2000
1.11E-134	-1.208113172	0.574	0.909	1.47E-130	1	Rps14
4.68E-134	1.507160683	0.382	0.068	6.17E-130	1	Ly6g
2.05E-132	-1.416231146	0.359	0.833	2.71E-128	1	Rps11
7.07E-130	-1.461708466	0.338	0.824	9.32E-126	1	Rps5
2.32E-128	-1.61798476	0.24	0.767	3.06E-124	1	Rps26
6.17E-128	-1.092583877	0.548	0.901	8.14E-124	1	Rpl35a
7.72E-128	-1.126490559	0.641	0.925	1.02E-123	1	Rpl37
1.22E-126	-1.186916175	0.504	0.891	1.61E-122	1	mt-Atp6
1.39E-126	-1.51846541	0.323	0.805	1.83E-122	1	Rpl36
1.92E-125	-1.308387523	0.363	0.83	2.53E-121	1	Ppia
2.99E-124	-1.33942043	0.435	0.844	3.94E-120	1	Rps23
2.26E-123	-1.273923604	0.408	0.843	2.98E-119	1	Rps16
1.20E-122	-1.768354848	0.128	0.692	1.58E-118	1	Gm10263
2.21E-122	0.933164587	0.998	0.925	2.92E-118	1	Camp
3.62E-121	-1.357161091	0.363	0.815	4.77E-117	1	Rplp2
1.08E-119	-1.510700752	0.263	0.762	1.43E-115	1	Rpl6
6.58E-119	-1.541078469	0.277	0.762	8.67E-115	1	Rplp1
6.63E-117	-1.525146195	0.258	0.751	8.74E-113	1	Rplp0
8.90E-117	-1.398457418	0.336	0.79	1.17E-112	1	Gm10076
2.62E-115	-1.283546358	0.365	0.817	3.46E-111	1	Rpl26
3.96E-114	-0.70903492	0.91	0.985	5.23E-110	1	mt-Co1
1.87E-112	-1.175725008	0.443	0.837	2.46E-108	1	Rps24
7.42E-112	-1.218484013	0.443	0.841	9.78E-108	1	Rpl13a
2.57E-111	-1.406079301	0.284	0.76	3.39E-107	1	Rps17
5.80E-111	-1.488596754	0.235	0.736	7.65E-107	1	Rpl10
1.21E-109	-1.139257401	0.479	0.849	1.60E-105	1	Rpl23a
2.03E-109	-1.697156497	0.177	0.685	2.68E-105	1	Rpl36a
2.05E-109	-1.389398251	0.296	0.763	2.71E-105	1	Rps4x
1.44E-107	-1.354565336	0.254	0.75	1.89E-103	1	Rpl28

9.31E-107	-1.278275304	0.33	0.781	1.23E-102	1	Rpl24
1.23E-106	-1.449098206	0.233	0.731	1.62E-102	1	Hmgb1
2.32E-106	-1.291465266	0.328	0.784	3.06E-102	1	Rps6
3.79E-106	-1.238713172	0.368	0.798	5.00E-102	1	Rpl17
3.80E-105	-1.21747622	0.397	0.806	5.01E-101	1	Rps3a1
7.46E-105	-1.102589783	0.468	0.835	9.84E-101	1	Rpl34
1.16E-104	-1.184033907	0.378	0.803	1.53E-100	1	Rpl21
5.38E-104	-1.190768412	0.351	0.797	7.10E-100	1	Rps3
7.50E-104	-1.38998503	0.231	0.724	9.89E-100	1	Rpsa
9.11E-104	-1.477688874	0.246	0.725	1.20E-99	1	Rpl14
2.11E-103	-1.308022205	0.3	0.754	2.78E-99	1	Rpl11
3.97E-103	1.171276405	0.552	0.183	5.24E-99	1	Cd177
1.76E-100	-1.024848314	0.536	0.871	2.33E-96	1	Rps27rt
7.87E-99	-1.59406144	0.137	0.641	1.04E-94	1	Rpl3
1.72E-98	-1.20705455	0.345	0.776	2.26E-94	1	Rpl23
4.21E-98	-1.878080112	0.357	0.774	5.55E-94	1	Tmsb10
7.84E-98	-1.333261191	0.267	0.729	1.03E-93	1	Rps7
8.72E-98	-1.392130643	0.229	0.701	1.15E-93	1	Rps20
1.10E-97	-1.186764448	0.363	0.783	1.45E-93	1	Rps15a
2.93E-95	-1.193376881	0.317	0.758	3.87E-91	1	Rpl19
7.24E-94	-1.395553138	0.24	0.696	9.54E-90	1	Rps12
1.55E-93	-1.345125154	0.225	0.698	2.04E-89	1	Eef1a1
6.09E-93	-1.1153467	0.37	0.778	8.03E-89	1	Rps15
1.46E-91	-1.175722218	0.338	0.755	1.92E-87	1	Rpl27a
7.18E-90	-1.126308517	0.336	0.762	9.48E-86	1	Rps25
1.31E-86	-1.460845148	0.151	0.617	1.73E-82	1	Rpl15
2.66E-84	-1.26181106	0.221	0.667	3.51E-80	1	Uba52
3.86E-84	-0.805279254	0.674	0.895	5.09E-80	1	Rps27a
2.70E-82	-1.161392245	0.277	0.706	3.57E-78	1	Rpl23a-ps3
4.87E-82	-1.699060263	0.05	0.507	6.43E-78	1	Rps18-ps3
1.32E-81	-1.107209797	0.332	0.742	1.75E-77	1	Rpl31

1.83E-80	-1.498593497	0.143	0.591	2.42E-76	1	Rps2
2.19E-79	-1.231001067	0.202	0.647	2.89E-75	1	Gm11808
2.69E-79	-1.191080345	0.258	0.678	3.55E-75	1	Rpl18
3.69E-79	-1.272334543	0.179	0.627	4.87E-75	1	Wdr89
1.39E-78	-1.878557852	0.267	0.67	1.83E-74	1	Crip1
4.29E-78	-1.346317811	0.172	0.612	5.66E-74	1	Rpl10a
1.62E-77	-1.414842012	0.109	0.556	2.13E-73	1	Snrpg
6.44E-77	-1.06585072	0.303	0.713	8.50E-73	1	Rpl27
7.64E-77	-1.589376973	0.074	0.511	1.01E-72	1	Rps26-ps1
8.12E-77	0.694402921	0.918	0.714	1.07E-72	1	Cd52
1.07E-76	-1.072491887	0.344	0.736	1.42E-72	1	mt-Cytb
1.08E-76	-1.148221953	0.246	0.683	1.42E-72	1	Rpl8
1.56E-76	-1.065462744	0.324	0.724	2.06E-72	1	Rps13
3.10E-74	-1.544225785	0.073	0.505	4.10E-70	1	Gm8730
4.04E-74	-1.249756083	0.33	0.715	5.33E-70	1	Plac8
2.77E-72	0.730316818	0.857	0.626	3.65E-68	1	Prdx5
5.61E-72	-1.214001403	0.183	0.608	7.39E-68	1	Rpl36al
8.77E-72	-1.316091538	0.128	0.561	1.16E-67	1	Rpl22
1.35E-71	-1.501727713	0.097	0.518	1.78E-67	1	Rpl12
3.87E-71	-1.161454537	0.242	0.653	5.11E-67	1	mt-Nd1
1.00E-70	-1.47183182	0.074	0.502	1.32E-66	1	Rpl10-ps3
2.59E-69	-1.160687603	0.212	0.624	3.41E-65	1	Rpl4
3.24E-68	-1.100630165	0.26	0.67	4.27E-64	1	Xist
5.01E-68	-1.475212944	0.057	0.468	6.60E-64	1	Rpl13-ps3
2.07E-67	-0.873228779	0.42	0.774	2.73E-63	1	Rpl9
2.67E-67	-1.491200081	0.067	0.473	3.52E-63	1	Gm10073
4.16E-66	-1.41591571	0.126	0.53	5.49E-62	1	Tubb5
5.00E-66	-0.926088045	0.351	0.739	6.59E-62	1	Tpt1
4.80E-64	-0.95040535	0.303	0.689	6.33E-60	1	Rps10
3.39E-63	-1.053720481	0.252	0.639	4.48E-59	1	Rps21
4.23E-63	-1.594927492	0.124	0.509	5.58E-59	1	Lgals1

2.46E-62	-1.336509281	0.074	0.471	3.24E-58	1	Gnb2l1
4.25E-62	-1.279515087	0.151	0.54	5.60E-58	1	Cbx3
5.82E-62	-1.388144579	0.109	0.501	7.68E-58	1	Ly6e
1.01E-61	-1.062432399	0.202	0.603	1.33E-57	1	mt-Nd4
1.45E-61	-1.343241371	0.103	0.489	1.92E-57	1	Rps12-ps3
2.83E-61	1.10437645	0.307	0.088	3.73E-57	1	Cd33
1.66E-60	-0.574590969	0.775	0.932	2.19E-56	1	Fau
4.34E-59	1.075746401	0.292	0.083	5.73E-55	1	Lrg1
6.72E-59	-1.305060294	0.084	0.465	8.87E-55	1	Gm9493
7.56E-59	-1.186952867	0.151	0.536	9.97E-55	1	Rpl22l1
1.27E-57	-1.32511002	0.052	0.422	1.67E-53	1	Gm8186
1.64E-57	-0.64471676	0.679	0.89	2.16E-53	1	Rps9
1.14E-55	-1.135575569	0.134	0.508	1.51E-51	1	Rps27l
2.83E-55	-1.153257028	0.132	0.5	3.74E-51	1	Rpl29
5.77E-55	-1.940931654	0.063	0.414	7.61E-51	1	Hist1h2ap
1.22E-54	0.313926891	1	0.995	1.61E-50	1	Tmsb4x
6.72E-53	0.992154742	0.408	0.166	8.86E-49	1	Adpgk
1.12E-52	-0.938572442	0.208	0.577	1.47E-48	1	Rpl7
2.58E-52	-0.839803739	0.328	0.683	3.41E-48	1	mt-Co2
2.88E-51	-1.290679369	0.046	0.385	3.80E-47	1	Snrpf
1.45E-50	-1.048287949	0.319	0.66	1.92E-46	1	Cst3
1.90E-50	0.944043473	0.481	0.23	2.50E-46	1	Tmcc1
2.23E-50	-1.21017423	0.088	0.431	2.95E-46	1	Rpl36-ps3
2.56E-50	-0.831737039	0.319	0.666	3.38E-46	1	Sec61g
5.80E-50	-1.275026891	0.071	0.411	7.65E-46	1	Npm1
1.22E-49	-1.070885855	0.103	0.455	1.60E-45	1	Hint1
1.42E-49	-1.009676508	0.145	0.504	1.87E-45	1	Rpl27-ps3
1.34E-48	0.325835161	0.998	0.898	1.77E-44	1	Lyz2
1.95E-48	-1.095365083	0.107	0.457	2.57E-44	1	Prdx1
2.31E-48	0.762433325	0.662	0.436	3.05E-44	1	Hp
5.09E-48	-0.616981832	0.628	0.837	6.71E-44	1	H2afz

8.13E-48	-1.217289196	0.067	0.394	1.07E-43	1	Dek
1.43E-47	-0.645213553	0.531	0.814	1.89E-43	1	Gm9843
2.88E-47	-1.121940626	0.109	0.446	3.79E-43	1	Hsp90ab1
5.67E-47	-0.946276342	0.179	0.528	7.49E-43	1	Rpl7a
8.67E-47	-1.175641317	0.055	0.377	1.14E-42	1	Rpl6l
1.46E-46	-0.900516849	0.227	0.573	1.93E-42	1	Hspa8
1.51E-46	0.698664769	0.718	0.482	1.99E-42	1	Cybb
1.57E-44	-0.956503663	0.137	0.479	2.08E-40	1	Naca
2.92E-44	-1.152902125	0.078	0.393	3.85E-40	1	Tuba1b
6.72E-44	1.074283464	0.479	0.261	8.87E-40	1	Slpi
7.12E-44	-0.98674943	0.122	0.454	9.39E-40	1	Serbp1
7.14E-44	0.876947591	0.418	0.192	9.42E-40	1	Mcemp1
7.62E-44	-1.19832253	0.057	0.367	1.01E-39	1	Set
8.84E-44	-0.936278639	0.143	0.475	1.17E-39	1	Rpl9-ps6
1.42E-43	0.8746443	0.313	0.115	1.87E-39	1	Syne1
3.71E-43	-0.970098578	0.128	0.458	4.89E-39	1	Rpl5
4.06E-43	-1.164343843	0.048	0.346	5.36E-39	1	Gm10036
9.89E-43	-0.966873214	0.135	0.46	1.30E-38	1	Ybx1
2.62E-42	-1.158799581	0.057	0.36	3.45E-38	1	Hnrnpa1
1.12E-41	-1.172755646	0.023	0.307	1.47E-37	1	Gm10269
1.41E-41	-0.987106551	0.155	0.475	1.86E-37	1	Psma7
3.72E-41	-0.684056311	0.445	0.729	4.90E-37	1	Sub1
2.86E-40	-1.336921419	0.053	0.34	3.77E-36	1	Ms4a6c
3.03E-40	-0.962955867	0.109	0.422	3.99E-36	1	mt-Atp8
3.55E-40	0.878913228	0.462	0.246	4.68E-36	1	Ncf1
7.51E-40	0.476702007	0.798	0.531	9.91E-36	1	Chil3
1.34E-39	-0.844736454	0.176	0.498	1.77E-35	1	Ndufa4
2.07E-39	-0.74210643	0.277	0.594	2.73E-35	1	Hnrnpa2b1
2.91E-39	0.8855893	0.326	0.131	3.84E-35	1	Itgam
3.55E-39	-0.770541092	0.26	0.587	4.68E-35	1	Calm2
9.53E-39	-0.894019076	0.135	0.452	1.26E-34	1	Eef2

1.40E-38	-1.005600921	0.135	0.438	1.84E-34	1	Stmn1
1.66E-38	0.905894855	0.338	0.143	2.20E-34	1	Cd9
1.94E-38	-0.922114857	0.128	0.433	2.56E-34	1	Anp32b
3.25E-38	-0.958711695	0.128	0.427	4.29E-34	1	Dbi
5.27E-38	-0.840530196	0.162	0.476	6.95E-34	1	Hnrnpa3
1.24E-37	-0.912627448	0.103	0.4	1.64E-33	1	H2afv
4.41E-37	-1.012053738	0.052	0.329	5.81E-33	1	Polr2l
6.13E-37	-1.033742725	0.082	0.369	8.09E-33	1	H2afy
7.93E-37	-0.984294579	0.282	0.566	1.05E-32	1	Psap
2.10E-36	-0.814905718	0.181	0.49	2.78E-32	1	Rpl30
4.05E-36	-0.830195945	0.153	0.452	5.34E-32	1	Sumo2
4.68E-36	-0.889941995	0.109	0.397	6.17E-32	1	Tmem256
1.02E-35	-1.000486076	0.082	0.359	1.35E-31	1	Nop10
1.52E-35	-1.012870771	0.067	0.336	2.01E-31	1	Atp5g1
2.83E-35	-0.562535437	0.475	0.765	3.73E-31	1	H3f3b
3.61E-35	-0.945992857	0.084	0.358	4.76E-31	1	Nucks1
4.60E-35	0.789938487	0.483	0.274	6.07E-31	1	Mgst1
4.93E-35	-0.999357637	0.076	0.347	6.51E-31	1	Ranbp1
9.12E-35	-0.878347845	0.126	0.408	1.20E-30	1	Eif2s2
1.02E-34	-1.230447498	0.034	0.284	1.34E-30	1	S100a10
1.70E-34	-1.014979449	0.061	0.325	2.24E-30	1	mt-Nd2
2.67E-34	-1.056067705	0.076	0.34	3.52E-30	1	Top2a
5.26E-34	-1.03247921	0.086	0.355	6.94E-30	1	2810417H13Rik
1.10E-33	-1.02146527	0.08	0.344	1.45E-29	1	Npc2
2.31E-33	0.889803182	0.286	0.117	3.05E-29	1	Vasp
3.25E-33	0.81134319	0.448	0.259	4.28E-29	1	Aldh2
3.25E-33	0.758683714	0.471	0.281	4.29E-29	1	Cd24a
5.68E-33	-0.902782631	0.057	0.314	7.49E-29	1	Nap1l1
1.17E-32	-0.942575216	0.071	0.326	1.54E-28	1	Tsix
1.28E-32	-0.891961473	0.08	0.347	1.68E-28	1	Eif3f
1.38E-32	-0.717610419	0.235	0.536	1.83E-28	1	Pabpc1

3.00E-32	-0.924313404	0.088	0.347	3.95E-28	1	Hsp90aa1
4.25E-32	0.802876259	0.406	0.213	5.60E-28	1	Cpne3
5.76E-32	-1.16216444	0.038	0.277	7.60E-28	1	Gm9844
6.88E-32	-0.800385213	0.115	0.388	9.08E-28	1	Ran
1.16E-31	0.496088584	0.781	0.655	1.53E-27	1	Arhgdib
1.48E-31	0.677771617	0.698	0.517	1.95E-27	1	S100a6
3.74E-31	-1.114388934	0.027	0.254	4.93E-27	1	Zeb2
4.06E-31	-0.796141572	0.198	0.473	5.36E-27	1	Atpif1
4.55E-31	-0.813825154	0.13	0.398	6.01E-27	1	Btf3
4.98E-31	-0.652108227	0.208	0.502	6.57E-27	1	Morf4l1
2.59E-30	-0.515241554	0.525	0.764	3.41E-26	1	Gpx1
4.69E-30	-0.551429763	0.412	0.688	6.19E-26	1	Cox4i1
1.10E-29	-0.549571905	0.334	0.627	1.45E-25	1	Cox7c
1.26E-29	-0.902266103	0.065	0.304	1.67E-25	1	Eef1g
3.24E-29	-1.147597648	0.061	0.293	4.27E-25	1	Ccr2
7.03E-29	0.659742888	0.552	0.383	9.28E-25	1	Dstn
9.87E-29	-0.752656204	0.086	0.337	1.30E-24	1	Snrpe
1.13E-28	-0.859215929	0.067	0.304	1.49E-24	1	Nsa2
1.15E-28	-0.517098902	0.473	0.716	1.52E-24	1	Oaz1
2.72E-28	-0.791189122	0.092	0.338	3.59E-24	1	Eef1b2
3.01E-28	-0.774027296	0.08	0.321	3.97E-24	1	Erh
3.69E-28	-0.690261943	0.248	0.526	4.87E-24	1	Mbnl1
7.92E-28	-0.773615664	0.09	0.332	1.04E-23	1	Cycs
8.99E-28	-0.83783204	0.065	0.296	1.19E-23	1	Bola2
1.41E-27	0.792431888	0.332	0.168	1.85E-23	1	Ckap4
1.86E-27	-0.997802824	0.084	0.313	2.45E-23	1	Pdcd4
3.05E-27	-0.810717147	0.069	0.297	4.03E-23	1	mt-Nd4l
3.48E-27	-0.752507367	0.103	0.344	4.60E-23	1	Slc25a5
4.00E-27	-0.759677119	0.143	0.393	5.28E-23	1	Srrm2
4.76E-27	-0.952608515	0.055	0.273	6.28E-23	1	Ncl
5.71E-27	-0.799598598	0.073	0.3	7.53E-23	1	Hnrnpab

6.61E-27	-0.728323159	0.109	0.351	8.71E-23	1	Lsm5
1.02E-26	-0.646208388	0.181	0.454	1.35E-22	1	Serinc3
1.08E-26	-0.726868499	0.101	0.34	1.42E-22	1	Hnrnpu
1.26E-26	-0.645175959	0.185	0.449	1.66E-22	1	Rbm3
1.30E-26	-0.767270362	0.09	0.324	1.72E-22	1	Hnrnpm
2.46E-26	-0.785403963	0.059	0.281	3.25E-22	1	Snrpd2
2.61E-26	-0.761349308	0.076	0.305	3.44E-22	1	Eif4a1
4.51E-26	-0.721011728	0.118	0.357	5.95E-22	1	Srsf3
4.70E-26	-0.698062508	0.147	0.398	6.20E-22	1	Eif5a
5.66E-26	-0.792270211	0.082	0.305	7.47E-22	1	Tagln2
1.93E-25	-1.327622492	0.08	0.29	2.54E-21	1	Prtn3
2.92E-25	-0.811467546	0.046	0.251	3.85E-21	1	Usp50
3.85E-25	-0.63824911	0.221	0.471	5.07E-21	1	Uqcr11
6.92E-25	-0.642691924	0.143	0.393	9.13E-21	1	Srsf2
9.33E-25	-0.734615133	0.067	0.282	1.23E-20	1	Snrpb
2.41E-24	-0.751364431	0.065	0.273	3.19E-20	1	Alyref
2.58E-24	-0.488712141	0.24	0.517	3.40E-20	1	Pcbp2
3.90E-24	-0.767365788	0.053	0.256	5.14E-20	1	Cks1b
3.93E-24	-0.773029867	0.069	0.28	5.18E-20	1	Prrc2c
4.44E-24	-0.550469699	0.214	0.475	5.85E-20	1	Uqcr10
5.19E-24	-0.738132257	0.084	0.302	6.85E-20	1	Tmpo
5.67E-24	-0.553908965	0.281	0.542	7.48E-20	1	Atp5h
1.23E-23	-0.772889931	0.057	0.26	1.62E-19	1	Calr
1.98E-23	-0.553237964	0.21	0.463	2.61E-19	1	Hnrnpf
2.64E-23	-0.529961453	0.271	0.528	3.48E-19	1	Sec61b
5.32E-23	-0.73550213	0.149	0.376	7.02E-19	1	H2-K1
7.11E-23	-0.702355926	0.101	0.316	9.38E-19	1	Atp5g2
1.23E-22	-0.721072413	0.069	0.27	1.62E-18	1	Eif3a
3.14E-22	-0.640959222	0.088	0.299	4.14E-18	1	Atp5b
3.52E-22	-0.513729826	0.294	0.546	4.65E-18	1	Tma7
4.49E-22	-0.590502086	0.181	0.417	5.93E-18	1	Tomm7

9.30E-22	-0.696602582	0.141	0.359	1.23E-17	1	Hsp90b1
9.48E-22	0.7242914	0.265	0.127	1.25E-17	1	Samd9l
1.08E-21	-0.584316157	0.141	0.367	1.42E-17	1	Cox7b
1.08E-21	-1.103403412	0.174	0.375	1.43E-17	1	Ifi27l2a
1.18E-21	-0.425558345	0.395	0.644	1.55E-17	1	Cox6c
1.33E-21	-0.723067431	0.065	0.256	1.76E-17	1	Eif5b
1.81E-21	-0.569604881	0.176	0.409	2.39E-17	1	Smdt1
1.98E-21	-0.462260186	0.571	0.788	2.61E-17	1	B2m
2.87E-21	-0.628856914	0.088	0.289	3.78E-17	1	Atp5o
3.55E-21	-0.707375703	0.084	0.28	4.68E-17	1	Gnas
5.13E-21	-0.482628261	0.231	0.48	6.77E-17	1	Ndufa2
5.74E-21	-0.602417792	0.109	0.318	7.58E-17	1	Sfpq
5.80E-21	-0.643833091	0.078	0.276	7.66E-17	1	Atp5g3
6.73E-21	-0.531407668	0.263	0.502	8.88E-17	1	Uqcrh
7.65E-21	-0.527292958	0.189	0.425	1.01E-16	1	Uqcrq
9.10E-21	-0.655293915	0.073	0.263	1.20E-16	1	Eif3h
1.51E-20	-0.60997739	0.097	0.299	2.00E-16	1	Tra2b
1.87E-20	-0.583185267	0.092	0.293	2.47E-16	1	Arglu1
1.98E-20	-0.537948699	0.156	0.383	2.62E-16	1	Son
2.03E-20	-0.469194648	0.378	0.623	2.68E-16	1	Atp5e
2.55E-20	-0.454513776	0.347	0.593	3.36E-16	1	Cox8a
2.72E-20	-0.604260082	0.122	0.331	3.59E-16	1	Eif3k
2.87E-20	0.503992944	0.578	0.454	3.79E-16	1	Rac2
4.40E-20	-0.632598384	0.067	0.251	5.81E-16	1	Dpm3
5.22E-20	-1.569899236	0.084	0.261	6.89E-16	1	Elane
5.37E-20	-0.464542221	0.296	0.54	7.08E-16	1	Atp5j2
5.38E-20	-0.608503131	0.067	0.253	7.09E-16	1	Ptges3
7.36E-20	0.63000216	0.408	0.268	9.71E-16	1	Lcp1
1.08E-19	-0.683890374	0.071	0.251	1.42E-15	1	Metap2
1.48E-19	0.390552241	0.771	0.703	1.95E-15	1	Myl6
1.52E-19	-0.429580309	0.679	0.833	2.00E-15	1	Ftl1

2.49E-19	-0.681220036	0.082	0.266	3.28E-15	1	Psmb8
5.41E-19	-0.598476819	0.109	0.303	7.14E-15	1	Psmb1
6.32E-19	-0.540982579	0.166	0.38	8.33E-15	1	BC005537
7.06E-19	0.650162978	0.311	0.176	9.31E-15	1	Hcst
9.15E-19	-0.528856407	0.122	0.323	1.21E-14	1	Pcbp1
9.60E-19	-0.577769163	0.088	0.272	1.27E-14	1	Lsm6
1.11E-18	-0.56285112	0.095	0.284	1.46E-14	1	Tmed2
1.35E-18	-0.587733841	0.099	0.288	1.78E-14	1	Ywhae
1.74E-18	-0.47850807	0.219	0.446	2.30E-14	1	Hnrnpk
1.85E-18	-0.584177232	0.073	0.252	2.44E-14	1	Park7
2.42E-18	-0.626736816	0.076	0.254	3.19E-14	1	Pycard
3.24E-18	-0.549859596	0.147	0.351	4.27E-14	1	Pomp
5.52E-18	-0.487220083	0.206	0.426	7.28E-14	1	Rbm39
8.25E-18	-0.513084008	0.447	0.66	1.09E-13	1	Fth1
1.64E-17	-0.482708695	0.16	0.366	2.17E-13	1	Ndufa6
1.99E-17	-0.586380066	0.094	0.27	2.62E-13	1	Atp5c1
3.46E-17	-1.165415516	0.116	0.284	4.56E-13	1	Mpo
5.51E-17	0.287079314	0.876	0.832	7.26E-13	1	Pfn1
5.56E-17	-0.539664108	0.115	0.299	7.34E-13	1	Cmtm7
6.18E-17	-0.655930644	0.099	0.271	8.15E-13	1	Cebpb
7.05E-17	-0.480143213	0.212	0.429	9.30E-13	1	Atox1
8.10E-17	-0.406674553	0.344	0.569	1.07E-12	1	Cox6b1
8.16E-17	-0.49074404	0.124	0.314	1.08E-12	1	Ndufc1
1.12E-16	-0.489067726	0.156	0.351	1.48E-12	1	Cox5a
1.59E-16	-0.453209117	0.195	0.399	2.10E-12	1	Minos1
2.02E-16	-0.436876071	0.218	0.43	2.67E-12	1	2010107E04Rik
5.64E-16	-0.518082908	0.097	0.266	7.44E-12	1	Timm13
7.25E-16	-0.570438259	0.118	0.291	9.56E-12	1	Reep5
1.21E-15	0.302754944	0.792	0.707	1.60E-11	1	Fcer1g
1.97E-15	-0.397579493	0.269	0.486	2.60E-11	1	Cox5b
3.10E-15	0.51711868	0.515	0.416	4.09E-11	1	Tkt

3.85E-15	-0.509058081	0.153	0.333	5.08E-11	1	Gm10116
5.81E-15	0.616644649	0.284	0.167	7.66E-11	1	Cnn2
1.06E-14	0.587857013	0.323	0.202	1.40E-10	1	Serpinb1a
1.20E-14	-0.460067651	0.101	0.265	1.58E-10	1	Ndufb10
1.24E-14	-0.328766174	0.46	0.666	1.63E-10	1	Atp5l
1.43E-14	-0.485864284	0.099	0.262	1.89E-10	1	Rbm25
1.70E-14	-0.440866419	0.739	0.783	2.24E-10	1	Hmgb2
2.52E-14	-0.434423878	0.141	0.318	3.33E-10	1	Uqcrb
2.98E-14	-0.422420919	0.153	0.333	3.93E-10	1	Nedd8
3.47E-14	-0.360161557	0.227	0.428	4.57E-10	1	Chchd2
4.10E-14	-0.482124066	0.099	0.253	5.40E-10	1	Ube2s
4.53E-14	-0.431422368	0.177	0.363	5.98E-10	1	Ptpn18
4.77E-14	-0.43267945	0.187	0.373	6.29E-10	1	Arl6ip1
5.44E-14	-0.410102769	0.128	0.301	7.17E-10	1	Clta
6.30E-14	0.584543233	0.256	0.147	8.32E-10	1	Degs1
7.18E-14	-0.458829132	0.128	0.294	9.47E-10	1	Anapc13
1.12E-13	0.304804614	0.744	0.679	1.47E-09	1	Cyba
1.60E-13	-0.430658943	0.109	0.264	2.11E-09	1	Ndufb2
1.93E-13	-0.475043975	0.109	0.264	2.55E-09	1	Ndufb3
2.00E-13	-0.35941447	0.319	0.531	2.64E-09	1	Cox6a1
2.45E-13	-0.309268639	0.29	0.505	3.23E-09	1	Atp5j
2.60E-13	-0.379402285	0.12	0.284	3.43E-09	1	Romo1
3.18E-13	-0.414539082	0.141	0.306	4.20E-09	1	Sarnp
3.38E-13	-0.380397555	0.162	0.341	4.46E-09	1	Ndufa7
3.43E-13	-0.384474404	0.214	0.401	4.52E-09	1	Tmem258
4.75E-13	0.655183829	0.288	0.18	6.27E-09	1	Prr13
9.00E-13	-0.410399254	0.135	0.297	1.19E-08	1	Luc7l2
9.00E-13	-0.348002805	0.292	0.491	1.19E-08	1	H2afj
1.03E-12	0.513168749	0.366	0.257	1.35E-08	1	Clec4a2
1.67E-12	-0.336781598	0.351	0.55	2.20E-08	1	Atp5k
2.29E-12	-0.341715765	0.248	0.439	3.01E-08	1	Arpp19

2.69E-12	0.644281201	0.273	0.173	3.55E-08	1	Gda
3.37E-12	-0.342851755	0.195	0.374	4.44E-08	1	Gpx4
3.94E-12	-0.425961791	0.12	0.268	5.20E-08	1	Ndufb8
4.07E-12	-0.378679872	0.109	0.259	5.37E-08	1	Hnrnph1
4.20E-12	0.481927256	0.324	0.214	5.54E-08	1	Cd63
5.88E-12	-0.437891614	0.109	0.254	7.75E-08	1	Zfp36l2
6.64E-12	-0.341640041	0.3	0.491	8.76E-08	1	Cox7a2
1.06E-11	-0.302070751	0.151	0.313	1.39E-07	1	Ost4
1.85E-11	-0.368170567	0.147	0.304	2.44E-07	1	D8Ertd738e
2.48E-11	-0.369503586	0.187	0.35	3.26E-07	1	Clic1
2.67E-11	-0.365318412	0.143	0.295	3.53E-07	1	Calm3
4.77E-11	0.480087343	0.433	0.346	6.29E-07	1	Msrb1
1.11E-10	-0.328611053	0.166	0.32	1.47E-06	1	Canx
1.21E-10	-0.403019122	0.12	0.256	1.60E-06	1	Pgls
1.29E-10	-0.308885275	0.16	0.313	1.70E-06	1	Sumo1
2.43E-10	-0.312570227	0.134	0.274	3.21E-06	1	Tmem167
2.84E-10	-0.313904922	0.137	0.28	3.75E-06	1	Ndufb9
3.90E-10	0.589622858	0.281	0.193	5.14E-06	1	Hipk1
4.09E-10	0.540295774	0.302	0.209	5.39E-06	1	Lims1
4.59E-10	-0.307056774	0.135	0.276	6.06E-06	1	Ndufs6
5.04E-10	-0.275520584	0.416	0.6	6.64E-06	1	Gm26917
5.58E-10	0.515597915	0.347	0.259	7.36E-06	1	Cotl1
1.30E-09	-0.275610803	0.286	0.466	1.71E-05	1	Usmg5
2.61E-09	-0.294052388	0.195	0.338	3.44E-05	1	Cks2
3.18E-09	0.540781609	0.265	0.18	4.19E-05	1	Pgd
4.27E-09	-0.321641139	0.214	0.369	5.64E-05	1	Gm42418
4.29E-09	-0.252378365	0.132	0.265	5.66E-05	1	Ndufs5
5.15E-09	0.254245524	0.712	0.684	6.79E-05	1	Actg1
5.22E-09	-0.3224506	0.168	0.305	6.88E-05	1	Laptm5
7.99E-09	0.376268873	0.494	0.441	0.000105339	1	Gmfg
2.42E-08	0.522199679	0.319	0.244	0.000319192	1	Ostf1

2.44E-08	-0.251436612	0.135	0.26	0.000321683	1	Psmb3	
2.96E-08	-0.296815035	0.405	0.554	0.000389894	1	Ubb	
3.20E-08	0.468081276	0.256	0.175	0.000422743	1	Cebpe	
6.11E-08	-0.280710493	0.145	0.266	0.000805394	1	Wtap	
8.98E-08	-0.31021347	0.151	0.267	0.001184637	1	Gm10282	
1.03E-07	0.52563404	0.261	0.187	0.001359638	1	R3hdm4	
1.66E-07	0.344706957	0.5	0.447	0.002194756	1	Alox5ap	
2.45E-07	-0.25363953	0.193	0.322	0.003226605	1	Arf5	
3.38E-07	0.4526187	0.347	0.278	0.004456239	1	Cd44	
3.40E-07	0.346026737	0.515	0.475	0.004487934	1	F630028O10Rik	
4.07E-07	0.473361972	0.29	0.218	0.00536384	1	Spi1	
5.85E-07	0.375487704	0.458	0.41	0.007723111	1	Gapdh	
5.90E-07	-0.250249933	0.246	0.376	0.00778428	1	Emp3	
7.54E-07	-0.254949959	0.143	0.253	0.009952321	1	Scand1	
8.51E-07	0.449785036	0.345	0.287	0.011224894	1	Pkm	
1.10E-06	-0.303485785	0.155	0.263	0.01444753	1	Btg1	
1.94E-06	0.380705507	0.321	0.252	0.025589236	1	Lmo4	
7.81E-06	0.405517766	0.403	0.369	0.102959438	1	Arpc5	
1.15E-05	0.350239442	0.279	0.214	0.151998925	1	Smim14	
1.85E-05	-0.307240594	0.542	0.621	0.244136262	1	Ly6c2	
2.45E-05	0.343514535	0.403	0.362	0.322849813	1	Gsr	
2.93E-05	0.413518348	0.305	0.251	0.385866134	1	Flna	
6.87E-05	0.356632822	0.368	0.32	0.905614221	1	Scp2	
0.00011521	0.411165466	0.305	0.267	1	1	Aldoa	
0.00117658	-0.254490752	0.511	0.594	1	1	Hmgn2	
1.33E-229	1.304980527	1	0.999	1.75E-225	2	S100a8	**Neutrophil**
2.06E-214	1.267085648	1	0.994	2.72E-210	2	S100a9	
3.25E-202	1.284437687	0.994	0.52	4.29E-198	2	Lcn2	
1.64E-198	1.261966447	0.998	0.598	2.17E-194	2	Wfdc21	
2.86E-198	1.2631261	1	0.925	3.77E-194	2	Camp	
1.66E-194	1.210636143	0.998	0.501	2.19E-190	2	Ltf	

2.94E-193	1.263990796	1	0.836	3.87E-189	2	Ngp
8.14E-171	-1.525007909	0.626	0.925	1.07E-166	2	Rps28
5.83E-164	-1.271998174	0.834	0.966	7.69E-160	2	Rpl41
2.35E-162	-1.041159555	0.915	0.981	3.10E-158	2	Rps29
9.98E-155	1.077704017	0.915	0.407	1.32E-150	2	Pglyrp1
2.81E-150	1.268075524	0.811	0.321	3.70E-146	2	Ifitm6
1.05E-148	-1.543793829	0.559	0.884	1.38E-144	2	Rpl35
1.20E-147	-1.359393543	0.574	0.9	1.59E-143	2	Rpl18a
4.05E-146	-1.120286602	0.771	0.953	5.35E-142	2	Rpl37a
3.59E-139	-1.249547622	0.669	0.917	4.74E-135	2	Rps19
5.88E-138	-1.406284716	0.545	0.878	7.75E-134	2	Rpl13
3.70E-137	-0.982492502	0.834	0.967	4.87E-133	2	Rps27
6.25E-132	-1.387263726	0.52	0.863	8.24E-128	2	Rps18
2.33E-131	0.980666258	0.931	0.519	3.07E-127	2	Chil3
1.52E-123	1.047944462	0.838	0.393	2.00E-119	2	Anxa1
3.20E-123	-1.344469677	0.41	0.828	4.21E-119	2	Rps11
4.70E-123	-1.216106	0.605	0.89	6.20E-119	2	Rpl32
2.28E-122	-1.404482235	0.408	0.818	3.01E-118	2	Rps5
5.22E-122	-1.12353688	0.624	0.904	6.89E-118	2	Rps14
3.61E-121	-1.082223497	0.694	0.92	4.77E-117	2	Rpl37
4.76E-121	-1.315086303	0.468	0.841	6.28E-117	2	Rps23
4.95E-116	-1.253063408	0.451	0.84	6.53E-112	2	Rpl13a
6.85E-113	-1.46873876	0.283	0.761	9.04E-109	2	Rplp1
5.01E-109	-0.817136602	0.863	0.963	6.61E-105	2	mt-Co3
3.31E-107	-1.52162957	0.235	0.714	4.37E-103	2	Gm2000
4.82E-107	-1.141216802	0.482	0.836	6.36E-103	2	Rps16
1.06E-106	-1.060065219	0.578	0.884	1.40E-102	2	mt-Atp6
1.84E-106	-1.172392572	0.555	0.861	2.43E-102	2	Rps8
2.21E-105	-1.246059541	0.434	0.807	2.91E-101	2	Rpl39
1.94E-104	-1.093751092	0.503	0.847	2.56E-100	2	Rpl23a
6.63E-104	-1.321508085	0.303	0.762	8.75E-100	2	Rps4x

1.39E-102	-1.047058388	0.59	0.872	1.83E-98	2	Rpl38
1.69E-101	-1.043908817	0.543	0.87	2.22E-97	2	Rps27rt
4.12E-101	-1.255584302	0.414	0.783	5.43E-97	2	Gm10076
1.36E-100	-1.477030531	0.202	0.685	1.80E-96	2	Gm10263
3.40E-100	-1.090836614	0.578	0.865	4.49E-96	2	Ptma
1.05E-97	-0.907523349	0.638	0.898	1.38E-93	2	Rps27a
2.81E-97	-1.137366034	0.457	0.806	3.70E-93	2	Rplp2
3.35E-97	-1.317979902	0.328	0.744	4.41E-93	2	Rplp0
5.77E-97	-1.330939469	0.291	0.731	7.62E-93	2	Rpl10
1.71E-96	-1.228550363	0.435	0.794	2.26E-92	2	Rpl36
3.00E-96	-1.13854965	0.445	0.801	3.95E-92	2	Rps3a1
1.21E-95	-1.109262276	0.453	0.809	1.60E-91	2	Rpl26
3.70E-95	-1.253680286	0.351	0.754	4.88E-91	2	Rpl6
2.92E-94	-1.18000257	0.383	0.779	3.85E-90	2	Rps6
2.36E-91	-1.056576946	0.414	0.799	3.11E-87	2	Rpl21
5.43E-91	-1.215775955	0.364	0.756	7.16E-87	2	Rps26
1.61E-90	-1.143197389	0.395	0.772	2.12E-86	2	Rpl23
4.51E-90	-1.184048487	0.358	0.753	5.95E-86	2	Rpl27a
8.83E-89	-0.980585456	0.509	0.831	1.16E-84	2	Rps24
1.31E-88	-1.306414469	0.256	0.695	1.73E-84	2	Eef1a1
6.46E-88	-1.31655253	0.329	0.717	8.53E-84	2	Rpl14
4.15E-86	-1.049748267	0.443	0.791	5.47E-82	2	Rpl17
6.95E-86	-1.115551523	0.372	0.753	9.17E-82	2	Rpl19
7.32E-86	-1.200326544	0.318	0.716	9.65E-82	2	Rpsa
2.37E-85	-1.072301741	0.424	0.778	3.13E-81	2	Rps15a
4.10E-85	-1.359392839	0.258	0.678	5.40E-81	2	Rpl36a
4.66E-85	-0.804559992	0.692	0.888	6.15E-81	2	Rpl35a
4.63E-83	-1.396102202	0.197	0.636	6.11E-79	2	Rpl3
7.49E-83	-1.156624392	0.328	0.723	9.88E-79	2	Rps7
1.41E-81	-1.097328153	0.37	0.747	1.86E-77	2	Rpl11
2.35E-81	-0.576396455	0.942	0.982	3.10E-77	2	mt-Co1

6.31E-80	-1.073226132	0.368	0.74	8.32E-76	2	Rpl28
1.34E-79	-1.139520045	0.303	0.703	1.77E-75	2	Rpl23a-ps3
1.93E-79	-1.772705686	0.453	0.765	2.55E-75	2	Tmsb10
5.40E-79	-1.905613504	0.276	0.669	7.12E-75	2	Crip1
7.31E-78	-1.063037537	0.405	0.749	9.64E-74	2	Rps17
7.60E-77	-0.881225461	0.547	0.828	1.00E-72	2	Rpl34
5.15E-76	-0.991434303	0.428	0.772	6.79E-72	2	Rpl24
7.42E-76	-0.946813719	0.472	0.786	9.79E-72	2	Rps3
7.56E-76	-0.653961277	0.775	0.932	9.97E-72	2	Fau
2.59E-75	-0.941075115	0.424	0.773	3.41E-71	2	Rps15
1.11E-74	-1.002370528	0.358	0.738	1.46E-70	2	Tpt1
4.21E-74	-1.148306497	0.304	0.69	5.55E-70	2	Rps12
1.72E-73	-1.136942973	0.266	0.663	2.27E-69	2	Uba52
6.83E-73	-1.266501941	0.349	0.713	9.01E-69	2	Plac8
3.02E-72	1.005297434	0.501	0.188	3.98E-68	2	Cd177
4.53E-70	-1.070684645	0.322	0.692	5.98E-66	2	Rps20
4.86E-70	-1.243639265	0.206	0.608	6.41E-66	2	Rpl10a
7.53E-70	-1.375699985	0.193	0.586	9.93E-66	2	Rps2
1.12E-69	-1.125709945	0.245	0.643	1.47E-65	2	Gm11808
7.34E-69	-1.7374683	0.106	0.51	9.68E-65	2	Lgals1
2.34E-68	-1.013370404	0.374	0.733	3.09E-64	2	mt-Cytb
2.70E-68	-1.073218746	0.295	0.674	3.56E-64	2	Rpl18
2.01E-67	-1.201701712	0.22	0.61	2.66E-63	2	Rpl15
1.77E-66	-1.046352234	0.306	0.678	2.33E-62	2	Rpl8
2.28E-66	-0.904676109	0.435	0.753	3.00E-62	2	Rps25
1.69E-65	-1.112420815	0.272	0.651	2.23E-61	2	mt-Nd1
2.61E-65	-1.397400844	0.104	0.502	3.45E-61	2	Rps18-ps3
1.59E-64	-0.952720275	0.383	0.719	2.09E-60	2	Rps13
1.66E-64	-1.098031574	0.239	0.621	2.18E-60	2	Wdr89
1.92E-63	-0.972767641	0.389	0.717	2.54E-59	2	Hmgb1
2.43E-62	-0.922506711	0.362	0.708	3.21E-58	2	Rpl27

4.04E-62	-1.405550125	0.11	0.5	5.33E-58	2	Ly6e
4.84E-62	-1.406771023	0.085	0.471	6.38E-58	2	Gm10073
1.54E-61	-0.936923049	0.414	0.735	2.03E-57	2	Rpl31
3.61E-61	-1.146371463	0.17	0.557	4.76E-57	2	Rpl22
6.70E-61	0.643652214	0.859	0.626	8.84E-57	2	Prdx5
1.62E-60	-1.38882959	0.202	0.573	2.14E-56	2	Psap
2.02E-59	0.676810381	0.788	0.476	2.66E-55	2	Cybb
8.53E-59	-1.244222119	0.118	0.5	1.13E-54	2	Gm8730
5.86E-58	-1.246708956	0.133	0.505	7.73E-54	2	Rps26-ps1
3.86E-57	-0.746915168	0.557	0.813	5.10E-53	2	Ppia
1.23E-56	-1.22379432	0.148	0.514	1.62E-52	2	Rpl12
3.40E-56	-1.26892184	0.094	0.464	4.49E-52	2	Gm9493
4.91E-56	-1.168064177	0.328	0.659	6.47E-52	2	Cst3
5.70E-56	-0.639125904	0.711	0.887	7.52E-52	2	Rps9
7.04E-56	-0.946288888	0.31	0.665	9.29E-52	2	Xist
1.86E-55	-0.767820273	0.603	0.84	2.45E-51	2	Ftl1
2.68E-55	-1.236298101	0.135	0.496	3.53E-51	2	Rpl10-ps3
3.46E-55	-0.973377605	0.293	0.636	4.57E-51	2	Rps21
1.78E-54	-1.193550283	0.119	0.487	2.35E-50	2	Rps12-ps3
1.22E-52	-0.853772568	0.378	0.682	1.61E-48	2	Rps10
6.03E-52	-0.631116835	0.992	0.998	7.95E-48	2	Malat1
1.35E-51	-0.954698809	0.283	0.617	1.79E-47	2	Rpl4
1.57E-50	-1.079518586	0.146	0.499	2.07E-46	2	Rpl29
1.07E-48	-1.123106405	0.119	0.462	1.42E-44	2	Rpl13-ps3
1.13E-48	-0.648116518	0.549	0.812	1.49E-44	2	Gm9843
1.77E-48	-0.935119279	0.258	0.598	2.34E-44	2	mt-Nd4
1.44E-47	-0.70561453	0.495	0.767	1.90E-43	2	Rpl9
8.27E-47	-1.193036669	0.127	0.455	1.09E-42	2	Prdx1
8.46E-47	-0.916015961	0.239	0.572	1.12E-42	2	Hspa8
1.19E-46	0.808027183	0.266	0.078	1.57E-42	2	AA467197
2.44E-45	-1.138326191	0.108	0.429	3.22E-41	2	Rpl36-ps3

6.65E-45	0.887832961	0.391	0.163	8.77E-41	2	Cebpe
1.48E-44	-0.74908227	0.457	0.727	1.95E-40	2	Sub1
1.57E-44	-0.846916031	0.241	0.574	2.07E-40	2	Rpl7
2.37E-44	-0.815577028	0.264	0.601	3.13E-40	2	Rpl36al
3.45E-44	-1.197887611	0.094	0.408	4.55E-40	2	Npm1
4.85E-43	-0.916128318	0.37	0.666	6.40E-39	2	Fth1
9.10E-43	0.285811716	1	0.898	1.20E-38	2	Lyz2
2.62E-42	-1.010613425	0.202	0.523	3.45E-38	2	Tubb5
3.31E-42	0.509959019	0.796	0.563	4.36E-38	2	S100a11
5.51E-42	-1.438610205	0.05	0.341	7.27E-38	2	Ms4a6c
6.25E-42	0.540618178	0.827	0.651	8.24E-38	2	Arhgdib
1.02E-41	-0.920943668	0.177	0.5	1.34E-37	2	Rpl27-ps3
4.17E-41	-0.975408484	0.148	0.464	5.50E-37	2	Gnb2l1
7.11E-40	0.789215341	0.437	0.211	9.38E-36	2	Cpne3
4.56E-39	-0.819004552	0.241	0.544	6.01E-35	2	Snrpg
3.76E-38	-0.96659141	0.148	0.443	4.95E-34	2	Hsp90ab1
3.04E-37	-0.874795472	0.195	0.489	4.01E-33	2	Rpl30
9.23E-37	-1.413485233	0.035	0.295	1.22E-32	2	Ccr2
1.59E-36	-0.858433749	0.15	0.451	2.10E-32	2	Eef2
5.31E-36	-0.903211905	0.127	0.42	7.01E-32	2	mt-Atp8
3.07E-35	-1.455280178	0.112	0.381	4.05E-31	2	Ifi27l2a
4.08E-35	-0.97610932	0.071	0.343	5.38E-31	2	Gm10036
4.80E-35	-0.883084648	0.183	0.472	6.33E-31	2	Psma7
1.94E-34	-1.002847084	0.089	0.358	2.56E-30	2	Nop10
1.99E-34	-0.861315878	0.168	0.454	2.62E-30	2	Rpl5
2.55E-34	-0.99233776	0.092	0.363	3.37E-30	2	Set
2.86E-34	-0.754721404	0.235	0.523	3.77E-30	2	Rpl7a
3.10E-34	-0.916195164	0.096	0.373	4.08E-30	2	Rpl6l
3.57E-34	-0.823831414	0.252	0.527	4.71E-30	2	Rpl22l1
6.16E-34	-1.212851411	0.015	0.255	8.13E-30	2	Zeb2
6.47E-34	0.443161337	0.842	0.67	8.54E-30	2	Cyba

7.49E-34	0.589623782	0.647	0.438	9.88E-30	2	Hp
1.18E-33	0.479349326	0.765	0.572	1.55E-29	2	Hmgn2
2.27E-33	-0.774462249	0.218	0.5	3.00E-29	2	Rps27l
7.29E-33	-1.205804781	0.035	0.277	9.61E-29	2	Gm9844
7.36E-33	-0.613736347	0.414	0.676	9.71E-29	2	mt-Co2
8.07E-33	-0.966201855	0.094	0.356	1.06E-28	2	Hnrnpa1
1.17E-32	-1.018446632	0.056	0.304	1.54E-28	2	Eef1g
1.26E-32	-1.150851695	0.064	0.315	1.66E-28	2	Pdcd4
1.41E-32	-0.844188908	0.173	0.45	1.86E-28	2	Serbp1
1.55E-32	-1.001940216	0.104	0.366	2.05E-28	2	H2afy
2.14E-32	-0.935560546	0.118	0.378	2.82E-28	2	Snrpf
2.79E-32	-0.932708137	0.067	0.325	3.68E-28	2	mt-Nd2
1.25E-31	-0.92683853	0.091	0.346	1.65E-27	2	Hsp90aa1
2.09E-31	-0.776409134	0.258	0.53	2.76E-27	2	Cbx3
7.94E-31	-0.772655629	0.179	0.448	1.05E-26	2	Hint1
8.67E-31	-0.874610914	0.089	0.345	1.14E-26	2	Slc25a5
1.42E-30	-0.863739648	0.083	0.338	1.87E-26	2	Eef1b2
1.80E-30	-0.948433686	0.091	0.342	2.37E-26	2	Npc2
2.27E-29	0.60294341	0.576	0.381	2.99E-25	2	Dstn
5.64E-29	-0.711289877	0.206	0.473	7.43E-25	2	Naca
9.76E-29	0.708031298	0.318	0.145	1.29E-24	2	Cd9
1.72E-28	-0.716641312	0.339	0.598	2.27E-24	2	H2-D1
2.38E-28	-0.859824759	0.087	0.325	3.13E-24	2	Polr2l
2.91E-28	-0.8639115	0.069	0.302	3.84E-24	2	Gm10269
3.13E-28	-1.061307934	0.058	0.282	4.13E-24	2	S100a10
7.01E-28	-0.777346121	0.17	0.424	9.25E-24	2	Dbi
7.42E-28	-0.65928944	0.233	0.493	9.79E-24	2	Ndufa4
1.07E-27	-0.755025994	0.162	0.412	1.41E-23	2	Gm8186
3.38E-27	-0.846385012	0.108	0.344	4.46E-23	2	Ranbp1
8.62E-27	-0.577081213	0.339	0.594	1.14E-22	2	Cox8a
1.39E-26	-0.752398182	0.146	0.387	1.83E-22	2	Tuba1b

1.58E-26	-0.816171014	0.1	0.333	2.09E-22	2	Atp5g1
2.60E-26	-0.822219302	0.114	0.344	3.44E-22	2	Eif3f
2.71E-26	-0.800165172	0.075	0.303	3.57E-22	2	Nsa2
5.55E-26	-0.94817788	0.06	0.272	7.32E-22	2	Ncl
6.84E-26	-0.66641533	0.216	0.468	9.03E-22	2	Rpl9-ps6
2.60E-25	-0.601905681	0.243	0.503	3.44E-21	2	Uqcrh
5.30E-25	-0.746567118	0.096	0.32	7.00E-21	2	Erh
5.46E-25	-0.64988069	0.222	0.471	7.20E-21	2	Hnrnpa3
6.32E-25	-0.539088269	0.617	0.784	8.33E-21	2	B2m
8.30E-25	-0.735602962	0.15	0.387	1.09E-20	2	Dek
2.21E-24	0.6706211	0.337	0.172	2.92E-20	2	Adpgk
2.72E-24	-0.528622973	0.435	0.655	3.59E-20	2	Sec61g
2.95E-24	-0.455563606	0.543	0.759	3.90E-20	2	H3f3b
5.48E-24	-1.065090231	0.185	0.403	7.23E-20	2	Hist1h2ap
6.53E-24	-0.692962633	0.166	0.395	8.62E-20	2	Btf3
1.62E-23	-0.583459114	0.252	0.498	2.14E-19	2	Morf4l1
1.21E-22	-0.570306592	0.231	0.47	1.60E-18	2	Uqcr11
2.07E-22	-0.64658857	0.162	0.384	2.73E-18	2	Ran
3.57E-22	-0.689097166	0.17	0.391	4.72E-18	2	Srrm2
3.90E-22	-0.484761072	0.468	0.683	5.15E-18	2	Cox4i1
6.57E-22	-0.73823953	0.092	0.294	8.66E-18	2	Bola2
7.39E-22	-0.830839819	0.075	0.266	9.75E-18	2	Psmb8
9.51E-22	-0.628313666	0.295	0.522	1.25E-17	2	Mbnl1
9.97E-22	-0.649989513	0.206	0.431	1.32E-17	2	Stmn1
2.01E-21	-0.607418141	0.179	0.403	2.66E-17	2	Eif2s2
2.27E-21	-1.621031497	0.079	0.262	2.99E-17	2	Elane
2.84E-21	-0.606776389	0.231	0.452	3.75E-17	2	Ybx1
3.02E-21	0.648897066	0.353	0.2	3.99E-17	2	Serpinb1a
5.89E-20	-0.529753833	0.306	0.53	7.77E-16	2	Pabpc1
5.97E-20	0.547319255	0.366	0.211	7.87E-16	2	Cd63
6.26E-20	-0.63322978	0.114	0.315	8.26E-16	2	Atp5g2

8.31E-20	-0.771270478	0.077	0.259	1.10E-15	2	Calr
1.44E-19	-0.601221068	0.183	0.393	1.90E-15	2	H2afv
1.48E-19	-0.586216383	0.254	0.468	1.96E-15	2	Atpif1
1.73E-19	-0.445382238	0.408	0.62	2.28E-15	2	Cox7c
1.96E-19	-0.412124148	0.605	0.757	2.58E-15	2	Gpx1
2.75E-19	-0.634054236	0.131	0.33	3.62E-15	2	Eif3k
2.92E-19	-0.585331659	0.104	0.302	3.85E-15	2	Eif4a1
3.55E-19	-0.554804891	0.235	0.449	4.68E-15	2	Serinc3
3.64E-19	-0.664002355	0.17	0.374	4.80E-15	2	H2-K1
3.86E-19	-0.636104321	0.135	0.335	5.09E-15	2	Gm10116
4.04E-19	-0.507515118	0.26	0.493	5.33E-15	2	H2afj
5.91E-19	-0.55851027	0.112	0.309	7.79E-15	2	Nap1l1
1.29E-18	-0.388798743	0.514	0.713	1.70E-14	2	Oaz1
1.31E-18	-0.619392608	0.129	0.32	1.72E-14	2	Tsix
2.01E-18	-0.598830081	0.083	0.262	2.65E-14	2	Eif3h
2.06E-18	-1.288194458	0.114	0.284	2.72E-14	2	Mpo
2.98E-18	-0.433196229	0.355	0.578	3.93E-14	2	Calm2
9.03E-18	-0.551383881	0.15	0.351	1.19E-13	2	Pomp
9.32E-18	-0.547813055	0.133	0.328	1.23E-13	2	Cycs
1.15E-17	-0.525444344	0.154	0.352	1.52E-13	2	Nucks1
1.48E-17	-0.493197652	0.345	0.553	1.96E-13	2	Mrpl33
1.49E-17	-0.556455541	0.129	0.323	1.96E-13	2	Canx
1.80E-17	-0.550861912	0.125	0.316	2.38E-13	2	Sfpq
2.02E-17	-0.480179216	0.193	0.399	2.67E-13	2	Minos1
5.70E-17	0.626445384	0.266	0.143	7.52E-13	2	Glrx
5.85E-17	-0.508174423	0.225	0.424	7.71E-13	2	Anp32b
7.84E-17	-0.585797372	0.102	0.277	1.03E-12	2	Prrc2c
9.38E-17	-1.141775524	0.125	0.286	1.24E-12	2	Prtn3
1.08E-16	-0.429509036	0.389	0.584	1.43E-12	2	Hnrnpa2b1
1.22E-16	-0.537309263	0.1	0.277	1.61E-12	2	Snrpd2
1.43E-16	-0.397805476	0.472	0.665	1.89E-12	2	Atp5l

1.56E-16	-0.523652294	0.173	0.363	2.05E-12	2	Ptpn18
1.96E-16	-0.418190535	0.264	0.477	2.58E-12	2	Ndufa2
1.99E-16	-0.722618861	0.106	0.271	2.63E-12	2	Cebpb
2.21E-16	-0.556748472	0.1	0.274	2.92E-12	2	Atp5g3
2.38E-16	-0.563330667	0.106	0.278	3.13E-12	2	Snrpb
2.44E-16	-0.598307739	0.123	0.299	3.22E-12	2	Tmpo
3.75E-16	-0.565271809	0.118	0.292	4.95E-12	2	mt-Nd4l
3.84E-16	-0.437502723	0.237	0.445	5.07E-12	2	Sumo2
7.67E-16	-0.337064842	0.628	0.788	1.01E-11	2	H3f3a
9.07E-16	-0.489403705	0.15	0.336	1.20E-11	2	Hnrnpu
9.40E-16	0.60222906	0.297	0.171	1.24E-11	2	Ckap4
1.10E-15	-0.544672292	0.102	0.27	1.45E-11	2	Alyref
1.24E-15	-0.539843066	0.1	0.267	1.64E-11	2	Eif3a
1.50E-15	-0.528715323	0.125	0.301	1.98E-11	2	Psmb1
1.59E-15	-0.480241599	0.229	0.427	2.10E-11	2	Atox1
2.08E-15	-0.449658349	0.287	0.484	2.75E-11	2	Cox5b
4.21E-15	-0.433708517	0.256	0.459	5.56E-11	2	Hnrnpf
4.33E-15	-0.574050845	0.171	0.347	5.71E-11	2	Notch2
4.43E-15	-0.551778635	0.1	0.262	5.84E-11	2	Rbm25
4.82E-15	-0.508609102	0.123	0.295	6.36E-11	2	Hnrnpab
7.11E-15	0.252204221	0.825	0.722	9.38E-11	2	Cd52
1.04E-14	0.493296132	0.387	0.255	1.37E-10	2	Clec4a2
1.09E-14	-0.441059145	0.224	0.413	1.44E-10	2	Tomm7
1.33E-14	0.487167447	0.333	0.197	1.75E-10	2	Fcnb
1.45E-14	-0.545933027	0.378	0.546	1.92E-10	2	S100a6
1.56E-14	-0.528735697	0.162	0.332	2.05E-10	2	Top2a
2.10E-14	-0.371176868	0.339	0.541	2.77E-10	2	Tma7
2.80E-14	-0.455765582	0.156	0.332	3.69E-10	2	Nedd8
3.05E-14	-0.412112895	0.195	0.379	4.02E-10	2	Son
3.07E-14	-0.525808916	0.096	0.252	4.05E-10	2	Cks1b
3.27E-14	-0.437406552	0.202	0.389	4.31E-10	2	Tmem256

3.31E-14	-0.430851231	0.218	0.405	4.37E-10	2	Smdt1
3.43E-14	-0.442154593	0.195	0.377	4.52E-10	2	BC005537
3.73E-14	-0.552747926	0.102	0.258	4.91E-10	2	Fxyd5
3.79E-14	-0.553761261	0.1	0.252	5.00E-10	2	Eif5b
5.48E-14	-0.490105108	0.168	0.341	7.23E-10	2	Ndufa7
7.06E-14	-0.460514747	0.125	0.29	9.31E-10	2	Arglu1
7.80E-14	-0.45658286	0.21	0.387	1.03E-09	2	Srsf2
1.06E-13	-0.458859557	0.131	0.296	1.40E-09	2	Tra2b
1.33E-13	-0.522449902	0.121	0.276	1.76E-09	2	Gnas
1.39E-13	-0.452311192	0.162	0.33	1.84E-09	2	Snrpe
2.01E-13	0.499964357	0.324	0.201	2.65E-09	2	Mcemp1
2.14E-13	-0.471646703	0.135	0.297	2.83E-09	2	Cmtm7
2.74E-13	-0.460704015	0.183	0.355	3.61E-09	2	Hsp90b1
2.88E-13	-0.484597517	0.129	0.285	3.80E-09	2	Ywhae
3.46E-13	-0.39912053	0.233	0.419	4.57E-09	2	Pfdn5
4.35E-13	-0.356794813	0.349	0.535	5.73E-09	2	Atp5j2
5.50E-13	-0.39945098	0.198	0.372	7.25E-09	2	Arl6ip1
5.78E-13	-0.45101488	0.154	0.318	7.63E-09	2	Hnrnpm
9.66E-13	-0.355262997	0.368	0.548	1.27E-08	2	Atp5k
1.01E-12	0.388771822	0.568	0.47	1.34E-08	2	F630028O10Rik
1.08E-12	-0.370896497	0.241	0.42	1.43E-08	2	Uqcrq
1.65E-12	-0.395964655	0.341	0.522	2.17E-08	2	Sec61b
1.77E-12	-0.437153557	0.141	0.296	2.34E-08	2	Luc7l2
3.05E-12	-0.426603871	0.143	0.299	4.02E-08	2	Clta
3.24E-12	-0.436771285	0.129	0.28	4.27E-08	2	Ndufb9
5.28E-12	-0.408571504	0.27	0.441	6.96E-08	2	Rbm3
7.32E-12	-0.493410991	0.127	0.27	9.66E-08	2	Ucp2
7.63E-12	-0.40176871	0.229	0.391	1.01E-07	2	Eif5a
1.05E-11	-0.335560485	0.372	0.557	1.38E-07	2	Ubb
1.41E-11	-0.395784585	0.135	0.285	1.86E-07	2	Atp5o
1.92E-11	-0.392641752	0.168	0.324	2.53E-07	2	Arf5

2.23E-11	-0.356526012	0.254	0.426	2.95E-07	2	2010107E04Rik
2.57E-11	-0.456444662	0.522	0.623	3.39E-07	2	Ly6c2
2.64E-11	-0.331309884	0.395	0.564	3.48E-07	2	Cox6b1
3.05E-11	-0.418328834	0.125	0.267	4.02E-07	2	Atp5c1
3.08E-11	-0.304947428	0.487	0.636	4.07E-07	2	Cox6c
3.19E-11	-0.502394581	0.119	0.253	4.20E-07	2	Zfp36l2
3.78E-11	-0.354315071	0.145	0.294	4.98E-07	2	Atp5b
4.08E-11	-0.381716246	0.118	0.258	5.38E-07	2	Hnrnph1
4.88E-11	-0.374459751	0.145	0.292	6.44E-07	2	Anapc13
4.92E-11	-0.517064766	0.131	0.265	6.49E-07	2	Btg1
8.88E-11	-0.342644241	0.158	0.312	1.17E-06	2	Ost4
9.14E-11	-0.314365464	0.356	0.528	1.21E-06	2	Cox6a1
9.73E-11	-0.396932255	0.158	0.303	1.28E-06	2	Rsrp1
1.33E-10	-0.358330832	0.127	0.267	1.75E-06	2	Ndufb8
1.49E-10	-0.361330484	0.2	0.35	1.97E-06	2	Srsf3
1.59E-10	0.40801869	0.503	0.417	2.10E-06	2	Tkt
3.23E-10	-0.335405335	0.206	0.361	4.26E-06	2	Cox7b
3.30E-10	-0.404471561	0.229	0.377	4.36E-06	2	Emp3
4.33E-10	-0.345504285	0.241	0.398	5.71E-06	2	Tmem258
5.80E-10	-0.398379054	0.15	0.288	7.65E-06	2	Reep5
6.58E-10	-0.33024243	0.195	0.344	8.68E-06	2	Lsm5
8.11E-10	-0.344909011	0.171	0.319	1.07E-05	2	Pcbp1
1.12E-09	-0.359399952	0.164	0.302	1.47E-05	2	D8Ertd738e
1.15E-09	-0.340690785	0.135	0.268	1.52E-05	2	Lsm6
1.27E-09	0.340896508	0.391	0.288	1.68E-05	2	Cd24a
1.48E-09	-0.395338071	0.204	0.345	1.95E-05	2	2810417H13Rik
1.64E-09	-0.346925743	0.164	0.299	2.16E-05	2	Sdcbp
1.78E-09	-0.364914305	0.168	0.305	2.34E-05	2	Laptm5
2.34E-09	-0.308234808	0.308	0.467	3.08E-05	2	Uqcr10
2.50E-09	-0.69865753	0.47	0.567	3.30E-05	2	Ifitm3
3.83E-09	-0.37071691	0.229	0.375	5.05E-05	2	Serp1

4.59E-09	-0.250529055	0.434	0.599	6.06E-05	2	Gm26917
5.79E-09	-0.314604269	0.145	0.273	7.64E-05	2	Tmem167
7.52E-09	-0.329008052	0.137	0.262	9.93E-05	2	Ndufb2
8.69E-09	-0.280369617	0.472	0.614	0.000114641	2	Atp5e
9.01E-09	-0.30460621	0.152	0.281	0.000118804	2	Romo1
9.06E-09	-0.263941588	0.308	0.471	0.000119555	2	Dynll1
9.10E-09	0.426538846	0.347	0.256	0.000120015	2	Ncf1
9.18E-09	-0.300851479	0.173	0.312	0.000121046	2	Sumo1
1.01E-08	-0.355257268	0.216	0.347	0.00013262	2	Clic1
1.48E-08	-0.285721572	0.197	0.334	0.000194661	2	Celf2
1.70E-08	-0.272068546	0.173	0.309	0.00022468	2	Ndufc1
1.82E-08	-0.389673872	0.177	0.304	0.00024	2	Sat1
2.21E-08	-0.406155664	0.139	0.255	0.000291019	2	Pgls
2.22E-08	-0.28129489	0.137	0.263	0.000292852	2	Timm13
2.26E-08	0.381743592	0.299	0.206	0.000297833	2	C3
2.94E-08	-0.352592325	0.775	0.819	0.000387824	2	Tyrobp
3.17E-08	-0.496034583	0.339	0.447	0.000418723	2	Lgals3
3.47E-08	-0.324037787	0.141	0.259	0.000458364	2	Capzb
4.60E-08	-0.288773376	0.143	0.261	0.000607022	2	Ndufb3
5.74E-08	-0.264741026	0.283	0.44	0.000757387	2	Hnrnpk
5.82E-08	0.38619446	0.397	0.317	0.000767619	2	Scp2
5.99E-08	-0.298841354	0.156	0.279	0.00079066	2	Tmed2
6.46E-08	-0.256592501	0.362	0.506	0.000852064	2	Pcbp2
8.39E-08	-0.310753334	0.166	0.288	0.001107128	2	Atp6v1g1
9.17E-08	0.381399984	0.335	0.251	0.001210074	2	Lmo4
1.19E-07	-0.279076116	0.175	0.297	0.001565129	2	Tagln2
1.26E-07	-0.338905735	0.152	0.265	0.001657727	2	Wtap
1.27E-07	-0.299812944	0.168	0.286	0.001681294	2	Rac1
1.54E-07	0.37721266	0.351	0.268	0.002036439	2	Aldh2
1.92E-07	-0.262005689	0.303	0.447	0.002532879	2	Rhoa
1.95E-07	-0.286163459	0.141	0.253	0.002578717	2	Scand1

2.15E-07	-0.282869309	0.347	0.481	0.002839182	2	Tceb2	
3.01E-07	-0.252730113	0.281	0.419	0.003973845	2	Rbm39	
3.70E-07	-0.354088468	0.21	0.326	0.00488233	2	Lsp1	
4.22E-07	-0.272664372	0.231	0.36	0.005566864	2	Ndufa6	
4.39E-07	-0.262061968	0.291	0.423	0.005792959	2	Chchd2	
4.86E-07	-0.278648521	0.241	0.37	0.006411085	2	Gpx4	
7.37E-07	-0.268611145	0.25	0.378	0.009716223	2	Capza2	
9.04E-07	-0.256061495	0.434	0.566	0.011920664	2	Calm1	
3.43E-06	-0.293953124	0.162	0.264	0.045199135	2	Vim	
3.89E-06	0.449988981	0.326	0.264	0.051263355	2	Gpi1	
4.02E-06	0.262927411	0.497	0.44	0.053081321	2	Gmfg	
3.06E-05	0.263029604	0.509	0.46	0.404085936	2	Rac2	
7.99E-05	0.284912494	0.345	0.286	1	2	Mgst1	
0.00016437	-0.29123952	0.237	0.323	1	2	Ptprc	
0.00110372	0.250066641	0.329	0.29	1	2	Ppp1cb	
0.00294072	0.264242895	0.295	0.255	1	2	Gm10282	
8.90E-209	1.272194119	0.996	0.606	1.17E-204	3	Crip1	**Mono**
1.88E-206	1.21578877	0.998	0.717	2.48E-202	3	Tmsb10	
3.62E-205	1.343516778	0.733	0.165	4.77E-201	3	Fn1	
8.41E-189	1.266929418	0.728	0.177	1.11E-184	3	F13a1	
3.21E-165	1.134835622	0.759	0.214	4.23E-161	3	Gm9844	
4.42E-152	0.985677048	0.714	0.184	5.83E-148	3	S100a4	
2.79E-145	1.131271394	0.724	0.224	3.69E-141	3	S100a10	
5.40E-141	0.980534266	0.802	0.275	7.13E-137	3	Ms4a6c	
8.66E-138	0.977649283	0.897	0.399	1.14E-133	3	Lgals3	
7.71E-124	0.965902355	0.379	0.064	1.02E-119	3	Klf4	
1.05E-120	0.808468657	0.965	0.495	1.38E-116	3	S100a6	
5.11E-111	0.816140997	0.716	0.236	6.75E-107	3	Ccr2	
6.89E-94	0.754651824	0.881	0.443	9.09E-90	3	Lgals1	
3.98E-93	0.720956578	0.93	0.587	5.25E-89	3	Ly6c2	
4.51E-92	0.884134568	0.412	0.098	5.94E-88	3	Ifi30	

4.89E-91	0.680412455	0.914	0.511	6.45E-87	3	Psap
1.63E-89	0.645704488	0.942	0.605	2.15E-85	3	Cst3
4.70E-88	0.9354591	0.346	0.076	6.20E-84	3	C1galt1c1
8.52E-88	0.625854057	0.926	0.527	1.12E-83	3	Ifitm3
1.47E-87	0.857579095	0.802	0.396	1.94E-83	3	Prdx1
1.59E-77	0.762035049	0.554	0.183	2.10E-73	3	Ahnak
7.55E-72	0.520524757	0.981	0.807	9.96E-68	3	Ftl1
8.36E-69	0.725747471	0.78	0.42	1.10E-64	3	Psma7
1.96E-68	0.790044478	0.58	0.229	2.59E-64	3	Cebpb
1.09E-65	0.707926423	0.362	0.1	1.44E-61	3	Tpm4
3.16E-64	0.604620742	0.922	0.767	4.16E-60	3	Hmgb2
4.37E-64	0.778920563	0.397	0.122	5.77E-60	3	Anxa5
2.87E-63	0.722867743	0.372	0.107	3.79E-59	3	Ctsc
3.05E-62	0.702524394	0.704	0.329	4.02E-58	3	Ifi27l2a
7.01E-62	0.674567415	0.735	0.374	9.25E-58	3	Dbi
1.02E-61	0.73148856	0.566	0.229	1.34E-57	3	Vim
7.02E-61	0.597694834	0.449	0.145	9.26E-57	3	Pld4
1.31E-60	0.501162852	0.957	0.808	1.73E-56	3	H2afz
8.52E-60	0.759277507	0.471	0.172	1.12E-55	3	Emb
1.27E-58	-2.159276478	0.994	0.994	1.67E-54	3	S100a9
1.37E-58	0.584089366	0.802	0.44	1.81E-54	3	Ly6e
3.91E-57	-2.053864532	1	0.999	5.16E-53	3	S100a8
2.97E-56	-2.036224071	0.418	0.648	3.92E-52	3	Wfdc21
3.06E-56	0.575084515	0.313	0.083	4.03E-52	3	Ms4a4c
3.96E-56	0.488079148	0.949	0.779	5.23E-52	3	Ppia
2.70E-54	-2.121989207	0.922	0.932	3.56E-50	3	Camp
2.48E-53	0.646583271	0.387	0.129	3.27E-49	3	Cd48
1.69E-52	-1.746209111	0.156	0.473	2.23E-48	3	Pglyrp1
2.15E-52	0.412349773	0.996	0.898	2.83E-48	3	Lyz2
5.68E-51	0.703945139	0.56	0.261	7.49E-47	3	Tmpo
2.35E-48	-2.164141127	0.792	0.854	3.10E-44	3	Ngp

2.05E-47	0.657636377	0.298	0.088	2.70E-43	3	Rassf4
4.89E-47	0.664540331	0.634	0.344	6.46E-43	3	Tuba1b
2.04E-46	-1.904173032	0.321	0.56	2.69E-42	3	Ltf
9.65E-45	0.424108214	0.942	0.76	1.27E-40	3	H3f3a
1.41E-44	0.641891509	0.366	0.129	1.86E-40	3	Capg
2.26E-43	0.285366554	1	0.995	2.98E-39	3	Tmsb4x
4.05E-43	-1.929418682	0.37	0.574	5.35E-39	3	Lcn2
1.15E-42	0.566939606	0.496	0.217	1.51E-38	3	Pycard
1.22E-42	0.608070138	0.453	0.189	1.61E-38	3	Lamp1
1.07E-41	0.406740981	0.926	0.687	1.41E-37	3	Sub1
1.70E-41	0.430778435	0.891	0.673	2.25E-37	3	Hmgb1
2.64E-41	0.657283408	0.405	0.165	3.48E-37	3	Fam96a
5.96E-41	0.576003138	0.514	0.234	7.86E-37	3	Anxa2
4.31E-40	0.341200217	0.967	0.802	5.69E-36	3	Tyrobp
6.39E-40	0.55214556	0.482	0.215	8.43E-36	3	Zeb2
1.69E-39	0.417465042	0.928	0.729	2.23E-35	3	Gpx1
3.39E-39	0.470204927	0.885	0.669	4.48E-35	3	Actg1
1.60E-38	0.556548751	0.508	0.237	2.11E-34	3	Ucp2
2.47E-38	0.54617089	0.681	0.404	3.26E-34	3	Hint1
1.30E-37	0.527092052	0.634	0.342	1.72E-33	3	Emp3
1.90E-37	0.355049814	0.973	0.831	2.51E-33	3	Ptma
2.18E-37	0.438705682	0.409	0.163	2.88E-33	3	Ccl6
7.77E-37	0.538577501	0.772	0.515	1.03E-32	3	Mrpl33
8.82E-37	0.648657917	0.348	0.138	1.16E-32	3	Cstb
1.98E-36	0.457595247	0.593	0.299	2.61E-32	3	Npc2
4.23E-36	0.474063266	0.796	0.535	5.58E-32	3	Calm1
4.54E-36	0.706549714	0.28	0.1	5.99E-32	3	Kpna2
5.37E-36	0.531291889	0.296	0.103	7.08E-32	3	Ap1s2
1.00E-35	0.570005774	0.615	0.355	1.32E-31	3	H2afv
1.29E-35	0.543611431	0.274	0.094	1.71E-31	3	Tifab
1.37E-34	0.562284114	0.508	0.257	1.81E-30	3	Reep5

4.30E-34	0.57402988	0.541	0.299	5.67E-30	3	Top2a
1.62E-33	0.694397112	0.358	0.157	2.14E-29	3	Cenpa
1.98E-33	0.351285518	0.907	0.698	2.61E-29	3	Fcer1g
2.75E-32	0.492116846	0.681	0.405	3.63E-28	3	Rbm3
4.05E-32	0.553196664	0.325	0.132	5.35E-28	3	Sod1
6.36E-32	0.528421679	0.57	0.317	8.39E-28	3	Clic1
2.41E-30	0.444457123	0.422	0.193	3.18E-26	3	Grn
2.54E-30	0.447982476	0.774	0.525	3.35E-26	3	Hspa8
3.57E-30	0.450731901	0.403	0.178	4.71E-26	3	Mpeg1
8.22E-30	0.548870244	0.551	0.315	1.08E-25	3	2810417H13Rik
4.23E-29	0.471740402	0.35	0.15	5.58E-25	3	Napsa
5.56E-29	0.512710793	0.687	0.481	7.33E-25	3	Tubb5
2.52E-28	0.452086299	0.547	0.299	3.33E-24	3	Gm10116
3.49E-28	0.455825037	0.34	0.145	4.60E-24	3	Prkcd
4.04E-28	0.450912656	0.325	0.134	5.33E-24	3	Tpd52
5.77E-28	0.289420491	0.422	0.188	7.61E-24	3	Ctss
1.31E-27	0.512471252	0.64	0.414	1.73E-23	3	Tspo
1.86E-26	0.379098104	0.739	0.509	2.45E-22	3	Coro1a
2.34E-26	0.531660344	0.401	0.201	3.08E-22	3	Mki67
2.67E-26	0.461696911	0.545	0.318	3.52E-22	3	Nucks1
1.06E-25	0.468765092	0.267	0.106	1.39E-21	3	Soat1
1.07E-25	0.376134916	0.551	0.296	1.41E-21	3	Lsp1
1.44E-25	0.580014792	0.549	0.342	1.90E-21	3	Arl6ip1
1.80E-25	0.433925905	0.506	0.276	2.37E-21	3	Sat1
5.41E-25	0.488955013	0.358	0.17	7.14E-21	3	Rbms1
6.30E-25	0.454982991	0.399	0.2	8.31E-21	3	Erp29
7.55E-25	0.488123868	0.27	0.11	9.96E-21	3	Ccl9
1.64E-24	0.418032985	0.689	0.449	2.16E-20	3	Cox5b
3.92E-24	0.478378958	0.409	0.215	5.17E-20	3	Hn1
6.46E-24	0.428192907	0.597	0.372	8.52E-20	3	Smdt1
2.50E-23	0.684866887	0.543	0.371	3.30E-19	3	Hist1h2ap

2.64E-23	0.431889138	0.304	0.135	3.49E-19	3	Pitpna
3.27E-23	0.448993419	0.309	0.144	4.31E-19	3	Lmnb1
4.77E-23	0.435420758	0.469	0.26	6.29E-19	3	Rac1
6.46E-23	0.451876332	0.317	0.15	8.53E-19	3	H2afx
8.00E-23	0.36712005	0.409	0.204	1.06E-18	3	Gm2a
8.59E-23	0.539264754	0.329	0.16	1.13E-18	3	Creg1
1.24E-22	0.429773665	0.35	0.168	1.63E-18	3	Kctd12
1.58E-22	0.504503163	0.368	0.192	2.09E-18	3	Birc5
1.91E-22	0.379604859	0.432	0.223	2.52E-18	3	Slfn2
2.52E-22	0.480277709	0.27	0.12	3.32E-18	3	Hist1h2ae
3.55E-22	0.326524473	0.86	0.678	4.68E-18	3	Sh3bgrl3
3.80E-22	0.372915269	0.704	0.471	5.01E-18	3	Arpc2
4.06E-22	0.396073177	0.743	0.544	5.36E-18	3	Calm2
4.59E-22	0.32168532	0.854	0.631	6.06E-18	3	Atp5l
9.96E-22	0.49550574	0.407	0.225	1.31E-17	3	Cks1b
4.39E-21	0.420421668	0.527	0.318	5.79E-17	3	Pomp
5.66E-21	0.447641557	0.407	0.226	7.46E-17	3	Ube2s
8.14E-21	0.423447002	0.521	0.317	1.07E-16	3	Notch2
9.33E-21	0.364888468	0.741	0.501	1.23E-16	3	Atp5j2
1.10E-20	0.365132116	0.422	0.225	1.45E-16	3	Msn
1.40E-20	0.30752346	0.877	0.701	1.84E-16	3	Cfl1
3.20E-20	0.335877786	0.57	0.346	4.22E-16	3	Lst1
3.56E-20	0.401042013	0.508	0.308	4.69E-16	3	Slc25a5
4.75E-20	0.347641127	0.395	0.205	6.27E-16	3	Selplg
5.45E-20	0.438881775	0.298	0.143	7.19E-16	3	Akr1a1
5.62E-20	0.404733788	0.393	0.211	7.41E-16	3	Fam111a
6.10E-20	0.502639446	0.302	0.149	8.05E-16	3	Nusap1
6.24E-20	0.400564421	0.56	0.345	8.24E-16	3	BC005537
7.12E-20	0.358445017	0.605	0.396	9.39E-16	3	Stmn1
8.37E-20	0.274686535	0.868	0.695	1.10E-15	3	Myl6
1.22E-19	0.53504088	0.292	0.144	1.61E-15	3	Ube2c

1.70E-19	0.437487153	0.36	0.19	2.24E-15	3	Arhgdia
1.90E-19	0.358214536	0.568	0.35	2.51E-15	3	Capza2
2.12E-19	0.445708948	0.251	0.111	2.79E-15	3	Smpdl3a
2.16E-19	0.403862244	0.362	0.191	2.85E-15	3	Cmpk1
3.24E-19	0.410703597	0.333	0.173	4.27E-15	3	Gm11273
7.02E-19	0.377062527	0.551	0.352	9.26E-15	3	Dek
7.26E-19	0.280532543	0.877	0.693	9.58E-15	3	Tpt1
9.74E-19	0.393104997	0.315	0.158	1.28E-14	3	Ethe1
1.02E-18	0.369825979	0.422	0.234	1.34E-14	3	Capzb
1.30E-18	0.349259788	0.669	0.462	1.72E-14	3	Morf4l1
1.31E-18	0.349471561	0.648	0.427	1.73E-14	3	Arpc1b
1.37E-18	0.414756106	0.502	0.317	1.81E-14	3	Lsm5
1.86E-18	0.410249184	0.457	0.272	2.46E-14	3	Tagln2
2.04E-18	0.372340491	0.543	0.339	2.69E-14	3	Fam49b
4.77E-18	0.335505155	0.72	0.518	6.30E-14	3	Atp5k
4.77E-18	0.332029786	0.607	0.401	6.30E-14	3	Cdc42
5.46E-18	0.410103027	0.323	0.17	7.21E-14	3	Tubb4b
5.55E-18	0.353360857	0.593	0.381	7.32E-14	3	Actr3
1.30E-17	0.408367001	0.342	0.181	1.72E-13	3	Klf13
1.31E-17	0.327280673	0.603	0.395	1.73E-13	3	Atox1
1.98E-17	0.287952939	0.84	0.65	2.61E-13	3	Cox4i1
1.49E-16	0.366555903	0.329	0.177	1.97E-12	3	Psmb2
1.55E-16	0.306987677	0.685	0.459	2.05E-12	3	Rps27l
2.44E-16	0.311923076	0.422	0.241	3.22E-12	3	Flna
2.52E-16	0.377113018	0.449	0.28	3.33E-12	3	Nap1l1
3.78E-16	0.363323911	0.547	0.359	4.98E-12	3	Tmem256
4.08E-16	0.334470665	0.255	0.121	5.39E-12	3	Ifi203
4.53E-16	0.377202591	0.27	0.135	5.97E-12	3	App
6.58E-16	0.333384689	0.58	0.393	8.68E-12	3	Anp32b
8.63E-16	0.377957663	0.282	0.146	1.14E-11	3	Sdhb
1.51E-15	0.373599024	0.416	0.249	1.99E-11	3	Tmem167

1.88E-15	0.326611702	0.685	0.507	2.48E-11	3	Atp5h
2.36E-15	0.357839757	0.292	0.151	3.11E-11	3	Ctsz
3.04E-15	0.384702858	0.399	0.238	4.01E-11	3	Ndufb3
4.82E-15	0.344835413	0.531	0.351	6.35E-11	3	Ran
5.77E-15	0.270562961	0.405	0.231	7.62E-11	3	Fxyd5
6.17E-15	-0.829198272	0.319	0.439	8.13E-11	3	Anxa1
6.96E-15	0.312635489	0.712	0.537	9.17E-11	3	Cox6b1
6.99E-15	0.292982879	0.428	0.252	9.22E-11	3	Cotl1
8.98E-15	0.283049294	0.745	0.553	1.18E-10	3	Hnrnpa2b1
1.31E-14	0.359291486	0.44	0.274	1.73E-10	3	Clta
2.18E-14	0.319876044	0.374	0.217	2.88E-10	3	Rrbp1
2.28E-14	0.274592391	0.669	0.474	3.00E-10	3	Gnai2
4.08E-14	0.332443858	0.403	0.243	5.38E-10	3	Atp5c1
4.33E-14	0.314696326	0.609	0.444	5.71E-10	3	Dynll1
4.35E-14	0.413391228	0.342	0.203	5.74E-10	3	Rad21
9.81E-14	0.308409773	0.368	0.218	1.29E-09	3	Sptssa
1.25E-13	0.335142358	0.389	0.238	1.65E-09	3	Psmb3
1.27E-13	0.306253533	0.282	0.151	1.68E-09	3	Gm8797
1.31E-13	0.304770716	0.251	0.127	1.73E-09	3	Stx7
1.45E-13	0.307811295	0.259	0.134	1.92E-09	3	Bri3
1.83E-13	0.337492355	0.364	0.218	2.41E-09	3	Csnk1a1
2.17E-13	0.310161098	0.267	0.142	2.86E-09	3	Dock10
2.27E-13	0.279609093	0.687	0.499	2.99E-09	3	Cox6a1
2.73E-13	0.298152904	0.617	0.436	3.60E-09	3	Hnrnpa3
3.06E-13	0.316508251	0.251	0.13	4.03E-09	3	Esd
4.01E-13	0.321547242	0.671	0.531	5.30E-09	3	Ubb
6.41E-13	0.336003958	0.327	0.19	8.45E-09	3	Ndufb5
6.60E-13	0.318424941	0.516	0.351	8.71E-09	3	Arpc3
6.67E-13	0.374904243	0.307	0.179	8.80E-09	3	2700094K13Rik
7.17E-13	0.294456131	0.426	0.265	9.46E-09	3	Iqgap1
7.76E-13	0.2818405	0.461	0.293	1.02E-08	3	Polr2l

7.98E-13	0.328493544	0.261	0.141	1.05E-08	3	Uqcrfs1
8.42E-13	0.267671065	0.743	0.559	1.11E-08	3	Rpl36al
9.51E-13	0.300004591	0.385	0.234	1.25E-08	3	S100a13
9.77E-13	0.270653987	0.589	0.418	1.29E-08	3	Serinc3
1.01E-12	0.254604346	0.79	0.61	1.33E-08	3	Cox6c
1.47E-12	0.276383257	0.636	0.456	1.93E-08	3	Tceb2
1.70E-12	0.326657217	0.321	0.188	2.24E-08	3	Tmco1
2.06E-12	0.25388427	0.512	0.34	2.72E-08	3	Atp6v0e
2.44E-12	0.266975005	0.385	0.237	3.22E-08	3	Sec11c
2.82E-12	-0.72736048	0.179	0.306	3.72E-08	3	Cd24a
3.04E-12	-0.403118197	1	0.997	4.01E-08	3	Malat1
3.72E-12	0.320890004	0.409	0.261	4.91E-08	3	Atp5o
4.72E-12	0.250401907	0.718	0.545	6.22E-08	3	Ndufa3
6.40E-12	0.281238034	0.278	0.156	8.45E-08	3	Ube2l3
6.83E-12	0.256412155	0.366	0.218	9.01E-08	3	Sell
1.14E-11	0.268329674	0.564	0.391	1.51E-07	3	Ndufa13
1.29E-11	0.357644409	0.346	0.22	1.70E-07	3	Anp32e
1.42E-11	0.300667693	0.276	0.156	1.87E-07	3	Sh3bgrl
2.73E-11	0.269555812	0.352	0.213	3.60E-07	3	Psme2
2.89E-11	-0.666880968	0.496	0.557	3.81E-07	3	Chil3
2.99E-11	0.299950967	0.348	0.214	3.95E-07	3	Ptpn6
3.32E-11	0.280558951	0.498	0.336	4.38E-07	3	Cox7b
4.96E-11	0.277763761	0.348	0.216	6.54E-07	3	Cxcr4
6.54E-11	0.282321977	0.337	0.207	8.63E-07	3	Tmed10
7.79E-11	0.273156509	0.449	0.301	1.03E-06	3	Cycs
1.24E-10	0.286420726	0.259	0.148	1.64E-06	3	Brk1
2.16E-10	0.264633349	0.539	0.379	2.85E-06	3	Gm8186
2.32E-10	0.293069173	0.411	0.276	3.06E-06	3	Psmb1
2.34E-10	0.260756263	0.477	0.326	3.09E-06	3	Srsf3
2.45E-10	0.314056745	0.313	0.191	3.23E-06	3	Ccdc12
3.00E-10	0.312053182	0.302	0.185	3.96E-06	3	Chmp2a

3.70E-10	0.29292324	0.412	0.275	4.89E-06	3	Eif4a1
4.36E-10	0.276620167	0.327	0.202	5.75E-06	3	Lamp2
6.21E-10	0.292186268	0.315	0.199	8.19E-06	3	Hnrnpd
9.64E-10	0.262185082	0.424	0.29	1.27E-05	3	Sumo1
1.08E-09	0.291692901	0.331	0.214	1.43E-05	3	Snrpd1
1.11E-09	0.267233423	0.463	0.324	1.47E-05	3	Cox5a
1.14E-09	0.257698899	0.374	0.244	1.50E-05	3	Tln1
1.56E-09	0.274045717	0.545	0.394	2.06E-05	3	Uqcrq
1.81E-09	0.266344967	0.37	0.246	2.39E-05	3	Ndufb8
2.16E-09	0.284060687	0.319	0.202	2.85E-05	3	Psmb6
2.81E-09	0.289017278	0.333	0.215	3.71E-05	3	Frg1
2.87E-09	0.278567053	0.325	0.211	3.78E-05	3	Gm10250
3.46E-09	0.292932525	0.263	0.16	4.57E-05	3	Higd1a
3.98E-09	0.255918586	0.3	0.187	5.25E-05	3	Eno1
4.33E-09	0.266585492	0.311	0.198	5.71E-05	3	Ppib
4.55E-09	0.260700815	0.356	0.234	6.00E-05	3	Atp5f1
4.99E-09	0.261270276	0.389	0.262	6.58E-05	3	Gabarap
5.22E-09	0.268778658	0.296	0.188	6.89E-05	3	Smc4
5.26E-09	0.263079273	0.368	0.247	6.93E-05	3	Alyref
7.28E-09	0.257328612	0.333	0.22	9.61E-05	3	Smc2
7.51E-09	0.259957241	0.265	0.16	9.91E-05	3	Gdi2
7.73E-09	0.256627723	0.327	0.212	0.000101947	3	Sf3b6
1.24E-08	-0.805226608	0.29	0.366	0.000163455	3	Ifitm6
1.27E-08	0.255589185	0.375	0.255	0.000167744	3	Snrpb
1.61E-08	0.281332707	0.298	0.194	0.000212267	3	Ndufb4
1.81E-08	0.255928034	0.323	0.214	0.000239182	3	Ndufc2
2.52E-08	0.257261543	0.44	0.316	0.000332877	3	Cks2
2.66E-08	0.260564982	0.364	0.245	0.000350478	3	Ndufs5
6.47E-08	0.278440134	0.34	0.237	0.000852865	3	Srp9
1.25E-07	0.282015795	0.261	0.169	0.001653733	3	Mrpl23
1.35E-07	0.251134087	0.253	0.162	0.001775013	3	Znhit1

6.39E-07	0.256831201	0.35	0.251	0.00843065	3	Aprt	
3.37E-06	-0.327016739	0.401	0.472	0.044487918	3	Cd47	
1.83E-05	-0.519646744	0.628	0.584	0.241616377	3	Hmgn2	
3.73E-05	-0.999671279	0.222	0.274	0.491648179	3	Mpo	
4.52E-05	-0.961078991	0.44	0.48	0.596561762	3	Retnlg	
8.93E-05	-0.421638665	0.206	0.268	1	3	Ncf1	
0.00013995	-0.504398112	0.243	0.299	1	3	Pdcd4	
0.00034842	-0.382624585	0.204	0.262	1	3	Gm10282	
0.00098323	-0.424717838	0.214	0.259	1	3	Ncl	
0.00154921	-0.299506903	0.393	0.426	1	3	Tkt	
0.00864545	-0.275943882	0.304	0.335	1	3	Clec12a	
2.48E-216	1.72733359	0.739	0.18	3.27E-212	4	Wfdc17	**Mono**
1.57E-204	1.398504881	0.971	0.525	2.07E-200	4	Ifitm3	
2.41E-200	1.141840852	0.998	0.898	3.17E-196	4	Lyz2	
1.23E-150	1.380016697	0.661	0.191	1.62E-146	4	S100a4	
7.48E-150	1.51380445	0.586	0.15	9.86E-146	4	Ccl6	
2.01E-149	1.386322797	0.71	0.238	2.66E-145	4	Ccr2	
1.97E-148	1.031500168	0.994	0.719	2.60E-144	4	Tmsb10	
1.49E-146	1.418049295	0.627	0.176	1.97E-142	4	Fn1	
8.62E-142	1.087851478	0.922	0.5	1.14E-137	4	S100a6	
1.17E-138	1.012781813	0.976	0.609	1.55E-134	4	Crip1	
7.99E-135	1.280393472	0.784	0.324	1.05E-130	4	Ifi27l2a	
8.55E-119	1.131144947	0.814	0.408	1.13E-114	4	Lgals3	
4.76E-110	1.241425564	0.565	0.177	6.28E-106	4	Ctss	
4.91E-108	0.782338955	0.967	0.809	6.48E-104	4	Ftl1	
2.24E-101	0.910625544	0.876	0.516	2.95E-97	4	Psap	
3.00E-91	1.135219125	0.549	0.194	3.96E-87	4	F13a1	
2.31E-89	1.040144354	0.667	0.288	3.05E-85	4	Ms4a6c	
6.90E-88	0.799897511	0.88	0.612	9.10E-84	4	Cst3	
9.35E-81	0.683424444	0.945	0.804	1.23E-76	4	Tyrobp	
1.91E-80	-1.6008558	0.157	0.623	2.51E-76	4	Hmgn2	

1.02E-72	1.071967348	0.504	0.188	1.35E-68	4	Ahnak
1.42E-72	0.700651905	0.892	0.7	1.87E-68	4	Fcer1g
3.85E-69	0.372706565	1	0.995	5.08E-65	4	Tmsb4x
5.32E-64	1.141822386	0.28	0.068	7.02E-60	4	Cx3cr1
3.12E-63	0.980502584	0.537	0.234	4.12E-59	4	Gm9844
2.41E-62	-2.03121254	0.21	0.587	3.18E-58	4	Lcn2
1.78E-60	-1.974075458	0.312	0.656	2.35E-56	4	Wfdc21
2.73E-58	1.059710984	0.304	0.085	3.60E-54	4	Ms4a4c
5.68E-53	1.046826207	0.327	0.106	7.50E-49	4	Ccl9
6.85E-53	1.052218898	0.473	0.216	9.03E-49	4	Zeb2
3.92E-51	1.01205845	0.294	0.089	5.17E-47	4	Ms4a6b
6.76E-51	-2.188775019	0.659	0.865	8.92E-47	4	Ngp
8.82E-49	0.567879307	0.871	0.719	1.16E-44	4	Cd52
2.70E-48	0.524361959	0.884	0.761	3.56E-44	4	B2m
2.79E-47	-1.724441963	0.251	0.565	3.68E-43	4	Ltf
3.96E-47	-1.444538167	0.139	0.473	5.22E-43	4	Pglyrp1
6.34E-47	0.827826932	0.59	0.345	8.36E-43	4	Lst1
6.17E-45	-2.129181351	0.986	0.995	8.14E-41	4	S100a9
7.07E-44	0.510136693	0.863	0.768	9.33E-40	4	H3f3a
1.10E-43	0.748048627	0.637	0.411	1.45E-39	4	Prdx1
9.36E-42	-0.67032588	0.747	0.85	1.23E-37	4	Ptma
1.08E-41	0.406780376	0.973	0.915	1.43E-37	4	Fau
7.97E-41	-2.050522735	0.998	0.999	1.05E-36	4	S100a8
1.02E-40	0.633345373	0.737	0.564	1.35E-36	4	H2-D1
5.31E-40	-2.023914866	0.837	0.939	7.00E-36	4	Camp
8.02E-40	-1.484759216	0.106	0.408	1.06E-35	4	Hist1h2ap
2.15E-39	0.968756615	0.294	0.108	2.83E-35	4	Smpdl3a
4.07E-38	0.705672762	0.629	0.436	5.36E-34	4	Alox5ap
2.31E-37	0.753349224	0.524	0.299	3.04E-33	4	Lsp1
2.17E-36	0.489427355	0.857	0.67	2.86E-32	4	Plac8
6.14E-36	-0.664306836	0.439	0.711	8.10E-32	4	Hmgb1

5.26E-35	0.813078069	0.52	0.325	6.94E-31	4	Ifitm2
5.32E-35	0.954819141	0.292	0.114	7.02E-31	4	Ctsc
1.19E-34	0.383257413	0.933	0.868	1.57E-30	4	Rps9
3.83E-34	0.808889954	0.441	0.234	5.05E-30	4	Tmcc1
8.24E-34	0.853242485	0.365	0.17	1.09E-29	4	Samhd1
1.18E-33	0.481262192	0.824	0.698	1.55E-29	4	Tpt1
1.49E-33	-0.898718434	0.137	0.436	1.96E-29	4	Stmn1
2.52E-33	-0.581558099	0.6	0.794	3.33E-29	4	Hmgb2
1.42E-32	0.768205901	0.502	0.307	1.87E-28	4	Npc2
1.90E-32	0.844818006	0.424	0.23	2.50E-28	4	Fxyd5
2.42E-32	-1.006621059	0.082	0.354	3.19E-28	4	2810417H13Rik
3.24E-32	0.696800562	0.612	0.435	4.27E-28	4	Psma7
1.89E-31	0.789619431	0.378	0.181	2.49E-27	4	Mpeg1
2.14E-31	0.343227513	0.976	0.874	2.83E-27	4	Rps14
6.41E-31	-1.050865977	0.035	0.276	8.45E-27	4	Gm10282
3.77E-30	-0.723038328	0.239	0.53	4.98E-26	4	Cbx3
1.89E-29	-0.963239717	0.063	0.315	2.49E-25	4	Cd24a
3.57E-29	0.816651805	0.271	0.111	4.70E-25	4	Ifi30
7.66E-29	0.515099035	0.749	0.603	1.01E-24	4	Ly6c2
8.18E-29	0.794908066	0.333	0.156	1.08E-24	4	Pld4
1.04E-28	0.262199994	0.996	0.953	1.37E-24	4	Rps27
1.09E-27	0.789223198	0.42	0.244	1.44E-23	4	Cebpb
4.31E-27	0.585889276	0.647	0.518	5.68E-23	4	Coro1a
1.49E-26	-0.839521189	0.094	0.337	1.96E-22	4	Top2a
7.89E-26	0.585754713	0.649	0.527	1.04E-21	4	Mrpl33
2.67E-25	0.81180992	0.286	0.132	3.53E-21	4	Anxa5
9.09E-25	0.65152918	0.543	0.401	1.20E-20	4	Atox1
1.78E-24	0.435938452	0.784	0.676	2.34E-20	4	Cyba
1.98E-24	-0.693354992	0.139	0.386	2.61E-20	4	Tuba1b
3.11E-24	0.740761665	0.443	0.282	4.10E-20	4	Sat1
5.01E-24	0.702168519	0.376	0.208	6.61E-20	4	Gm2a

4.76E-23	0.724733613	0.402	0.244	6.28E-19	4	Anxa2
7.86E-23	0.407891432	0.808	0.74	1.04E-18	4	Gpx1
1.23E-22	-0.689686695	0.273	0.516	1.62E-18	4	Tubb5
1.24E-21	0.388796777	0.849	0.787	1.64E-17	4	Gm9843
1.32E-21	0.778805795	0.28	0.137	1.74E-17	4	Capg
1.06E-20	0.492459661	0.6	0.458	1.39E-16	4	Ly6e
2.75E-20	0.275889993	0.937	0.837	3.63E-16	4	Rps27rt
5.22E-20	0.674296774	0.443	0.308	6.89E-16	4	Gm10116
6.99E-20	0.425081424	0.718	0.643	9.22E-16	4	Itm2b
1.19E-19	0.342643767	0.841	0.684	1.57E-15	4	Rpl10
1.65E-19	0.388084354	0.743	0.682	2.18E-15	4	Actg1
2.15E-19	-1.618567745	0.082	0.261	2.83E-15	4	Elane
6.37E-19	-0.507927088	0.165	0.393	8.41E-15	4	H2afv
1.22E-18	-0.543608077	0.133	0.342	1.61E-14	4	Cks2
4.94E-18	-0.512338985	0.196	0.426	6.52E-14	4	Anp32b
8.00E-18	0.610867268	0.504	0.395	1.06E-13	4	Dbi
8.32E-18	0.289109627	0.894	0.798	1.10E-13	4	Rpl34
1.07E-17	-0.579277223	0.198	0.413	1.41E-13	4	Dstn
1.31E-17	0.417278056	0.714	0.661	1.72E-13	4	Cox4i1
2.23E-17	0.446091353	0.618	0.492	2.94E-13	4	Cybb
2.35E-17	0.605289928	0.439	0.306	3.10E-13	4	Ptprc
2.68E-17	0.711614032	0.265	0.14	3.53E-13	4	Tpd52
2.82E-17	0.590477989	0.469	0.344	3.72E-13	4	Msrb1
3.10E-17	-0.548027859	0.182	0.389	4.09E-13	4	Srrm2
3.21E-17	0.651363006	0.329	0.201	4.24E-13	4	Lamp1
7.13E-17	0.274577363	0.904	0.785	9.41E-13	4	Rps11
7.62E-17	0.641430035	0.363	0.23	1.01E-12	4	Slfn2
8.35E-17	-0.579716195	0.155	0.357	1.10E-12	4	Set
1.09E-16	0.580838864	0.396	0.253	1.44E-12	4	S100a10
2.88E-16	-0.560342002	0.08	0.252	3.79E-12	4	Cks1b
4.52E-16	0.624565542	0.41	0.284	5.96E-12	4	Laptm5

7.01E-16	-0.452781716	0.149	0.351	9.24E-12	4	Nucks1
7.89E-16	0.316035059	0.822	0.777	1.04E-11	4	Shfm1
9.87E-16	0.323110308	0.767	0.701	1.30E-11	4	Sub1
2.69E-15	0.590840004	0.271	0.147	3.55E-11	4	Ctsh
3.35E-15	-1.116016217	0.12	0.282	4.42E-11	4	Mpo
4.47E-15	-0.518945872	0.086	0.253	5.90E-11	4	Ube2s
5.25E-15	0.541862142	0.465	0.355	6.93E-11	4	Serp1
6.61E-15	-1.033464677	0.127	0.285	8.72E-11	4	Prtn3
7.36E-15	-0.638590207	0.257	0.443	9.71E-11	4	Anxa1
1.21E-14	-0.458913698	0.196	0.4	1.59E-10	4	1810037I17Rik
1.29E-14	0.556199512	0.369	0.246	1.70E-10	4	Vim
1.03E-13	-0.387703545	0.184	0.381	1.36E-09	4	Ran
1.48E-13	-0.387766504	0.2	0.392	1.95E-09	4	Eif5a
2.04E-13	0.595411414	0.396	0.282	2.69E-09	4	Mgst1
2.46E-13	-0.372660779	0.192	0.387	3.24E-09	4	Srsf2
2.51E-13	0.569377978	0.32	0.203	3.31E-09	4	Ctsb
2.85E-13	0.575075212	0.3	0.187	3.76E-09	4	Emb
2.94E-13	-0.546767757	0.11	0.267	3.87E-09	4	Ncl
1.01E-12	-0.405688758	0.133	0.3	1.33E-08	4	Tagln2
1.64E-12	0.356349069	0.694	0.645	2.17E-08	4	Atp5l
1.80E-12	-0.389055178	0.457	0.652	2.38E-08	4	Xist
3.06E-12	0.623001059	0.259	0.158	4.04E-08	4	Napsa
4.70E-12	0.55119526	0.384	0.284	6.19E-08	4	Rsrp1
6.17E-12	-0.450979334	0.173	0.338	8.14E-08	4	Hsp90aa1
6.66E-12	0.485059202	0.506	0.439	8.78E-08	4	Arpc1b
8.82E-12	-0.365865705	0.171	0.346	1.16E-07	4	Irf2bp2
1.19E-11	-0.391040557	0.114	0.268	1.57E-07	4	Alyref
5.43E-11	0.471534283	0.484	0.412	7.17E-07	4	Cdc42
5.97E-11	0.364643599	0.631	0.569	7.88E-07	4	Rpl36al
6.18E-11	0.587609777	0.412	0.328	8.16E-07	4	Pomp
9.91E-11	-0.333859618	0.155	0.314	1.31E-06	4	Erh

2.34E-10	-0.291734103	0.251	0.438	3.09E-06	4	Tkt
3.37E-10	-0.276329888	0.251	0.438	4.45E-06	4	Arpp19
4.48E-10	-0.364431005	0.292	0.464	5.91E-06	4	Atpif1
4.52E-10	0.442273532	0.535	0.486	5.97E-06	4	Arpc2
5.37E-10	-0.313804152	0.143	0.293	7.08E-06	4	Hnrnpab
1.65E-09	0.250418785	0.684	0.593	2.18E-05	4	Rpl3
3.24E-09	-0.285434668	0.19	0.35	4.28E-05	4	Srsf3
6.24E-09	-0.349251414	0.19	0.336	8.23E-05	4	Ranbp1
8.67E-09	0.299869059	0.659	0.636	0.000114389	4	Sec61g
1.21E-08	-0.266909327	0.169	0.316	0.000160267	4	Tsix
1.28E-08	-0.313478325	0.194	0.338	0.000168834	4	Ap3s1
1.36E-08	0.527152424	0.304	0.223	0.000179314	4	Rrbp1
2.35E-08	0.493218241	0.324	0.245	0.000309547	4	Psmb8
2.99E-08	-0.266428062	0.222	0.38	0.000394485	4	Dek
4.93E-08	0.514258143	0.402	0.327	0.000650315	4	Notch2
5.57E-08	0.427869184	0.408	0.351	0.000734805	4	Fam49b
6.88E-08	-0.265621703	0.31	0.476	0.00090692	4	Rac2
1.18E-07	-0.316390726	0.357	0.495	0.001553614	4	Rpl12
1.51E-07	0.48922512	0.324	0.249	0.001992787	4	Btg1
2.32E-07	-0.318775247	0.243	0.379	0.003064131	4	Myh9
2.43E-07	0.490357859	0.29	0.217	0.003199025	4	Gltscr2
4.35E-07	0.431253826	0.406	0.359	0.005739538	4	BC005537
6.26E-07	-0.814012076	0.324	0.489	0.008253649	4	Retnlg
6.85E-07	0.522137409	0.302	0.237	0.009030659	4	Zfp36l2
7.79E-07	0.441717658	0.41	0.364	0.010275932	4	Capza2
8.50E-07	0.483392434	0.318	0.253	0.011209248	4	Ucp2
8.63E-07	0.473563806	0.339	0.282	0.011384373	4	Clta
9.91E-07	-0.275418715	0.153	0.267	0.013067747	4	Lmo4
1.01E-06	0.484266374	0.304	0.236	0.013339758	4	Msn
1.21E-06	0.446966274	0.373	0.322	0.015978574	4	Eif3f
1.42E-06	0.291948144	0.512	0.463	0.018781613	4	Rpl10-ps3

2.48E-06	-0.264054298	0.161	0.272	0.03276212	4	Ncf1
2.92E-06	0.458195903	0.337	0.276	0.03851991	4	Rap1b
2.93E-06	0.408358122	0.394	0.344	0.038586011	4	Ptpn18
2.97E-06	0.365641495	0.455	0.424	0.039143153	4	Eef2
4.86E-06	0.49967365	0.251	0.19	0.064157549	4	Klf13
5.01E-06	0.335778822	0.463	0.429	0.066049562	4	Tspo
5.86E-06	0.404705785	0.32	0.262	0.077268491	4	Cotl1
1.10E-05	0.392838359	0.429	0.396	0.145751532	4	Actr3
1.29E-05	0.400242084	0.257	0.197	0.170035432	4	Ccdc12
1.43E-05	0.268195977	0.512	0.466	0.188962712	4	Gm8730
1.96E-05	0.348992063	0.41	0.369	0.259181427	4	Arpc5
2.23E-05	0.318390504	0.492	0.481	0.294630762	4	Uqcrh
4.54E-05	0.425167031	0.318	0.273	0.599224943	4	Rac1
5.80E-05	0.457702173	0.251	0.201	0.765474287	4	Cmpk1
6.19E-05	0.439533967	0.373	0.347	0.816744104	4	Ndufa6
9.95E-05	0.366727413	0.396	0.363	1	4	Emp3
0.00011697	0.497470436	0.294	0.256	1	4	Aprt
0.00016622	0.432593661	0.339	0.302	1	4	Taldo1
0.00019248	0.392037116	0.345	0.316	1	4	Nedd8
0.00024724	0.400344591	0.292	0.253	1	4	Atp5c1
0.00028703	0.380769476	0.282	0.241	1	4	Rap1a
0.00031573	0.390647949	0.316	0.286	1	4	Sdcbp
0.00039651	0.396434997	0.296	0.261	1	4	Gnas
0.00043179	0.359502502	0.32	0.281	1	4	Nsa2
0.00044616	0.336960815	0.312	0.275	1	4	Iqgap1
0.00055751	0.386687461	0.335	0.309	1	4	Arf5
0.0006629	0.343884038	0.269	0.226	1	4	Sell
0.00068406	0.367179683	0.355	0.326	1	4	Cd53
0.0007006	0.274829739	0.473	0.474	1	4	H2afj
0.0008516	0.419157261	0.278	0.247	1	4	Capzb
0.00137008	0.45421617	0.324	0.314	1	4	Eif3k

0.0016941	0.391133458	0.302	0.275	1	4	Reep5	
0.00183871	0.319896712	0.384	0.376	1	4	Btf3	
0.0019749	0.310988654	0.32	0.295	1	4	Cdk2ap2	
0.00209655	0.375099119	0.324	0.305	1	4	Polr2l	
0.00230282	0.355752733	0.28	0.248	1	4	Ostf1	
0.00247058	0.345437849	0.304	0.279	1	4	Myl12b	
0.00249431	0.347363757	0.257	0.221	1	4	Spi1	
0.00426085	0.31527876	0.341	0.325	1	4	Lamtor4	
0.00554087	0.32138276	0.28	0.254	1	4	Flna	
0.00564429	0.347230902	0.259	0.232	1	4	Atp6v1f	
1.46E-146	1.123722329	0.937	0.56	1.93E-142	5	Hmgn2	**Neutrophil**
3.77E-143	1.478665363	0.629	0.176	4.97E-139	5	Fcnb	
5.43E-129	1.049275684	1	0.926	7.16E-125	5	Camp	
1.64E-111	0.955416772	0.996	0.838	2.16E-107	5	Ngp	
1.54E-107	0.81492306	0.998	0.602	2.03E-103	5	Wfdc21	
9.52E-98	0.872010436	1	0.999	1.26E-93	5	S100a8	
1.76E-97	0.890613292	1	0.994	2.33E-93	5	S100a9	
2.37E-95	0.845289285	0.918	0.524	3.13E-91	5	Chil3	
5.50E-95	-2.053441055	0.363	0.769	7.26E-91	5	Tmsb10	
3.79E-87	0.80312827	0.851	0.416	5.00E-83	5	Pglyrp1	
2.31E-86	0.756084256	0.944	0.528	3.05E-82	5	Lcn2	
1.12E-76	-0.71583715	0.886	0.962	1.48E-72	5	Rps27	
2.17E-68	-1.881692889	0.287	0.664	2.86E-64	5	Crip1	
2.72E-63	-0.771742223	0.73	0.893	3.59E-59	5	Rps14	
4.66E-61	-0.876897889	0.581	0.811	6.15E-57	5	Rps11	
9.55E-59	-0.894126304	0.583	0.8	1.26E-54	5	Rps5	
2.73E-58	0.980132094	0.525	0.237	3.61E-54	5	Gm10282	
3.85E-57	-1.465673151	0.197	0.57	5.07E-53	5	Psap	
1.42E-56	-0.753851217	0.646	0.859	1.88E-52	5	Rps27rt	
6.24E-56	-0.967619996	0.415	0.717	8.24E-52	5	Rpl10	
1.05E-55	-0.727208377	0.754	0.884	1.38E-51	5	Rpl18a	

1.82E-55	-1.643662036	0.136	0.504	2.40E-51	5	Lgals1
3.88E-55	-1.137453863	0.311	0.668	5.12E-51	5	Fth1
1.27E-51	-0.753020476	0.713	0.862	1.67E-47	5	Rpl13
2.42E-51	-0.659199084	0.803	0.909	3.19E-47	5	Rpl37
7.08E-50	-0.636812181	0.806	0.908	9.34E-46	5	Rps28
3.90E-49	-0.77269606	0.648	0.821	5.15E-45	5	Rpl13a
8.29E-49	-0.885198659	0.467	0.73	1.09E-44	5	Rplp0
4.87E-48	0.535114985	0.84	0.518	6.42E-44	5	Ltf
9.02E-48	-1.396474484	0.225	0.556	1.19E-43	5	S100a6
6.97E-47	-0.828804565	0.581	0.78	9.20E-43	5	Rpl36
7.03E-47	0.808038502	0.419	0.163	9.27E-43	5	Cebpe
3.03E-46	-0.734248601	0.585	0.796	4.00E-42	5	Rpl26
1.45E-45	-0.598657588	0.715	0.885	1.92E-41	5	Rps9
5.46E-45	-0.858030291	0.521	0.739	7.20E-41	5	Rplp1
7.24E-45	-0.622709897	0.784	0.905	9.56E-41	5	Rps19
8.27E-45	-0.868699368	0.4	0.679	1.09E-40	5	Eef1a1
1.53E-44	-0.757990684	0.497	0.739	2.02E-40	5	Rpl27a
3.04E-44	-0.558202493	0.914	0.959	4.00E-40	5	Rpl41
4.02E-44	-0.679931951	0.749	0.866	5.31E-40	5	Rpl35
1.19E-43	-1.262745984	0.123	0.452	1.58E-39	5	Prdx1
1.11E-42	-1.162501718	0.168	0.492	1.47E-38	5	Ly6e
2.41E-42	-0.7340518	0.538	0.763	3.18E-38	5	Rps6
8.72E-42	0.531204826	1	0.999	1.15E-37	5	Actb
2.10E-41	-0.693660718	0.579	0.787	2.77E-37	5	Rps3a1
2.90E-41	-0.664559952	0.693	0.847	3.82E-37	5	Rps18
4.03E-41	-0.75356719	0.523	0.741	5.31E-37	5	Rps4x
6.26E-40	-0.619590955	0.739	0.877	8.26E-36	5	Rpl32
1.79E-39	-0.655054137	0.644	0.824	2.36E-35	5	Rps23
1.83E-39	-0.695059557	0.613	0.837	2.41E-35	5	Ftl1
3.07E-39	-0.674919398	0.605	0.79	4.04E-35	5	Rpl39
4.64E-38	-0.641176688	0.598	0.781	6.12E-34	5	Rpl21

6.12E-38	-0.908031148	0.471	0.7	8.08E-34	5	Plac8
8.40E-38	0.663634343	0.691	0.461	1.11E-33	5	F630028O10Rik
1.57E-37	-0.852962553	0.354	0.619	2.08E-33	5	Rpl3
1.84E-37	-0.547867447	0.987	0.998	2.42E-33	5	Malat1
3.26E-37	-0.484437974	0.898	0.942	4.30E-33	5	Rpl37a
5.05E-37	-0.648672566	0.59	0.792	6.66E-33	5	Rplp2
1.12E-36	-0.84297824	0.326	0.595	1.48E-32	5	Rpl10a
1.12E-36	0.433503932	0.948	0.766	1.48E-32	5	Hmgb2
3.85E-36	-0.589900563	0.616	0.82	5.08E-32	5	Rpl34
4.74E-36	-0.665736861	0.518	0.738	6.25E-32	5	Rpl19
1.65E-35	-0.584848934	0.721	0.859	2.18E-31	5	Rpl38
2.19E-35	-0.730888588	0.456	0.692	2.89E-31	5	Gm2000
3.33E-35	-0.6926379	0.495	0.722	4.39E-31	5	Sub1
5.03E-35	-0.595217516	0.667	0.818	6.64E-31	5	Rps16
2.68E-34	-0.939084967	0.203	0.49	3.54E-30	5	Gm8730
3.49E-34	-0.952308005	0.402	0.65	4.61E-30	5	Cst3
3.62E-34	-0.745404161	0.492	0.701	4.78E-30	5	Rpl14
5.72E-34	-0.614217173	0.596	0.774	7.55E-30	5	Rps3
1.09E-33	-0.610205112	0.518	0.737	1.44E-29	5	Rpl6
1.44E-33	-0.610014648	0.598	0.775	1.90E-29	5	Rpl17
3.89E-33	0.854299826	0.559	0.354	5.13E-29	5	Myh9
5.41E-33	-0.38189166	0.959	0.977	7.14E-29	5	Rps29
6.27E-33	-1.544632541	0.108	0.378	8.27E-29	5	Ifi27l2a
1.00E-32	-0.587075066	0.555	0.76	1.32E-28	5	Rps15
1.51E-32	-1.305780926	0.071	0.336	1.99E-28	5	Ms4a6c
2.04E-32	0.620817935	0.609	0.38	2.69E-28	5	Dstn
1.06E-31	-0.726461766	0.352	0.609	1.40E-27	5	Rpl4
1.10E-31	-1.304102334	0.026	0.275	1.46E-27	5	Gm9844
2.24E-31	-0.63208726	0.566	0.736	2.95E-27	5	Rps26
2.76E-31	-0.575595755	0.551	0.764	3.64E-27	5	Rps15a
3.32E-31	-0.691374668	0.432	0.66	4.38E-27	5	Rpl18

7.35E-31	-0.580327285	0.594	0.756	9.70E-27	5	Rpl24
7.37E-31	-1.34466542	0.043	0.292	9.72E-27	5	Ccr2
2.00E-30	-0.653829888	0.423	0.664	2.64E-26	5	Gm10263
2.14E-30	-0.894114875	0.227	0.485	2.82E-26	5	Rpl10-ps3
4.31E-30	0.851361905	0.365	0.173	5.68E-26	5	Cap1
6.80E-30	-1.114858608	0.326	0.577	8.97E-26	5	Ifitm3
9.67E-30	-0.746587474	0.356	0.596	1.28E-25	5	Rpl15
2.40E-29	-0.992061851	0.197	0.457	3.16E-25	5	Lgals3
5.91E-29	-0.558929345	0.732	0.844	7.80E-25	5	Rps8
1.10E-28	-0.56566048	0.575	0.767	1.45E-24	5	Gm10076
2.24E-28	-0.446800594	0.743	0.887	2.96E-24	5	Rps27a
2.30E-28	-0.959960295	0.151	0.401	3.03E-24	5	Npm1
4.55E-28	-0.811440646	0.194	0.458	6.00E-24	5	Gnb2l1
5.45E-28	-0.595643724	0.562	0.787	7.20E-24	5	B2m
8.47E-28	-0.841168938	0.263	0.501	1.12E-23	5	Rpl12
1.16E-27	0.442673326	0.806	0.654	1.53E-23	5	Arhgdib
1.53E-27	-0.552106116	0.531	0.731	2.01E-23	5	Rpl11
2.21E-27	-0.487567689	0.678	0.83	2.92E-23	5	Rpl23a
2.32E-27	-0.600375241	0.516	0.705	3.06E-23	5	Rps7
2.40E-27	-1.140362443	0.028	0.252	3.16E-23	5	Zeb2
4.52E-27	-0.599559784	0.518	0.705	5.97E-23	5	Rps13
8.94E-27	-1.123599158	0.052	0.28	1.18E-22	5	S100a10
1.31E-26	-0.553847221	0.583	0.754	1.73E-22	5	Rpl23
4.39E-26	-0.558076451	0.475	0.7	5.80E-22	5	Rpsa
4.49E-26	0.622491764	0.553	0.371	5.92E-22	5	1810037I17Rik
1.78E-25	-0.795436475	0.225	0.456	2.35E-21	5	Gm10073
2.74E-25	-0.657063063	0.272	0.525	3.61E-21	5	Sec61b
5.42E-25	-0.953063733	0.108	0.339	7.15E-21	5	Npc2
5.50E-25	-0.810627235	0.199	0.436	7.26E-21	5	Hsp90ab1
9.80E-25	-0.544690043	0.497	0.694	1.29E-20	5	Rpl27
1.19E-24	-0.570965323	0.456	0.662	1.57E-20	5	Rpl8

2.04E-24	-0.761579635	0.218	0.451	2.69E-20	5	Rpl13-ps3
2.51E-24	-0.59289144	0.691	0.825	3.32E-20	5	Tyrobp
3.77E-24	0.632269845	0.395	0.21	4.97E-20	5	Cd63
5.95E-24	-0.754376572	0.227	0.466	7.85E-20	5	Psma7
1.15E-23	-0.611810064	0.497	0.672	1.52E-19	5	Rps12
1.81E-23	-0.358984415	0.834	0.926	2.39E-19	5	Fau
2.71E-23	-0.893941599	0.084	0.3	3.57E-19	5	Eef1g
2.92E-23	-0.70341652	0.367	0.569	3.86E-19	5	Rps2
3.83E-23	-0.515101367	0.559	0.721	5.06E-19	5	Rpl28
1.64E-22	-0.532271189	0.551	0.721	2.16E-18	5	Rpl31
2.63E-22	-0.660125975	0.361	0.594	3.47E-18	5	H2-D1
2.99E-22	-0.913824965	0.095	0.31	3.94E-18	5	Pdcd4
3.10E-22	-0.764799539	0.201	0.419	4.09E-18	5	Rpl36-ps3
3.38E-22	-0.609822478	0.477	0.657	4.46E-18	5	Rpl36a
3.39E-22	-0.5361887	0.492	0.67	4.47E-18	5	Rps10
3.59E-22	-0.514157648	0.486	0.685	4.73E-18	5	Rpl23a-ps3
3.14E-21	-0.433593152	0.706	0.812	4.14E-17	5	Rps24
8.13E-21	-0.580657603	0.419	0.604	1.07E-16	5	Wdr89
8.68E-21	-0.657861282	0.22	0.443	1.15E-16	5	Eef2
3.52E-20	0.596106371	0.305	0.151	4.64E-16	5	Ms4a3
3.71E-20	-0.459995528	0.549	0.72	4.89E-16	5	Tpt1
7.29E-20	-0.60485034	0.302	0.519	9.62E-16	5	Mbnl1
1.19E-19	-0.610267786	0.339	0.54	1.57E-15	5	Rpl22
3.78E-19	-0.625872351	0.283	0.485	4.98E-15	5	Rps18-ps3
4.75E-19	-0.633259118	0.279	0.471	6.27E-15	5	Rps12-ps3
8.03E-19	-0.81404854	0.073	0.258	1.06E-14	5	Fxyd5
9.34E-19	0.704388972	0.328	0.182	1.23E-14	5	Rrm2
1.65E-18	-0.500383883	0.523	0.673	2.17E-14	5	Rps20
1.85E-18	-0.469921167	0.475	0.65	2.44E-14	5	Sec61g
1.99E-18	-0.504026673	0.441	0.621	2.63E-14	5	Rps21
3.15E-18	-0.575629502	0.279	0.479	4.15E-14	5	Rpl30

3.25E-18	-0.660505759	0.136	0.339	4.29E-14	5	Slc25a5
1.23E-17	-0.412446549	0.607	0.737	1.63E-13	5	Rps25
1.32E-17	-0.550777424	0.242	0.446	1.74E-13	5	Rpl5
1.55E-17	-0.668495589	0.153	0.349	2.05E-13	5	Pomp
1.76E-17	-0.44730812	0.473	0.663	2.32E-13	5	Atp5l
2.21E-17	-0.589481919	0.302	0.489	2.91E-13	5	Rps26-ps1
2.48E-17	-0.745711425	0.08	0.257	3.27E-13	5	Calr
2.90E-17	-0.549789092	0.32	0.513	3.82E-13	5	Rpl7a
3.30E-17	-0.3740177	0.667	0.801	4.36E-13	5	Gm9843
1.67E-16	-0.706657389	0.177	0.358	2.20E-12	5	H2afy
1.75E-16	-0.552333908	0.305	0.488	2.31E-12	5	Rpl27-ps3
2.16E-16	-0.634930901	0.151	0.339	2.85E-12	5	Eif3f
2.20E-16	-0.581053279	0.192	0.39	2.90E-12	5	Btf3
2.36E-16	-0.70628609	0.181	0.372	3.12E-12	5	H2-K1
2.53E-16	-0.64359961	0.136	0.319	3.34E-12	5	Polr2l
3.61E-16	-0.327715434	0.816	0.877	4.76E-12	5	Rpl35a
3.74E-16	0.435749444	0.393	0.236	4.93E-12	5	Elane
9.31E-16	-0.666139636	0.147	0.329	1.23E-11	5	Ptprc
2.41E-15	0.397117704	0.592	0.444	3.18E-11	5	Hp
2.89E-15	-0.509385236	0.276	0.465	3.81E-11	5	Naca
3.74E-15	-0.492932694	0.384	0.56	4.94E-11	5	Rpl7
6.01E-15	-0.470689393	0.38	0.558	7.92E-11	5	Hspa8
7.68E-15	0.304342079	0.762	0.636	1.01E-10	5	Prdx5
7.77E-15	-0.70564953	0.16	0.328	1.03E-10	5	Lsp1
1.31E-14	-0.45435064	0.419	0.585	1.73E-10	5	Rpl36al
2.09E-14	-0.369448669	0.631	0.754	2.76E-10	5	Rpl9
2.13E-14	-0.393183981	0.564	0.715	2.81E-10	5	mt-Cytb
2.71E-14	-0.321008764	0.786	0.865	3.57E-10	5	mt-Atp6
4.59E-14	-0.541092871	0.156	0.331	6.06E-10	5	Gm10116
6.33E-14	-0.688430561	0.11	0.266	8.35E-10	5	Ncl
6.64E-14	0.575658439	0.33	0.203	8.76E-10	5	Serpinb1a

7.18E-14	0.439446346	0.397	0.253	9.47E-10	5	Ncf1
7.21E-14	-0.455723859	0.27	0.462	9.51E-10	5	Rpl9-ps6
7.35E-14	-0.577606369	0.181	0.349	9.69E-10	5	Nop10
8.81E-14	-0.548354819	0.203	0.369	1.16E-09	5	Snrpf
1.28E-13	-0.533765589	0.287	0.446	1.68E-09	5	Gm9493
1.89E-13	0.284688248	0.592	0.417	2.50E-09	5	Anxa1
2.34E-13	-0.475173951	0.33	0.489	3.09E-09	5	Rps27l
3.62E-13	0.518544868	0.257	0.141	4.77E-09	5	Lta4h
4.33E-13	-0.384571729	0.497	0.642	5.71E-09	5	Uba52
4.52E-13	-0.364772514	0.531	0.676	5.96E-09	5	Cox4i1
4.55E-13	-0.470423308	0.192	0.363	6.00E-09	5	Rpl6l
6.74E-13	-0.57520516	0.108	0.262	8.89E-09	5	Psmb8
8.17E-13	-0.472021213	0.268	0.439	1.08E-08	5	Hint1
1.10E-12	0.474728093	0.335	0.204	1.45E-08	5	C3
1.11E-12	-0.532582074	0.138	0.295	1.46E-08	5	Gm10269
1.12E-12	-0.459223524	0.527	0.645	1.48E-08	5	Xist
2.09E-12	0.471004322	0.302	0.177	2.76E-08	5	Adpgk
2.53E-12	-0.543852767	0.119	0.269	3.34E-08	5	Ucp2
3.06E-12	-0.471806352	0.274	0.444	4.03E-08	5	Serinc3
6.32E-12	-0.36767818	0.464	0.623	8.34E-08	5	Gm11808
7.74E-12	-0.516799899	0.173	0.325	1.02E-07	5	Atp5g1
8.38E-12	-0.513321658	0.175	0.329	1.11E-07	5	Eef1b2
1.42E-11	-0.505536557	0.162	0.315	1.87E-07	5	mt-Nd2
1.42E-11	-0.37025391	0.434	0.581	1.87E-07	5	mt-Nd4
1.65E-11	-0.535436532	0.197	0.352	2.18E-07	5	Hsp90b1
1.79E-11	-0.348867088	0.631	0.728	2.36E-07	5	Rps17
1.91E-11	-0.524965051	0.147	0.295	2.52E-07	5	Nsa2
3.82E-11	-0.587537257	0.147	0.289	5.04E-07	5	Slpi
4.10E-11	-0.463046093	0.121	0.266	5.40E-07	5	Atp5c1
5.33E-11	-0.288671101	0.575	0.706	7.04E-07	5	Oaz1
5.48E-11	-0.421953709	0.33	0.481	7.23E-07	5	Rpl29

5.65E-11	-0.404699264	0.179	0.332	7.45E-07	5	Gm10036
9.36E-11	-0.414424562	0.207	0.359	1.23E-06	5	Cox7b
9.42E-11	0.40632041	0.475	0.357	1.24E-06	5	Gsr
1.25E-10	0.481109632	0.281	0.173	1.65E-06	5	Ckap4
4.13E-10	-0.466940603	0.119	0.252	5.45E-06	5	Zfp36l2
5.11E-10	-0.446407163	0.136	0.272	6.74E-06	5	Snrpd2
6.18E-10	-0.396319616	0.302	0.444	8.15E-06	5	Ybx1
6.66E-10	-0.426711375	0.261	0.407	8.79E-06	5	mt-Atp8
7.55E-10	0.413784062	0.43	0.319	9.95E-06	5	Ap3s1
7.94E-10	-0.369724434	0.339	0.493	1.05E-05	5	Uqcrh
9.29E-10	-0.460786756	0.164	0.301	1.23E-05	5	Rsrp1
9.39E-10	-0.380587924	0.253	0.403	1.24E-05	5	Gm8186
9.98E-10	-0.374306946	0.629	0.721	1.32E-05	5	Fcer1g
1.04E-09	-0.39221	0.369	0.516	1.37E-05	5	Rpl22l1
1.47E-09	-0.370789228	0.395	0.547	1.93E-05	5	Mrpl33
1.51E-09	0.405459664	0.432	0.324	2.00E-05	5	Clec12a
1.88E-09	-0.416730419	0.16	0.297	2.47E-05	5	Psmb1
2.26E-09	-0.346012516	0.263	0.408	2.98E-05	5	Tomm7
2.65E-09	-0.480171686	0.171	0.304	3.49E-05	5	Sat1
2.72E-09	-0.360751487	0.177	0.315	3.59E-05	5	Hnrnpm
3.27E-09	-0.37895726	0.16	0.296	4.31E-05	5	Eif4a1
3.55E-09	-0.405925128	0.207	0.351	4.68E-05	5	Ifitm2
3.79E-09	0.395535014	0.369	0.258	5.00E-05	5	Clec4a2
4.02E-09	-0.381240394	0.177	0.311	5.30E-05	5	Erh
4.76E-09	0.391269571	0.425	0.316	6.27E-05	5	Scp2
4.97E-09	-0.39263833	0.13	0.257	6.56E-05	5	Eif3h
5.85E-09	-0.539786534	0.145	0.263	7.72E-05	5	Btg1
7.92E-09	-0.425310164	0.235	0.373	0.000104461	5	Serp1
9.03E-09	0.423513438	0.387	0.286	0.00011907	5	Ppp1cb
9.73E-09	-0.430342806	0.218	0.351	0.00012841	5	Set
1.00E-08	-0.336826323	0.499	0.629	0.000132362	5	mt-Nd1

1.18E-08	-0.337923259	0.281	0.422	0.00015591	5	Chchd2
1.21E-08	-0.344233396	0.136	0.265	0.000160076	5	Ndufb8
1.28E-08	-0.383106894	0.184	0.317	0.000168742	5	Canx
2.59E-08	-0.359909075	0.181	0.308	0.000341199	5	Atp5g2
3.04E-08	-0.372535581	0.24	0.372	0.000401619	5	BC005537
3.80E-08	-0.339038926	0.134	0.257	0.000501667	5	Rbm25
5.50E-08	-0.322815524	0.279	0.416	0.000725632	5	Uqcrq
5.57E-08	-0.357841958	0.14	0.258	0.000734518	5	Capzb
5.61E-08	-0.334036594	0.356	0.488	0.000739576	5	Morf4l1
6.65E-08	-0.301956766	0.328	0.468	0.000877612	5	Dynll1
9.15E-08	0.36158482	0.361	0.262	0.001207564	5	Gpi1
9.95E-08	-0.353811263	0.317	0.436	0.001311938	5	Serbp1
1.10E-07	-0.319909209	0.283	0.422	0.00145454	5	2010107E04Rik
1.32E-07	-0.325831354	0.276	0.414	0.001745314	5	Pfdn5
1.55E-07	-0.394665773	0.171	0.287	0.002050443	5	Iqgap1
1.70E-07	-0.362613498	0.322	0.44	0.002241218	5	Tspo
1.72E-07	-0.339416529	0.158	0.277	0.002270456	5	Ndufb9
2.35E-07	-0.985234303	0.356	0.486	0.003099257	5	Retnlg
2.36E-07	-0.453957274	0.227	0.341	0.003116748	5	Notch2
2.78E-07	-0.415188481	0.19	0.302	0.003671819	5	Laptm5
2.81E-07	-0.342505691	0.173	0.286	0.003711563	5	Bola2
3.41E-07	0.328290746	0.495	0.418	0.004502823	5	Tkt
3.60E-07	-0.322745016	0.147	0.261	0.004744113	5	Timm13
3.93E-07	-0.414695478	0.158	0.264	0.005182009	5	Anxa2
4.19E-07	0.343486105	0.346	0.251	0.005523143	5	Lmo4
4.32E-07	-0.352998212	0.225	0.344	0.005701782	5	Hnrnpa1
5.17E-07	-0.317514562	0.205	0.323	0.00681615	5	Eif3k
5.94E-07	-0.292490145	0.337	0.46	0.007841667	5	Uqcr11
7.64E-07	-0.26551532	0.149	0.261	0.01007375	5	Eif3a
8.35E-07	0.348269552	0.311	0.223	0.011021064	5	Cpne3
9.23E-07	0.465146493	0.261	0.189	0.012173603	5	Tuba4a

1.03E-06	-0.317662871	0.158	0.268	0.013600662	5	Atp5g3
1.07E-06	-0.315511716	0.22	0.334	0.014061342	5	Hsp90aa1
1.12E-06	-0.285970532	0.428	0.527	0.014709778	5	Snrpg
1.20E-06	-0.286832011	0.266	0.395	0.015821972	5	Tmem258
1.27E-06	-0.341156269	0.147	0.251	0.01673028	5	Atp5f1
1.29E-06	0.407925318	0.27	0.187	0.017012644	5	R3hdm4
1.32E-06	-0.253990582	0.207	0.327	0.017406126	5	Nedd8
1.32E-06	-0.324099941	0.186	0.295	0.017449575	5	Clta
1.34E-06	-0.307053989	0.296	0.413	0.017627248	5	Ndufa13
1.44E-06	-0.270166559	0.177	0.29	0.018930187	5	Atp5b
1.77E-06	-0.306916965	0.177	0.286	0.023309355	5	Atp6v1g1
1.96E-06	0.394027663	0.313	0.235	0.025799364	5	Rnaseh2c
2.03E-06	-0.332094888	0.222	0.333	0.026761368	5	Ranbp1
2.21E-06	0.373760684	0.356	0.282	0.029099416	5	Ndufv3
2.89E-06	-0.31500228	0.192	0.301	0.038122948	5	Nap1l1
3.25E-06	0.299032587	0.296	0.207	0.042809661	5	Cd177
4.44E-06	-0.269435745	0.346	0.459	0.058548065	5	Usmg5
4.98E-06	0.408521189	0.354	0.283	0.065742407	5	Lrrc58
5.07E-06	-0.263642067	0.3	0.42	0.066863009	5	Atox1
5.30E-06	-0.257359016	0.197	0.306	0.069922864	5	Ndufc1
5.38E-06	-0.268415991	0.274	0.391	0.070975414	5	Minos1
5.90E-06	-0.277143737	0.313	0.425	0.077852786	5	Cdc42
6.20E-06	-0.308775781	0.212	0.32	0.081749417	5	Cycs
6.53E-06	0.274289491	0.525	0.465	0.086143668	5	Txn1
6.96E-06	0.281093595	0.371	0.29	0.091824562	5	Cd24a
7.30E-06	0.301652728	0.389	0.313	0.09625743	5	Top2a
8.56E-06	-0.279008838	0.229	0.334	0.112892575	5	Ndufa7
9.61E-06	-0.251090375	0.369	0.483	0.126814198	5	H2afj
1.00E-05	-0.43178563	0.168	0.256	0.132482776	5	Tmcc1
1.05E-05	-0.292346442	0.179	0.277	0.13825732	5	Dazap2
1.16E-05	-0.294525195	0.179	0.278	0.152399399	5	Romo1

1.32E-05	-0.293202545	0.166	0.263	0.174349101	5	Vim	
1.38E-05	-0.289128926	0.173	0.271	0.181814494	5	Gnas	
1.51E-05	-0.352348032	0.199	0.291	0.199029301	5	Cmtm7	
1.80E-05	0.449723144	0.281	0.214	0.23678271	5	Hnrnpul1	
2.39E-05	0.26384953	0.514	0.46	0.31582521	5	Rac2	
2.58E-05	-0.26037136	0.356	0.458	0.340115731	5	Alox5ap	
2.82E-05	0.261115007	0.402	0.328	0.37157002	5	2810417H13Rik	
3.07E-05	-0.320749008	0.266	0.363	0.405446746	5	Gm42418	
3.56E-05	-0.301587283	0.257	0.355	0.469823966	5	Ptpn18	
7.62E-05	-0.252863453	0.168	0.256	1	5	Psmb3	
9.27E-05	-0.26974932	0.317	0.41	1	5	Dbi	
0.00010112	-0.251724434	0.685	0.734	1	5	Cd52	
0.0001683	-0.280689137	0.201	0.283	1	5	Reep5	
0.00022536	0.250909154	0.467	0.421	1	5	Arpp19	
0.0002523	-0.266984759	0.544	0.62	1	5	Ly6c2	
0.00028143	-0.255219872	0.179	0.257	1	5	Ndufb2	
0.00049289	0.277344165	0.27	0.206	1	5	Mcemp1	
0.00049739	0.291307633	0.328	0.27	1	5	Aldh2	
0.00051224	-0.283205817	0.292	0.37	1	5	Lst1	
0.00076668	0.320010758	0.283	0.231	1	5	Abhd2	
0.00110563	0.30629115	0.261	0.214	1	5	Mki67	
0.00143278	0.353329928	0.268	0.226	1	5	Smc2	
0.00201372	-0.294564928	0.201	0.262	1	5	Cebpb	
0.00570131	-0.311683858	0.307	0.364	1	5	Ifitm6	
1.22E-137	0.985181641	1	0.72	1.61E-133	6	Tmsb10	**Mono**
1.31E-120	0.997834288	0.974	0.611	1.73E-116	6	Crip1	
1.14E-74	0.954086485	0.603	0.238	1.51E-70	6	S100a10	
8.26E-72	0.567980368	0.972	0.832	1.09E-67	6	Ptma	
1.58E-60	0.821387136	0.638	0.292	2.08E-56	6	Ms4a6c	
8.09E-58	0.673148559	0.792	0.453	1.07E-53	6	Lgals1	
3.69E-57	0.679899797	0.837	0.597	4.87E-53	6	Ly6c2	

2.07E-52	0.830610379	0.497	0.2	2.72E-48	6	F13a1
6.02E-49	0.739279	0.54	0.236	7.95E-45	6	Gm9844
2.39E-46	-1.805390289	0.397	0.648	3.16E-42	6	Wfdc21
2.65E-46	0.458131852	0.941	0.811	3.49E-42	6	Ftl1
3.75E-46	0.506789049	0.857	0.615	4.94E-42	6	Cst3
1.72E-45	0.77685158	0.618	0.349	2.27E-41	6	Dek
3.13E-43	0.837471603	0.573	0.315	4.13E-39	6	2810417H13Rik
6.31E-43	0.606113476	0.725	0.416	8.32E-39	6	Lgals3
5.07E-42	-1.881160199	0.987	0.995	6.69E-38	6	S100a9
9.50E-42	-1.543024791	0.169	0.469	1.25E-37	6	Pglyrp1
8.55E-41	0.472018552	0.926	0.768	1.13E-36	6	Hmgb2
2.80E-40	0.463844556	0.92	0.782	3.69E-36	6	Ppia
2.84E-40	0.763654548	0.258	0.076	3.75E-36	6	Klf4
4.09E-40	-1.793391255	0.879	0.935	5.40E-36	6	Camp
4.39E-40	-1.644151002	0.293	0.56	5.79E-36	6	Ltf
1.97E-39	0.833458114	0.62	0.367	2.60E-35	6	Hist1h2ap
2.60E-39	-1.844125373	0.764	0.856	3.43E-35	6	Ngp
5.69E-39	0.501870254	0.811	0.522	7.51E-35	6	Psap
2.26E-38	0.424207533	0.946	0.81	2.98E-34	6	H2afz
2.69E-38	0.507048493	1	0.999	3.55E-34	6	Actb
6.41E-37	0.456042627	0.816	0.51	8.46E-33	6	S100a6
1.08E-36	0.610875313	0.681	0.431	1.42E-32	6	Psma7
1.09E-35	-1.776268892	0.998	0.999	1.43E-31	6	S100a8
1.63E-35	0.461827958	0.87	0.677	2.15E-31	6	Hmgb1
1.77E-35	0.736308854	0.26	0.085	2.34E-31	6	C1galt1c1
6.26E-35	-1.61062819	0.345	0.575	8.26E-31	6	Lcn2
3.50E-34	0.598969151	0.46	0.208	4.61E-30	6	S100a4
5.83E-31	0.695121481	0.46	0.242	7.69E-27	6	Cebpb
1.55E-29	0.580071339	0.644	0.412	2.05E-25	6	Prdx1
2.32E-29	0.597690584	0.69	0.483	3.05E-25	6	Tubb5
5.31E-29	0.649202791	0.568	0.354	7.00E-25	6	Myh9

6.32E-27	0.584165276	0.597	0.388	8.33E-23	6	Dbi
2.34E-25	0.777435383	0.28	0.12	3.08E-21	6	Hist1h2ae
2.80E-25	0.593580612	0.395	0.196	3.70E-21	6	Fn1
1.47E-24	0.321565433	0.931	0.765	1.94E-20	6	Rpl39
2.12E-24	0.357441879	0.866	0.713	2.80E-20	6	Rps26
2.20E-24	-0.483921926	0.998	0.997	2.90E-20	6	Malat1
6.75E-23	0.376837049	0.813	0.697	8.90E-19	6	Sub1
2.50E-22	0.558638444	0.551	0.353	3.29E-18	6	Tuba1b
2.11E-21	0.593340201	0.508	0.32	2.78E-17	6	Notch2
3.94E-21	0.524957227	0.584	0.414	5.20E-17	6	Hint1
1.73E-20	0.375816842	0.471	0.259	2.28E-16	6	Ccr2
5.81E-20	0.553718893	0.375	0.2	7.67E-16	6	Ahnak
6.23E-20	0.504711	0.575	0.401	8.21E-16	6	Stmn1
1.33E-19	0.4372617	0.688	0.524	1.75E-15	6	Mrpl33
2.58E-18	0.383939608	0.636	0.456	3.41E-14	6	Ly6e
2.80E-18	0.319089959	0.839	0.737	3.69E-14	6	Gpx1
5.65E-17	0.529182254	0.356	0.199	7.45E-13	6	Lamp1
7.29E-17	0.463257326	0.568	0.421	9.61E-13	6	Tspo
1.67E-16	0.47719878	0.338	0.184	2.20E-12	6	Emb
1.75E-16	-0.9116777	0.193	0.373	2.30E-12	6	Ifitm6
1.78E-16	0.409945559	0.664	0.524	2.35E-12	6	Atp5k
1.98E-16	0.618097554	0.319	0.177	2.62E-12	6	Cap1
5.49E-16	-0.789685181	0.275	0.441	7.24E-12	6	Anxa1
5.62E-16	0.284363424	0.837	0.712	7.42E-12	6	Rps17
5.89E-16	0.522752357	0.453	0.302	7.77E-12	6	Cycs
6.37E-16	0.486488355	0.466	0.307	8.41E-12	6	Gm10116
1.56E-15	0.523586562	0.358	0.21	2.06E-11	6	Gm2a
3.22E-15	0.374124386	0.664	0.496	4.25E-11	6	Cbx3
4.73E-15	0.482309548	0.38	0.229	6.23E-11	6	Pycard
4.86E-15	0.431850581	0.403	0.245	6.41E-11	6	Anxa2
9.84E-15	-0.689261488	0.141	0.308	1.30E-10	6	Cd24a

1.35E-14	0.436942373	0.514	0.365	1.78E-10	6	H2afv
1.76E-14	0.263601021	0.855	0.711	2.32E-10	6	Rpl6
3.33E-14	0.256797106	0.844	0.719	4.40E-10	6	Rps25
7.75E-14	0.400961882	0.51	0.357	1.02E-09	6	Capza2
8.10E-14	0.500766565	0.258	0.135	1.07E-09	6	Anxa5
8.56E-14	0.313195415	0.768	0.68	1.13E-09	6	Actg1
1.64E-13	0.267076597	0.742	0.611	2.17E-09	6	mt-Nd1
2.27E-13	0.470445682	0.456	0.315	3.00E-09	6	Ranbp1
2.52E-13	0.463602715	0.39	0.245	3.32E-09	6	Vim
3.37E-13	0.407855451	0.414	0.267	4.45E-09	6	Reep5
6.18E-13	0.340758427	0.633	0.505	8.15E-09	6	Cox6a1
8.22E-13	0.279991398	0.759	0.621	1.08E-08	6	Uba52
8.52E-13	0.269264384	0.852	0.769	1.12E-08	6	H3f3a
8.96E-13	0.454728204	0.341	0.206	1.18E-08	6	Erp29
1.18E-12	0.250748608	0.809	0.648	1.55E-08	6	Eef1a1
1.36E-12	0.347710656	0.681	0.559	1.79E-08	6	Hnrnpa2b1
1.62E-12	0.411217965	0.469	0.325	2.14E-08	6	Hnrnpa1
2.39E-12	0.509570181	0.41	0.279	3.15E-08	6	Lrrc58
2.64E-12	0.404761978	0.49	0.353	3.48E-08	6	BC005537
4.62E-12	0.438127635	0.523	0.399	6.09E-08	6	Anp32b
7.71E-12	0.369732027	0.618	0.513	1.02E-07	6	Atp5h
8.45E-12	0.466420785	0.44	0.309	1.11E-07	6	Top2a
9.19E-12	0.388825503	0.557	0.433	1.21E-07	6	Actr2
1.02E-11	0.278759335	0.725	0.602	1.35E-07	6	Gm11808
3.53E-11	0.293447723	0.659	0.516	4.66E-07	6	Rpl22
6.66E-11	0.514853192	0.325	0.216	8.78E-07	6	Snrpd1
6.87E-11	0.348667669	0.592	0.466	9.06E-07	6	Rps26-ps1
7.84E-11	0.338356589	0.449	0.312	1.03E-06	6	Npc2
8.80E-11	0.350174296	0.341	0.211	1.16E-06	6	Selplg
1.05E-10	0.493745647	0.39	0.277	1.39E-06	6	Tmpo
1.58E-10	0.325623943	0.588	0.466	2.08E-06	6	H2afj

1.62E-10	0.260286157	0.768	0.687	2.14E-06	6	Sh3bgrl3
1.64E-10	-0.382540384	0.321	0.477	2.17E-06	6	Cd47
1.66E-10	0.328064464	0.655	0.548	2.20E-06	6	Calm1
2.30E-10	0.325757671	0.473	0.335	3.03E-06	6	H2afy
3.24E-10	0.359932531	0.536	0.419	4.27E-06	6	Rbm3
7.85E-10	0.259098179	0.744	0.659	1.04E-05	6	Cox4i1
1.05E-09	0.348035221	0.479	0.357	1.39E-05	6	Emp3
1.23E-09	0.297957875	0.625	0.512	1.62E-05	6	Snrpg
1.27E-09	0.376761376	0.495	0.382	1.68E-05	6	Smdt1
1.33E-09	0.306437597	0.547	0.435	1.76E-05	6	Hnrnpf
1.74E-09	0.268581563	0.677	0.566	2.30E-05	6	Rpl36al
2.30E-09	0.352919126	0.503	0.383	3.04E-05	6	Gm8186
2.52E-09	0.342914756	0.471	0.358	3.32E-05	6	Ran
2.59E-09	0.332884232	0.549	0.443	3.41E-05	6	Hnrnpa3
3.23E-09	0.447546021	0.28	0.177	4.25E-05	6	Fam96a
4.75E-09	0.462822225	0.338	0.234	6.27E-05	6	Msn
4.93E-09	0.32176491	0.449	0.328	6.51E-05	6	Clic1
1.37E-08	0.464110586	0.295	0.199	0.000180911	6	Birc5
1.46E-08	0.275997621	0.636	0.538	0.000192349	6	Hspa8
1.52E-08	0.400768443	0.321	0.222	0.000200866	6	Smc2
2.22E-08	0.254888893	0.696	0.619	0.000293132	6	Cox6c
2.37E-08	0.335809424	0.438	0.328	0.000313193	6	Nucks1
2.82E-08	0.369066123	0.525	0.429	0.000372534	6	Rhoa
2.96E-08	0.319084702	0.544	0.453	0.00039025	6	Ndufa2
3.49E-08	0.356619431	0.451	0.35	0.000459858	6	Snrpf
7.18E-08	0.282299608	0.54	0.432	0.000946969	6	Gm10073
7.18E-08	0.413051543	0.345	0.25	0.000947741	6	Alyref
8.90E-08	0.295998965	0.557	0.471	0.0011737	6	Rps27l
2.08E-07	-0.38969913	0.412	0.508	0.002744529	6	Cybb
2.08E-07	0.408038944	0.291	0.199	0.00274604	6	Tacc1
2.12E-07	0.326763346	0.412	0.317	0.002792955	6	Slc25a5

2.77E-07	0.353200522	0.347	0.251	0.003649302	6	Ucp2
4.28E-07	0.378184633	0.26	0.176	0.00564499	6	Pcna
4.45E-07	0.322767031	0.462	0.366	0.005863581	6	Srrm2
5.07E-07	-0.425495916	0.165	0.271	0.00669247	6	Ncf1
5.66E-07	0.314539139	0.466	0.386	0.007465589	6	Ywhaz
5.72E-07	-0.529863427	0.514	0.555	0.007541864	6	Chil3
7.08E-07	0.314790791	0.436	0.342	0.009335492	6	Cox7b
7.43E-07	0.267803928	0.427	0.328	0.009803565	6	Pomp
8.76E-07	0.342957015	0.299	0.211	0.011551934	6	Mki67
1.22E-06	-0.260500988	0.592	0.67	0.016110772	6	Arhgdib
1.46E-06	0.36427932	0.36	0.274	0.019258962	6	Anapc13
1.47E-06	0.251475119	0.644	0.554	0.019404998	6	Calm2
1.50E-06	0.311081674	0.304	0.216	0.019787126	6	Rbm8a
1.63E-06	0.310585078	0.484	0.4	0.021436224	6	Uqcrq
1.84E-06	0.306897623	0.451	0.366	0.024287393	6	Srsf2
2.24E-06	0.350406655	0.252	0.175	0.02954791	6	Dnmt1
2.75E-06	0.394097498	0.302	0.224	0.036266943	6	Anp32e
3.92E-06	0.257184806	0.562	0.484	0.051707045	6	Gnai2
5.79E-06	0.302497085	0.317	0.23	0.076352914	6	Zeb2
5.80E-06	0.381245365	0.289	0.213	0.076454083	6	Hnrnpul1
7.40E-06	0.418416665	0.308	0.234	0.097570618	6	Cks1b
7.70E-06	0.387996106	0.334	0.258	0.101528092	6	Prrc2c
9.66E-06	0.333586782	0.262	0.184	0.127476979	6	2700094K13Rik
1.07E-05	0.38586687	0.319	0.244	0.140778787	6	Sec11c
1.07E-05	0.313865094	0.377	0.298	0.141348606	6	Uqcrb
1.09E-05	-0.289232827	0.178	0.276	0.144033798	6	Gpi1
1.29E-05	-0.265022663	0.36	0.472	0.169836193	6	Rac2
1.49E-05	0.324943483	0.358	0.281	0.197161793	6	Clta
1.67E-05	0.346010176	0.256	0.184	0.219989736	6	Psmb2
1.88E-05	0.364347505	0.345	0.273	0.247644791	6	mt-Nd4l
2.09E-05	0.36208124	0.306	0.236	0.275537988	6	Ube2s

2.24E-05	-0.344727009	0.191	0.281	0.295606841	6	Aldh2
2.39E-05	0.301591247	0.38	0.3	0.315284539	6	Polr2l
2.44E-05	0.297130479	0.273	0.198	0.322305309	6	Ssr4
2.75E-05	0.300408526	0.362	0.288	0.363303614	6	Nap1l1
3.78E-05	0.276598146	0.354	0.273	0.498535762	6	Slpi
3.82E-05	0.317780715	0.254	0.184	0.50377469	6	Thoc7
4.42E-05	-0.391102806	0.547	0.591	0.583221881	6	Hmgn2
5.97E-05	0.265073894	0.369	0.293	0.786928098	6	Ndufc1
6.71E-05	0.281257596	0.295	0.221	0.88571243	6	Cxcr4
6.88E-05	0.305738764	0.351	0.281	0.907622353	6	Eif4a1
8.05E-05	0.293730568	0.384	0.306	1	6	Arf5
8.28E-05	0.289771361	0.399	0.327	1	6	Lsm5
8.96E-05	0.333815826	0.262	0.199	1	6	Supt16
9.43E-05	-0.31677105	0.334	0.24	1	6	Elane
0.00010741	0.317854847	0.286	0.22	1	6	Rpgrip1
0.00011854	0.301855031	0.321	0.251	1	6	Wtap
0.00012508	0.290150901	0.321	0.252	1	6	Mtpn
0.00013201	0.306354684	0.338	0.272	1	6	Rac1
0.00013749	0.264492768	0.536	0.486	1	6	Arpc2
0.00025165	0.321065227	0.26	0.2	1	6	Cmpk1
0.00025904	0.310937142	0.334	0.277	1	6	Hnrnpab
0.00030864	0.271339857	0.358	0.298	1	6	Ndufb11
0.00034563	0.252051254	0.28	0.216	1	6	Gm10250
0.00045756	0.295492165	0.26	0.202	1	6	Ndufa5
0.00053545	0.30925888	0.273	0.215	1	6	Ssr1
0.00068096	0.268259094	0.299	0.242	1	6	S100a13
0.00085011	0.282308109	0.43	0.375	1	6	Lbr
0.00089961	0.284471238	0.349	0.297	1	6	Tpm3
0.00090743	0.271276939	0.26	0.204	1	6	Hnrnpd
0.00104167	0.272689792	0.312	0.261	1	6	Snrpb
0.00108862	0.276904537	0.323	0.269	1	6	Atp5o

0.00125832	0.271321758	0.336	0.285	1	6	Ywhah	
0.00126089	0.283341289	0.338	0.28	1	6	Nsa2	
0.00134383	0.251777857	0.275	0.222	1	6	Fam111a	
0.00141518	0.252304492	0.273	0.217	1	6	Ndufa11	
0.00197061	0.288071758	0.28	0.226	1	6	Sptssa	
0.00345879	-0.339357316	0.625	0.644	1	6	Fth1	
0.00404581	0.258342706	0.291	0.247	1	6	Psmb3	
0.00431709	-0.89253415	0.423	0.481	1	6	Retnlg	
0.00449911	0.294882184	0.258	0.214	1	6	Birc6	
0.00472777	0.273718461	0.258	0.211	1	6	Rad21	
0.00539664	0.261345344	0.28	0.235	1	6	Ptges3	
0.00977093	0.252963513	0.262	0.218	1	6	Sf3b6	
5.72E-201	1.430937501	0.407	0.039	7.54E-197	7	Sox4	**Mac?**
6.61E-166	-1.183805715	0.968	0.998	8.72E-162	7	Tmsb4x	
4.10E-137	1.322007412	0.434	0.072	5.41E-133	7	Ccnd2	
1.44E-121	1.372204521	0.544	0.132	1.91E-117	7	Cdk6	
2.05E-115	0.829047106	0.983	0.751	2.70E-111	7	Rpl36	
1.07E-108	0.701124195	0.995	0.89	1.41E-104	7	Rps19	
1.39E-105	0.7342617	0.993	0.859	1.83E-101	7	Rpl32	
2.21E-103	-1.784055486	0.73	0.918	2.91E-99	7	Lyz2	
2.06E-101	-1.447614108	0.431	0.841	2.71E-97	7	Tyrobp	
2.87E-101	0.916221315	0.936	0.617	3.79E-97	7	Xist	
1.46E-99	0.727053274	0.995	0.825	1.92E-95	7	Rps18	
3.78E-97	0.687443035	0.995	0.841	4.99E-93	7	Rpl13	
4.70E-95	1.142810885	0.27	0.037	6.20E-91	7	Ifitm1	
7.76E-95	0.667881799	0.995	0.874	1.02E-90	7	Rps14	
1.49E-93	0.667288326	0.99	0.866	1.97E-89	7	Rpl18a	
5.27E-93	0.643290936	0.99	0.849	6.95E-89	7	Rpl35	
1.32E-90	0.788964561	0.966	0.667	1.75E-86	7	Rpl14	
1.52E-88	0.603899339	0.995	0.896	2.01E-84	7	Rpl37	
1.53E-87	-1.494418645	0.333	0.757	2.02E-83	7	Cd52	

1.72E-86	0.66537477	0.98	0.826	2.27E-82	7	Rps8
6.77E-86	0.720896742	0.988	0.771	8.93E-82	7	Rps5
2.38E-85	0.563751562	0.998	0.895	3.14E-81	7	Rps28
2.57E-85	0.694198441	0.968	0.759	3.39E-81	7	Rps3a1
5.89E-85	0.743124906	0.971	0.709	7.77E-81	7	Rps4x
1.07E-80	0.747098709	0.949	0.624	1.41E-76	7	Rpl36a
8.63E-80	0.688214308	0.973	0.732	1.14E-75	7	Rps6
1.89E-76	0.505389768	1	0.934	2.49E-72	7	Rpl37a
1.96E-76	0.645465797	0.98	0.763	2.59E-72	7	Rpl39
1.63E-74	0.644578214	0.961	0.755	2.15E-70	7	Rpl21
3.38E-73	0.615560362	0.968	0.794	4.46E-69	7	Rps24
1.60E-72	0.614555553	0.973	0.768	2.11E-68	7	Rpl26
5.47E-72	0.670946168	0.963	0.726	7.21E-68	7	Rpl23
5.63E-72	0.700419914	0.953	0.674	7.43E-68	7	Rps7
2.63E-71	-1.243495123	0.348	0.739	3.47E-67	7	Fcer1g
8.90E-71	0.659536422	0.961	0.749	1.17E-66	7	Rpl17
2.48E-70	0.672996268	0.953	0.731	3.27E-66	7	Rpl9
6.59E-69	0.849439965	0.811	0.462	8.69E-65	7	Rpl12
1.01E-68	0.656395363	0.946	0.666	1.33E-64	7	Rpsa
1.25E-68	0.573745864	0.973	0.797	1.65E-64	7	Rpl13a
2.43E-68	-1.511091338	0.223	0.641	3.21E-64	7	Ly6c2
2.58E-66	-1.220241747	0.331	0.708	3.40E-62	7	Cyba
7.05E-65	0.628596403	0.956	0.748	9.30E-61	7	Rps3
3.57E-64	1.048151278	0.414	0.125	4.70E-60	7	Lmo2
5.78E-64	0.858068443	0.728	0.36	7.63E-60	7	Npm1
5.84E-64	-1.736769476	0.088	0.529	7.70E-60	7	Cybb
1.09E-63	0.649918024	0.946	0.657	1.44E-59	7	Gm2000
1.92E-63	0.72698117	0.877	0.533	2.54E-59	7	Rps2
1.07E-62	0.939131546	0.382	0.104	1.41E-58	7	Mef2c
1.15E-62	0.622313858	0.953	0.706	1.52E-58	7	Rpl6
1.65E-62	0.754932128	0.88	0.555	2.17E-58	7	Rpl10a

3.65E-61	0.557427015	0.975	0.8	4.82E-57	7	Rps23
8.58E-61	1.046512287	0.466	0.162	1.13E-56	7	Myb
6.26E-60	0.660091338	0.917	0.645	8.26E-56	7	Rps20
2.13E-59	0.919548951	0.542	0.211	2.81E-55	7	Mif
6.87E-59	0.593312399	0.953	0.705	9.06E-55	7	Rpl27a
6.92E-59	1.025773948	0.654	0.324	9.13E-55	7	H2afy
1.10E-58	0.70362823	0.877	0.581	1.45E-54	7	Rpl3
6.24E-58	0.59693881	0.922	0.703	8.23E-54	7	Rpl11
8.15E-58	0.521982627	0.968	0.841	1.08E-53	7	Rpl38
5.01E-57	0.581734945	0.941	0.732	6.61E-53	7	Rps15
1.22E-56	0.593115053	0.958	0.735	1.61E-52	7	Rps15a
2.70E-55	0.679494405	0.863	0.56	3.56E-51	7	Rpl15
3.50E-55	0.558903514	0.963	0.708	4.62E-51	7	Rps26
5.67E-55	0.562503544	0.953	0.707	7.48E-51	7	Rplp1
3.89E-54	0.65622527	0.895	0.643	5.13E-50	7	Eef1a1
6.05E-54	0.58695094	0.917	0.642	7.98E-50	7	Rps12
1.84E-53	0.762758903	0.725	0.382	2.43E-49	7	Rpl36-ps3
5.00E-53	0.910844116	0.453	0.166	6.60E-49	7	Erdr1
6.58E-53	0.52051545	0.961	0.809	8.68E-49	7	Rpl23a
7.91E-53	0.815411595	0.596	0.263	1.04E-48	7	Eef1g
2.99E-52	0.498786861	0.968	0.796	3.94E-48	7	Rps16
2.45E-50	-1.10463494	0.348	0.665	3.23E-46	7	Prdx5
2.99E-50	0.739247512	0.745	0.413	3.94E-46	7	Gm9493
6.74E-50	0.392582665	1	0.953	8.90E-46	7	Rps27
1.08E-49	-1.740757153	0.252	0.573	1.42E-45	7	Chil3
3.01E-49	0.705686717	0.73	0.398	3.97E-45	7	Hsp90ab1
1.79E-48	0.544870447	0.917	0.696	2.36E-44	7	Rpl28
5.35E-48	0.514181743	0.953	0.766	7.06E-44	7	Rplp2
9.61E-48	-1.600342955	0.221	0.553	1.27E-43	7	S100a6
1.98E-47	0.999790635	0.375	0.126	2.61E-43	7	Pim1
4.21E-47	0.853978086	0.532	0.236	5.55E-43	7	Ncl

5.30E-47	0.583547975	0.875	0.628	6.99E-43	7	Rpl18
5.38E-47	0.654936087	0.772	0.45	7.10E-43	7	Rps18-ps3
8.12E-47	0.573052681	0.909	0.629	1.07E-42	7	Gm10263
1.65E-46	0.518049441	0.936	0.732	2.18E-42	7	Rpl24
2.38E-46	0.374717896	1	0.953	3.14E-42	7	Rpl41
2.54E-46	0.549030455	0.929	0.696	3.35E-42	7	Rplp0
2.88E-45	0.641720204	0.799	0.485	3.80E-41	7	Rpl22l1
1.16E-44	0.518697068	0.924	0.707	1.53E-40	7	Rps17
4.36E-43	-1.143029803	0.294	0.601	5.75E-39	7	S100a11
6.37E-43	0.338257318	0.995	0.975	8.41E-39	7	Rps29
2.15E-42	0.923150584	0.571	0.276	2.83E-38	7	Pdcd4
3.06E-42	-1.696644552	0.145	0.468	4.03E-38	7	Pglyrp1
4.78E-42	0.411052492	0.971	0.866	6.31E-38	7	Rpl35a
1.37E-41	0.460917255	0.951	0.784	1.80E-37	7	Rps11
3.60E-41	0.59304254	0.841	0.574	4.75E-37	7	Rpl4
1.76E-40	0.459141929	0.953	0.795	2.32E-36	7	Rpl34
2.73E-40	-2.029664668	0.998	0.994	3.61E-36	7	S100a9
3.80E-40	0.5518252	0.865	0.572	5.02E-36	7	Wdr89
8.91E-40	0.483584793	0.909	0.709	1.18E-35	7	Rpl19
9.78E-40	-1.859665899	0.429	0.644	1.29E-35	7	Wfdc21
1.19E-39	0.602155258	0.757	0.437	1.57E-35	7	Rps12-ps3
1.44E-39	-1.248735283	0.154	0.475	1.90E-35	7	Hp
1.44E-39	0.514114307	0.895	0.678	1.90E-35	7	Rps13
3.87E-39	0.679299934	0.282	0.083	5.10E-35	7	Ptprcap
6.17E-39	0.42210887	0.971	0.866	8.14E-35	7	Rps9
1.53E-38	0.678883042	0.605	0.317	2.02E-34	7	Hnrnpa1
2.40E-38	0.388013875	0.975	0.87	3.17E-34	7	Rps27a
4.86E-38	-1.947599407	0.922	0.932	6.41E-34	7	Camp
9.91E-38	-2.04484054	0.772	0.855	1.31E-33	7	Ngp
1.80E-37	0.508858288	0.895	0.656	2.38E-33	7	Rpl23a-ps3
2.23E-37	0.652240491	0.694	0.417	2.94E-33	7	Rpl13-ps3

2.98E-37	-1.945111295	1	0.999	3.93E-33	7	S100a8
7.23E-37	0.405149875	0.975	0.835	9.54E-33	7	Rps27rt
1.12E-36	0.684611142	0.603	0.324	1.48E-32	7	Set
1.42E-36	0.792015978	0.314	0.111	1.87E-32	7	Pebp1
1.45E-36	-0.636118356	0.623	0.791	1.91E-32	7	Shfm1
3.14E-36	0.484328492	0.88	0.632	4.14E-32	7	Rpl8
3.32E-36	0.613375839	0.716	0.43	4.39E-32	7	Rpl9-ps6
5.48E-36	-1.808296332	0.333	0.573	7.23E-32	7	Lcn2
2.26E-35	0.47547953	0.882	0.666	2.98E-31	7	Rpl27
2.86E-35	-0.794184521	0.998	0.999	3.78E-31	7	Actb
3.09E-35	-1.321014577	0.164	0.457	4.07E-31	7	Lgals3
5.53E-35	0.576289747	0.713	0.421	7.30E-31	7	Gm10073
1.01E-34	0.61773752	0.441	0.185	1.34E-30	7	Srgn
1.06E-34	0.573152377	0.775	0.509	1.40E-30	7	Rpl22
1.12E-34	0.639774427	0.694	0.422	1.48E-30	7	Gnb2l1
6.31E-34	0.542773805	0.75	0.457	8.33E-30	7	Rps26-ps1
6.61E-34	-0.547053409	0.684	0.846	8.72E-30	7	Pfn1
2.09E-33	-0.782817024	0.466	0.678	2.76E-29	7	Arhgdib
3.30E-32	0.285415238	1	0.997	4.35E-28	7	Malat1
3.46E-32	-1.245385598	0.167	0.447	4.57E-28	7	Anxa1
1.15E-31	-1.052171682	0.23	0.495	1.52E-27	7	F630028O10Rik
3.54E-31	0.640264812	0.578	0.32	4.67E-27	7	Nop10
5.30E-31	0.618819248	0.738	0.496	6.99E-27	7	Pabpc1
8.06E-31	0.379948515	0.926	0.742	1.06E-26	7	Gm10076
1.25E-30	0.667707856	0.272	0.094	1.65E-26	7	Tia1
1.41E-30	0.739954939	0.289	0.106	1.86E-26	7	Hmgn1
1.64E-30	0.575671346	0.708	0.453	2.17E-26	7	Gm8730
2.49E-30	0.573668855	0.748	0.492	3.28E-26	7	Cbx3
2.81E-30	-1.448539437	0.105	0.377	3.70E-26	7	Ifitm6
5.83E-30	0.463750878	0.855	0.644	7.69E-26	7	Rps10
8.77E-29	-0.626294662	0.735	0.826	1.16E-24	7	Ftl1

9.07E-29	-1.576456726	0.35	0.554	1.20E-24	7	Ltf
1.51E-28	0.341899449	0.971	0.834	1.99E-24	7	Ptma
2.13E-28	0.60118041	0.549	0.302	2.81E-24	7	Eef1b2
3.06E-28	0.585520451	0.657	0.412	4.04E-24	7	Serbp1
3.97E-28	0.604191018	0.576	0.336	5.24E-24	7	Rpl6l
5.39E-28	-0.932859465	0.436	0.645	7.12E-24	7	Cst3
1.21E-27	-1.063802508	0.343	0.557	1.59E-23	7	Psap
2.76E-27	0.367361768	0.895	0.696	3.64E-23	7	Rpl31
2.07E-26	0.522441351	0.696	0.455	2.73E-22	7	Rpl29
2.59E-26	-0.63893519	0.676	0.786	3.42E-22	7	Hmgb2
3.79E-26	0.593213345	0.439	0.215	4.99E-22	7	Hspe1
1.72E-25	0.795075743	0.275	0.11	2.27E-21	7	Vamp5
2.09E-25	0.50506263	0.745	0.534	2.76E-21	7	Rpl7
3.06E-25	0.381066331	0.907	0.716	4.04E-21	7	Rps25
4.55E-25	0.37797108	0.912	0.681	6.00E-21	7	Rpl10
5.10E-25	-0.97564297	0.123	0.369	6.73E-21	7	Msrb1
1.09E-24	0.420038039	0.821	0.594	1.44E-20	7	Rps21
1.32E-24	-0.825413283	0.221	0.467	1.75E-20	7	Alox5ap
2.68E-24	-1.115442286	0.054	0.28	3.53E-20	7	Clec4a2
7.60E-24	-0.578587943	0.527	0.704	1.00E-19	7	Sh3bgrl3
4.14E-23	0.595895646	0.377	0.183	5.46E-19	7	St13
4.37E-23	-0.889125343	0.142	0.381	5.76E-19	7	Gsr
8.91E-23	0.479803638	0.65	0.412	1.18E-18	7	Eef2
9.20E-23	0.487173137	0.581	0.342	1.21E-18	7	Snrpf
9.61E-23	-1.011851904	0.078	0.305	1.27E-18	7	Mgst1
1.33E-22	0.47520855	0.654	0.416	1.76E-18	7	Rpl5
1.59E-22	0.475384985	0.652	0.438	2.09E-18	7	Naca
1.83E-22	0.53004418	0.311	0.134	2.41E-18	7	Bcl11a
2.45E-22	-1.170601299	0.113	0.331	3.23E-18	7	Ms4a6c
4.01E-22	-1.232961732	0.074	0.288	5.29E-18	7	Ccr2
4.23E-22	0.619235519	0.279	0.119	5.58E-18	7	Rpl13a-ps1

4.93E-22	0.474221932	0.679	0.45	6.50E-18	7	Rpl30
6.73E-22	0.579374003	0.434	0.229	8.87E-18	7	Zfp36l2
1.24E-21	-0.854512277	0.186	0.41	1.64E-17	7	Dstn
1.65E-21	0.507196015	0.654	0.437	2.18E-17	7	Atpif1
1.96E-21	0.600685055	0.593	0.401	2.58E-17	7	Stmn1
4.66E-21	0.315428592	0.895	0.691	6.14E-17	7	mt-Cytb
8.61E-21	0.426024798	0.689	0.46	1.14E-16	7	Rpl27-ps3
9.18E-21	0.539580639	0.471	0.271	1.21E-16	7	Gm10269
1.18E-20	0.611111468	0.375	0.192	1.56E-16	7	Pan3
2.35E-20	0.617145852	0.279	0.124	3.10E-16	7	Zfos1
2.39E-20	0.534732598	0.507	0.309	3.16E-16	7	Gm10036
2.94E-20	0.506737721	0.525	0.312	3.88E-16	7	Hsp90aa1
5.97E-19	-0.64688798	0.338	0.54	7.88E-15	7	Coro1a
8.96E-19	0.537904626	0.326	0.16	1.18E-14	7	Ddx21
9.48E-19	0.528458267	0.453	0.265	1.25E-14	7	Arglu1
1.68E-18	0.565946074	0.547	0.361	2.21E-14	7	Srrm2
3.23E-18	-0.955943004	0.066	0.252	4.26E-14	7	Slfn2
3.95E-18	-0.511689462	0.493	0.659	5.21E-14	7	Itm2b
4.99E-18	0.388314648	0.721	0.506	6.59E-14	7	Snrpg
6.01E-18	-1.28338081	0.184	0.371	7.93E-14	7	Ifi27l2a
6.23E-18	-0.894123929	0.086	0.275	8.22E-14	7	Ncf1
7.49E-18	-0.914717077	0.113	0.306	9.88E-14	7	Sat1
1.19E-17	0.542871327	0.277	0.129	1.57E-13	7	Paip2
1.61E-17	0.380037264	0.694	0.486	2.12E-13	7	Rpl7a
1.91E-17	0.519734882	0.341	0.177	2.52E-13	7	Pnisr
6.80E-17	-0.648761637	0.284	0.476	8.97E-13	7	Rac2
8.14E-17	-0.852523011	0.142	0.328	1.07E-12	7	Lsp1
1.06E-16	0.435210242	0.478	0.292	1.39E-12	7	mt-Nd2
1.91E-16	0.377223266	0.772	0.6	2.52E-12	7	Gm11808
3.44E-16	0.532248104	0.301	0.154	4.53E-12	7	Fubp1
3.56E-16	-0.789816412	0.098	0.278	4.70E-12	7	Cotl1

3.72E-16	0.551530866	0.292	0.147	4.90E-12	7	Hnrnpa0
6.03E-16	0.537498365	0.306	0.161	7.95E-12	7	Cbx1
6.33E-16	0.47798034	0.453	0.273	8.35E-12	7	Cmtm7
6.59E-16	-0.71277909	0.154	0.344	8.69E-12	7	Clec12a
6.92E-16	0.549499859	0.275	0.136	9.13E-12	7	Mat2a
7.31E-16	-0.861679186	0.093	0.268	9.64E-12	7	Cebpb
7.81E-16	0.452454302	0.473	0.29	1.03E-11	7	Erh
1.23E-15	-0.378801096	0.679	0.786	1.63E-11	7	Serf2
1.45E-15	-0.882361555	0.088	0.261	1.91E-11	7	Tmcc1
2.29E-15	0.450056656	0.297	0.15	3.02E-11	7	Tardbp
2.57E-15	0.340841973	0.787	0.621	3.40E-11	7	Uba52
2.68E-15	0.511753736	0.36	0.201	3.54E-11	7	Cox7a2l
9.26E-15	0.493759145	0.279	0.142	1.22E-10	7	Cnbp
9.83E-15	0.490372414	0.304	0.16	1.30E-10	7	Tomm20
1.16E-14	-0.445457969	0.578	0.694	1.53E-10	7	Actg1
1.42E-14	-0.766766135	0.098	0.267	1.88E-10	7	Anxa2
1.68E-14	0.541288634	0.292	0.156	2.22E-10	7	G3bp1
2.15E-14	0.44033756	0.299	0.154	2.84E-10	7	Sepw1
5.13E-14	-0.827520368	0.125	0.289	6.76E-10	7	Slpi
7.02E-14	0.452394164	0.333	0.183	9.26E-10	7	Ogt
1.21E-13	-0.630500017	0.243	0.409	1.59E-09	7	Actr3
1.43E-13	0.517315133	0.311	0.174	1.88E-09	7	Gm6133
2.16E-13	-0.662941503	0.172	0.339	2.85E-09	7	Cd53
3.09E-13	-0.713065437	0.208	0.375	4.08E-09	7	Lst1
3.48E-13	-0.404667085	0.615	0.715	4.59E-09	7	Myl6
6.92E-13	0.466438587	0.304	0.169	9.13E-09	7	Lsm3
7.72E-13	0.383913745	0.574	0.418	1.02E-08	7	Sumo2
3.27E-12	0.496283674	0.265	0.142	4.31E-08	7	Chd4
3.29E-12	-0.688782923	0.137	0.287	4.33E-08	7	Reep5
3.41E-12	0.437257065	0.267	0.141	4.50E-08	7	Atxn7l3b
4.25E-12	0.419127274	0.502	0.356	5.61E-08	7	Ran

4.59E-12	0.440113564	0.262	0.139	6.05E-08	7	Prdx2
4.63E-12	0.409318731	0.468	0.315	6.11E-08	7	Ranbp1
4.67E-12	-0.498616868	0.35	0.499	6.16E-08	7	Gnai2
1.30E-11	0.456668372	0.441	0.296	1.72E-07	7	Tsix
1.35E-11	0.313345013	0.635	0.455	1.78E-07	7	Rpl10-ps3
1.42E-11	0.411583178	0.368	0.227	1.88E-07	7	Metap2
1.44E-11	0.36055985	0.517	0.363	1.90E-07	7	Srsf2
1.50E-11	-0.675350542	0.123	0.265	1.97E-07	7	Flna
2.34E-11	0.407988079	0.593	0.464	3.09E-07	7	Ndufa4
2.89E-11	0.503136009	0.304	0.179	3.81E-07	7	Hp1bp3
3.70E-11	-0.642305092	0.123	0.265	4.88E-07	7	Vim
4.21E-11	-0.625676978	0.529	0.591	5.56E-07	7	Hmgn2
5.14E-11	-0.348629783	0.669	0.75	6.77E-07	7	Gpx1
5.47E-11	0.364801587	0.444	0.295	7.22E-07	7	Hnrnpm
5.49E-11	-0.344935292	0.625	0.721	7.24E-07	7	Cfl1
5.53E-11	-0.878835731	0.48	0.564	7.30E-07	7	Ifitm3
5.79E-11	0.37285354	0.321	0.191	7.64E-07	7	Gm10020
5.86E-11	0.321166213	0.5	0.338	7.72E-07	7	Ptpn18
8.33E-11	-0.555913122	0.186	0.333	1.10E-06	7	Scp2
9.67E-11	0.346520859	0.297	0.169	1.28E-06	7	Eif3e
9.86E-11	0.421016857	0.27	0.154	1.30E-06	7	Banf1
1.11E-10	-0.521674526	0.221	0.364	1.47E-06	7	Fam49b
1.14E-10	0.342625894	0.637	0.488	1.50E-06	7	Tubb5
1.16E-10	0.413895634	0.382	0.245	1.53E-06	7	Eif3a
1.18E-10	0.38881706	0.363	0.23	1.56E-06	7	Ptges3
2.23E-10	0.400511577	0.257	0.145	2.94E-06	7	Hnrnpr
2.47E-10	0.441877391	0.297	0.18	3.26E-06	7	Srsf6
2.92E-10	0.341369291	0.517	0.367	3.85E-06	7	Btf3
3.03E-10	0.315003984	0.544	0.382	4.00E-06	7	Gm8186
5.98E-10	0.316962317	0.468	0.316	7.89E-06	7	Eif3f
8.76E-10	0.335686729	0.377	0.24	1.16E-05	7	Rbm25

8.81E-10	-0.615390443	0.13	0.259	1.16E-05	7	Ostf1
1.05E-09	-0.424004242	0.422	0.544	1.38E-05	7	Mrpl33
1.08E-09	0.352854521	0.277	0.16	1.42E-05	7	Mtdh
1.74E-09	0.399998984	0.314	0.196	2.29E-05	7	Srsf7
2.16E-09	0.252078039	0.5	0.348	2.86E-05	7	H2-K1
2.93E-09	0.343906511	0.417	0.285	3.87E-05	7	Nap1l1
3.49E-09	-0.967871933	0.645	0.636	4.60E-05	7	Crip1
3.61E-09	0.332906125	0.358	0.231	4.76E-05	7	Zfp207
4.29E-09	-0.361414992	0.451	0.562	5.66E-05	7	Calm1
4.80E-09	-0.520969512	0.26	0.379	6.34E-05	7	Arpc5
4.86E-09	-0.524560473	0.176	0.305	6.41E-05	7	Cdk2ap2
6.11E-09	-0.564855697	0.211	0.342	8.06E-05	7	2810417H13Rik
7.12E-09	0.412662506	0.346	0.23	9.39E-05	7	Park7
7.74E-09	0.366917891	0.319	0.203	0.000102068	7	Fus
1.00E-08	-0.523511508	0.145	0.266	0.000132388	7	Aprt
1.10E-08	0.300219382	0.561	0.425	0.000145077	7	Ybx1
1.27E-08	0.366629855	0.267	0.163	0.000167413	7	Prpf4b
1.50E-08	-0.475231991	0.184	0.313	0.000198196	7	Taldo1
1.53E-08	-0.434064112	0.331	0.452	0.000201719	7	Arpc1b
1.73E-08	0.351191751	0.333	0.215	0.000228678	7	Gltscr2
1.74E-08	-1.34274627	0.145	0.254	0.000229315	7	Elane
1.93E-08	0.303764305	0.441	0.306	0.000254735	7	Atp5g1
2.23E-08	0.387255168	0.412	0.291	0.000294255	7	Atp5g2
2.28E-08	0.343179827	0.267	0.162	0.000300304	7	Ddx17
2.70E-08	0.421199721	0.292	0.188	0.000355742	7	Ywhaq
3.28E-08	0.318470314	0.39	0.265	0.000432869	7	Ywhae
3.31E-08	-0.612632444	0.404	0.482	0.000436836	7	Lgals1
6.96E-08	0.325281169	0.348	0.233	0.000918428	7	Eif5b
7.98E-08	0.272376855	0.529	0.4	0.001052096	7	Rbm39
8.29E-08	-0.40276802	0.336	0.449	0.001093853	7	Actr2
8.49E-08	0.286060404	0.458	0.33	0.001120202	7	Srsf3

8.62E-08	0.380114148	0.267	0.168	0.001137674	7	Prpf38b
1.28E-07	-0.461909877	0.321	0.424	0.001683469	7	Cdc42
1.34E-07	0.344192413	0.26	0.162	0.001761766	7	Srsf1
1.51E-07	-0.437670367	0.174	0.291	0.001996911	7	Cd44
1.64E-07	-0.511265828	0.172	0.281	0.002157425	7	Aldh2
2.20E-07	0.28707603	0.304	0.197	0.002900883	7	Zfp706
2.41E-07	0.284957315	0.284	0.177	0.003181902	7	Mrpl52
2.50E-07	0.332769248	0.382	0.27	0.003300442	7	Bola2
2.81E-07	-0.407469326	0.333	0.438	0.003711453	7	Tspo
3.03E-07	-0.472738473	0.211	0.323	0.0040019	7	Ptprc
3.07E-07	-0.486159496	0.262	0.376	0.00405079	7	Myh9
3.21E-07	-0.447715693	0.284	0.391	0.004238582	7	1810037I17Rik
3.35E-07	0.295717948	0.297	0.192	0.00442466	7	AI662270
4.08E-07	-0.354504269	0.375	0.481	0.005376322	7	H2afj
4.27E-07	0.275725369	0.52	0.388	0.005628916	7	mt-Atp8
4.35E-07	-0.471782603	0.267	0.37	0.005744018	7	Serp1
4.39E-07	-0.333445197	0.468	0.538	0.005796402	7	Atp5k
4.67E-07	-0.355809889	0.397	0.496	0.006163251	7	Arpc2
4.68E-07	0.347778039	0.26	0.167	0.006171516	7	Matr3
5.48E-07	-0.526828926	0.164	0.264	0.007228262	7	Gm10282
6.95E-07	0.298205252	0.382	0.274	0.00916342	7	Hnrnpab
7.26E-07	0.377193005	0.275	0.182	0.009582178	7	Ramp1
9.30E-07	-0.433091312	0.395	0.474	0.012263077	7	Ly6e
9.70E-07	-0.381974254	0.223	0.333	0.012794519	7	Lamtor4
1.03E-06	-0.435786325	0.152	0.253	0.013590095	7	S100a13
1.17E-06	0.294534655	0.319	0.215	0.015379492	7	Etfb
1.28E-06	0.292334848	0.26	0.169	0.01688959	7	Uqcc2
1.32E-06	0.302898805	0.38	0.276	0.01738003	7	Tra2b
1.97E-06	0.26105199	0.27	0.176	0.025974199	7	Trmt112
2.61E-06	0.312657585	0.397	0.294	0.034453188	7	Sfpq
3.19E-06	0.295460269	0.299	0.206	0.042032506	7	Hnrnpc

3.22E-06	-0.316485136	0.164	0.264	0.042525642	7	Lmo4
3.45E-06	0.32222546	0.316	0.224	0.045470787	7	Sptssa
3.47E-06	-0.405575829	0.341	0.429	0.045806306	7	Tkt
3.59E-06	0.253508206	0.309	0.209	0.047337362	7	Pdap1
5.10E-06	-0.37625326	0.275	0.374	0.067241837	7	Capza2
6.52E-06	0.331397599	0.252	0.171	0.085964315	7	Mrpl23
6.70E-06	0.36123948	0.265	0.182	0.088356818	7	Skp1a
7.46E-06	-0.446160703	0.172	0.264	0.098411995	7	Ucp2
7.54E-06	0.262766354	0.326	0.23	0.099522758	7	Dpm3
8.02E-06	0.343957649	0.301	0.215	0.105754772	7	Top1
9.87E-06	-0.473263887	0.208	0.302	0.13022316	7	Cd24a
1.44E-05	-0.292618019	0.596	0.64	0.190165349	7	Sec61g
1.57E-05	-0.32135583	0.289	0.385	0.206683642	7	Lbr
1.63E-05	0.273531045	0.35	0.257	0.214888629	7	Prrc2c
1.65E-05	0.260397072	0.353	0.256	0.217793433	7	Snrpd2
1.85E-05	0.337555046	0.252	0.175	0.244204659	7	Dnmt1
2.09E-05	-0.329847938	0.228	0.324	0.275298037	7	Capza1
2.53E-05	0.30775831	0.275	0.193	0.333508922	7	Caprin1
2.63E-05	0.325248876	0.267	0.187	0.346686867	7	Hnrnpl
2.64E-05	-0.437014604	0.24	0.324	0.348406411	7	Gm10116
2.71E-05	-0.307821941	0.517	0.581	0.357728603	7	H2-D1
3.29E-05	-0.356749051	0.243	0.333	0.433874507	7	Ap3s1
3.49E-05	-0.291134665	0.279	0.375	0.460978451	7	Myeov2
3.91E-05	-0.539817719	0.186	0.262	0.516130267	7	Gm9844
4.04E-05	-0.349588544	0.338	0.416	0.533496399	7	Atox1
4.24E-05	-0.26707703	0.502	0.562	0.558825453	7	Ndufa3
4.49E-05	-0.344460773	0.189	0.276	0.591693152	7	Aldoa
5.11E-05	-0.308421902	0.275	0.37	0.674505787	7	Arpc3
5.60E-05	-0.309713507	0.309	0.396	0.738927511	7	Smdt1
6.16E-05	-0.396014272	0.174	0.26	0.81306143	7	Btg1
9.49E-05	0.256425175	0.257	0.184	1	7	Eif3j1

0.00010428	-0.364015523	0.225	0.307	1	7	Myl12a	
0.00011064	0.295031799	0.252	0.178	1	7	Rsrc2	
0.00011401	0.283646046	0.321	0.242	1	7	Hnrnph1	
0.00011556	-0.297014875	0.27	0.359	1	7	Atp6v0e	
0.00011936	-0.339096357	0.174	0.254	1	7	Sec11c	
0.00012629	-0.370411217	0.297	0.37	1	7	Emp3	
0.00013532	-0.307769349	0.331	0.409	1	7	Ndufa13	
0.00015928	-0.310696724	0.189	0.273	1	7	Fis1	
0.00016597	0.288330536	0.284	0.211	1	7	Atrx	
0.00020856	-0.319518409	0.211	0.292	1	7	Gnb2	
0.00023573	-0.282434659	0.252	0.337	1	7	Lsm5	
0.00032199	-0.312327049	0.282	0.354	1	7	Ndufa1	
0.00072876	-0.357272149	0.26	0.326	1	7	Npc2	
0.0008521	-0.345684454	0.221	0.283	1	7	Lcp1	
0.00087328	-0.316765843	0.216	0.282	1	7	Iqgap1	
0.00088374	-0.326774333	0.233	0.296	1	7	Pkm	
0.00113649	-0.258768663	0.292	0.367	1	7	BC005537	
0.00160427	0.349552286	0.414	0.352	1	7	Gm42418	
0.00216771	-0.25680019	0.206	0.273	1	7	Gpi1	
0.0023148	-0.270152984	0.257	0.318	1	7	Mrps21	
0.00332204	-0.273156906	0.25	0.307	1	7	Uqcrb	
0.00406716	-0.831515638	0.431	0.479	1	7	Retnlg	
0.00905264	-0.258819911	0.245	0.296	1	7	Ppp1cb	
8.24E-305	2.252947419	0.967	0.23	1.09E-300	8	Prtn3	**Mac**
8.04E-298	2.675979809	0.919	0.206	1.06E-293	8	Elane	
1.51E-289	2.309410553	0.943	0.229	2.00E-285	8	Mpo	
1.77E-222	1.799248537	0.71	0.128	2.34E-218	8	Ms4a3	
3.69E-217	1.817151168	0.694	0.122	4.86E-213	8	Ctsg	
4.21E-162	1.473177713	0.547	0.092	5.55E-158	8	Mt1	
8.65E-139	1.312118936	0.591	0.127	1.14E-134	8	Nkg7	
1.30E-128	1.431644249	0.466	0.082	1.71E-124	8	Hyou1	

5.53E-128	1.291485584	0.509	0.099	7.29E-124	8	Gstm1
6.93E-126	0.625142341	1	0.974	9.14E-122	8	Rps29
2.70E-120	1.324779048	0.64	0.175	3.57E-116	8	Srgn
5.21E-99	0.609330168	0.997	0.953	6.87E-95	8	Rpl41
6.96E-99	0.704811454	0.997	0.85	9.17E-95	8	Rpl35
1.04E-94	-1.787621995	0.772	0.914	1.37E-90	8	Lyz2
8.34E-87	0.60401423	1	0.895	1.10E-82	8	Rps28
1.19E-83	0.802730138	0.954	0.625	1.57E-79	8	Rpl36a
3.06E-82	1.101617468	0.39	0.085	4.04E-78	8	Rgcc
8.09E-82	1.11046957	0.501	0.138	1.07E-77	8	Sdf2l1
1.82E-80	1.088363326	0.623	0.221	2.40E-76	8	Calr
7.91E-79	0.619735643	0.997	0.833	1.04E-74	8	Ptma
1.64E-78	0.542491253	0.997	0.935	2.16E-74	8	Rpl37a
8.39E-77	0.893663183	0.282	0.047	1.11E-72	8	B4galt6
6.10E-76	0.564002812	0.997	0.89	8.05E-72	8	Rps19
2.21E-71	0.617431749	0.986	0.827	2.92E-67	8	Rps8
8.76E-69	1.122784532	0.656	0.272	1.16E-64	8	Pdcd4
4.87E-67	0.646071162	0.967	0.74	6.42E-63	8	Gm10076
2.63E-66	0.560548702	0.992	0.86	3.47E-62	8	Rpl32
6.43E-66	-1.301749668	0.412	0.749	8.48E-62	8	Cd52
4.90E-63	0.581742465	0.984	0.8	6.46E-59	8	Rps23
5.03E-62	0.603092493	0.976	0.764	6.63E-58	8	Rpl39
4.56E-61	0.623062687	0.954	0.668	6.01E-57	8	Plac8
1.14E-60	0.950695534	0.377	0.101	1.51E-56	8	Gatm
2.70E-59	0.638398531	0.949	0.641	3.56E-55	8	Rps12
3.77E-58	0.783855135	0.827	0.486	4.97E-54	8	Rpl22l1
2.58E-57	0.893568764	0.48	0.163	3.40E-53	8	Myb
1.20E-56	0.517321043	0.978	0.827	1.58E-52	8	Rps18
1.37E-56	0.939273473	0.656	0.307	1.81E-52	8	Ap3s1
2.40E-56	0.605631498	0.949	0.658	3.17E-52	8	Gm2000
3.30E-56	-1.139648542	0.404	0.733	4.36E-52	8	Fcer1g

7.37E-55	0.494534188	0.984	0.87	9.72E-51	8	Rps27a
4.27E-54	0.483833616	0.997	0.952	5.64E-50	8	mt-Co3
2.25E-53	0.571311885	0.962	0.734	2.97E-49	8	Rps6
2.58E-53	0.608568115	0.943	0.708	3.40E-49	8	Rps17
1.30E-52	0.478187622	0.986	0.897	1.71E-48	8	Rpl37
5.03E-52	0.56111393	0.94	0.715	6.63E-48	8	Rps25
2.66E-51	0.547093097	0.97	0.852	3.51E-47	8	mt-Atp6
8.07E-51	0.815739218	0.428	0.141	1.06E-46	8	Cdk6
4.04E-50	0.56658327	0.935	0.671	5.33E-46	8	Rpl14
1.68E-49	-0.474350604	1	0.996	2.22E-45	8	Tmsb4x
3.25E-49	0.801139121	0.26	0.06	4.29E-45	8	Alas1
6.69E-48	0.488069547	0.97	0.842	8.83E-44	8	Rpl38
2.43E-47	0.60171624	0.921	0.63	3.21E-43	8	Gm10263
5.10E-47	0.471346018	0.981	0.809	6.73E-43	8	Rpl23a
5.87E-47	0.445445883	0.986	0.865	7.75E-43	8	Rpl35a
1.61E-46	-1.707366751	0.23	0.579	2.12E-42	8	Ifitm3
3.13E-46	0.526024575	0.97	0.706	4.13E-42	8	Rpl6
5.16E-46	0.57375117	0.921	0.691	6.81E-42	8	mt-Cytb
6.95E-46	0.502959928	0.965	0.767	9.17E-42	8	Rplp2
2.65E-45	0.664229989	0.783	0.437	3.50E-41	8	Rps12-ps3
7.01E-45	0.501659001	0.954	0.754	9.25E-41	8	Rpl36
1.43E-44	0.870091156	0.293	0.081	1.89E-40	8	Igfbp4
2.14E-44	0.796374985	0.569	0.26	2.82E-40	8	Bola2
6.53E-44	-1.677477853	0.228	0.551	8.62E-40	8	S100a6
7.65E-43	-1.417127492	0.556	0.751	1.01E-38	8	Tmsb10
1.12E-42	0.771380995	0.52	0.215	1.48E-38	8	Mif
1.76E-42	0.594784594	0.808	0.489	2.32E-38	8	Sec61b
2.29E-42	0.845917836	0.488	0.206	3.03E-38	8	Etfb
7.04E-42	0.48227193	0.946	0.729	9.28E-38	8	Rpl23
7.54E-42	0.863535131	0.263	0.069	9.94E-38	8	Pam
9.90E-42	0.706503392	0.664	0.338	1.31E-37	8	Snrpf

2.29E-41	-1.317848883	0.179	0.521	3.02E-37	8	Cybb
2.69E-41	0.482236717	0.951	0.71	3.55E-37	8	Rps26
5.92E-41	0.834834455	0.401	0.146	7.80E-37	8	Kdelr2
7.95E-41	0.584036336	0.883	0.604	1.05E-36	8	mt-Nd1
1.27E-40	0.475075747	0.949	0.709	1.68E-36	8	Rplp1
6.87E-40	0.60347462	0.802	0.465	9.07E-36	8	Rpl12
2.22E-39	0.475972686	0.938	0.738	2.93E-35	8	Rps15a
3.96E-39	-0.827764986	0.683	0.823	5.23E-35	8	Tyrobp
5.70E-39	0.521976559	0.854	0.536	7.52E-35	8	Rps2
5.77E-38	0.490025422	0.916	0.678	7.61E-34	8	Rps7
9.21E-38	0.555590022	0.843	0.594	1.22E-33	8	Rps21
1.17E-37	-1.793381299	0.049	0.379	1.54E-33	8	Ifitm6
2.90E-36	0.68404988	0.629	0.317	3.83E-32	8	Hnrnpa1
6.15E-36	0.72456908	0.29	0.09	8.12E-32	8	Pdia4
8.67E-36	0.699471369	0.314	0.103	1.14E-31	8	Svip
2.33E-35	0.755942666	0.379	0.145	3.07E-31	8	Rpn1
2.83E-35	0.471966398	0.919	0.656	3.73E-31	8	Rpl23a-ps3
4.03E-35	0.439998951	0.959	0.77	5.31E-31	8	Rpl26
4.60E-35	0.536077953	0.818	0.508	6.07E-31	8	Rpl22
5.00E-35	0.710223867	0.623	0.324	6.59E-31	8	Set
8.36E-35	0.57521303	0.753	0.453	1.10E-30	8	Rps18-ps3
1.01E-34	0.65678936	0.61	0.297	1.33E-30	8	Atp5g1
1.29E-34	-1.963428382	0.339	0.571	1.70E-30	8	Lcn2
2.94E-34	0.52437174	0.864	0.574	3.87E-30	8	Wdr89
2.96E-34	-1.491042555	0.455	0.648	3.90E-30	8	Crip1
5.47E-34	0.553875499	0.729	0.416	7.21E-30	8	Gm9493
4.23E-33	0.662019934	0.61	0.32	5.58E-29	8	Nop10
5.91E-33	0.458381018	0.921	0.734	7.80E-29	8	Rpl24
6.62E-33	-1.362678925	0.314	0.567	8.73E-29	8	Chil3
8.12E-33	0.629030569	0.52	0.239	1.07E-28	8	Ncl
8.16E-33	0.451363525	0.919	0.697	1.08E-28	8	Rpl28

1.45E-32	0.416958032	0.924	0.696	1.92E-28	8	Rpl31
2.83E-32	0.403623738	0.94	0.752	3.73E-28	8	Rpl17
3.65E-32	0.40245387	0.938	0.797	4.81E-28	8	Rps24
4.48E-32	-2.001577512	0.832	0.85	5.91E-28	8	Ngp
9.44E-32	0.561073907	0.702	0.385	1.25E-27	8	Rpl36-ps3
1.64E-31	-1.313863454	0.168	0.455	2.17E-27	8	Lgals3
1.95E-31	-1.864356273	0.333	0.554	2.58E-27	8	Ltf
2.49E-31	0.571253852	0.602	0.308	3.28E-27	8	Ranbp1
4.71E-31	0.50536698	0.813	0.556	6.21E-27	8	mt-Nd4
1.44E-30	0.368607436	0.973	0.844	1.89E-26	8	Rpl13
2.18E-30	0.564616073	0.675	0.365	2.87E-26	8	Npm1
2.26E-30	0.695899171	0.374	0.153	2.98E-26	8	P4hb
3.17E-30	0.469950232	0.856	0.624	4.18E-26	8	Sec61g
6.92E-30	0.374573511	0.973	0.773	9.12E-26	8	Rps5
1.05E-29	0.668623124	0.45	0.205	1.39E-25	8	Ssr1
1.12E-29	0.597366676	0.331	0.123	1.47E-25	8	Med21
1.27E-29	0.592932484	0.436	0.19	1.67E-25	8	Ssr4
1.94E-29	0.333133499	0.986	0.867	2.56E-25	8	Rpl18a
2.89E-29	0.297706214	1	0.978	3.81E-25	8	mt-Co1
2.94E-29	0.576483902	0.734	0.462	3.88E-25	8	Rps27l
8.38E-29	0.413292255	0.908	0.71	1.11E-24	8	Rpl27a
4.33E-28	-1.127486722	0.322	0.557	5.71E-24	8	Psap
4.54E-28	0.449798659	0.875	0.649	5.99E-24	8	Rps20
4.84E-28	0.405304032	0.894	0.706	6.39E-24	8	Rpl11
5.04E-28	0.520831694	0.705	0.418	6.64E-24	8	Ybx1
6.72E-28	0.567697358	0.257	0.084	8.87E-24	8	Atp8b4
8.89E-28	0.510648953	0.778	0.492	1.17E-23	8	Cbx3
1.13E-27	-1.580829902	0.908	0.932	1.49E-23	8	Camp
1.56E-27	0.505584697	0.724	0.422	2.05E-23	8	Gm10073
1.57E-27	0.612289418	0.455	0.209	2.07E-23	8	Cd63
1.58E-27	0.368892675	0.954	0.761	2.09E-23	8	Rps3a1

1.61E-27	0.588825318	0.347	0.138	2.12E-23	8	Manf
1.94E-27	0.464255809	0.854	0.618	2.56E-23	8	Uba52
4.28E-27	0.725974672	0.322	0.129	5.64E-23	8	Snhg9
5.12E-27	0.344053018	0.951	0.796	6.75E-23	8	Rpl34
5.48E-27	0.508128261	0.71	0.435	7.23E-23	8	Atpif1
7.92E-27	0.483761987	0.734	0.449	1.05E-22	8	Rpl30
1.33E-26	0.658240008	0.515	0.27	1.76E-22	8	Cmtm7
1.38E-26	0.382749877	0.935	0.733	1.82E-22	8	Rps15
1.52E-26	0.505351284	0.694	0.411	2.01E-22	8	Serbp1
2.04E-26	0.54156996	0.591	0.316	2.69E-22	8	Clec12a
6.70E-26	0.469007231	0.743	0.459	8.84E-22	8	Rps26-ps1
1.01E-25	0.400618462	0.905	0.67	1.34E-21	8	Rpsa
1.92E-25	-0.726721779	0.545	0.693	2.53E-21	8	Cyba
2.25E-25	0.411924518	0.873	0.668	2.97E-21	8	Rpl27
1.54E-24	-0.486151345	0.995	0.998	2.03E-20	8	Malat1
2.45E-24	0.478007195	0.58	0.326	3.23E-20	8	Hsp90b1
4.11E-24	0.504830588	0.615	0.363	5.42E-20	8	Eif5a
4.46E-24	0.675712324	0.442	0.222	5.88E-20	8	Abhd2
5.18E-24	0.556400187	0.537	0.293	6.83E-20	8	Canx
6.84E-24	0.465958998	0.683	0.403	9.03E-20	8	Hsp90ab1
3.36E-23	0.455653229	0.707	0.435	4.44E-19	8	Uqcr11
9.06E-23	-1.52932978	0.141	0.372	1.19E-18	8	Ifi27l2a
1.94E-22	0.348490138	0.94	0.799	2.56E-18	8	Rps16
2.12E-22	0.465178258	0.669	0.417	2.80E-18	8	Rpl5
2.26E-22	0.339877851	0.897	0.736	2.99E-18	8	Rpl9
7.23E-22	0.539399669	0.499	0.265	9.54E-18	8	mt-Nd4l
7.66E-22	0.328722061	0.913	0.698	1.01E-17	8	Rplp0
1.26E-21	0.508996229	0.556	0.312	1.66E-17	8	Hsp90aa1
1.88E-21	0.515362593	0.431	0.214	2.48E-17	8	Nme1
2.07E-21	0.352268392	0.916	0.714	2.73E-17	8	Rps4x
3.32E-21	-1.064547328	0.106	0.329	4.38E-17	8	Lsp1

4.72E-21	0.289376187	0.949	0.8	6.22E-17	8	Rpl13a
6.41E-21	0.579036213	0.371	0.177	8.45E-17	8	Ramp1
7.14E-21	0.401405008	0.832	0.644	9.42E-17	8	mt-Co2
7.30E-21	0.562657442	0.393	0.194	9.63E-17	8	Hspa5
8.71E-21	0.613176759	0.417	0.213	1.15E-16	8	Rpgrip1
9.34E-21	0.332994874	0.908	0.677	1.23E-16	8	Hmgb1
1.58E-20	0.446066905	0.707	0.456	2.08E-16	8	Rpl29
1.82E-20	0.371326015	0.821	0.587	2.41E-16	8	Rpl3
2.66E-20	0.387826287	0.732	0.454	3.51E-16	8	Gm8730
4.72E-20	0.414225487	0.751	0.506	6.23E-16	8	Snrpg
5.55E-20	0.55825947	0.336	0.155	7.32E-16	8	Paip1
1.32E-19	0.509265201	0.585	0.361	1.74E-15	8	Tmem256
1.35E-19	0.560341915	0.29	0.125	1.78E-15	8	Sephs2
2.27E-19	0.382550357	0.808	0.565	2.99E-15	8	Rpl15
2.90E-19	0.427283979	0.531	0.299	3.82E-15	8	Cycs
3.16E-19	-0.566027039	0.566	0.694	4.16E-15	8	Actg1
3.44E-19	0.3097082	0.932	0.758	4.54E-15	8	Rpl21
4.55E-19	0.351628465	0.802	0.562	6.01E-15	8	Rpl10a
5.52E-19	0.349253451	0.837	0.647	7.28E-15	8	Rps10
5.66E-19	0.50680683	0.371	0.184	7.46E-15	8	St13
5.99E-19	0.373723208	0.775	0.561	7.90E-15	8	Rpl36al
6.14E-19	0.508302974	0.293	0.128	8.10E-15	8	Csgalnact2
7.57E-19	0.352581631	0.816	0.574	9.99E-15	8	Hmgn2
7.80E-19	0.54366162	0.388	0.194	1.03E-14	8	Tacc1
1.34E-18	0.460303659	0.252	0.102	1.77E-14	8	Ifngr1
1.35E-18	0.388277738	0.743	0.521	1.78E-14	8	Atp5k
1.43E-18	-1.085992669	0.068	0.269	1.88E-14	8	Gm9844
1.81E-18	0.411889717	0.618	0.384	2.39E-14	8	Tomm7
2.86E-18	0.342771676	0.854	0.635	3.77E-14	8	Rpl8
3.07E-18	0.507065836	0.257	0.108	4.05E-14	8	Atp6ap2
3.35E-18	0.304565399	0.913	0.752	4.42E-14	8	Rps3

4.64E-18	0.47397659	0.369	0.182	6.13E-14	8	Gm10320
9.31E-18	0.346093594	0.816	0.599	1.23E-13	8	Gm11808
9.60E-18	0.403814008	0.737	0.512	1.27E-13	8	Tma7
1.01E-17	0.300421103	0.883	0.68	1.33E-13	8	Rps13
2.67E-17	0.539585692	0.257	0.113	3.53E-13	8	Cenpw
4.40E-17	0.441772086	0.255	0.109	5.80E-13	8	Nars
5.03E-17	0.343008208	0.818	0.577	6.63E-13	8	Rpl4
7.97E-17	0.470690792	0.331	0.161	1.05E-12	8	Ddx21
8.07E-17	0.28646245	0.897	0.711	1.06E-12	8	Rpl19
9.03E-17	0.47860073	0.252	0.109	1.19E-12	8	Pdia6
1.19E-16	0.47683316	0.553	0.354	1.56E-12	8	Gsr
1.60E-16	-1.457456939	0.992	0.995	2.10E-12	8	S100a9
1.75E-16	0.415170503	0.369	0.188	2.31E-12	8	AI662270
1.94E-16	0.406916179	0.599	0.372	2.56E-12	8	Eif2s2
3.17E-16	0.424564491	0.667	0.463	4.18E-12	8	Rpl27-ps3
4.52E-16	0.362956713	0.724	0.486	5.96E-12	8	Rpl7a
5.12E-16	0.441399194	0.412	0.229	6.76E-12	8	Cks1b
7.65E-16	0.401106755	0.412	0.218	1.01E-11	8	Hspe1
7.88E-16	0.457956123	0.566	0.361	1.04E-11	8	Srsf2
7.89E-16	0.448090492	0.355	0.182	1.04E-11	8	Dut
8.44E-16	0.461639224	0.425	0.241	1.11E-11	8	Ndufb2
8.74E-16	-0.911896446	0.266	0.439	1.15E-11	8	Anxa1
9.52E-16	0.368353327	0.713	0.499	1.26E-11	8	Pabpc1
1.62E-15	0.486111944	0.336	0.171	2.13E-11	8	Pdia3
1.67E-15	0.414087	0.396	0.214	2.21E-11	8	Krtcap2
1.80E-15	0.43073382	0.485	0.287	2.37E-11	8	Ndufc1
1.97E-15	-0.651183564	0.379	0.537	2.59E-11	8	Coro1a
2.27E-15	0.468011133	0.556	0.362	2.99E-11	8	Srrm2
2.67E-15	0.450877397	0.472	0.273	3.52E-11	8	Eef1g
3.22E-15	0.347762624	0.661	0.421	4.25E-11	8	Rpl13-ps3
4.17E-15	0.408877018	0.656	0.461	5.50E-11	8	Ndufa4

4.18E-15	0.477645632	0.363	0.194	5.51E-11	8	Supt16
4.84E-15	0.383459359	0.591	0.384	6.39E-11	8	Dstn
5.27E-15	0.468052433	0.266	0.125	6.95E-11	8	Lsm7
6.23E-15	0.474881466	0.482	0.291	8.22E-11	8	Erh
7.03E-15	0.394437631	0.588	0.38	9.28E-11	8	Gm8186
7.83E-15	0.397898496	0.537	0.334	1.03E-10	8	H2afy
8.29E-15	0.450863956	0.423	0.243	1.09E-10	8	Eif3a
1.07E-14	0.523076864	0.268	0.133	1.41E-10	8	Dtymk
1.13E-14	0.396269876	0.461	0.273	1.50E-10	8	Gm10269
1.17E-14	0.372864136	0.686	0.465	1.55E-10	8	F630028O10Rik
1.22E-14	0.441589915	0.363	0.196	1.61E-10	8	Ndufa5
1.43E-14	0.429523625	0.428	0.246	1.88E-10	8	Alyref
1.45E-14	0.410354067	0.407	0.224	1.91E-10	8	Usp50
1.59E-14	0.482358511	0.276	0.134	2.10E-10	8	Npm3
2.05E-14	0.342857412	0.648	0.436	2.71E-10	8	Rpl9-ps6
2.11E-14	0.42130185	0.314	0.16	2.79E-10	8	Tomm20
3.09E-14	0.4924461	0.293	0.149	4.08E-10	8	Dpy30
3.22E-14	-0.675123003	0.192	0.376	4.25E-10	8	Emp3
3.37E-14	0.260390857	0.921	0.784	4.44E-10	8	Ppia
3.77E-14	0.359154838	0.542	0.339	4.98E-10	8	Rpl6l
4.34E-14	0.399261174	0.295	0.145	5.72E-10	8	Rnaset2a
4.93E-14	0.397748209	0.322	0.167	6.51E-10	8	Mrpl23
5.72E-14	0.486333208	0.396	0.228	7.55E-10	8	Ptges3
7.35E-14	0.414611652	0.36	0.196	9.70E-10	8	Rasgrp2
9.89E-14	0.365637186	0.507	0.306	1.30E-09	8	Eef1b2
1.17E-13	0.369959658	0.444	0.26	1.55E-09	8	Romo1
1.27E-13	-0.814915412	0.157	0.325	1.67E-09	8	Ptprc
1.44E-13	0.416765377	0.333	0.18	1.90E-09	8	Eif3j1
1.44E-13	-0.958613841	0.117	0.284	1.90E-09	8	Ccr2
1.45E-13	0.435310267	0.306	0.159	1.92E-09	8	Ssr3
2.06E-13	0.489282887	0.257	0.128	2.72E-09	8	Rsl24d1

5.40E-13	0.366605156	0.336	0.181	7.12E-09	8	2700094K13Rik
5.57E-13	-0.758919655	0.209	0.373	7.34E-09	8	Lst1
5.99E-13	-0.797263434	0.108	0.273	7.90E-09	8	S100a10
6.84E-13	-0.475945379	0.724	0.774	9.03E-09	8	B2m
8.37E-13	0.35189678	0.312	0.163	1.10E-08	8	Tmed3
9.34E-13	0.546816913	0.257	0.131	1.23E-08	8	Ythdf2
1.10E-12	0.354113062	0.637	0.44	1.45E-08	8	Usmg5
1.26E-12	0.384904912	0.444	0.277	1.66E-08	8	Eif4a1
1.50E-12	0.367689938	0.523	0.338	1.98E-08	8	Cox7b
1.84E-12	0.257696764	0.799	0.603	2.43E-08	8	Ly6c2
2.00E-12	0.459277106	0.268	0.139	2.63E-08	8	Rbm15
2.83E-12	-0.819816675	0.103	0.259	3.73E-08	8	Tmcc1
2.93E-12	0.444165912	0.274	0.142	3.87E-08	8	Nhp2
2.95E-12	0.36997883	0.396	0.228	3.89E-08	8	Park7
3.09E-12	-0.601393088	0.566	0.647	4.07E-08	8	Fth1
3.27E-12	0.390344015	0.412	0.248	4.32E-08	8	Gm10282
3.50E-12	0.296700183	0.753	0.557	4.62E-08	8	Hnrnpa2b1
4.42E-12	0.416538383	0.409	0.248	5.83E-08	8	Lsm6
4.74E-12	0.372746647	0.407	0.242	6.25E-08	8	Ndufb3
4.85E-12	0.400429311	0.295	0.158	6.40E-08	8	Baz1a
9.47E-12	0.277737547	0.661	0.455	1.25E-07	8	Rpl10-ps3
9.71E-12	0.316701315	0.496	0.311	1.28E-07	8	Gm10036
1.21E-11	0.389750803	0.374	0.221	1.59E-07	8	Spcs2
1.28E-11	0.325648373	0.32	0.175	1.69E-07	8	Gm6133
1.78E-11	0.347080406	0.436	0.267	2.35E-07	8	Arglu1
2.12E-11	0.32479557	0.469	0.295	2.80E-07	8	Tsix
2.19E-11	0.311196931	0.58	0.386	2.89E-07	8	mt-Atp8
2.20E-11	0.350593509	0.637	0.465	2.90E-07	8	H2afj
2.47E-11	0.333601013	0.539	0.355	3.25E-07	8	Ran
2.61E-11	0.285668674	0.767	0.616	3.45E-07	8	Cox6c
2.92E-11	0.335440196	0.472	0.294	3.85E-07	8	mt-Nd2

3.30E-11	0.285354855	0.743	0.535	4.35E-07	8	Rpl7
4.10E-11	0.313763027	0.336	0.189	5.41E-07	8	Llph
5.41E-11	0.309325141	0.491	0.322	7.13E-07	8	Lsm5
8.49E-11	0.399355385	0.285	0.158	1.12E-06	8	G3bp1
1.08E-10	0.283656625	0.393	0.231	1.42E-06	8	Eif5b
1.11E-10	-0.479845457	0.569	0.65	1.46E-06	8	Prdx5
1.30E-10	0.424388249	0.309	0.18	1.72E-06	8	Skp1a
1.58E-10	0.397430655	0.369	0.226	2.08E-06	8	Snrpd3
2.04E-10	0.321405348	0.466	0.296	2.70E-06	8	Polr2l
2.14E-10	0.276276397	0.575	0.396	2.82E-06	8	Uqcrq
2.55E-10	0.333150436	0.466	0.295	3.37E-06	8	Hnrnpm
3.33E-10	0.402690299	0.301	0.169	4.40E-06	8	Tnfaip8
4.21E-10	0.376085888	0.355	0.215	5.56E-06	8	Pgam1
4.35E-10	0.332531728	0.61	0.444	5.74E-06	8	Uqcr10
5.42E-10	0.329825496	0.447	0.292	7.15E-06	8	Sfpq
5.48E-10	-0.686720335	0.13	0.264	7.23E-06	8	Anxa2
5.68E-10	0.306605142	0.491	0.327	7.50E-06	8	Nucks1
7.21E-10	0.359886695	0.317	0.187	9.51E-06	8	Tuba4a
7.54E-10	0.344458986	0.352	0.213	9.95E-06	8	Swi5
7.73E-10	0.365098854	0.293	0.169	1.02E-05	8	Siva1
7.86E-10	0.259598182	0.618	0.428	1.04E-05	8	Gnb2l1
8.10E-10	0.389025428	0.276	0.156	1.07E-05	8	Lig1
8.22E-10	0.419075444	0.336	0.2	1.08E-05	8	Fcnb
8.64E-10	-0.636660375	0.607	0.633	1.14E-05	8	Cst3
9.85E-10	0.282539292	0.607	0.416	1.30E-05	8	Hint1
9.93E-10	0.349429558	0.447	0.298	1.31E-05	8	Pcbp1
1.05E-09	0.361306289	0.266	0.148	1.38E-05	8	1110001J03Rik
1.07E-09	0.31378246	0.255	0.137	1.41E-05	8	Smim4
1.12E-09	0.351420229	0.282	0.159	1.48E-05	8	Stra13
1.14E-09	0.310487573	0.271	0.15	1.50E-05	8	Ndufa12
1.57E-09	0.343733981	0.341	0.204	2.07E-05	8	Serpinb1a

1.58E-09	-0.454456522	0.469	0.561	2.08E-05	8	Calm1
1.61E-09	0.441828098	0.336	0.21	2.13E-05	8	Birc6
1.62E-09	0.38360715	0.401	0.254	2.14E-05	8	Snrpd2
1.64E-09	0.331845455	0.369	0.228	2.17E-05	8	Metap2
1.69E-09	0.334422934	0.304	0.177	2.23E-05	8	Erdr1
2.67E-09	0.303268605	0.447	0.295	3.52E-05	8	Uqcrb
2.99E-09	0.400394929	0.282	0.164	3.94E-05	8	Pdcd5
4.65E-09	-0.359839619	0.645	0.713	6.13E-05	8	Myl6
5.61E-09	0.298221122	0.423	0.273	7.41E-05	8	Hnrnpab
7.03E-09	0.270087248	0.575	0.402	9.27E-05	8	Chchd2
7.22E-09	-0.323876766	0.762	0.776	9.52E-05	8	H3f3a
8.16E-09	0.319172346	0.534	0.377	0.000107577	8	Tmem258
8.28E-09	0.320716892	0.252	0.14	0.000109202	8	Chchd1
8.38E-09	0.277920741	0.618	0.467	0.00011055	8	Cox7a2
1.04E-08	0.308303177	0.436	0.292	0.000136866	8	Ost4
1.39E-08	0.292586082	0.572	0.415	0.000183033	8	Arpp19
1.42E-08	-0.61303922	0.198	0.324	0.000186774	8	Ms4a6c
1.44E-08	0.264563421	0.344	0.21	0.000190119	8	Tmed10
1.71E-08	0.258113367	0.748	0.594	0.000225277	8	Atp5e
1.86E-08	0.372130759	0.309	0.192	0.000245701	8	Caprin1
1.87E-08	0.335076194	0.388	0.255	0.000247105	8	Prrc2c
2.19E-08	0.437694739	0.252	0.146	0.000288693	8	Comt
2.19E-08	-0.653569808	0.141	0.261	0.000289247	8	Btg1
2.29E-08	-1.29787994	0.997	0.999	0.0003019	8	S100a8
2.38E-08	0.315554365	0.407	0.268	0.00031405	8	Lamtor2
3.22E-08	-0.602760393	0.176	0.298	0.000425026	8	Mgst1
5.74E-08	0.289672579	0.393	0.262	0.000757651	8	Tmed2
6.40E-08	0.285657855	0.496	0.354	0.000844755	8	Eif4g2
6.55E-08	-0.564342583	0.144	0.263	0.000864122	8	Vim
6.92E-08	-0.468725305	0.358	0.457	0.000912665	8	Alox5ap
8.65E-08	0.282875364	0.252	0.146	0.00114111	8	Ostc

8.97E-08	0.309967685	0.271	0.161	0.001182741	8	Yme1l1
1.03E-07	0.281614495	0.425	0.291	0.001359188	8	Atp5g2
1.48E-07	0.268462615	0.306	0.19	0.001945841	8	2410015M20Rik
1.49E-07	-0.513415708	0.22	0.335	0.001970236	8	Cd53
1.59E-07	0.260694839	0.683	0.543	0.002098951	8	Cox6b1
1.94E-07	-0.552302301	0.152	0.265	0.002556288	8	Ucp2
2.01E-07	0.345581078	0.325	0.212	0.002644825	8	Hnrnpul1
2.05E-07	0.264074749	0.382	0.255	0.002709132	8	Vapa
2.45E-07	0.262194501	0.369	0.238	0.003232709	8	Srp9
3.57E-07	0.314669014	0.358	0.24	0.004713833	8	Hnrnph1
4.19E-07	0.307025752	0.266	0.161	0.005529094	8	Cited2
4.43E-07	0.294879896	0.268	0.161	0.005838836	8	Pfdn2
1.01E-06	0.256166535	0.447	0.319	0.013351176	8	Ndufa7
1.07E-06	-0.47357942	0.534	0.58	0.014156803	8	H2-D1
1.21E-06	0.303016846	0.29	0.186	0.015976028	8	Sf3b5
2.09E-06	0.293051663	0.287	0.187	0.027574582	8	Polr2k
2.11E-06	-0.466342982	0.157	0.264	0.027810289	8	Lmo4
2.36E-06	0.289790588	0.355	0.246	0.031079082	8	Timm13
2.69E-06	0.279392358	0.317	0.21	0.035489627	8	Slc25a3
2.73E-06	0.264931386	0.295	0.193	0.036035975	8	Gm10020
2.79E-06	-0.530175231	0.179	0.272	0.036836367	8	Cotl1
6.89E-06	0.251567781	0.322	0.22	0.090852827	8	Lsm4
9.49E-06	-0.257077039	0.691	0.693	0.125189768	8	Sh3bgrl3
9.72E-06	-1.053783712	0.669	0.628	0.128212716	8	Wfdc21
1.14E-05	0.288413132	0.268	0.179	0.150698742	8	Sypl
1.19E-05	0.306816473	0.255	0.168	0.157543002	8	Naa38
1.31E-05	-0.385794273	0.285	0.377	0.17325146	8	Arpc5
2.96E-05	0.285120239	0.26	0.175	0.390903397	8	Dnmt1
3.03E-05	0.301822898	0.274	0.187	0.400109369	8	Rrm2
3.08E-05	-0.330232659	0.358	0.436	0.405659549	8	Serinc3
3.11E-05	-0.700423247	0.425	0.449	0.409941921	8	Pglyrp1

3.47E-05	0.295639184	0.255	0.173	0.457846143	8	Smarca5	
3.72E-05	0.29061703	0.29	0.199	0.491119857	8	Pan3	
9.07E-05	0.25892214	0.268	0.182	1	8	Cap1	
0.00012606	-0.364412721	0.355	0.421	1	8	Cdc42	
0.00013077	-0.302476693	0.436	0.493	1	8	Arpc2	
0.00029343	-0.394084846	0.209	0.284	1	8	Lcp1	
0.00039813	-0.414712386	0.184	0.254	1	8	Ostf1	
0.00042271	-0.389623425	0.276	0.341	1	8	Clic1	
0.00047844	-0.335809809	0.209	0.285	1	8	Rap1b	
0.00051047	-0.382960748	0.211	0.282	1	8	Iqgap1	
0.00094796	-0.338922737	0.22	0.289	1	8	Tmpo	
0.00095662	-0.278063622	0.851	0.775	1	8	Hmgb2	
0.00118445	-0.381715377	0.388	0.431	1	8	Prdx1	
0.00148646	-0.343980478	0.222	0.288	1	8	Cd44	
0.00198343	-0.365466251	0.266	0.326	1	8	Npc2	
0.00290795	-0.280574615	0.317	0.371	1	8	Capza2	
0.00306956	-0.285705219	0.195	0.259	1	8	Flna	
0.00379662	-0.302099843	0.455	0.464	1	8	Rac2	
0.00426912	-0.364784385	0.252	0.296	1	8	Laptm5	
0.00717594	-0.266258075	0.225	0.278	1	8	Aldh2	
0.00735073	-0.307585427	0.228	0.28	1	8	Reep5	
0.00755834	-0.286198171	0.564	0.583	1	8	S100a11	
0	2.579738724	0.71	0.029	0	9	Siglech	**plasmacytoid DC**
0	2.523141786	0.662	0.042	0	9	Cox6a2	
0	2.511162531	0.907	0.145	0	9	Bst2	
0	2.449824196	0.808	0.092	0	9	Tcf4	
0	1.980860514	0.369	0.007	0	9	Ccr9	
0	1.689518085	0.293	0.002	0	9	D13Ertd608e	
5.48E-282	2.3542833	0.721	0.099	7.23E-278	9	Ly6d	
3.95E-262	2.121077476	0.859	0.185	5.22E-258	9	Irf8	
8.68E-225	1.943883546	0.721	0.125	1.15E-220	9	Cd74	

6.75E-223	1.816751033	0.349	0.02	8.90E-219	9	Ctsl
2.60E-221	1.909262931	0.558	0.069	3.43E-217	9	Runx2
2.37E-202	1.592662354	0.318	0.018	3.12E-198	9	Spib
7.44E-193	1.235492035	1	0.997	9.81E-189	9	Malat1
4.00E-190	1.973757933	0.608	0.106	5.28E-186	9	Dnajc7
8.33E-190	1.852954996	0.561	0.086	1.10E-185	9	Rnase6
9.82E-189	1.728324026	0.276	0.014	1.30E-184	9	Ly6a
7.98E-178	1.583241701	0.49	0.063	1.05E-173	9	H2-Aa
6.48E-176	1.581182224	0.299	0.019	8.55E-172	9	Ccnd1
7.84E-142	1.388220014	0.963	0.624	1.03E-137	9	Fth1
2.07E-133	1.542431802	0.439	0.068	2.73E-129	9	H2-Ab1
3.80E-128	1.524332931	0.301	0.031	5.02E-124	9	Atp1b1
8.88E-128	1.448071163	0.296	0.03	1.17E-123	9	Cd7
2.07E-123	1.604350044	0.361	0.05	2.73E-119	9	Lair1
2.27E-115	1.579871987	0.521	0.123	2.99E-111	9	Bcl11a
1.18E-97	1.023860732	0.932	0.696	1.56E-93	9	Rpl31
5.02E-97	1.489103872	0.369	0.068	6.62E-93	9	Nucb2
2.44E-96	1.433130736	0.313	0.048	3.22E-92	9	Smim5
5.83E-92	0.598634387	1	0.978	7.69E-88	9	mt-Co1
7.52E-90	-1.733039774	0.611	0.923	9.91E-86	9	Lyz2
5.62E-85	-1.442095683	0.321	0.806	7.42E-81	9	Hmgb2
2.76E-83	0.828053868	0.963	0.806	3.64E-79	9	Tyrobp
2.10E-82	1.484967555	0.307	0.054	2.78E-78	9	Sema4b
7.54E-82	0.589413664	1	0.954	9.94E-78	9	Rps27
1.21E-81	0.929169985	0.89	0.523	1.59E-77	9	Psap
9.43E-81	1.312355281	0.555	0.192	1.24E-76	9	Ctsb
4.41E-79	1.377585697	0.341	0.07	5.82E-75	9	Tspan13
2.42E-77	-1.110252572	0.42	0.843	3.19E-73	9	H2afz
2.12E-74	0.970677638	0.837	0.457	2.79E-70	9	Lgals1
2.82E-68	1.286898736	0.47	0.152	3.72E-64	9	Pld4
1.72E-61	1.25308203	0.434	0.14	2.27E-57	9	Ctsh

1.78E-61	0.6494613	0.963	0.837	2.34E-57	9	Rps27rt
8.36E-61	1.017419542	0.746	0.49	1.10E-56	9	Mbnl1
5.87E-60	0.628575983	0.983	0.852	7.74E-56	9	mt-Atp6
1.34E-56	1.149067995	0.482	0.179	1.76E-52	9	Mpeg1
1.97E-54	0.837273653	0.792	0.564	2.60E-50	9	H2-D1
6.20E-54	0.711855846	0.896	0.763	8.18E-50	9	B2m
5.46E-52	-1.753805977	0.152	0.576	7.21E-48	9	Chil3
2.64E-51	0.441136686	0.994	0.952	3.48E-47	9	mt-Co3
8.11E-50	0.821327821	0.735	0.453	1.07E-45	9	Ly6e
1.32E-48	1.168906438	0.468	0.197	1.75E-44	9	Grn
1.48E-48	1.117418342	0.349	0.108	1.96E-44	9	Mef2c
3.85E-48	0.759075972	0.806	0.627	5.08E-44	9	Xist
1.35E-45	-1.95302502	0.287	0.65	1.77E-41	9	Wfdc21
2.48E-45	-1.841177818	0.076	0.469	3.28E-41	9	Pglyrp1
1.38E-44	1.158109768	0.256	0.065	1.82E-40	9	Cyth4
8.68E-44	-0.445783247	0.992	0.996	1.15E-39	9	Tmsb4x
1.02E-43	-1.53418236	0.031	0.418	1.35E-39	9	Dstn
1.14E-43	-1.933061705	0.206	0.579	1.50E-39	9	Lcn2
8.07E-42	1.158513171	0.383	0.145	1.06E-37	9	St8sia4
3.72E-41	-1.667986927	0.037	0.405	4.90E-37	9	Hist1h2ap
6.42E-41	-2.03294732	0.994	0.999	8.47E-37	9	S100a8
5.13E-40	-1.217496936	0.11	0.499	6.77E-36	9	F630028O10Rik
2.25E-39	-1.151497464	0.231	0.608	2.96E-35	9	Hmgn2
4.35E-39	-1.429062044	0.079	0.45	5.74E-35	9	Anxa1
5.94E-38	-1.768779492	0.22	0.56	7.83E-34	9	Ltf
1.44E-37	0.576707846	0.89	0.684	1.90E-33	9	Rpl10
6.12E-36	-1.407543947	0.025	0.359	8.08E-32	9	Ifitm2
9.46E-36	0.486586151	0.93	0.801	1.25E-31	9	Rpl13a
3.49E-34	0.936775655	0.561	0.346	4.60E-30	9	H2-K1
6.90E-34	-1.22011366	0.223	0.578	9.10E-30	9	Ifitm3
1.87E-33	-1.235731999	0.115	0.457	2.47E-29	9	Lgals3

4.09E-33	-1.959237371	0.825	0.937	5.39E-29	9	Camp
7.37E-33	0.414800841	0.98	0.876	9.72E-29	9	Rps14
1.98E-32	-2.041369422	0.989	0.995	2.61E-28	9	S100a9
3.70E-32	0.533751682	0.845	0.735	4.88E-28	9	H3f3b
3.82E-32	-2.052067637	0.665	0.86	5.04E-28	9	Ngp
5.80E-32	0.973740698	0.358	0.152	7.65E-28	9	Sepw1
8.44E-32	0.913413011	0.465	0.242	1.11E-27	9	Btg1
1.83E-31	0.462378699	0.932	0.786	2.42E-27	9	Rps11
3.25E-31	0.99521681	0.287	0.103	4.29E-27	9	H2-T23
1.25E-30	-1.4183856	0.068	0.377	1.65E-26	9	Ifitm6
1.40E-30	-0.852088574	0.242	0.601	1.85E-26	9	S100a11
4.04E-30	-0.974448999	0.135	0.473	5.33E-26	9	Hp
6.71E-30	-1.141521977	0.068	0.381	8.85E-26	9	Lst1
7.32E-30	-1.216099762	0.037	0.335	9.66E-26	9	Top2a
2.80E-29	1.163672879	0.268	0.096	3.69E-25	9	Insig1
3.12E-29	0.551732828	0.837	0.696	4.12E-25	9	mt-Cytb
1.83E-28	-0.61587906	0.476	0.792	2.41E-24	9	H3f3a
6.24E-28	1.050763168	0.273	0.103	8.23E-24	9	Snx5
1.00E-27	0.945988699	0.428	0.235	1.32E-23	9	Pgls
1.59E-27	1.006105559	0.265	0.097	2.10E-23	9	Bmp2k
2.19E-27	1.021331073	0.299	0.123	2.89E-23	9	Rnaset2b
4.03E-27	-1.115947922	0.062	0.349	5.32E-23	9	2810417H13Rik
1.10E-26	0.433296571	0.851	0.674	1.45E-22	9	Plac8
1.96E-26	-0.957672757	0.085	0.382	2.59E-22	9	Gsr
3.46E-26	0.744511237	0.583	0.423	4.57E-22	9	Serinc3
1.28E-25	0.937049803	0.279	0.109	1.69E-21	9	Ly86
1.33E-25	-0.666114391	0.355	0.683	1.76E-21	9	Arhgdib
2.22E-24	-0.922898735	0.087	0.369	2.93E-20	9	Msrb1
5.18E-24	0.593605171	0.699	0.563	6.84E-20	9	mt-Nd4
7.34E-24	0.926774856	0.315	0.141	9.68E-20	9	Trim30a
9.35E-24	-0.837938169	0.068	0.341	1.23E-19	9	Cks2

6.65E-23	-0.84728368	0.073	0.342	8.78E-19	9	Ap3s1
7.81E-23	0.851676724	0.428	0.254	1.03E-18	9	Gnas
1.75E-22	-0.741173099	0.144	0.44	2.31E-18	9	Tkt
2.43E-22	-0.730259656	0.138	0.429	3.21E-18	9	Stmn1
4.40E-22	-0.982573605	0.039	0.28	5.81E-18	9	Cotl1
6.70E-22	0.910510281	0.38	0.209	8.84E-18	9	Smim14
7.05E-22	0.699205631	0.496	0.306	9.30E-18	9	Lsp1
8.48E-22	0.841461728	0.268	0.113	1.12E-17	9	Unc93b1
3.40E-21	0.555683849	0.732	0.613	4.49E-17	9	mt-Nd1
4.69E-21	-1.008626364	0.039	0.27	6.18E-17	9	Cebpb
4.69E-21	-0.924427849	0.037	0.269	6.19E-17	9	Flna
4.87E-21	-0.617109459	0.372	0.661	6.43E-17	9	Prdx5
6.77E-21	-1.010686259	0.048	0.279	8.93E-17	9	Clec4a2
8.45E-21	-0.932377371	0.062	0.304	1.11E-16	9	Mgst1
5.34E-20	0.749576001	0.454	0.295	7.04E-16	9	mt-Nd2
5.79E-20	-0.89614495	0.045	0.27	7.63E-16	9	Gm10282
7.11E-20	-0.664044202	0.149	0.429	9.38E-16	9	Gapdh
8.35E-20	0.904669289	0.369	0.211	1.10E-15	9	Fgfr1op2
8.41E-20	-0.764616001	0.245	0.512	1.11E-15	9	Tubb5
9.25E-20	0.845455723	0.315	0.161	1.22E-15	9	Cd164
1.28E-19	-0.831043587	0.045	0.271	1.69E-15	9	Aprt
1.67E-19	-1.008665129	0.096	0.33	2.20E-15	9	Ms4a6c
7.78E-19	0.845318583	0.372	0.211	1.03E-14	9	Selplg
2.46E-18	0.752211031	0.47	0.333	3.25E-14	9	Hsp90b1
3.11E-18	-0.686963008	0.127	0.382	4.10E-14	9	Tuba1b
4.00E-18	-0.620479543	0.186	0.46	5.27E-14	9	Gmfg
5.09E-18	-0.412215913	0.532	0.795	6.72E-14	9	Shfm1
6.54E-18	-0.77730808	0.138	0.383	8.62E-14	9	Myh9
1.49E-17	-0.81081042	0.085	0.309	1.97E-13	9	Cd24a
4.09E-17	0.81874631	0.372	0.221	5.40E-13	9	Sell
6.20E-17	0.702821862	0.485	0.356	8.18E-13	9	Serp1

1.57E-16	-0.499882681	0.507	0.743	2.07E-12	9	Cd52
2.05E-16	0.819164136	0.327	0.185	2.71E-12	9	Ogt
2.70E-16	0.587865804	0.361	0.197	3.56E-12	9	Ctss
5.16E-16	-0.555158269	0.208	0.465	6.81E-12	9	Atpif1
5.18E-16	-0.550795981	0.149	0.392	6.83E-12	9	Lbr
6.43E-16	-0.568369492	0.175	0.422	8.48E-12	9	Anp32b
8.89E-16	-0.755334896	0.068	0.267	1.17E-11	9	Vim
1.18E-15	-0.582127971	0.172	0.411	1.56E-11	9	Actr3
1.32E-15	-0.591806361	0.107	0.33	1.73E-11	9	Capza1
1.69E-15	0.431398346	0.724	0.651	2.22E-11	9	mt-Co2
1.79E-15	-0.580086423	0.135	0.368	2.36E-11	9	Fam49b
2.24E-15	0.426725058	0.727	0.632	2.95E-11	9	Sec61g
2.33E-15	0.790952801	0.31	0.175	3.07E-11	9	Kctd12
6.07E-15	-0.591236349	0.09	0.296	8.00E-11	9	Tmpo
8.59E-15	-1.141033711	0.093	0.283	1.13E-10	9	Prtn3
1.12E-14	-0.478229543	0.225	0.484	1.48E-10	9	Txn1
1.14E-14	-0.98306478	0.476	0.646	1.51E-10	9	Crip1
1.39E-14	-0.789831039	0.073	0.26	1.83E-10	9	Tmcc1
1.40E-14	-0.59078222	0.079	0.282	1.85E-10	9	Aldoa
1.61E-14	-0.422381707	0.794	0.845	2.12E-10	9	Ptma
1.63E-14	-1.49277532	0.076	0.257	2.14E-10	9	Elane
1.89E-14	0.694561601	0.375	0.244	2.49E-10	9	Psmb8
3.24E-14	-0.591568803	0.099	0.303	4.27E-10	9	Pkm
5.05E-14	-0.401999557	0.439	0.701	6.66E-10	9	Actg1
5.84E-14	-0.574640206	0.065	0.251	7.71E-10	9	Rnaseh2c
5.93E-14	0.314219479	0.856	0.788	7.82E-10	9	Ppia
8.65E-14	0.564552295	0.555	0.462	1.14E-09	9	Rpl10-ps3
1.41E-13	0.261654464	0.946	0.87	1.86E-09	9	Rpl18a
1.45E-13	-0.568464793	0.079	0.268	1.92E-09	9	Lmo4
1.51E-13	-0.512751196	0.186	0.416	1.99E-09	9	Dbi
1.70E-13	-0.540223607	0.093	0.288	2.25E-09	9	Bola2

2.25E-13	0.60528142	0.439	0.315	2.96E-09	9	Npc2
2.78E-13	-0.453650895	0.169	0.397	3.67E-09	9	1810037I17Rik
3.34E-13	-0.46363553	0.096	0.292	4.41E-09	9	Hnrnpab
3.78E-13	0.540396005	0.535	0.462	4.99E-09	9	Cd47
8.07E-13	-1.049443584	0.104	0.28	1.06E-08	9	Mpo
1.04E-12	-0.505207796	0.163	0.38	1.37E-08	9	Dek
1.58E-12	-0.442724806	0.994	1	2.08E-08	9	Actb
1.99E-12	-0.549993197	0.099	0.285	2.63E-08	9	Aldh2
2.36E-12	-0.470479892	0.29	0.521	3.12E-08	9	Cbx3
2.40E-12	-0.442040472	0.301	0.549	3.16E-08	9	Mrpl33
3.33E-12	-0.459520647	0.141	0.347	4.40E-08	9	Nucks1
4.66E-12	-0.427798773	0.175	0.388	6.15E-08	9	H2afv
1.00E-11	-0.466278677	0.115	0.303	1.32E-07	9	Ppp1cb
1.86E-11	-0.636923265	0.099	0.267	2.45E-07	9	Gm9844
2.26E-11	-0.443009579	0.113	0.299	2.98E-07	9	Ywhah
2.34E-11	0.258661694	0.831	0.719	3.09E-07	9	Rps4x
3.44E-11	-0.319973431	0.462	0.729	4.54E-07	9	Cfl1
3.53E-11	-0.405153791	0.115	0.303	4.65E-07	9	Nap1l1
3.74E-11	-0.684640595	0.377	0.541	4.94E-07	9	S100a6
5.41E-11	-0.358145839	0.499	0.721	7.13E-07	9	Myl6
1.08E-10	0.635184658	0.327	0.218	1.43E-06	9	Psme2
1.88E-10	0.668799671	0.262	0.157	2.48E-06	9	Ctsz
2.12E-10	-0.418801992	0.146	0.334	2.80E-06	9	Scp2
2.56E-10	-0.371896331	0.099	0.266	3.38E-06	9	Alyref
3.83E-10	-0.558131911	0.113	0.273	5.05E-06	9	S100a10
5.11E-10	0.338681067	0.721	0.695	6.74E-06	9	Oaz1
5.22E-10	-0.388843133	0.11	0.278	6.88E-06	9	Gpi1
7.09E-10	0.699172578	0.318	0.219	9.35E-06	9	Rpgrip1
7.34E-10	-0.406325151	0.273	0.468	9.68E-06	9	Rps12-ps3
8.65E-10	-0.360909738	0.158	0.342	1.14E-05	9	Lsm5
8.97E-10	-0.466562402	0.096	0.251	1.18E-05	9	Zfp36l2

1.08E-09	0.29851793	0.814	0.717	1.42E-05	9	Rpl19
1.59E-09	-0.493642523	0.127	0.288	2.10E-05	9	Slpi
1.96E-09	-0.35302367	0.276	0.489	2.59E-05	9	Gng5
3.00E-09	-0.428250341	0.11	0.265	3.95E-05	9	Anxa2
4.03E-09	0.352395299	0.476	0.352	5.31E-05	9	Ifi27l2a
4.93E-09	-0.301068556	0.22	0.423	6.50E-05	9	Atox1
6.32E-09	-0.638092574	0.135	0.282	8.34E-05	9	Ccr2
7.08E-09	-0.289378788	0.234	0.438	9.33E-05	9	Ndufb7
8.96E-09	0.378388436	0.614	0.571	0.000118249	9	Rpl36al
2.00E-08	-0.304596979	0.211	0.402	0.000264018	9	Ywhaz
2.76E-08	0.624555365	0.313	0.225	0.000364561	9	Rrbp1
2.83E-08	-0.367425296	0.169	0.334	0.000373241	9	Ranbp1
3.35E-08	-0.301791461	0.13	0.285	0.000441614	9	Rac1
6.03E-08	0.432479005	0.541	0.505	0.000795208	9	Sec61b
7.25E-08	-0.288438543	0.242	0.438	0.000956612	9	Rbm3
1.34E-07	0.407264192	0.566	0.54	0.001764652	9	Ubb
1.88E-07	-0.263457545	0.344	0.545	0.002480505	9	Atp5k
2.64E-07	-0.322597491	0.132	0.273	0.003488509	9	Gnb1
8.40E-07	0.478406985	0.389	0.33	0.011085272	9	Irf2bp2
1.86E-06	-0.757801235	0.732	0.74	0.024518778	9	Tmsb10
2.25E-06	0.499980552	0.344	0.284	0.029737791	9	Tagln2
2.68E-06	0.316562914	0.642	0.598	0.035400221	9	Rpl3
3.38E-06	-0.329393173	0.563	0.665	0.044553029	9	Rps12
1.18E-05	-0.298663999	0.175	0.301	0.156304369	9	Sat1
1.21E-05	-0.253639107	0.208	0.349	0.159315033	9	Set
1.28E-05	0.347286961	0.496	0.462	0.169128366	9	Rac2
1.30E-05	0.581387967	0.285	0.227	0.172078008	9	Spcs2
3.37E-05	0.303114462	0.628	0.65	0.445053805	9	Itm2b
3.89E-05	0.595150516	0.265	0.207	0.512891408	9	Tram1
5.96E-05	0.523782912	0.265	0.208	0.786166057	9	Fus
7.11E-05	0.342224373	0.279	0.208	0.938097459	9	Ahnak

0.00013923	0.605384212	0.27	0.222	1	9	Krtcap2	
0.00043869	-0.719027246	0.335	0.485	1	9	Retnlg	
0.00051092	0.420944451	0.361	0.333	1	9	Pomp	
0.00067933	0.369412678	0.361	0.326	1	9	Cd53	
0.00078489	0.397063944	0.383	0.363	1	9	Son	
0.00137838	0.375113781	0.33	0.292	1	9	Laptm5	
0.00156519	0.442213536	0.259	0.22	1	9	Gltscr2	
0.00848106	0.415749448	0.268	0.242	1	9	Rap1a	
0.00906724	0.369262578	0.315	0.304	1	9	Tsix	
0.00918761	0.29510345	0.389	0.386	1	9	Tmem258	
9.16E-160	1.847357759	0.791	0.196	1.21E-155	10	Wfdc17	Monocyte
2.59E-135	1.51143059	0.976	0.539	3.41E-131	10	Ifitm3	
5.58E-127	1.356390306	0.253	0.017	7.36E-123	10	Sirpb1c	
7.19E-116	1.0712853	1	0.901	9.48E-112	10	Lyz2	
3.53E-111	1.624304228	0.397	0.059	4.66E-107	10	Gngt2	
2.61E-106	1.4893792	0.839	0.336	3.44E-102	10	Ifi27l2a	
6.78E-97	1.479456017	0.64	0.186	8.95E-93	10	Ctss	
1.29E-91	1.801560521	0.497	0.116	1.71E-87	10	Apoe	
4.67E-83	1.214557875	0.647	0.206	6.15E-79	10	S100a4	
1.03E-80	1.294802061	0.312	0.048	1.36E-76	10	Clec4a3	
7.14E-74	1.027917951	0.945	0.672	9.42E-70	10	Plac8	
7.98E-73	1.298899273	0.548	0.166	1.05E-68	10	Ccl6	
1.66E-71	0.981108593	0.89	0.527	2.20E-67	10	Psap	
8.09E-71	1.244152885	0.661	0.256	1.07E-66	10	Ccr2	
1.18E-70	0.983819265	0.901	0.619	1.56E-66	10	Cst3	
5.99E-70	1.293524532	0.339	0.065	7.90E-66	10	Csf1r	
2.73E-68	1.38799775	0.387	0.088	3.60E-64	10	Ms4a4c	
1.03E-62	0.739415858	0.962	0.808	1.36E-58	10	Tyrobp	
1.67E-62	1.283723215	0.51	0.169	2.21E-58	10	Samhd1	
9.09E-62	1.151443341	0.264	0.044	1.20E-57	10	Clec4a1	
2.66E-61	0.822797426	0.928	0.763	3.51E-57	10	B2m	

1.44E-59	0.739216508	0.952	0.814	1.90E-55	10	Ftl1
8.19E-59	1.097183984	0.678	0.3	1.08E-54	10	Ms4a6c
4.70E-54	0.855419607	0.853	0.517	6.20E-50	10	S100a6
5.81E-53	1.111710549	0.682	0.349	7.66E-49	10	Lst1
8.93E-52	-1.703435965	0.13	0.609	1.18E-47	10	Hmgn2
1.05E-50	0.647568602	0.99	0.728	1.39E-46	10	Tmsb10
4.36E-50	0.684525163	0.935	0.623	5.75E-46	10	Crip1
1.17E-47	0.38558007	1	0.997	1.54E-43	10	Malat1
1.65E-47	-1.111936846	0.493	0.793	2.18E-43	10	Hmgb2
5.42E-47	1.181244653	0.342	0.093	7.14E-43	10	Ms4a6b
3.98E-45	0.843937172	0.801	0.566	5.26E-41	10	H2-D1
1.90E-44	0.98445446	0.616	0.308	2.50E-40	10	Npc2
3.01E-42	0.668105252	0.908	0.705	3.97E-38	10	Fcer1g
3.12E-38	0.394537338	0.997	0.955	4.11E-34	10	Rps27
2.83E-37	1.063475858	0.277	0.075	3.73E-33	10	Cx3cr1
1.54E-36	1.036434002	0.346	0.112	2.04E-32	10	Ccl9
9.93E-35	0.768720516	0.702	0.426	1.31E-30	10	Lgals3
2.91E-34	-1.834610447	0.312	0.645	3.84E-30	10	Wfdc21
2.54E-31	-1.745696697	0.216	0.557	3.35E-27	10	Ltf
2.70E-31	1.097217841	0.284	0.09	3.56E-27	10	Sepp1
2.88E-31	0.741734241	0.702	0.458	3.80E-27	10	Ly6e
1.18E-30	-0.865365183	0.387	0.705	1.56E-26	10	Hmgb1
1.53E-29	1.012255188	0.295	0.098	2.02E-25	10	Marcks
2.01E-29	0.303329748	1	0.996	2.66E-25	10	Tmsb4x
6.27E-29	-0.715955958	0.736	0.847	8.26E-25	10	Ptma
8.01E-29	-1.730803594	0.264	0.572	1.06E-24	10	Lcn2
8.02E-29	0.879038471	0.466	0.225	1.06E-24	10	Zeb2
2.47E-28	0.970495575	0.507	0.284	3.25E-24	10	Sat1
2.68E-28	0.532542745	0.873	0.723	3.54E-24	10	Cd52
2.96E-28	-1.127779053	0.168	0.513	3.90E-24	10	Tubb5
2.51E-27	0.436981704	0.945	0.869	3.31E-23	10	Rps9

3.23E-27	0.970331219	0.305	0.11	4.26E-23	10	Ly86
5.67E-27	-1.824410038	0.089	0.399	7.48E-23	10	Hist1h2ap
8.57E-27	0.953364725	0.305	0.112	1.13E-22	10	Mndal
6.17E-26	0.399574899	0.942	0.84	8.14E-22	10	Rps27rt
6.67E-26	-1.117412593	0.106	0.428	8.80E-22	10	Stmn1
1.28E-25	0.76384817	0.438	0.201	1.69E-21	10	Ahnak
4.40E-25	-1.31724931	0.158	0.461	5.81E-21	10	Pglyrp1
5.92E-25	-1.294193842	0.038	0.332	7.81E-21	10	Top2a
8.03E-25	-1.264479725	0.048	0.347	1.06E-20	10	2810417H13Rik
1.36E-24	-1.927027311	0.815	0.936	1.79E-20	10	Camp
9.35E-24	-1.993537466	0.979	0.995	1.23E-19	10	S100a9
9.39E-24	-0.939453366	0.205	0.523	1.24E-19	10	Cbx3
2.87E-23	0.782018104	0.425	0.211	3.78E-19	10	Gm2a
5.66E-23	0.910969836	0.336	0.142	7.46E-19	10	Trim30a
5.73E-23	0.840357452	0.394	0.186	7.56E-19	10	Mpeg1
1.92E-22	-1.904381147	1	0.999	2.53E-18	10	S100a8
3.20E-22	0.826391977	0.329	0.141	4.22E-18	10	Tpd52
7.92E-22	0.97898484	0.301	0.124	1.05E-17	10	Ifi203
1.16E-21	0.313003913	1	0.936	1.53E-17	10	Rpl37a
4.07E-21	-1.190239374	0.021	0.269	5.37E-17	10	Gm10282
1.05E-20	0.35942027	0.973	0.917	1.38E-16	10	Fau
1.26E-20	-1.959809456	0.736	0.855	1.66E-16	10	Ngp
1.37E-20	0.769028706	0.445	0.241	1.81E-16	10	Tmcc1
6.18E-20	0.711338132	0.408	0.201	8.16E-16	10	Fn1
2.70E-19	0.777177846	0.507	0.332	3.56E-15	10	Ifitm2
3.66E-19	0.86879728	0.284	0.12	4.83E-15	10	Rgs2
4.22E-19	0.516385371	0.342	0.149	5.57E-15	10	Cd74
2.26E-18	0.794067878	0.414	0.232	2.98E-14	10	Slfn2
2.31E-18	-1.024564821	0.062	0.307	3.05E-14	10	Cd24a
9.70E-18	-0.798233461	0.113	0.38	1.28E-13	10	Tuba1b
1.33E-17	0.720728609	0.479	0.311	1.76E-13	10	Gm10116

3.57E-17	0.720107683	0.483	0.308	4.71E-13	10	Ptprc
4.91E-17	0.773743453	0.421	0.25	6.48E-13	10	Cebpb
5.95E-17	0.836147887	0.274	0.121	7.85E-13	10	Ctsc
1.37E-16	0.288129697	0.962	0.878	1.81E-12	10	Rps14
9.96E-16	0.303353029	0.962	0.873	1.31E-11	10	Rps27a
1.82E-15	-0.455183495	0.664	0.798	2.40E-11	10	Ppia
2.36E-15	-0.696852526	0.106	0.347	3.12E-11	10	Nucks1
2.68E-15	0.368167956	0.747	0.638	3.54E-11	10	Fth1
5.28E-15	0.381400211	0.842	0.789	6.97E-11	10	Gm9843
5.88E-15	0.6643434	0.503	0.351	7.76E-11	10	H2-K1
1.12E-14	0.702313777	0.318	0.165	1.48E-10	10	Sp100
1.46E-14	-0.808632684	0.202	0.44	1.93E-10	10	Anxa1
1.64E-14	-0.625607117	0.168	0.42	2.16E-10	10	Anp32b
1.67E-14	0.813371878	0.267	0.128	2.20E-10	10	Mcl1
2.47E-14	0.512659688	0.589	0.444	3.25E-10	10	Alox5ap
2.47E-14	0.399862221	0.818	0.741	3.26E-10	10	Gpx1
4.70E-14	0.26730978	0.979	0.869	6.20E-10	10	Rpl18a
5.50E-14	0.356522561	0.846	0.771	7.25E-10	10	H3f3a
5.98E-14	-0.610869977	0.147	0.39	7.89E-10	10	Lbr
6.80E-14	0.48998334	0.634	0.495	8.97E-10	10	Cybb
1.12E-13	0.353977518	0.825	0.69	1.47E-09	10	Rpl10
1.16E-13	0.695952389	0.387	0.238	1.53E-09	10	Fxyd5
1.39E-13	-1.258075378	0.086	0.282	1.84E-09	10	Prtn3
2.11E-13	0.494284482	0.63	0.523	2.79E-09	10	Coro1a
2.80E-13	0.637187092	0.483	0.347	3.69E-09	10	Msrb1
3.53E-13	0.326442719	0.86	0.739	4.66E-09	10	Rpl24
3.71E-13	-0.528455994	0.147	0.387	4.89E-09	10	H2afv
3.85E-13	-0.602004881	0.116	0.343	5.08E-09	10	Irf2bp2
5.44E-13	0.621224768	0.373	0.214	7.17E-09	10	F13a1
6.01E-13	-0.521216925	0.339	0.593	7.93E-09	10	S100a11
6.64E-13	-0.645329897	0.12	0.337	8.76E-09	10	Ap3s1

8.05E-13	-0.602564087	0.151	0.376	1.06E-08	10	Ran
8.13E-13	0.592977387	0.466	0.309	1.07E-08	10	Lsp1
1.04E-12	-0.619301417	0.175	0.407	1.38E-08	10	Dstn
2.34E-12	-0.516190159	0.086	0.29	3.08E-08	10	Hnrnpab
3.22E-12	0.603341181	0.517	0.406	4.25E-08	10	Atox1
3.66E-12	0.619107553	0.305	0.163	4.83E-08	10	Pld4
3.71E-12	-1.608598997	0.079	0.255	4.90E-08	10	Elane
5.29E-12	0.311918811	0.887	0.79	6.98E-08	10	Rps11
6.28E-12	0.368543847	0.784	0.704	8.29E-08	10	Tpt1
1.01E-11	-0.658480195	0.168	0.379	1.33E-07	10	Myh9
1.62E-11	0.454006757	0.627	0.532	2.13E-07	10	Mrpl33
1.64E-11	0.566575296	0.346	0.205	2.16E-07	10	Ctsb
2.51E-11	0.5659737	0.39	0.248	3.31E-07	10	Btg1
2.86E-11	-0.526593631	0.13	0.336	3.77E-07	10	Cks2
3.22E-11	0.283453735	0.887	0.801	4.25E-07	10	Rpl34
3.77E-11	-0.613072198	0.377	0.561	4.98E-07	10	Chil3
4.38E-11	-0.541436877	0.24	0.461	5.77E-07	10	Atpif1
4.65E-11	-0.452098662	0.247	0.48	6.13E-07	10	Txn1
5.52E-10	0.625865767	0.26	0.143	7.29E-06	10	Capg
6.02E-10	-0.440486647	0.175	0.382	7.94E-06	10	Srsf2
6.20E-10	-1.083477625	0.113	0.277	8.17E-06	10	Mpo
6.33E-10	0.574635031	0.462	0.363	8.35E-06	10	Capza2
7.13E-10	0.619049931	0.356	0.237	9.41E-06	10	Zfp36l2
7.18E-10	0.46983203	0.538	0.423	9.47E-06	10	Prdx1
8.26E-10	0.515912909	0.38	0.25	1.09E-05	10	Anxa2
1.46E-09	-0.419361422	0.349	0.57	1.92E-05	10	Calm2
1.71E-09	-0.510072594	0.151	0.334	2.26E-05	10	Hsp90aa1
2.16E-09	-0.444909383	0.178	0.377	2.85E-05	10	Dek
3.01E-09	-0.298872922	0.705	0.825	3.98E-05	10	H2afz
3.13E-09	-0.469712148	0.096	0.264	4.13E-05	10	Alyref
3.19E-09	0.669717495	0.428	0.328	4.21E-05	10	Notch2

3.37E-09	0.626116926	0.318	0.206	4.44E-05	10	Cox7a2l
5.76E-09	0.511016615	0.459	0.359	7.59E-05	10	Serp1
1.01E-08	-0.302719693	0.366	0.577	0.000132581	10	Hnrnpa2b1
1.43E-08	-0.467776397	0.168	0.343	0.000188534	10	Hnrnpa1
1.77E-08	0.489055407	0.377	0.252	0.000232961	10	Gm9844
2.60E-08	0.629863022	0.308	0.206	0.000343149	10	Lamp1
3.17E-08	-0.462319796	0.175	0.349	0.000417963	10	Set
3.60E-08	0.58737678	0.322	0.219	0.000474899	10	Spi1
3.80E-08	-0.382953328	0.199	0.393	0.000501161	10	1810037I17Rik
3.83E-08	-0.350127259	0.332	0.543	0.000504962	10	Atp5k
5.47E-08	-0.374319625	0.199	0.386	0.000721537	10	Eif5a
5.51E-08	-0.407098075	0.161	0.333	0.000726361	10	Ranbp1
5.59E-08	0.503621737	0.476	0.394	0.000737725	10	Actr3
7.79E-08	-0.394810973	0.134	0.294	0.001027822	10	Tagln2
1.01E-07	0.547928283	0.387	0.293	0.0013351	10	Cdk2ap2
1.25E-07	0.607044764	0.253	0.155	0.001647409	10	Itgal
1.33E-07	0.536103939	0.325	0.225	0.001757657	10	Rrbp1
1.53E-07	0.520547236	0.384	0.287	0.002016052	10	Rsrp1
1.53E-07	0.311856679	0.719	0.64	0.002022233	10	Rpl18
1.82E-07	0.380420849	0.551	0.463	0.002399135	10	Rpl10-ps3
2.94E-07	-0.302032993	0.168	0.34	0.003880791	10	Lsm5
3.42E-07	-0.38022056	0.257	0.436	0.004509513	10	Serbp1
4.69E-07	-0.355965409	0.586	0.71	0.006184181	10	mt-Cytb
5.15E-07	0.535007292	0.346	0.254	0.006789818	10	Ucp2
5.43E-07	0.390833805	0.521	0.445	0.007163746	10	Psma7
5.49E-07	0.297412037	0.692	0.664	0.007241745	10	Cox4i1
6.14E-07	0.40547216	0.534	0.48	0.008099005	10	Uqcrh
6.21E-07	0.289055242	0.729	0.682	0.008190276	10	Cyba
6.74E-07	-0.404271368	0.144	0.291	0.00889513	10	Eef1g
7.61E-07	-0.264298358	0.209	0.394	0.010044031	10	Eif2s2
9.31E-07	0.477354685	0.377	0.29	0.012281052	10	Laptm5

9.48E-07	0.521573266	0.366	0.276	0.012501723	10	Rap1b
2.22E-06	-0.285808955	0.182	0.345	0.029271464	10	Srsf3
2.69E-06	-0.272724466	0.349	0.528	0.035503024	10	Snrpg
2.73E-06	0.321992874	0.654	0.648	0.036071441	10	Itm2b
2.77E-06	0.440581503	0.466	0.4	0.036568161	10	Dbi
4.61E-06	-0.266326133	0.253	0.435	0.060798362	10	Ndufb7
6.26E-06	-0.330479758	0.322	0.485	0.082559954	10	F630028O10Rik
9.78E-06	0.367664426	0.572	0.555	0.128984299	10	Calm1
1.21E-05	-0.396179091	0.154	0.285	0.159461936	10	Slpi
1.25E-05	-0.281707185	0.127	0.255	0.165136679	10	Sec11c
1.25E-05	-0.320621968	0.342	0.491	0.165239038	10	Rpl12
1.45E-05	0.518587462	0.298	0.219	0.191507949	10	Gltscr2
1.55E-05	0.511167128	0.271	0.192	0.203979007	10	Emb
1.65E-05	-0.346604316	0.442	0.56	0.217691998	10	Rps2
2.21E-05	0.522555031	0.312	0.238	0.29145759	10	Msn
2.25E-05	0.477081784	0.267	0.186	0.297429541	10	Prr13
2.33E-05	0.283341123	0.699	0.692	0.307473684	10	Sh3bgrl3
3.11E-05	-0.262807134	0.161	0.293	0.409608043	10	Ndufv3
3.73E-05	0.420789482	0.394	0.325	0.491614791	10	Cd53
5.13E-05	0.326360631	0.521	0.488	0.676303574	10	Arpc2
5.27E-05	0.433404538	0.38	0.321	0.695224208	10	Slc25a5
5.47E-05	0.39472494	0.634	0.614	0.722143939	10	Ly6c2
6.28E-05	0.466548204	0.339	0.274	0.8290116	10	Rac1
7.08E-05	0.574417371	0.257	0.189	0.93365189	10	Chmp4b
8.54E-05	0.379307724	0.421	0.369	1	10	Arpc5
9.94E-05	-0.432020432	0.38	0.482	1	10	Lgals1
0.00012027	-0.274382972	0.366	0.512	1	10	Rpl22l1
0.00012974	0.494348582	0.301	0.241	1	10	Rap1a
0.00015431	0.559303538	0.257	0.191	1	10	Klf13
0.00022038	-0.326125658	0.182	0.293	1	10	Lrrc58
0.0002478	0.346182892	0.568	0.544	1	10	Hspa8

0.00028663	0.446501386	0.284	0.222	1	10	Psme2	
0.00030134	0.443831737	0.26	0.197	1	10	Hmha1	
0.00033025	-0.279382271	0.161	0.268	1	10	Ncf1	
0.000387	0.254228639	0.64	0.649	1	10	Atp5l	
0.00044378	0.43059448	0.271	0.209	1	10	Grn	
0.0004772	-0.262051903	0.579	0.666	1	10	Rps20	
0.00049074	0.365164628	0.473	0.443	1	10	Arpc1b	
0.00064145	-0.253023524	0.562	0.648	1	10	Rpl36a	
0.00085801	0.391211649	0.397	0.364	1	10	Emp3	
0.00105758	0.273732805	0.565	0.574	1	10	Rpl36al	
0.00120501	0.460830231	0.274	0.223	1	10	Ptpn6	
0.00122362	0.411069009	0.301	0.249	1	10	Psmb8	
0.00173392	0.398886417	0.384	0.354	1	10	Fam49b	
0.00175488	0.4013379	0.257	0.204	1	10	Akap13	
0.00187265	0.301274319	0.469	0.45	1	10	Naca	
0.00269493	0.349151554	0.342	0.303	1	10	Taldo1	
0.00440307	0.314467998	0.438	0.431	1	10	Serinc3	
0.00492829	0.389509963	0.346	0.325	1	10	Eif3f	
0.00558372	0.337921863	0.425	0.417	1	10	Cdc42	
0.00562546	0.439363868	0.253	0.214	1	10	Erp29	
0.00629879	0.304117296	0.418	0.403	1	10	Pfdn5	
0.00636643	0.389755255	0.291	0.254	1	10	Vim	
0.00665193	0.40424636	0.281	0.242	1	10	Scand1	
0.00855571	0.255092808	0.452	0.438	1	10	Gnb2l1	
4.78E-149	1.542749756	0.549	0.076	6.30E-145	11	Runx2	**plasmacytoid DC**
4.07E-137	1.50964618	0.623	0.11	5.37E-133	11	Tcf4	
6.04E-115	1.387772599	0.698	0.165	7.97E-111	11	Bst2	
2.32E-114	1.436825697	0.75	0.199	3.06E-110	11	Irf8	
2.39E-108	1.933379136	0.56	0.115	3.15E-104	11	Ly6d	
4.16E-107	1.44116729	0.425	0.061	5.49E-103	11	Cox6a2	
2.22E-97	1.293728225	0.302	0.033	2.93E-93	11	Cd7	

7.34E-81	1.222550367	0.881	0.46	9.68E-77	11	Lgals1
6.91E-80	1.010168928	0.354	0.054	9.12E-76	11	Siglech
6.04E-71	1.267315868	0.504	0.13	7.97E-67	11	Bcl11a
6.09E-41	-1.156617934	0.754	0.912	8.03E-37	11	Lyz2
5.16E-36	0.908791276	0.362	0.111	6.81E-32	11	Mef2c
1.74E-33	0.718142734	0.78	0.492	2.29E-29	11	Mbnl1
2.36E-33	0.862323721	0.373	0.124	3.11E-29	11	Dnajc7
1.56E-32	0.938059042	0.515	0.233	2.06E-28	11	Pgls
4.69E-31	-0.420905852	1	0.996	6.18E-27	11	Tmsb4x
7.35E-31	0.685172243	0.836	0.628	9.70E-27	11	Xist
4.36E-30	1.000181469	0.31	0.098	5.75E-26	11	Bmp2k
5.50E-29	0.707669812	0.325	0.102	7.26E-25	11	Rnase6
2.29E-27	0.318160985	0.996	0.978	3.02E-23	11	mt-Co1
4.09E-26	0.392359193	0.974	0.854	5.39E-22	11	mt-Atp6
1.19E-24	0.520276507	0.847	0.634	1.57E-20	11	Fth1
4.61E-24	-1.150184296	0.265	0.565	6.08E-20	11	Chil3
2.13E-23	0.69366982	0.474	0.219	2.81E-19	11	Sell
3.80E-23	0.702898323	0.582	0.335	5.02E-19	11	H2afy
3.42E-22	0.457144818	0.907	0.7	4.51E-18	11	Rpl31
4.29E-22	0.589202491	0.716	0.458	5.66E-18	11	Ly6e
4.86E-21	0.303686852	1	0.997	6.41E-17	11	Malat1
3.67E-20	0.380568629	0.903	0.674	4.84E-16	11	Plac8
6.05E-20	0.681836246	0.429	0.211	7.98E-16	11	Selplg
5.73E-19	0.766298085	0.373	0.174	7.56E-15	11	Kctd12
9.02E-19	0.553723854	0.41	0.186	1.19E-14	11	Mpeg1
2.80E-17	-0.652522655	0.422	0.655	3.69E-13	11	Prdx5
2.94E-17	-1.090569554	0.996	0.999	3.88E-13	11	S100a8
5.31E-17	0.455868791	0.851	0.737	7.01E-13	11	H3f3b
5.47E-17	-0.997459937	0.328	0.569	7.21E-13	11	Ifitm3
9.90E-17	0.266345581	0.989	0.953	1.31E-12	11	mt-Co3
1.76E-16	0.694442297	0.269	0.111	2.33E-12	11	Hmgn1

3.93E-16	0.66400517	0.254	0.101	5.18E-12	11	Marcks
2.18E-15	-0.574865514	0.493	0.692	2.87E-11	11	Cyba
3.87E-15	0.351386544	0.944	0.803	5.10E-11	11	Rpl13a
1.06E-14	0.538256472	0.526	0.307	1.39E-10	11	Lsp1
1.14E-14	-0.833903483	0.194	0.439	1.50E-10	11	Anxa1
1.42E-14	0.759288569	0.254	0.11	1.88E-10	11	Rexo2
2.09E-14	0.315731315	0.948	0.788	2.75E-10	11	Rps11
2.93E-14	-0.950924072	0.243	0.456	3.87E-10	11	Pglyrp1
4.12E-14	0.371860762	0.757	0.534	5.44E-10	11	Psap
4.28E-14	-0.746007109	0.179	0.406	5.64E-10	11	Dstn
6.77E-14	-1.115767435	0.47	0.637	8.93E-10	11	Wfdc21
1.20E-13	0.362877022	0.888	0.687	1.58E-09	11	Rpl10
1.20E-13	-1.039780589	0.157	0.369	1.58E-09	11	Ifitm6
1.96E-13	-0.594157038	0.604	0.736	2.59E-09	11	Cd52
2.92E-13	0.527293382	0.388	0.204	3.85E-09	11	Ctsb
4.86E-13	-0.810814764	0.157	0.373	6.42E-09	11	Lst1
9.47E-13	0.316258324	0.937	0.838	1.25E-08	11	Ptma
1.13E-12	0.324760015	0.922	0.787	1.49E-08	11	Ppia
1.49E-12	0.612462087	0.302	0.144	1.97E-08	11	Trim30a
2.31E-12	0.361803145	0.832	0.691	3.05E-08	11	Oaz1
3.18E-12	-0.671595988	0.284	0.486	4.20E-08	11	F630028O10Rik
5.44E-12	0.481183487	0.254	0.113	7.18E-08	11	Ly86
6.06E-12	-1.041080002	0.407	0.565	7.99E-08	11	Lcn2
7.49E-12	-0.664763023	0.257	0.463	9.88E-08	11	Hp
1.29E-11	-1.016086653	0.772	0.853	1.70E-07	11	Ngp
1.37E-11	-0.700617612	0.172	0.374	1.80E-07	11	Gsr
1.58E-11	0.323476762	0.866	0.719	2.08E-07	11	Rps4x
2.38E-11	0.60435346	0.541	0.407	3.14E-07	11	Stmn1
2.43E-11	-1.051574192	0.918	0.932	3.20E-07	11	Camp
3.44E-11	0.424011286	0.802	0.686	4.54E-07	11	Hmgb1
3.89E-11	-1.056743318	1	0.994	5.13E-07	11	S100a9

4.08E-11	0.315230757	0.836	0.698	5.39E-07	11	mt-Cytb
5.14E-11	0.391912064	0.269	0.126	6.79E-07	11	Ifi203
6.21E-11	0.266079843	0.94	0.778	8.19E-07	11	Rps5
6.70E-11	0.581635936	0.366	0.214	8.84E-07	11	Fgfr1op2
3.87E-10	-0.748896737	0.175	0.347	5.11E-06	11	Ifitm2
6.65E-10	0.375275648	0.593	0.424	8.77E-06	11	Serinc3
1.46E-09	-0.443609181	0.534	0.671	1.92E-05	11	Arhgdib
1.48E-09	0.487054223	0.287	0.153	1.96E-05	11	St8sia4
2.09E-09	0.388044591	0.362	0.206	2.76E-05	11	Ahnak
2.15E-09	0.507654188	0.336	0.198	2.84E-05	11	Zfp706
2.48E-09	0.52840221	0.28	0.151	3.28E-05	11	Ctsh
5.67E-09	-0.671947573	0.287	0.445	7.47E-05	11	Lgals3
9.63E-09	-0.605546076	0.194	0.36	0.000127079	11	Msrb1
1.36E-08	0.410917726	0.597	0.461	0.000179854	11	Cd47
2.17E-08	0.392057341	0.489	0.334	0.000286833	11	Hsp90b1
2.32E-08	-0.549800957	0.362	0.507	0.000305802	11	Cybb
2.85E-08	0.316544768	0.817	0.701	0.000375345	11	Sub1
3.09E-08	0.263039336	0.877	0.768	0.000406973	11	Rps3a1
4.45E-08	-0.643662104	0.149	0.297	0.00058666	11	Mgst1
4.45E-08	-0.855918899	0.437	0.546	0.000586844	11	Ltf
9.04E-08	0.509867552	0.276	0.158	0.001192683	11	Sepw1
1.07E-07	-0.566021217	0.187	0.333	0.001417653	11	Ap3s1
1.15E-07	0.456187398	0.403	0.273	0.001517625	11	Atp6v1g1
1.25E-07	0.33595548	0.612	0.461	0.001649379	11	Rpl10-ps3
1.38E-07	-0.64972393	0.123	0.263	0.001816266	11	Cebpb
1.41E-07	0.285975085	0.754	0.627	0.001855231	11	Cst3
1.80E-07	0.371779364	0.597	0.451	0.002380632	11	Dynll1
2.60E-07	-0.307112255	0.698	0.784	0.003426878	11	Shfm1
3.05E-07	0.308535475	0.515	0.359	0.004017048	11	Emp3
3.60E-07	0.432485008	0.254	0.144	0.004743685	11	Psmb9
3.60E-07	0.439760265	0.254	0.145	0.004753848	11	Cd48

5.44E-07	0.272786449	0.709	0.564	0.007178258	11	mt-Nd4
8.40E-07	0.34073301	0.5	0.371	0.011077813	11	Btf3
8.51E-07	0.267197725	0.739	0.594	0.01122005	11	Rpl3
8.71E-07	0.369686183	0.466	0.336	0.011488888	11	Set
1.20E-06	0.338247536	0.284	0.164	0.015777283	11	Pld4
1.46E-06	0.290043213	0.743	0.633	0.019235005	11	Sec61g
1.93E-06	-0.543756009	0.153	0.28	0.025411262	11	Aldh2
2.25E-06	0.320945772	0.343	0.219	0.029737841	11	Psme2
2.61E-06	-0.510420371	0.142	0.271	0.03444961	11	Clec4a2
2.77E-06	0.471744649	0.31	0.206	0.036587765	11	Bzw1
2.84E-06	0.394791551	0.31	0.201	0.037509742	11	Tacc1
3.06E-06	0.301975076	0.619	0.503	0.040328994	11	Cbx3
3.76E-06	0.421797828	0.265	0.163	0.049608618	11	Clk1
4.43E-06	-0.481324195	0.172	0.302	0.058490583	11	Cd24a
5.09E-06	0.429802803	0.463	0.372	0.067117865	11	H2afv
5.23E-06	0.32257268	0.31	0.197	0.068960541	11	Psme1
5.30E-06	-1.111405223	0.138	0.252	0.069922221	11	Elane
5.47E-06	0.418649697	0.287	0.184	0.072122292	11	Ramp1
5.87E-06	0.469772574	0.332	0.216	0.077484449	11	Gm2a
6.88E-06	0.309487898	0.507	0.385	0.090813483	11	Smdt1
9.08E-06	0.34099728	0.41	0.3	0.119739792	11	Tsix
1.00E-05	-0.406277671	0.485	0.586	0.132427649	11	S100a11
1.20E-05	0.264942026	0.586	0.465	0.158168005	11	Rps18-ps3
1.48E-05	0.369103832	0.299	0.201	0.194973179	11	Nono
1.48E-05	0.48069102	0.563	0.494	0.195228336	11	Tubb5
1.52E-05	0.268253107	0.586	0.464	0.201162272	11	Gm8730
1.54E-05	-0.446580254	0.183	0.297	0.20275494	11	Pkm
1.57E-05	-0.519901091	0.16	0.271	0.206832404	11	Cotl1
1.57E-05	-0.395382232	0.257	0.39	0.207447417	11	1810037I17Rik
1.61E-05	0.376234816	0.306	0.206	0.21230529	11	Tram1
2.04E-05	0.341598697	0.369	0.259	0.269229956	11	Gnas

2.11E-05	0.396525375	0.388	0.283	0.2781623	11	Tagln2
2.80E-05	0.366298817	0.28	0.183	0.368967536	11	Pnisr
2.81E-05	0.437128275	0.272	0.18	0.371184033	11	Erdr1
4.74E-05	0.254657297	0.571	0.474	0.625518053	11	Morf4l1
4.79E-05	0.310449668	0.31	0.207	0.632459846	11	Grn
4.90E-05	-0.44299193	0.153	0.26	0.646359693	11	Flna
4.99E-05	-0.522032502	0.194	0.298	0.658398161	11	Sat1
6.09E-05	-0.333518652	0.366	0.474	0.802728522	11	Txn1
6.46E-05	-0.385247738	0.164	0.275	0.851762513	11	Aldoa
6.64E-05	0.412398139	0.254	0.166	0.875522498	11	Cd164
6.79E-05	0.298497125	0.347	0.244	0.896079837	11	Rbm25
7.56E-05	0.299219988	0.317	0.22	0.996671162	11	Rpgrip1
8.14E-05	0.332970791	0.369	0.273	1	11	Arglu1
8.49E-05	0.386466204	0.254	0.167	1	11	Tmed3
8.81E-05	-0.910874987	0.183	0.274	1	11	Mpo
0.00011898	0.332338931	0.358	0.265	1	11	Tmed2
0.0001198	0.330782918	0.313	0.215	1	11	Smim14
0.00014078	-0.41397125	0.164	0.263	1	11	Aprt
0.00015683	-0.78670611	0.187	0.276	1	11	Prtn3
0.00016841	0.3406008	0.351	0.259	1	11	Snrpd2
0.00017381	0.326269902	0.373	0.28	1	11	Luc7l2
0.00017656	0.314684922	0.429	0.332	1	11	Nucks1
0.00018711	-0.35993173	0.358	0.449	1	11	Gmfg
0.0001954	-0.397720585	0.175	0.273	1	11	Gpi1
0.00021049	0.332003489	0.414	0.321	1	11	Ranbp1
0.00022675	0.355380417	0.444	0.365	1	11	Dek
0.00026395	-0.337381358	0.216	0.323	1	11	Gm10116
0.00028637	0.314612989	0.422	0.329	1	11	Irf2bp2
0.00028745	0.35906498	0.366	0.28	1	11	Cmtm7
0.00031297	0.291175689	0.358	0.269	1	11	Ywhae
0.00031893	0.276454107	0.254	0.168	1	11	Sp100

0.00039291	-0.412181533	0.172	0.261	1	11	Gm10282	
0.000399	0.508005529	0.396	0.331	1	11	2810417H13Rik	
0.0003995	0.302343913	0.269	0.189	1	11	Hnrnpl	
0.00046596	-0.32150437	0.302	0.407	1	11	Dbi	
0.00050217	0.293299946	0.31	0.226	1	11	Csnk1a1	
0.00056176	0.319137991	0.265	0.189	1	11	Chmp4b	
0.00064727	-0.256040005	0.321	0.428	1	11	Tkt	
0.00069151	0.330942263	0.261	0.184	1	11	Fkbp2	
0.00101941	0.286042178	0.291	0.213	1	11	Erp29	
0.00103094	0.332385641	0.325	0.248	1	11	Psmb8	
0.00106019	-0.359464088	0.526	0.59	1	11	Hmgn2	
0.00114803	0.29891901	0.373	0.299	1	11	Erh	
0.00119878	0.352233196	0.31	0.236	1	11	Cks1b	
0.00185563	0.398936781	0.388	0.316	1	11	Top2a	
0.00188561	0.254666686	0.328	0.251	1	11	Btg1	
0.00197644	-0.391064455	0.175	0.253	1	11	Tmcc1	
0.00204134	0.294604306	0.295	0.222	1	11	Lsm4	
0.00213801	0.282636873	0.418	0.354	1	11	Gm42418	
0.00222751	-0.321709286	0.179	0.261	1	11	Lmo4	
0.00225359	0.263755595	0.28	0.208	1	11	Fus	
0.00455531	0.622177659	0.422	0.383	1	11	Hist1h2ap	
0.00532494	0.253716717	0.388	0.318	1	11	Hnrnpu	
0.00597122	0.26074753	0.261	0.197	1	11	Ppp3ca	
0.00609923	-0.282511044	0.291	0.362	1	11	Gpx4	
0.00660094	0.265720693	0.302	0.244	1	11	Hnrnph1	
0.006946	0.278212573	0.302	0.237	1	11	Pycard	
0.00730491	0.251794077	0.261	0.203	1	11	Rasgrp2	
0.00731161	0.251962408	0.332	0.267	1	11	Abracl	
0.00749488	0.271550086	0.257	0.196	1	11	AI662270	
0.00821235	-0.333115176	0.205	0.277	1	11	Ccr2	
3.00E-219	1.491967393	0.851	0.125	3.96E-215	12	Ctsg	**Mac**

6.72E-197	1.77570706	0.977	0.24	8.86E-193	12	Mpo
1.87E-180	1.550359175	0.969	0.243	2.46E-176	12	Prtn3
1.52E-170	1.532645678	0.927	0.218	2.01E-166	12	Elane
5.01E-159	1.338895346	0.9	0.204	6.61E-155	12	Mif
6.87E-99	0.828477808	1	0.829	9.06E-95	12	Rps8
7.56E-97	0.988845431	0.812	0.22	9.97E-93	12	Calr
2.87E-96	0.719896982	0.479	0.077	3.79E-92	12	Igfbp4
4.73E-96	0.854504589	0.659	0.141	6.24E-92	12	Ms4a3
7.54E-87	0.632074861	1	0.954	9.94E-83	12	Rpl41
1.94E-85	0.82802024	0.755	0.199	2.56E-81	12	Etfb
7.01E-85	0.822354441	0.778	0.206	9.25E-81	12	Hspe1
2.11E-83	0.54330433	1	0.975	2.79E-79	12	Rps29
5.77E-81	0.811026491	0.996	0.645	7.61E-77	12	Rps12
9.98E-80	0.589520974	0.54	0.109	1.32E-75	12	Dctpp1
1.96E-77	0.60771937	1	0.897	2.59E-73	12	Rps28
6.60E-75	0.780808352	0.992	0.629	8.70E-71	12	Rpl36a
4.78E-74	0.838291197	0.966	0.436	6.31E-70	12	Rps12-ps3
2.19E-73	0.734161981	1	0.709	2.89E-69	12	Rps17
3.95E-72	0.85401621	0.923	0.36	5.20E-68	12	Npm1
9.53E-71	0.76329113	0.874	0.291	1.26E-66	12	Atp5g1
1.30E-70	0.741433506	0.674	0.181	1.71E-66	12	Srgn
1.97E-70	0.767673306	0.996	0.536	2.60E-66	12	Rps2
5.34E-70	0.591102358	1	0.898	7.05E-66	12	Rpl37
8.10E-69	0.726234345	0.774	0.233	1.07E-64	12	Ncl
1.93E-68	0.693122997	0.996	0.711	2.55E-64	12	Rplp1
4.19E-68	0.693558892	1	0.672	5.52E-64	12	Rpl14
1.36E-67	0.68845052	1	0.755	1.79E-63	12	Rpl36
1.38E-67	0.620708192	1	0.843	1.82E-63	12	Rpl38
1.55E-67	0.782799454	0.816	0.272	2.05E-63	12	Pdcd4
4.58E-67	0.609524659	1	0.852	6.04E-63	12	Rpl35
9.18E-67	-0.742497212	1	0.996	1.21E-62	12	Tmsb4x

1.29E-66	0.663601561	0.996	0.743	1.71E-62	12	Gm10076
1.32E-66	0.601238821	0.494	0.108	1.74E-62	12	Pebp1
3.18E-66	0.70034865	0.996	0.648	4.20E-62	12	Rps20
1.12E-65	0.624251289	0.575	0.14	1.47E-61	12	Rpn1
1.23E-65	0.631097099	0.322	0.052	1.63E-61	12	Dennd5b
1.32E-65	0.647335494	0.67	0.184	1.74E-61	12	Ssr4
3.00E-65	0.577583571	0.437	0.089	3.96E-61	12	Hspa9
4.83E-65	0.757172136	0.973	0.464	6.37E-61	12	Rpl12
7.81E-65	0.572784146	0.536	0.125	1.03E-60	12	Npm3
8.60E-65	0.535764957	0.28	0.04	1.14E-60	12	Cst7
1.31E-64	0.70961102	0.996	0.671	1.72E-60	12	Plac8
1.63E-64	0.640960289	0.996	0.803	2.15E-60	12	Rps23
1.88E-64	0.599918422	1	0.862	2.48E-60	12	Rpl32
2.17E-64	0.601775022	1	0.836	2.86E-60	12	Ptma
3.32E-64	0.532819576	0.46	0.096	4.38E-60	12	Hspd1
4.05E-64	0.67947472	0.483	0.108	5.34E-60	12	Gstm1
4.58E-64	0.767693179	0.927	0.397	6.04E-60	12	Hsp90ab1
4.68E-64	0.567647016	0.429	0.087	6.18E-60	12	Nop56
1.18E-63	0.627888661	1	0.769	1.56E-59	12	Rplp2
1.43E-63	0.485612555	0.299	0.046	1.89E-59	12	Cd93
2.09E-63	0.641883946	0.713	0.206	2.75E-59	12	Nme1
3.22E-63	0.658095181	0.82	0.261	4.25E-59	12	Cmtm7
3.40E-63	0.558177086	0.429	0.087	4.48E-59	12	Pdia4
3.60E-63	0.719066787	0.996	0.592	4.75E-59	12	Rps21
1.97E-62	0.573979575	0.571	0.141	2.59E-58	12	Sdf2l1
3.27E-62	0.704102266	0.877	0.314	4.31E-58	12	Nop10
3.48E-62	0.585924272	0.67	0.185	4.59E-58	12	Hspa5
6.50E-62	0.70052501	0.785	0.256	8.58E-58	12	Bola2
9.22E-62	0.428561846	0.31	0.05	1.22E-57	12	Phpt1
9.56E-62	0.630558355	0.517	0.124	1.26E-57	12	Snhg9
3.72E-61	0.54155163	1	0.892	4.91E-57	12	Rps19

5.16E-61	0.435636662	0.276	0.041	6.81E-57	12	Phgdh
5.35E-61	0.62483578	1	0.767	7.05E-57	12	Rpl39
8.72E-61	0.69544455	0.46	0.104	1.15E-56	12	Mt1
8.82E-61	0.653386452	1	0.712	1.16E-56	12	Rps26
2.09E-60	0.644355702	0.536	0.133	2.76E-56	12	Nhp2
2.10E-60	0.58277939	1	0.829	2.78E-56	12	Rps18
3.56E-60	0.453409955	0.467	0.102	4.70E-56	12	Pdia6
4.44E-60	0.636281648	0.716	0.214	5.86E-56	12	Usp50
4.98E-60	-1.727604368	0.939	0.904	6.57E-56	12	Lyz2
9.49E-60	0.536211553	1	0.867	1.25E-55	12	Rpl35a
2.53E-59	0.515443094	0.406	0.084	3.33E-55	12	Pa2g4
1.16E-58	0.585235077	0.567	0.148	1.53E-54	12	G3bp1
1.58E-58	0.533698108	0.705	0.204	2.09E-54	12	Krtcap2
6.92E-58	0.535906047	1	0.872	9.12E-54	12	Rps27a
1.39E-57	0.563364673	1	0.811	1.83E-53	12	Rpl23a
2.47E-57	0.615571101	1	0.73	3.26E-53	12	Rpl23
4.00E-57	0.670349007	0.82	0.291	5.27E-53	12	Cycs
5.13E-57	0.457049955	0.579	0.148	6.77E-53	12	P4hb
8.77E-57	0.741098836	0.946	0.487	1.16E-52	12	Rpl22l1
2.08E-56	0.597172938	0.805	0.262	2.74E-52	12	Eef1g
3.57E-56	0.578074598	1	0.763	4.72E-52	12	Rps3a1
3.90E-56	0.68515955	0.874	0.335	5.14E-52	12	Snrpf
4.19E-56	0.663443253	0.977	0.574	5.53E-52	12	Wdr89
4.77E-56	0.617572897	0.586	0.164	6.29E-52	12	Myb
5.18E-56	0.512243475	0.621	0.17	6.83E-52	12	Ramp1
5.22E-56	0.492039331	0.579	0.152	6.88E-52	12	Slirp
9.72E-56	0.617122495	1	0.661	1.28E-51	12	Gm2000
1.90E-55	0.65813303	0.977	0.559	2.51E-51	12	Rpl10a
2.26E-55	0.459741791	0.341	0.065	2.98E-51	12	Cct3
2.56E-55	0.651372259	0.858	0.324	3.38E-51	12	H2afy
5.27E-55	0.5896393	1	0.737	6.95E-51	12	Rps6

5.68E-55	0.665515138	0.962	0.418	7.49E-51	12	Gm10073
1.22E-54	0.659458311	0.862	0.32	1.61E-50	12	Set
2.02E-54	0.702468306	0.897	0.383	2.66E-50	12	Rpl36-ps3
2.18E-54	0.555746649	0.525	0.138	2.87E-50	12	Nkg7
1.09E-53	0.612055661	0.996	0.667	1.43E-49	12	Rpl27
1.34E-53	0.588993315	1	0.716	1.77E-49	12	Rps25
6.56E-53	0.469266416	1	0.936	8.65E-49	12	Rpl37a
7.53E-53	0.572371695	0.299	0.055	9.93E-49	12	Srm
1.19E-52	0.681276357	0.808	0.304	1.57E-48	12	Ranbp1
1.84E-52	0.580235487	1	0.71	2.42E-48	12	Rpl6
2.07E-52	0.614870159	0.797	0.281	2.73E-48	12	Erh
3.96E-52	0.446028551	0.284	0.049	5.23E-48	12	Pcyox1l
5.11E-52	0.558674238	1	0.738	6.74E-48	12	Rps15a
5.15E-52	0.695737444	0.797	0.306	6.80E-48	12	Hsp90aa1
7.72E-52	0.553048385	0.996	0.771	1.02E-47	12	Rpl26
9.36E-52	0.445776165	0.291	0.051	1.24E-47	12	BC035044
9.74E-52	0.611901635	0.774	0.266	1.29E-47	12	Eif4a1
1.52E-51	0.593901931	0.981	0.658	2.00E-47	12	Rpl23a-ps3
1.76E-51	0.500402531	0.513	0.135	2.32E-47	12	Manf
1.81E-51	0.450021926	0.533	0.142	2.39E-47	12	Cdk6
2.64E-51	0.4786681	0.318	0.061	3.48E-47	12	Alas1
1.05E-50	0.575871138	1	0.709	1.39E-46	12	Rpl27a
1.98E-50	0.553123926	0.774	0.263	2.61E-46	12	Gm10269
2.02E-50	0.65817641	0.954	0.455	2.66E-46	12	Rps26-ps1
2.25E-50	0.600034745	0.996	0.632	2.97E-46	12	Gm10263
3.04E-50	0.527160467	0.521	0.141	4.01E-46	12	Ndufa12
4.60E-50	0.50759785	0.437	0.107	6.06E-46	12	Gm10053
4.70E-50	0.515335004	0.563	0.159	6.20E-46	12	Uqcc2
9.30E-50	0.584418759	0.992	0.705	1.23E-45	12	Rpl11
1.24E-49	0.62508491	0.981	0.507	1.63E-45	12	Rpl22
1.78E-49	0.642062226	0.885	0.371	2.35E-45	12	Gm8186

2.64E-49	0.655482034	0.954	0.502	3.48E-45	12	Snrpg
2.77E-49	0.478851408	0.544	0.15	3.65E-45	12	Tomm5
3.15E-49	0.434691947	0.303	0.058	4.15E-45	12	Fam162a
5.79E-49	0.485859657	0.345	0.073	7.64E-45	12	Lyar
8.24E-49	0.442901953	0.521	0.139	1.09E-44	12	1110001J03Rik
1.00E-48	0.545688461	0.728	0.243	1.32E-44	12	Snrpd2
1.53E-48	0.484017618	0.598	0.176	2.01E-44	12	Gm10320
1.64E-48	0.466613891	0.448	0.112	2.17E-44	12	Syncrip
2.08E-48	0.584779761	0.506	0.138	2.75E-44	12	Comt
3.65E-48	0.517048787	0.598	0.178	4.81E-44	12	St13
5.35E-48	0.611109099	0.889	0.364	7.06E-44	12	Eif2s2
6.37E-48	0.550759403	0.992	0.698	8.40E-44	12	Rpl28
6.96E-48	0.562293306	0.801	0.285	9.18E-44	12	Polr2l
9.98E-48	0.499550624	0.337	0.072	1.32E-43	12	C1qbp
1.62E-47	0.445949744	0.287	0.055	2.14E-43	12	Gnl3
4.03E-47	0.501945882	0.655	0.204	5.32E-43	12	Pgam1
6.83E-47	0.499145549	0.525	0.148	9.01E-43	12	Nhp2l1
1.05E-46	0.61990533	0.939	0.455	1.38E-42	12	Rpl27-ps3
1.12E-46	0.401559191	0.31	0.063	1.48E-42	12	1500011K16Rik
2.87E-46	0.54351456	0.571	0.174	3.79E-42	12	2700094K13Rik
3.96E-46	0.526798677	0.989	0.734	5.23E-42	12	Rpl24
5.01E-46	0.545427519	0.989	0.683	6.60E-42	12	Rpl10
5.17E-46	0.438898862	0.36	0.082	6.82E-42	12	Nop58
5.67E-46	0.493996448	0.594	0.182	7.48E-42	12	AI662270
1.03E-45	0.518326438	0.437	0.113	1.36E-41	12	Mrpl42
1.23E-45	0.447952181	0.437	0.112	1.63E-41	12	Fkbp3
1.92E-45	0.379442101	0.33	0.07	2.54E-41	12	Tmem147
7.53E-45	0.512206155	0.996	0.734	9.93E-41	12	Rps15
7.55E-45	0.4451246	0.36	0.082	9.96E-41	12	Atp8b4
1.21E-44	0.431553484	0.295	0.06	1.60E-40	12	Syce2
2.59E-44	0.482782064	1	0.845	3.41E-40	12	Rpl13

2.65E-44	0.589890571	0.946	0.445	3.50E-40	12	Rpl30
3.29E-44	0.520588833	0.801	0.297	4.34E-40	12	Eef1b2
3.93E-44	0.456826404	0.571	0.172	5.18E-40	12	Eif3j1
4.01E-44	0.377480452	0.379	0.091	5.29E-40	12	Mrpl36
4.89E-44	0.497468344	0.782	0.278	6.45E-40	12	Atp5g2
4.99E-44	0.466957765	0.67	0.22	6.58E-40	12	Ptges3
6.30E-44	0.444200698	0.544	0.16	8.31E-40	12	Mrpl23
8.85E-44	0.594634211	0.939	0.489	1.17E-39	12	Sec61b
1.44E-43	0.53856241	0.992	0.643	1.90E-39	12	Rps10
1.74E-43	0.566569708	0.946	0.45	2.30E-39	12	Rps18-ps3
1.89E-43	0.566176101	0.969	0.562	2.49E-39	12	Rpl15
2.22E-43	0.43967619	0.464	0.127	2.92E-39	12	Dtymk
2.74E-43	0.437523825	0.628	0.199	3.61E-39	12	Slc25a3
2.88E-43	0.58905822	0.9	0.432	3.80E-39	12	Atpif1
3.13E-43	0.524812506	0.992	0.679	4.12E-39	12	Rps7
4.47E-43	0.555391709	0.793	0.301	5.90E-39	12	Gm10036
4.95E-43	0.477253283	0.996	0.799	6.53E-39	12	Rps16
6.41E-43	0.435952341	0.598	0.187	8.46E-39	12	Zfp706
8.93E-43	0.534340173	0.797	0.307	1.18E-38	12	Ap3s1
9.28E-43	0.550177666	0.946	0.458	1.22E-38	12	Lgals1
9.58E-43	0.502230485	0.985	0.679	1.26E-38	12	Rps13
1.13E-42	0.578885736	0.816	0.329	1.49E-38	12	Cox7b
1.36E-42	0.414650547	0.333	0.076	1.79E-38	12	Fkbp4
1.67E-42	0.440483175	0.398	0.102	2.20E-38	12	Fbl
2.05E-42	0.425855727	0.398	0.101	2.71E-38	12	Gadd45gip1
2.18E-42	0.462261992	0.693	0.233	2.87E-38	12	Ndufb2
2.21E-42	0.389008218	0.383	0.095	2.91E-38	12	Cct8
2.63E-42	0.480840025	0.992	0.697	3.47E-38	12	Rpl31
3.56E-42	0.589579941	0.916	0.431	4.70E-38	12	Uqcr11
4.05E-42	0.410598359	0.314	0.07	5.35E-38	12	Mrps28
4.23E-42	0.576454782	0.908	0.414	5.57E-38	12	Ybx1

4.83E-42	0.438063894	0.536	0.162	6.37E-38	12	Eef1d
5.36E-42	0.449351131	0.544	0.165	7.07E-38	12	Pdia3
6.54E-42	0.406803042	0.444	0.119	8.63E-38	12	Fkbp1a
7.51E-42	0.507426084	0.797	0.304	9.90E-38	12	Slc25a5
7.58E-42	0.477754278	0.655	0.219	1.00E-37	12	Park7
8.20E-42	0.522541901	0.985	0.633	1.08E-37	12	Rpl8
8.26E-42	0.472566521	0.996	0.797	1.09E-37	12	Rps24
1.17E-41	0.34923056	0.284	0.058	1.54E-37	12	Prmt1
1.80E-41	0.390313256	0.487	0.138	2.37E-37	12	Ostc
1.80E-41	0.481375858	0.609	0.198	2.37E-37	12	Pdap1
1.86E-41	0.328601355	0.352	0.084	2.46E-37	12	Ccnd2
2.26E-41	0.452737949	0.437	0.12	2.98E-37	12	Lsm7
2.30E-41	0.414413389	0.253	0.048	3.03E-37	12	Gria3
3.07E-41	0.378406664	0.529	0.156	4.05E-37	12	Ddx21
1.30E-40	0.408473673	0.295	0.064	1.72E-36	12	Eif3b
2.46E-40	0.566137193	0.828	0.346	3.25E-36	12	Ran
2.60E-40	0.514246562	0.927	0.418	3.43E-36	12	Gnb2l1
2.63E-40	0.466892907	1	0.775	3.47E-36	12	Rps5
3.19E-40	0.411142444	0.391	0.101	4.20E-36	12	Gng12
8.48E-40	0.47800263	1	0.783	1.12E-35	12	Ppia
1.18E-39	0.374621007	0.402	0.106	1.55E-35	12	Pfdn1
1.29E-39	0.50613553	0.391	0.105	1.70E-35	12	Ybx3
1.57E-39	0.402534992	0.521	0.158	2.07E-35	12	Naa38
1.66E-39	0.55769053	0.889	0.407	2.19E-35	12	Hint1
1.82E-39	0.405732141	0.46	0.132	2.41E-35	12	Rpl7a-ps5
1.89E-39	0.460671784	0.989	0.753	2.49E-35	12	Rpl17
2.64E-39	0.431907711	0.785	0.287	3.49E-35	12	Canx
5.58E-39	0.416686215	0.36	0.091	7.37E-35	12	Hells
5.96E-39	0.426637331	0.64	0.217	7.87E-35	12	Snrpd3
9.10E-39	0.458594856	0.372	0.097	1.20E-34	12	Cacybp
9.96E-39	0.445508781	0.563	0.182	1.31E-34	12	Llph

1.10E-38	0.531836247	0.893	0.408	1.45E-34	12	Serbp1
1.30E-38	0.35530462	0.295	0.066	1.71E-34	12	Nifk
1.30E-38	0.52255042	0.9	0.411	1.71E-34	12	Rpl5
1.99E-38	0.529889008	0.889	0.392	2.62E-34	12	2010107E04Rik
2.99E-38	0.496820859	0.943	0.447	3.94E-34	12	Rpl10-ps3
4.63E-38	0.53523471	0.862	0.378	6.11E-34	12	Tomm7
4.85E-38	0.524334664	0.843	0.358	6.39E-34	12	Eif5a
5.03E-38	0.357634524	0.437	0.123	6.63E-34	12	Tapbpl
5.60E-38	0.331360797	0.326	0.078	7.38E-34	12	Emc6
6.49E-38	0.391232929	0.456	0.133	8.56E-34	12	Morf4l2
1.18E-37	0.397257457	0.387	0.104	1.56E-33	12	Svip
1.32E-37	0.421574957	0.575	0.19	1.74E-33	12	Ndufa5
1.56E-37	0.53479415	0.923	0.459	2.06E-33	12	Rps27l
1.85E-37	-2.036701585	1	0.994	2.44E-33	12	S100a9
1.88E-37	0.309643311	0.318	0.075	2.48E-33	12	Rpn2
2.76E-37	0.373812874	0.487	0.148	3.64E-33	12	mt-Nd5
3.79E-37	0.44032433	0.739	0.274	5.00E-33	12	Nap1l1
4.67E-37	0.552064866	0.778	0.317	6.16E-33	12	Hnrnpa1
6.43E-37	0.497988082	0.973	0.617	8.49E-33	12	Uba52
6.79E-37	0.48133598	0.992	0.694	8.96E-33	12	Sub1
7.23E-37	0.344842564	0.548	0.173	9.54E-33	12	Thoc7
8.01E-37	0.29554518	0.253	0.052	1.06E-32	12	Bid
9.44E-37	0.458098069	0.992	0.714	1.25E-32	12	Rps4x
9.69E-37	0.388289227	0.609	0.204	1.28E-32	12	Swi5
1.13E-36	0.302578518	0.372	0.097	1.49E-32	12	Tifab
1.15E-36	0.453190199	0.996	0.751	1.52E-32	12	Rps3
1.54E-36	0.317169454	0.257	0.054	2.04E-32	12	Bcap29
1.62E-36	0.391434338	0.36	0.095	2.13E-32	12	Srpk1
1.82E-36	0.332292352	1	0.953	2.41E-32	12	mt-Co3
2.28E-36	0.495192488	0.985	0.595	3.01E-32	12	Gm11808
2.29E-36	0.373866336	0.437	0.128	3.02E-32	12	Eny2

2.36E-36	0.425279438	0.506	0.161	3.11E-32	12	Ndufab1
2.52E-36	-1.938915858	1	0.999	3.32E-32	12	S100a8
3.46E-36	0.394129436	0.67	0.24	4.56E-32	12	Lsm6
4.11E-36	0.495610236	0.989	0.623	5.43E-32	12	Sec61g
4.35E-36	0.350981882	0.387	0.106	5.74E-32	12	Eif3i
5.21E-36	0.397151998	0.391	0.108	6.87E-32	12	Nudc
5.44E-36	0.353924078	0.284	0.065	7.17E-32	12	Ddx18
6.25E-36	0.289279847	0.299	0.07	8.24E-32	12	Dnajc15
8.42E-36	0.440800197	0.732	0.28	1.11E-31	12	Ndufc1
9.85E-36	0.418492066	0.314	0.079	1.30E-31	12	Impdh2
1.08E-35	0.412662504	0.475	0.148	1.42E-31	12	Banf1
1.11E-35	0.467384873	0.977	0.584	1.47E-31	12	Rpl3
1.29E-35	0.494769441	0.897	0.415	1.70E-31	12	Gm9493
1.66E-35	0.360097551	0.559	0.184	2.19E-31	12	Gm10020
1.70E-35	0.433358507	0.996	0.678	2.24E-31	12	Hmgb1
1.74E-35	0.332088017	0.253	0.054	2.29E-31	12	Agpat5
1.84E-35	0.40215023	0.33	0.084	2.43E-31	12	Cdk4
1.89E-35	0.282225272	0.307	0.074	2.49E-31	12	Ahsa1
2.30E-35	0.351395095	0.674	0.239	3.04E-31	12	Ndufb8
2.50E-35	0.470488052	0.782	0.313	3.30E-31	12	Lsm5
2.98E-35	0.336416739	0.368	0.099	3.93E-31	12	Eif1ax
3.22E-35	0.363033032	0.556	0.183	4.24E-31	12	Caprin1
3.83E-35	0.362510377	0.521	0.169	5.06E-31	12	Gm6133
4.58E-35	0.429362506	1	0.8	6.04E-31	12	Rpl13a
4.81E-35	0.465494755	0.981	0.63	6.34E-31	12	Rpl18
5.55E-35	0.396310905	1	0.869	7.32E-31	12	Rpl18a
6.16E-35	0.4491898	0.816	0.331	8.12E-31	12	Rpl6l
6.65E-35	0.396966637	0.314	0.079	8.78E-31	12	Tcp1
1.28E-34	0.360888124	0.257	0.057	1.69E-30	12	Idh3g
1.88E-34	0.439396845	0.693	0.264	2.48E-30	12	Atp5b
2.40E-34	0.311197444	0.621	0.213	3.17E-30	12	Spcs2

2.84E-34	0.424038705	0.985	0.605	3.74E-30	12	mt-Nd1
4.60E-34	0.373688666	0.517	0.17	6.07E-30	12	Erdr1
4.85E-34	0.368206522	0.747	0.285	6.40E-30	12	mt-Nd2
5.36E-34	0.407998724	0.54	0.181	7.07E-30	12	Emb
7.29E-34	0.323271931	0.533	0.174	9.62E-30	12	Mrps33
7.50E-34	0.495513724	0.912	0.452	9.90E-30	12	Rpl29
1.02E-33	0.326702978	0.559	0.188	1.34E-29	12	Srsf7
1.31E-33	0.405574747	0.992	0.797	1.73E-29	12	Rpl34
2.65E-33	0.333922168	0.337	0.089	3.50E-29	12	Glipr1
4.09E-33	0.405064882	0.352	0.098	5.39E-29	12	Ssr2
5.16E-33	0.428795056	0.992	0.711	6.80E-29	12	Rpl19
6.80E-33	0.405345207	0.582	0.208	8.97E-29	12	Atp5a1
6.93E-33	0.277186173	0.257	0.058	9.14E-29	12	Hint2
7.35E-33	0.369044781	0.299	0.075	9.69E-29	12	Zfp422
8.64E-33	0.386611652	0.318	0.084	1.14E-28	12	Tmem245
9.85E-33	0.366126535	0.349	0.097	1.30E-28	12	Mrpl34
1.16E-32	0.349270267	0.284	0.07	1.53E-28	12	Pabpc4
1.31E-32	0.310075611	0.284	0.069	1.72E-28	12	Slc25a4
1.86E-32	0.430021097	0.996	0.699	2.45E-28	12	Rplp0
2.57E-32	0.407089315	0.989	0.735	3.39E-28	12	Rpl9
3.14E-32	0.455739211	0.958	0.575	4.15E-28	12	Rpl4
6.87E-32	0.485784212	0.912	0.482	9.07E-28	12	Rpl7a
6.97E-32	0.364925862	0.429	0.135	9.19E-28	12	Chchd1
1.01E-31	0.344963998	0.398	0.12	1.33E-27	12	Polr2f
1.20E-31	0.439272629	0.966	0.557	1.59E-27	12	Rpl36al
1.49E-31	0.335688918	0.391	0.116	1.97E-27	12	Ndufv2
1.66E-31	0.327262303	0.268	0.065	2.19E-27	12	Dnajc2
1.67E-31	0.373676044	0.778	0.322	2.21E-27	12	Hsp90b1
1.69E-31	0.430186381	0.977	0.692	2.24E-27	12	mt-Cytb
2.43E-31	0.293949411	0.452	0.143	3.20E-27	12	Magt1
2.46E-31	0.347936306	0.375	0.111	3.24E-27	12	Lsm2

3.00E-31	0.351950856	0.575	0.204	3.96E-27	12	Ssr1
3.37E-31	0.321818952	0.31	0.083	4.44E-27	12	Cct2
3.84E-31	0.401917103	0.996	0.759	5.07E-27	12	Rpl21
5.29E-31	0.32285084	0.414	0.128	6.97E-27	12	Psma5
9.39E-31	0.320331574	0.475	0.156	1.24E-26	12	Tomm20
9.45E-31	0.333216196	0.582	0.208	1.25E-26	12	Snrpd1
9.94E-31	0.397099682	0.659	0.256	1.31E-26	12	Atp5o
1.35E-30	0.256987039	0.395	0.118	1.78E-26	12	Rpl13a-ps1
1.57E-30	0.363916658	0.467	0.155	2.07E-26	12	Higd1a
1.69E-30	0.414759128	0.996	0.67	2.23E-26	12	Rpsa
1.70E-30	0.33901785	0.372	0.111	2.24E-26	12	Cdc26
2.58E-30	0.422249308	0.889	0.416	3.40E-26	12	Rpl13-ps3
4.62E-30	0.434591839	0.851	0.387	6.09E-26	12	Uqcrq
5.62E-30	0.378430267	0.996	0.786	7.42E-26	12	Rps11
5.63E-30	0.325038465	0.337	0.096	7.43E-26	12	Ddost
6.23E-30	0.354588005	0.261	0.065	8.22E-26	12	Paics
6.42E-30	0.466383995	0.958	0.588	8.47E-26	12	Cox7c
6.44E-30	0.404853369	0.59	0.224	8.50E-26	12	Cks1b
6.51E-30	0.378401967	0.368	0.111	8.59E-26	12	Wbp5
6.57E-30	0.340891715	0.517	0.179	8.67E-26	12	Dut
6.60E-30	0.340189614	0.521	0.183	8.71E-26	12	2410015M20Rik
7.27E-30	0.385188844	0.713	0.286	9.59E-26	12	Uqcrb
1.04E-29	0.333389838	0.36	0.107	1.37E-25	12	Nars
1.20E-29	0.282372688	0.402	0.125	1.58E-25	12	Smim11
1.65E-29	0.298837177	0.257	0.063	2.17E-25	12	Exosc5
2.19E-29	0.405823708	0.996	0.645	2.90E-25	12	Eef1a1
2.94E-29	0.449711835	0.866	0.43	3.88E-25	12	Rpl9-ps6
3.07E-29	0.278655675	0.395	0.123	4.05E-25	12	Zfos1
3.77E-29	0.273357954	0.318	0.088	4.97E-25	12	Mdh1
4.85E-29	0.381917329	0.628	0.244	6.39E-25	12	Atp5g3
6.33E-29	0.372717373	0.732	0.299	8.35E-25	12	Snrpe

6.77E-29	0.29583059	0.391	0.122	8.93E-25	12	Nudt21
9.27E-29	0.261057907	0.257	0.064	1.22E-24	12	Dph3
1.29E-28	0.255137978	0.268	0.069	1.70E-24	12	Ndufs3
1.45E-28	0.342957836	0.307	0.087	1.91E-24	12	Mrpl12
2.02E-28	0.337922699	0.456	0.155	2.67E-24	12	Ssr3
2.33E-28	0.269471625	0.33	0.095	3.08E-24	12	Mgat2
2.49E-28	0.457042915	0.923	0.517	3.29E-24	12	Atp5k
2.86E-28	0.314738866	0.364	0.112	3.77E-24	12	Eif2s3x
3.42E-28	0.310924079	0.552	0.202	4.51E-24	12	Erp29
3.74E-28	0.253844137	0.295	0.081	4.93E-24	12	Mydgf
4.12E-28	0.331577832	0.559	0.208	5.43E-24	12	Ndufc2
4.28E-28	0.305038246	0.341	0.102	5.65E-24	12	Phb2
4.42E-28	0.306747225	0.69	0.265	5.83E-24	12	Tra2b
4.91E-28	0.314423509	0.314	0.09	6.48E-24	12	Sms
6.46E-28	0.381712461	0.743	0.318	8.52E-24	12	Cox5a
6.65E-28	0.369745431	0.839	0.378	8.77E-24	12	mt-Atp8
6.69E-28	0.302261366	0.337	0.1	8.82E-24	12	Lman2
6.80E-28	0.344451668	0.314	0.091	8.97E-24	12	Gspt1
7.04E-28	0.256759143	0.307	0.086	9.28E-24	12	Dnajc3
1.04E-27	0.333973036	0.663	0.262	1.37E-23	12	mt-Nd4l
1.12E-27	0.422882197	0.9	0.451	1.48E-23	12	Gm8730
1.30E-27	0.324776609	1	0.876	1.71E-23	12	Rps14
1.39E-27	0.325573771	0.517	0.188	1.84E-23	12	Ndufb5
1.55E-27	0.321303112	0.49	0.173	2.05E-23	12	Gm11273
1.58E-27	0.331631958	0.521	0.19	2.08E-23	12	Supt16
1.66E-27	0.305454432	0.333	0.099	2.19E-23	12	Nup210
2.15E-27	0.350838499	0.387	0.125	2.84E-23	12	Naa50
2.32E-27	0.284462336	0.322	0.094	3.06E-23	12	Mcm5
3.17E-27	0.319463796	0.303	0.087	4.19E-23	12	Hmgn5
3.52E-27	0.352149182	0.318	0.094	4.65E-23	12	Rgcc
3.70E-27	0.314765615	0.372	0.118	4.88E-23	12	Magoh

7.99E-27	0.280863937	0.529	0.192	1.05E-22	12	Ewsr1
8.36E-27	0.383117984	0.632	0.255	1.10E-22	12	Romo1
8.36E-27	0.3138039	0.372	0.119	1.10E-22	12	Mrpl54
9.33E-27	0.264701501	0.307	0.089	1.23E-22	12	Kcnq1ot1
1.12E-26	0.288810147	0.678	0.264	1.47E-22	12	Hnrnpab
1.50E-26	-0.639770481	1	0.997	1.98E-22	12	Malat1
1.90E-26	0.265859948	0.41	0.136	2.50E-22	12	Eif3c
2.36E-26	0.293452199	0.318	0.095	3.11E-22	12	Huwe1
2.54E-26	0.314014447	0.586	0.225	3.35E-22	12	Eif5b
3.02E-26	0.320816903	0.605	0.238	3.99E-22	12	Timm13
3.52E-26	0.264638299	0.299	0.087	4.65E-22	12	Cfdp1
3.58E-26	0.266951492	0.383	0.124	4.72E-22	12	Med21
4.08E-26	0.400742876	0.946	0.53	5.38E-22	12	Rpl7
4.10E-26	0.428418362	0.874	0.455	5.41E-22	12	Ndufa4
4.87E-26	0.306786704	0.295	0.086	6.42E-22	12	Rcc2
4.93E-26	0.371566929	0.87	0.431	6.51E-22	12	Psma7
5.03E-26	0.330728411	0.418	0.144	6.64E-22	12	mt-Nd3
5.18E-26	0.281573038	0.567	0.216	6.83E-22	12	Anp32e
6.50E-26	-1.980443646	0.992	0.928	8.58E-22	12	Camp
7.04E-26	0.290786601	0.464	0.165	9.28E-22	12	Lsm3
7.25E-26	0.319394902	0.456	0.161	9.56E-22	12	U2af1
8.51E-26	0.292509788	0.284	0.081	1.12E-21	12	Mrpl21
1.35E-25	0.279503005	0.513	0.19	1.78E-21	12	Ssb
1.51E-25	0.401822838	0.831	0.394	2.00E-21	12	Chchd2
1.68E-25	0.250506347	0.337	0.105	2.21E-21	12	Naa15
1.86E-25	0.369956885	0.839	0.39	2.45E-21	12	Anp32b
2.44E-25	0.275006988	0.303	0.089	3.22E-21	12	Mcm6
3.83E-25	0.253479825	0.28	0.08	5.05E-21	12	Golga3
4.13E-25	0.362412866	0.31	0.096	5.45E-21	12	Mcm7
4.84E-25	0.300357115	0.582	0.229	6.38E-21	12	Atp5f1
4.86E-25	0.270752877	0.467	0.17	6.41E-21	12	Trmt112

5.17E-25	0.270886144	0.391	0.131	6.82E-21	12	Ndufs8
6.82E-25	0.306206256	0.464	0.168	8.99E-21	12	Dnmt1
1.17E-24	0.278425048	0.345	0.111	1.55E-20	12	Tomm6
1.36E-24	0.264744488	0.632	0.254	1.80E-20	12	Tmed2
1.45E-24	0.302526529	0.598	0.239	1.91E-20	12	Eif3a
1.53E-24	0.313531709	0.414	0.146	2.02E-20	12	Dpy30
1.60E-24	0.281758682	0.349	0.113	2.11E-20	12	Pfdn6
2.62E-24	0.275301439	0.33	0.104	3.46E-20	12	Ptpn7
2.82E-24	0.277426269	0.636	0.264	3.72E-20	12	Slpi
2.89E-24	0.285978378	0.345	0.112	3.82E-20	12	Cenpw
3.17E-24	0.291437836	0.356	0.118	4.18E-20	12	Zc3h15
3.26E-24	0.255729358	0.345	0.111	4.30E-20	12	Tpi1
3.49E-24	0.420935369	0.904	0.53	4.60E-20	12	Hspa8
3.72E-24	0.323142872	0.276	0.081	4.91E-20	12	2810004N23Rik
5.88E-24	0.271709107	0.295	0.089	7.75E-20	12	Eif3g
6.71E-24	0.37343472	0.866	0.434	8.85E-20	12	Naca
7.93E-24	0.293817865	0.253	0.072	1.05E-19	12	Knop1
8.59E-24	0.283035203	0.678	0.284	1.13E-19	12	Ost4
9.91E-24	0.316491475	0.307	0.096	1.31E-19	12	Cct5
1.30E-23	0.285893397	0.253	0.072	1.72E-19	12	Grpel1
1.36E-23	0.316949394	0.793	0.355	1.80E-19	12	Srsf2
1.43E-23	0.258343131	0.261	0.075	1.89E-19	12	1810022K09Rik
1.48E-23	0.259572325	0.475	0.177	1.96E-19	12	Psmb2
1.64E-23	0.262912056	0.686	0.285	2.16E-19	12	Sfpq
1.73E-23	0.28710535	0.598	0.242	2.28E-19	12	Alyref
2.13E-23	0.295356047	0.456	0.171	2.81E-19	12	Pcna
2.60E-23	0.313189663	0.291	0.09	3.43E-19	12	Rbbp7
3.11E-23	0.288012053	0.291	0.089	4.10E-19	12	Aimp1
3.36E-23	0.264300974	0.406	0.144	4.43E-19	12	Snrpb2
3.36E-23	0.339512829	0.801	0.365	4.44E-19	12	Minos1
3.57E-23	0.347382629	0.828	0.396	4.70E-19	12	Gapdh

4.22E-23	0.25166662	0.402	0.142	5.57E-19	12	Ndufb6
4.29E-23	0.369826071	0.885	0.436	5.66E-19	12	Uqcr10
5.07E-23	0.343706001	0.751	0.351	6.69E-19	12	Tuba1b
5.12E-23	0.286471183	0.716	0.31	6.75E-19	12	Ndufa7
6.06E-23	-0.921977325	0.77	0.728	8.00E-19	12	Cd52
9.17E-23	0.275577168	0.291	0.091	1.21E-18	12	Phf5a
1.22E-22	0.30647342	0.67	0.289	1.61E-18	12	Hnrnpm
1.29E-22	0.352851233	0.774	0.369	1.71E-18	12	Tmem258
1.45E-22	0.252514005	0.406	0.146	1.91E-18	12	Hnrnpa0
1.82E-22	0.253354859	0.613	0.25	2.40E-18	12	Ndufs6
2.21E-22	-0.631002017	1	0.999	2.91E-18	12	Actb
2.45E-22	0.369272866	0.935	0.535	3.24E-18	12	Cox6b1
3.46E-22	0.267682398	0.383	0.137	4.57E-18	12	Mrpl51
4.01E-22	0.293719839	0.299	0.096	5.29E-18	12	Hyou1
4.36E-22	0.315957217	0.69	0.305	5.76E-18	12	Hnrnpu
4.87E-22	0.367906823	0.962	0.61	6.43E-18	12	Cox6c
8.77E-22	0.269371907	0.307	0.1	1.16E-17	12	Fundc2
1.02E-21	0.277412252	0.41	0.153	1.34E-17	12	Lig1
1.38E-21	0.363352582	0.858	0.452	1.82E-17	12	Cox5b
1.41E-21	0.323810801	0.755	0.358	1.86E-17	12	Tmem256
1.80E-21	0.265582156	0.782	0.359	2.38E-17	12	Btf3
2.17E-21	0.27313772	0.318	0.106	2.87E-17	12	Anxa3
2.42E-21	0.312669187	0.962	0.554	3.19E-17	12	mt-Nd4
2.78E-21	0.275814058	0.414	0.155	3.67E-17	12	Stra13
4.69E-21	0.253037779	0.326	0.111	6.19E-17	12	Mlec
5.04E-21	-2.01755944	0.966	0.845	6.65E-17	12	Ngp
5.41E-21	0.265210327	0.253	0.077	7.14E-17	12	Psmd7
5.96E-21	0.280469283	0.77	0.353	7.86E-17	12	Myeov2
7.48E-21	0.304333317	0.835	0.409	9.87E-17	12	Eef2
8.57E-21	0.260278616	1	0.853	1.13E-16	12	mt-Atp6
9.77E-21	0.278396346	0.287	0.094	1.29E-16	12	Denr

1.10E-20	0.256247787	0.264	0.083	1.45E-16	12	Mrps36
2.78E-20	0.278409646	0.264	0.084	3.67E-16	12	Wdr61
4.55E-20	-1.268640147	0.341	0.508	6.00E-16	12	Cybb
5.10E-20	0.259801658	0.801	0.386	6.72E-16	12	Dbi
6.58E-20	-0.843351828	0.705	0.683	8.69E-16	12	Cyba
9.68E-20	0.27901238	0.257	0.082	1.28E-15	12	Plk4
1.36E-19	0.298598329	0.693	0.323	1.79E-15	12	Srsf3
2.37E-19	0.356825588	0.824	0.436	3.13E-15	12	Usmg5
3.96E-19	-1.477884738	0.406	0.537	5.22E-15	12	S100a6
4.90E-19	-1.592235393	0.464	0.556	6.46E-15	12	Chil3
3.45E-18	0.25506582	0.908	0.491	4.55E-14	12	Cbx3
5.92E-18	0.326825983	0.927	0.558	7.80E-14	12	Cox8a
7.19E-18	0.310410583	0.858	0.482	9.49E-14	12	Tubb5
6.97E-17	0.275183706	0.831	0.435	9.19E-13	12	Hnrnpa3
2.47E-16	0.296211873	0.877	0.505	3.26E-12	12	Atp5j2
2.53E-16	-1.848158243	0.471	0.544	3.34E-12	12	Ltf
3.01E-16	-1.838717106	0.674	0.628	3.97E-12	12	Wfdc21
4.12E-16	0.302960705	0.923	0.601	5.44E-12	12	Ly6c2
9.39E-16	-0.659265096	0.923	0.811	1.24E-11	12	Tyrobp
1.10E-15	-1.547483961	0.172	0.368	1.46E-11	12	Ifitm6
3.82E-15	-1.90475191	0.536	0.559	5.04E-11	12	Lcn2
5.37E-15	0.278516235	0.912	0.553	7.08E-11	12	Hnrnpa2b1
8.14E-15	0.275471824	0.877	0.496	1.07E-10	12	Pabpc1
9.30E-15	0.275059277	0.939	0.588	1.23E-10	12	Atp5e
1.72E-14	0.259982461	0.958	0.636	2.27E-10	12	Atp5l
1.94E-14	-0.700602501	0.778	0.711	2.56E-10	12	Fcer1g
9.45E-14	-1.517397692	0.337	0.452	1.25E-09	12	Pglyrp1
3.37E-13	0.253536286	0.812	0.461	4.44E-09	12	Cox7a2
2.73E-12	-0.740643231	0.716	0.642	3.60E-08	12	Prdx5
9.25E-11	-1.103546225	0.176	0.322	1.22E-06	12	Lsp1
5.69E-10	-0.348049994	0.931	0.831	7.50E-06	12	Pfn1

2.93E-09	-1.051660041	0.375	0.441	3.87E-05	12	Lgals3	
7.81E-09	-1.104367481	0.375	0.432	0.000103013	12	Anxa1	
7.91E-09	-1.223155255	0.625	0.556	0.000104403	12	Ifitm3	
1.37E-07	-1.466946614	0.287	0.362	0.001801723	12	Ifi27l2a	
4.12E-07	-1.146479823	0.778	0.631	0.005431775	12	Crip1	
8.72E-06	-0.31702692	0.95	0.767	0.115018044	12	H3f3a	
4.86E-05	-0.842245154	0.651	0.539	0.641372779	12	Psap	
6.89E-05	-0.428702514	0.816	0.642	0.908481165	12	Itm2b	
7.81E-05	-0.341974389	0.885	0.701	1	12	Myl6	
0.00010778	-0.41244844	0.847	0.657	1	12	Arhgdib	
0.00019588	-0.89083066	0.192	0.252	1	12	Tmcc1	
0.00021606	-0.740664181	0.207	0.268	1	12	Clec4a2	
0.00032307	-0.681867734	0.498	0.453	1	12	Hp	
0.00093038	-0.669888899	0.31	0.182	1	12	Bst2	
0.00279064	-0.748052113	0.307	0.316	1	12	Ptprc	
0.00365551	-0.263192132	0.448	0.272	1	12	Lcp1	
0.00491455	-0.34139923	0.935	0.764	1	12	B2m	
0.00507574	-0.362867189	0.41	0.251	1	12	Gm9844	
0.00710066	-0.727040209	0.391	0.363	1	12	Lst1	
0.00739616	-0.645582701	0.375	0.369	1	12	Myh9	
0.00750575	-0.272591073	0.429	0.266	1	12	Gabarap	
0.00752387	-0.292227356	0.854	0.679	1	12	Actg1	
0	2.267786924	0.92	0.047	0	13	Car2	**Car enzyme HPC**
0	2.014054229	0.764	0.025	0	13	Car1	
0	1.607811611	0.598	0.006	0	13	Mt2	
0	1.496237731	0.704	0.041	0	13	Fam132a	
0	1.452960043	0.573	0.023	0	13	Nfia	
0	1.371486656	0.668	0.024	0	13	Aqp1	
0	1.248682834	0.553	0.011	0	13	Ermap	
0	1.23085922	0.523	0.017	0	13	Smim1	
0	1.181794729	0.533	0.013	0	13	Abcb4	

0	1.157353285	0.503	0.011	0	13	Klf1	
0	0.784008122	0.327	0.004	0	13	Ces2g	
0	0.773726524	0.347	0.003	0	13	Atp1b2	
1.44E-287	0.661837613	0.281	0.003	1.90E-283	13	Sphk1	
1.40E-274	0.976126885	0.442	0.015	1.85E-270	13	Stard10	
7.22E-265	1.24923057	0.583	0.033	9.52E-261	13	Minpp1	
4.60E-253	1.145293937	0.558	0.031	6.06E-249	13	Cpox	
4.61E-238	0.774100436	0.357	0.011	6.08E-234	13	Gata1	
1.10E-236	0.741048274	0.302	0.007	1.45E-232	13	Nxpe2	
7.83E-226	0.86724766	0.377	0.014	1.03E-221	13	Pla2g12a	
1.76E-223	0.692719483	0.332	0.01	2.32E-219	13		8-Sep
6.79E-213	0.778998979	0.286	0.007	8.95E-209	13	Rhag	
1.79E-211	0.997418807	0.497	0.03	2.36E-207	13	Mns1	
1.24E-202	1.198585987	0.618	0.051	1.64E-198	13	Blvrb	
1.13E-199	0.94799177	0.377	0.017	1.49E-195	13	Hemgn	
7.84E-190	0.746148068	0.317	0.012	1.03E-185	13	Gstm5	
2.27E-184	0.629840711	0.276	0.008	2.99E-180	13	Cyth3	
3.21E-180	0.709144622	0.281	0.009	4.23E-176	13	Hebp1	
1.23E-176	0.885708836	0.332	0.015	1.62E-172	13	Ctse	
2.64E-174	1.387304941	0.789	0.106	3.49E-170	13	Glrx5	
2.75E-172	0.647591966	0.251	0.007	3.63E-168	13	Cldn13	
3.82E-169	1.523591465	0.739	0.099	5.03E-165	13	Mt1	
9.92E-164	1.003891906	0.533	0.047	1.31E-159	13	Odc1	
2.17E-162	0.942524586	0.553	0.051	2.87E-158	13	Cox6b2	
8.45E-159	1.090063705	0.598	0.063	1.12E-154	13	Hmgb3	
8.77E-155	0.794257956	0.432	0.032	1.16E-150	13	Chchd10	
5.04E-153	1.073906568	0.578	0.06	6.65E-149	13	Tfrc	
1.54E-149	0.683197161	0.332	0.018	2.04E-145	13	Rangrf	
1.50E-148	1.119455002	0.573	0.063	1.97E-144	13	Pabpc4	
3.69E-148	0.734587923	0.342	0.02	4.87E-144	13	Ubac1	
8.68E-148	0.724798358	0.307	0.016	1.15E-143	13	Zfpm1	

5.90E-146	0.806386704	0.342	0.02	7.78E-142	13	Ifrd2
9.28E-146	0.90934805	0.528	0.054	1.22E-141	13	Hba-a2
2.13E-136	0.602962204	0.296	0.016	2.80E-132	13	Mthfd1
3.36E-134	0.700098618	0.362	0.026	4.43E-130	13	Tmem238
1.36E-133	0.988408633	0.633	0.082	1.79E-129	13	Bola3
5.41E-123	0.715632123	0.367	0.029	7.13E-119	13	Hmbs
4.10E-121	0.59171546	0.312	0.021	5.41E-117	13	Kcnn4
2.60E-120	1.152292061	0.553	0.076	3.43E-116	13	Hba-a1
3.37E-120	0.985122209	0.668	0.1	4.45E-116	13	Hmgn1
3.30E-114	1.437494092	0.879	0.235	4.35E-110	13	Ncl
3.91E-113	0.916186288	0.533	0.069	5.16E-109	13	C1qbp
8.49E-113	0.833888949	0.508	0.062	1.12E-108	13	Gclm
9.70E-112	1.08370971	0.729	0.128	1.28E-107	13	Prdx2
2.51E-111	0.807493916	0.457	0.051	3.31E-107	13	Wdr43
5.16E-111	1.176220053	0.749	0.147	6.80E-107	13	Hdgf
9.87E-111	0.865045242	0.553	0.074	1.30E-106	13	2810004N23Rik
1.26E-110	0.613517854	0.302	0.022	1.66E-106	13	Alad
2.60E-110	0.658395853	0.357	0.031	3.42E-106	13	Ahctf1
4.39E-110	-1.79981761	0.97	0.997	5.79E-106	13	Tmsb4x
8.95E-110	0.987925014	0.638	0.1	1.18E-105	13	Ybx3
1.83E-109	1.052181746	0.543	0.074	2.42E-105	13	Golga3
5.96E-109	1.11413149	0.819	0.172	7.86E-105	13	Dut
2.08E-107	0.634496528	0.312	0.024	2.74E-103	13	Gm10110
1.14E-106	0.678166199	0.367	0.034	1.50E-102	13	Ammecr1
1.69E-106	0.84364426	0.447	0.052	2.23E-102	13	Gnl3
1.18E-105	0.680406535	0.492	0.061	1.55E-101	13	Ppp1r14b
2.66E-104	0.871028876	0.462	0.056	3.51E-100	13	Pola2
3.17E-104	1.007032878	0.598	0.096	4.18E-100	13	Hspd1
1.96E-103	0.613004601	0.302	0.023	2.59E-99	13	Ubxn2a
1.02E-101	1.143438996	0.839	0.195	1.35E-97	13	Pdap1
1.18E-100	0.800520781	0.548	0.079	1.56E-96	13	Nop58

1.66E-99	1.019145969	0.789	0.165	2.18E-95	13	Mrpl52
2.67E-99	0.767227068	0.412	0.047	3.52E-95	13	Noa1
4.13E-97	1.119010208	0.859	0.216	5.45E-93	13	Metap2
1.14E-96	0.79819273	0.452	0.057	1.51E-92	13	Pfas
1.32E-96	0.63731643	0.442	0.054	1.74E-92	13	Eef1e1
2.03E-96	0.894962427	0.558	0.087	2.68E-92	13	Nop56
3.25E-96	1.070588359	0.734	0.153	4.29E-92	13	Ddx21
4.49E-95	0.908237625	0.302	0.026	5.92E-91	13	Fn3krp
9.63E-95	1.262190928	0.975	0.541	1.27E-90	13	Rps2
7.20E-93	0.699169348	0.382	0.043	9.50E-89	13	Nolc1
9.00E-93	0.65480802	0.377	0.042	1.19E-88	13	Gar1
2.97E-91	0.640279023	0.266	0.021	3.92E-87	13	Slc16a1
2.73E-90	0.787718237	0.553	0.089	3.60E-86	13	Hspa9
5.08E-90	0.552319019	0.302	0.027	6.70E-86	13	Phf10
8.12E-90	0.709665857	0.437	0.057	1.07E-85	13	Tcf3
1.81E-89	0.841924379	0.603	0.105	2.39E-85	13	Vamp5
1.13E-88	0.594920859	0.352	0.038	1.49E-84	13	Mospd3
2.84E-88	0.759651449	0.528	0.083	3.75E-84	13	Pa2g4
2.15E-87	0.7658948	0.523	0.082	2.83E-83	13	Mrpl12
4.27E-86	0.79633224	1	0.954	5.63E-82	13	Rpl41
5.26E-86	0.791466746	0.603	0.108	6.93E-82	13	Pebp1
2.85E-84	0.677882531	0.462	0.067	3.76E-80	13	Knop1
2.98E-84	0.619877798	0.266	0.023	3.92E-80	13	Orc2
4.03E-84	0.597380169	0.397	0.05	5.31E-80	13	Ugcg
1.78E-83	0.53646108	0.352	0.04	2.35E-79	13	Pdcd11
1.11E-82	0.699646663	0.513	0.082	1.46E-78	13	Cfdp1
1.23E-82	0.720711654	0.563	0.097	1.62E-78	13	Phb2
4.64E-82	1.047498031	0.884	0.265	6.12E-78	13	Eef1g
1.97E-81	0.824032491	0.658	0.133	2.59E-77	13	Nhp2
1.06E-80	1.059981396	0.794	0.211	1.40E-76	13	Hspe1
3.69E-80	0.651076298	0.528	0.087	4.86E-76	13	Tnfaip2

3.29E-79	1.101080372	0.869	0.297	4.34E-75	13	Atp5g1
9.68E-79	0.463012464	0.276	0.026	1.28E-74	13	Hsph1
3.31E-78	0.710313046	0.427	0.062	4.37E-74	13	Pole3
1.21E-77	0.9525319	0.98	0.715	1.60E-73	13	Rplp1
1.88E-77	0.547096954	0.291	0.03	2.48E-73	13	E2f4
7.08E-76	0.688110446	0.442	0.067	9.34E-72	13	Svbp
1.45E-75	0.896256935	0.724	0.169	1.92E-71	13	Tmem14c
2.48E-75	1.097734517	0.905	0.324	3.27E-71	13	Set
2.88E-75	0.560323565	0.322	0.037	3.80E-71	13	Dkc1
7.76E-75	0.658402862	0.457	0.072	1.02E-70	13	Lyar
1.21E-74	0.535704407	0.296	0.032	1.60E-70	13	Ruvbl1
1.89E-74	0.961045687	1	0.648	2.49E-70	13	Rps12
2.94E-74	0.597704429	0.307	0.035	3.88E-70	13	Nsun2
3.00E-74	0.940408029	0.99	0.676	3.96E-70	13	Rpl14
3.06E-74	0.484825541	0.276	0.028	4.03E-70	13	Ddx1
1.63E-73	1.110910316	0.935	0.435	2.15E-69	13	Atpif1
5.52E-73	0.430412679	0.266	0.026	7.28E-69	13	Gpatch4
1.13E-72	0.60690252	0.387	0.055	1.50E-68	13	Srm
9.61E-72	0.916252394	0.985	0.702	1.27E-67	13	Rplp0
1.53E-71	0.959374421	0.99	0.468	2.02E-67	13	Rpl12
3.32E-71	0.692910168	0.457	0.075	4.38E-67	13	Aplp2
3.50E-71	0.936012582	0.523	0.101	4.62E-67	13	Khsrp
5.00E-71	0.820964068	0.598	0.125	6.60E-67	13	Snhg9
6.43E-71	0.532763537	0.302	0.035	8.48E-67	13	Scoc
1.02E-70	0.622303222	0.417	0.064	1.35E-66	13	Nifk
1.37E-70	0.663875997	0.508	0.091	1.80E-66	13	Ppm1g
3.00E-70	0.560354029	0.633	0.13	3.95E-66	13	Bcl11a
5.41E-70	0.780699581	0.467	0.081	7.13E-66	13	Tmem245
8.13E-70	0.822775996	0.623	0.137	1.07E-65	13	Comt
3.38E-69	0.668109962	0.487	0.086	4.46E-65	13	Phf5a
4.50E-69	0.647736507	0.588	0.119	5.93E-65	13	Zfos1

5.57E-68	0.464607951	0.256	0.026	7.34E-64	13	Nudt19
5.63E-68	0.656831106	0.563	0.112	7.43E-64	13	Cbx5
9.16E-68	0.649911781	0.457	0.079	1.21E-63	13	Mrps36
3.06E-67	0.650353328	0.372	0.054	4.04E-63	13	Ssbp3
5.24E-67	0.965475866	0.935	0.424	6.91E-63	13	Gm10073
7.08E-67	0.481178764	0.347	0.048	9.33E-63	13	Timm44
1.09E-66	0.644098824	0.548	0.109	1.44E-62	13	Abcf1
1.12E-66	0.626718673	0.417	0.068	1.48E-62	13	Sfxn1
1.78E-66	0.986540448	0.935	0.442	2.35E-62	13	Rps12-ps3
1.89E-66	0.539786825	0.332	0.044	2.50E-62	13	Ap1b1
5.35E-66	0.81807426	0.98	0.746	7.05E-62	13	Gm10076
1.38E-65	0.558323857	0.447	0.077	1.82E-61	13	Impdh2
1.65E-65	0.663191479	0.492	0.092	2.18E-61	13	Mcm7
3.96E-65	0.882051989	0.261	0.029	5.23E-61	13	Loxl2
4.10E-65	0.581954954	0.392	0.061	5.41E-61	13	Ngfrap1
4.15E-65	0.55575618	0.352	0.05	5.47E-61	13	Tlk1
6.14E-65	0.990864178	0.925	0.412	8.10E-61	13	Serbp1
1.03E-64	0.905164762	0.955	0.455	1.36E-60	13	Rpl29
1.04E-64	0.582109873	0.327	0.044	1.38E-60	13	Myc
6.00E-64	0.748797815	1	0.831	7.91E-60	13	Rps8
7.39E-64	0.722939553	0.643	0.151	9.75E-60	13	Tomm5
8.94E-64	0.74559949	0.693	0.172	1.18E-59	13	Eif3j1
5.57E-63	0.720421925	0.995	0.831	7.35E-59	13	Rps18
3.89E-62	0.528447169	0.307	0.041	5.13E-58	13	Fen1
1.26E-61	0.718088556	0.266	0.032	1.67E-57	13	Gm13075
1.81E-61	0.782203344	0.633	0.154	2.39E-57	13	Tomm20
2.04E-61	0.505832908	0.437	0.077	2.69E-57	13	Ddx46
3.72E-61	0.820442325	0.749	0.21	4.91E-57	13	Nme1
6.85E-61	0.474967022	0.317	0.044	9.03E-57	13	Alkbh1
9.07E-61	0.451406729	0.281	0.035	1.20E-56	13	Ndufaf4
1.06E-60	0.466634537	0.291	0.038	1.39E-56	13	Tomm40

1.41E-60	0.904978512	0.925	0.455	1.86E-56	13	Gm8730
3.92E-60	0.859068424	0.749	0.218	5.17E-56	13	Usp50
6.24E-60	0.484219325	0.302	0.04	8.23E-56	13	Ube2m
9.83E-60	0.715377378	0.337	0.052	1.30E-55	13	Hbb-bt
2.52E-59	0.607104346	0.422	0.076	3.33E-55	13	Nedd4
6.53E-59	0.986985496	0.869	0.367	8.62E-55	13	Npm1
1.02E-58	0.686523492	0.99	0.854	1.35E-54	13	Rpl35
1.26E-58	0.766346764	0.99	0.758	1.66E-54	13	Rpl36
1.65E-58	0.683887406	0.563	0.127	2.18E-54	13	Dtymk
2.74E-58	0.52139999	0.508	0.104	3.61E-54	13	Rexo2
4.50E-58	0.769708419	0.397	0.07	5.94E-54	13	Exosc2
6.42E-58	0.53757581	0.362	0.059	8.47E-54	13	Fam162a
1.00E-57	0.475886582	0.327	0.049	1.32E-53	13	Rsl1d1
1.09E-57	0.525972572	0.271	0.035	1.43E-53	13	Wdr12
1.11E-57	0.567192052	0.412	0.074	1.47E-53	13	Psmd7
1.74E-57	0.521501251	0.276	0.036	2.30E-53	13	Gclc
4.29E-57	0.70205649	0.628	0.155	5.66E-53	13	Prpf4b
7.46E-57	0.402651886	0.437	0.088	9.84E-53	13	Hbb-bs
8.94E-57	-1.929801013	0.894	0.906	1.18E-52	13	Lyz2
1.02E-56	0.859022367	0.874	0.318	1.34E-52	13	Hnrnpa1
3.66E-56	0.477831763	0.367	0.061	4.83E-52	13	Acp1
3.78E-56	0.473041158	0.286	0.039	4.99E-52	13	Gm6576
6.78E-56	0.42942333	0.271	0.035	8.94E-52	13	Ddx27
1.08E-55	0.543629806	0.412	0.076	1.42E-51	13	Fkbp4
1.68E-55	0.471856709	0.286	0.04	2.22E-51	13	Josd2
2.09E-55	0.688313021	0.995	0.805	2.75E-51	13	Rps23
2.27E-55	0.665736571	0.995	0.803	2.99E-51	13	Rpl13a
2.28E-55	0.414408777	0.412	0.074	3.00E-51	13	Tspan13
5.08E-55	0.695956229	0.628	0.158	6.70E-51	13	Cenpa
5.79E-55	0.589575993	0.472	0.097	7.63E-51	13	Psip1
7.96E-55	0.640858984	0.397	0.073	1.05E-50	13	Rrm1

9.69E-55	0.646226653	0.995	0.847	1.28E-50	13	Rpl13
1.18E-54	0.495310912	0.452	0.089	1.56E-50	13	Gspt1
1.42E-54	0.442745445	0.312	0.047	1.88E-50	13	Nenf
1.76E-54	0.676219379	0.503	0.11	2.32E-50	13	Eif2s3x
2.01E-54	0.664440653	0.638	0.165	2.65E-50	13	Hypk
2.68E-54	0.381003422	0.307	0.045	3.53E-50	13	Acsl5
3.03E-54	0.776456507	0.965	0.632	3.99E-50	13	Fth1
4.05E-54	0.483165363	0.276	0.038	5.34E-50	13	Eprs
4.42E-54	0.602780481	0.995	0.899	5.83E-50	13	Rpl37
1.42E-53	0.722092896	0.754	0.224	1.87E-49	13	Eif5b
1.51E-53	0.749412068	0.97	0.637	1.99E-49	13	Rpl8
1.66E-53	0.895863203	0.829	0.31	2.19E-49	13	Hsp90aa1
3.59E-53	0.379587369	0.256	0.033	4.73E-49	13	Fdx1l
3.76E-53	0.521379958	0.327	0.052	4.96E-49	13	Nudt5
5.25E-53	0.450954241	0.312	0.048	6.92E-49	13	Oat
6.91E-53	0.441783867	0.357	0.061	9.12E-49	13	Larp1
8.44E-53	0.618031925	0.598	0.149	1.11E-48	13	Nhp2l1
8.97E-53	-1.6017693	0.648	0.82	1.18E-48	13	Tyrobp
1.42E-52	0.475243606	0.256	0.034	1.88E-48	13	1190007I07Rik
2.00E-52	0.517399146	0.382	0.07	2.63E-48	13	Mrps28
2.44E-52	0.466622944	0.251	0.033	3.22E-48	13	Vkorc1l1
3.17E-52	0.6894447	0.99	0.716	4.18E-48	13	Rps26
4.26E-52	0.473924493	0.307	0.047	5.62E-48	13	Cpsf2
7.46E-52	0.426675089	0.332	0.054	9.84E-48	13	Rcor1
7.68E-52	0.535124425	0.492	0.107	1.01E-47	13	Wapl
8.45E-52	0.63377095	0.578	0.144	1.11E-47	13	Cdk6
9.34E-52	0.500334901	0.497	0.108	1.23E-47	13	Ssrp1
1.72E-51	0.752116746	0.98	0.633	2.28E-47	13	Rpl36a
2.35E-51	0.798080253	0.618	0.17	3.09E-47	13	Erdr1
2.55E-51	0.579940019	0.623	0.159	3.36E-47	13	Srrm1
2.73E-51	0.515016114	0.357	0.063	3.61E-47	13	Immt

5.03E-51	0.638550298	0.99	0.863	6.63E-47	13	Rpl32
7.61E-51	0.442759882	0.382	0.07	1.00E-46	13	Lgals9
1.38E-50	0.451557848	0.372	0.068	1.82E-46	13	Ubtf
1.68E-50	0.457062083	0.291	0.044	2.22E-46	13	Guk1
1.91E-50	0.556869772	0.427	0.087	2.52E-46	13	Kcnq1ot1
2.51E-50	0.392426571	0.276	0.04	3.31E-46	13	BC003965
2.58E-50	0.645800469	0.99	0.771	3.41E-46	13	Rplp2
2.61E-50	0.64178618	0.99	0.774	3.44E-46	13	Rpl26
2.62E-50	0.691004245	0.98	0.674	3.46E-46	13	Rpsa
3.46E-50	0.511638715	0.347	0.061	4.56E-46	13	Prdx3
3.70E-50	0.522361127	0.477	0.104	4.88E-46	13	Eif4ebp1
6.95E-50	0.511579211	0.492	0.11	9.16E-46	13	Bag1
8.73E-50	0.442210333	0.266	0.038	1.15E-45	13	Unc13d
9.51E-50	0.391395861	0.256	0.036	1.25E-45	13	Suclg2
1.98E-49	0.500975329	0.407	0.081	2.61E-45	13	Wdr61
2.22E-49	0.574876006	0.513	0.12	2.93E-45	13	Lsm7
2.80E-49	0.636887068	0.482	0.11	3.70E-45	13	Lsm2
4.58E-49	0.586953301	0.628	0.167	6.05E-45	13	Myb
4.90E-49	0.827016413	0.92	0.418	6.47E-45	13	Ybx1
5.22E-49	0.644726239	0.347	0.062	6.88E-45	13	Parp1
7.78E-49	0.427854694	0.256	0.036	1.03E-44	13	Nfix
1.69E-48	-1.844382732	0.412	0.74	2.23E-44	13	Cd52
2.32E-48	0.45195295	0.302	0.049	3.06E-44	13	Amd1
2.48E-48	0.400205772	0.281	0.043	3.28E-44	13	Timm17a
2.69E-48	0.463361062	0.322	0.055	3.54E-44	13	Bccip
2.83E-48	0.503496085	0.437	0.092	3.73E-44	13	Rpia
6.23E-48	0.539536791	0.357	0.067	8.22E-44	13	Cct3
9.43E-48	0.412274011	0.276	0.042	1.24E-43	13	Mak16
1.03E-47	0.57127501	0.583	0.152	1.36E-43	13	G3bp1
2.19E-47	0.533116142	0.422	0.088	2.89E-43	13	Hdac2
3.08E-47	0.640813791	0.814	0.278	4.06E-43	13	Pdcd4

3.91E-47	0.717165943	0.94	0.653	5.16E-43	13	Rps20
4.15E-47	0.457588979	0.276	0.043	5.47E-43	13	Mybbp1a
4.99E-47	0.657235105	0.729	0.222	6.58E-43	13	Ptges3
5.29E-47	0.540633313	0.508	0.122	6.98E-43	13	1110004F10Rik
5.43E-47	0.545000474	0.427	0.092	7.17E-43	13	Mrpl36
6.05E-47	0.43542364	0.276	0.043	7.98E-43	13	Zfp800
7.85E-47	0.743408196	0.764	0.266	1.04E-42	13	Hnrnpab
1.25E-46	0.505556476	0.387	0.078	1.64E-42	13	Psmd11
1.31E-46	0.524919117	1	0.936	1.73E-42	13	Rpl37a
1.39E-46	0.570773213	0.623	0.169	1.84E-42	13	Bsg
1.47E-46	0.44116675	0.271	0.042	1.93E-42	13	RP23-4H17.3
1.52E-46	0.58964708	0.598	0.161	2.00E-42	13	Ndufab1
1.55E-46	0.765108447	0.683	0.21	2.04E-42	13	Rpgrip1
1.85E-46	0.700919826	0.834	0.287	2.44E-42	13	mt-Nd2
2.15E-46	0.685781163	0.704	0.217	2.84E-42	13	Mif
2.69E-46	0.468448973	0.357	0.068	3.55E-42	13	Gm16286
5.31E-46	0.780272003	0.824	0.321	7.00E-42	13	Nop10
6.98E-46	0.567773457	0.412	0.088	9.21E-42	13	Eif4b
7.36E-46	0.470278231	0.533	0.131	9.71E-42	13	Lmo2
1.26E-45	0.468714954	0.347	0.065	1.66E-41	13	Ddx18
1.36E-45	0.366611276	0.251	0.037	1.80E-41	13	Chchd4
2.10E-45	0.472435535	0.377	0.076	2.76E-41	13	Gm10260
2.97E-45	0.508023844	0.497	0.12	3.92E-41	13	Dnajc19
3.57E-45	0.46727004	0.397	0.083	4.70E-41	13	Txn2
6.17E-45	0.436527943	0.462	0.105	8.13E-41	13	Eif4g1
1.42E-44	0.69105757	0.97	0.563	1.87E-40	13	Rpl10a
1.57E-44	0.401828858	0.291	0.049	2.07E-40	13	Eif2s1
2.18E-44	0.647834034	0.985	0.734	2.88E-40	13	Rpl23
2.50E-44	0.630039743	0.985	0.713	3.30E-40	13	Rpl27a
2.73E-44	0.430775367	0.322	0.059	3.61E-40	13	Aars
2.86E-44	0.38896989	0.327	0.06	3.77E-40	13	Ipo5

6.23E-44	0.437934208	0.332	0.062	8.22E-40	13	Rif1
6.64E-44	0.507554812	0.467	0.111	8.75E-40	13	Wbp5
7.67E-44	0.549866665	0.442	0.103	1.01E-39	13	Gadd45gip1
8.38E-44	0.34032129	0.281	0.046	1.11E-39	13	Msi2
1.43E-43	0.360271422	0.317	0.057	1.89E-39	13	Cdc34
1.44E-43	0.379964424	0.256	0.04	1.90E-39	13	Cul5
1.94E-43	0.655364855	0.734	0.238	2.56E-39	13	Eif3a
2.12E-43	0.554773579	1	0.893	2.80E-39	13	Rps19
3.26E-43	0.625943126	0.628	0.185	4.30E-39	13	Gm10020
3.89E-43	0.738137529	0.91	0.436	5.12E-39	13	Uqcr11
4.18E-43	0.667930382	0.975	0.578	5.52E-39	13	Rpl4
4.94E-43	0.469967111	0.432	0.098	6.52E-39	13	Cdv3
5.60E-43	0.686914918	0.799	0.29	7.39E-39	13	Polr2l
8.60E-43	0.700040082	0.95	0.567	1.13E-38	13	Rpl15
1.09E-42	0.399685045	0.312	0.056	1.44E-38	13	Rad23a
1.69E-42	0.745965771	0.91	0.38	2.23E-38	13	mt-Atp8
1.92E-42	0.632441476	0.829	0.301	2.53E-38	13	Eef1b2
3.42E-42	0.365328728	0.276	0.046	4.51E-38	13	Cir1
3.43E-42	0.442043974	0.417	0.093	4.53E-38	13	Wbp11
4.65E-42	0.57491138	0.593	0.169	6.14E-38	13	Tubb4b
6.09E-42	0.379053798	0.332	0.064	8.04E-38	13	Zc3h13
6.49E-42	0.731072414	0.764	0.287	8.57E-38	13	Erh
6.91E-42	0.805003315	0.925	0.495	9.12E-38	13	Cbx3
7.93E-42	0.372723101	0.251	0.04	1.05E-37	13	Sf3b4
9.55E-42	0.752770533	0.487	0.131	1.26E-37	13	Hist1h1b
9.59E-42	0.441136994	0.367	0.076	1.27E-37	13	Topbp1
9.78E-42	0.482525211	0.482	0.12	1.29E-37	13	Polr2f
2.66E-41	0.53507097	0.583	0.165	3.51E-37	13	Smarca5
2.95E-41	0.416637799	0.291	0.052	3.89E-37	13	Prkar2b
6.05E-41	0.412669375	0.563	0.15	7.98E-37	13	Sepw1
6.89E-41	0.427058087	0.342	0.069	9.09E-37	13	Nasp

1.20E-40	0.523469129	0.583	0.165	1.58E-36	13	Srsf10
1.27E-40	0.488135477	0.422	0.099	1.67E-36	13	Tsn
1.35E-40	0.432449069	0.427	0.1	1.78E-36	13	Eif1ax
1.43E-40	0.360217217	0.377	0.08	1.89E-36	13	Cyc1
1.53E-40	0.578693355	0.975	0.737	2.01E-36	13	Rpl24
1.72E-40	0.536165665	0.382	0.085	2.26E-36	13	Cdk4
1.80E-40	0.48169319	0.442	0.107	2.37E-36	13	Gatm
2.06E-40	0.669332745	0.94	0.597	2.72E-36	13	Rps21
2.09E-40	0.673752992	0.849	0.341	2.76E-36	13	Snrpf
2.69E-40	0.475481528	0.417	0.097	3.54E-36	13	Mrpl34
3.06E-40	0.488987762	0.271	0.048	4.04E-36	13	Psme3
3.26E-40	0.424791315	0.362	0.076	4.30E-36	13	Sod2
4.80E-40	0.424847786	0.322	0.063	6.33E-36	13	Trim28
5.32E-40	0.449850618	0.543	0.147	7.02E-36	13	Tardbp
6.92E-40	0.41160777	0.296	0.055	9.13E-36	13	Vars
8.87E-40	0.38948789	0.251	0.041	1.17E-35	13	Nt5c3
9.01E-40	0.620437117	0.985	0.713	1.19E-35	13	Rps17
9.32E-40	0.608778954	0.538	0.151	1.23E-35	13	Lig1
1.05E-39	0.335003301	0.322	0.063	1.38E-35	13	Rbm26
1.08E-39	0.783321856	0.96	0.609	1.43E-35	13	mt-Nd1
1.15E-39	0.473419851	0.417	0.099	1.51E-35	13	Cacybp
1.16E-39	0.474074969	0.342	0.071	1.53E-35	13	Trp53
1.22E-39	0.404976487	0.387	0.086	1.61E-35	13	Drap1
1.28E-39	0.475214098	0.276	0.049	1.69E-35	13	Wdhd1
1.41E-39	0.646910713	0.593	0.182	1.86E-35	13	St13
1.59E-39	0.422286967	0.442	0.107	2.09E-35	13	Eif3i
1.61E-39	0.471404315	0.432	0.104	2.12E-35	13	Naa15
2.47E-39	0.531614617	0.492	0.131	3.25E-35	13	Npm3
2.78E-39	0.472601658	0.387	0.088	3.66E-35	13	Txnl1
2.80E-39	0.501692726	0.563	0.161	3.69E-35	13	Rdx
2.98E-39	0.507564162	0.538	0.149	3.93E-35	13	Banf1

4.11E-39	0.674291783	0.879	0.388	5.42E-35	13	Rpl36-ps3
5.46E-39	0.550116451	0.99	0.77	7.20E-35	13	Rpl39
5.53E-39	0.490401161	0.503	0.133	7.29E-35	13	Mat2a
7.06E-39	0.613325029	0.97	0.666	9.32E-35	13	Gm2000
7.32E-39	0.660713343	0.774	0.29	9.66E-35	13	Hnrnpm
7.84E-39	0.46528564	0.347	0.074	1.03E-34	13	1810022K09Rik
7.96E-39	0.393881901	0.372	0.082	1.05E-34	13	Taf10
8.83E-39	0.501879578	0.457	0.116	1.16E-34	13	Bclaf1
1.06E-38	0.517329489	0.442	0.112	1.40E-34	13	Rnps1
1.09E-38	0.557836701	0.995	0.739	1.43E-34	13	Rps6
1.31E-38	-1.602401345	0.417	0.693	1.72E-34	13	Cyba
2.15E-38	0.438986871	0.503	0.134	2.84E-34	13	Rpl7a-ps5
2.21E-38	0.431583727	0.312	0.062	2.91E-34	13	Hcfc1
3.91E-38	0.641762743	0.93	0.461	5.15E-34	13	Rps26-ps1
4.00E-38	0.321791297	0.307	0.06	5.27E-34	13	Smap2
4.23E-38	0.413693679	0.397	0.093	5.58E-34	13	Denr
4.84E-38	0.415326246	0.352	0.076	6.39E-34	13	Cebpz
4.87E-38	0.4165196	0.387	0.089	6.43E-34	13	Rbbp7
5.14E-38	0.584906217	0.975	0.701	6.78E-34	13	Rpl28
6.00E-38	0.489814413	0.523	0.145	7.92E-34	13	Emg1
6.92E-38	0.457526796	0.437	0.109	9.12E-34	13	Tipin
9.16E-38	0.672896078	0.849	0.363	1.21E-33	13	Eif5a
1.13E-37	0.482930847	0.472	0.124	1.49E-33	13	Rsl24d1
1.57E-37	0.602751772	0.965	0.714	2.07E-33	13	Rpl6
1.71E-37	0.570126211	0.975	0.779	2.26E-33	13	Rps5
1.93E-37	0.43531912	0.442	0.112	2.55E-33	13	Snrnp70
2.00E-37	0.388385835	0.377	0.086	2.64E-33	13	Rcc2
2.12E-37	0.349359581	0.377	0.085	2.79E-33	13	Vdac1
2.34E-37	0.714330125	0.854	0.405	3.09E-33	13	Hsp90ab1
2.37E-37	0.577676231	0.764	0.268	3.12E-33	13	Gm10269
2.43E-37	0.489481171	0.618	0.19	3.20E-33	13	Srsf7

3.08E-37	0.431868725	0.266	0.049	4.07E-33	13	Kit
3.14E-37	-1.505774433	0.523	0.72	4.14E-33	13	Fcer1g
3.14E-37	0.363607858	0.397	0.094	4.14E-33	13	Sumo3
3.99E-37	0.349925351	0.322	0.066	5.27E-33	13	Mrpl41
4.13E-37	0.672523592	0.894	0.464	5.44E-33	13	Rps27l
4.13E-37	0.291414881	0.312	0.062	5.45E-33	13	Sars
6.62E-37	0.387701017	0.482	0.127	8.74E-33	13	Top2b
6.81E-37	0.768831965	0.583	0.191	8.98E-33	13	Supt16
7.14E-37	0.360258226	0.296	0.058	9.42E-33	13	Snhg6
7.19E-37	0.552487094	0.518	0.145	9.48E-33	13	Dpy30
7.33E-37	0.397854172	0.327	0.069	9.67E-33	13	Eif4e2
7.43E-37	0.446680968	0.508	0.14	9.80E-33	13	Mrps24
7.78E-37	0.50339853	0.296	0.059	1.03E-32	13	Eif1ad
1.04E-36	0.666096904	0.859	0.377	1.37E-32	13	Gm8186
1.06E-36	0.388072637	0.296	0.059	1.40E-32	13	C330006A16Rik
1.08E-36	0.372313981	0.377	0.087	1.42E-32	13	Dazap1
1.11E-36	0.549149119	0.513	0.144	1.46E-32	13	mt-Nd3
1.38E-36	0.410582046	0.357	0.08	1.82E-32	13	Tcp1
1.53E-36	0.649314547	0.859	0.37	2.02E-32	13	Eif2s2
1.60E-36	0.464031273	0.985	0.873	2.11E-32	13	Rps27a
2.75E-36	0.354608817	0.327	0.069	3.63E-32	13	Cd81
2.77E-36	0.552981593	0.628	0.203	3.65E-32	13	Slc25a3
3.05E-36	0.279658601	0.276	0.052	4.02E-32	13	Adk
3.06E-36	0.501451313	0.714	0.238	4.04E-32	13	Timm13
3.08E-36	0.590188956	0.709	0.245	4.06E-32	13	Atp5g3
3.49E-36	0.318101657	0.327	0.069	4.60E-32	13	Prorsd1
3.95E-36	0.3570954	0.437	0.11	5.22E-32	13	Tomm6
4.00E-36	0.570999742	0.98	0.682	5.28E-32	13	Rps7
4.45E-36	0.505305134	0.99	0.761	5.86E-32	13	Rpl21
4.92E-36	0.646158041	0.92	0.507	6.48E-32	13	Snrpg
5.96E-36	0.386051249	0.291	0.058	7.86E-32	13	Nap1l4

6.04E-36	0.458945356	0.447	0.116	7.97E-32	13	Mrpl42
6.46E-36	0.374197548	0.251	0.045	8.52E-32	13	Ktn1
8.53E-36	0.534518316	0.995	0.765	1.13E-31	13	Rps3a1
8.54E-36	0.427043069	0.357	0.081	1.13E-31	13	Dhx9
1.18E-35	0.432970941	0.266	0.05	1.56E-31	13	Myef2
1.21E-35	0.438622014	0.573	0.171	1.60E-31	13	Gm6133
1.39E-35	0.670333884	0.899	0.456	1.84E-31	13	Rps18-ps3
1.70E-35	0.374039629	0.472	0.126	2.25E-31	13	Ctbp1
2.30E-35	0.398836501	0.462	0.123	3.04E-31	13	Ubap2l
2.32E-35	0.289071726	0.261	0.048	3.06E-31	13	Aqr
2.49E-35	0.320158816	0.312	0.064	3.28E-31	13	Add1
2.89E-35	0.316440847	0.261	0.048	3.81E-31	13	Prpf19
3.21E-35	0.618263611	0.744	0.282	4.24E-31	13	Cd24a
3.61E-35	0.525517544	0.99	0.741	4.77E-31	13	Rps15a
3.64E-35	0.37614158	0.286	0.057	4.80E-31	13	Mzt1
3.74E-35	0.417817525	0.482	0.132	4.94E-31	13	Nol7
4.02E-35	0.509889571	0.442	0.117	5.31E-31	13	Picalm
5.09E-35	0.393671242	0.402	0.099	6.71E-31	13	Gps2
5.27E-35	0.439391048	0.367	0.087	6.96E-31	13	Mcm3
5.76E-35	0.579731707	0.97	0.649	7.60E-31	13	Eef1a1
6.37E-35	0.409320829	0.276	0.055	8.40E-31	13	Bola1
8.27E-35	0.484439847	0.99	0.845	1.09E-30	13	Rpl38
8.44E-35	0.561039729	0.965	0.718	1.11E-30	13	Rps4x
9.12E-35	0.358431295	0.281	0.056	1.20E-30	13	Rbmxl1
9.34E-35	0.483327109	1	0.837	1.23E-30	13	Ptma
9.87E-35	0.347323715	0.367	0.086	1.30E-30	13	Ddx24
1.01E-34	0.48934572	0.503	0.145	1.34E-30	13	Hnrnpa0
1.15E-34	0.529920083	0.97	0.754	1.52E-30	13	Rps3
1.43E-34	0.341144482	0.452	0.119	1.89E-30	13	Mrpl54
1.46E-34	0.396295018	0.437	0.114	1.93E-30	13	Celf1
1.46E-34	0.32706219	0.312	0.066	1.93E-30	13	Eif3b

1.60E-34	0.538511709	0.98	0.756	2.12E-30	13	Rpl17
1.71E-34	0.608924744	0.784	0.31	2.25E-30	13	Ranbp1
2.06E-34	0.352730954	0.281	0.056	2.72E-30	13	Cdk2ap1
2.27E-34	0.601022932	0.935	0.58	2.99E-30	13	Wdr89
2.43E-34	0.473892318	0.603	0.191	3.20E-30	13	Ssb
2.81E-34	0.556063114	0.95	0.634	3.71E-30	13	Rpl18
3.15E-34	0.344429865	0.261	0.05	4.15E-30	13	Aco2
3.16E-34	0.441179315	0.427	0.111	4.16E-30	13	Gtf2a2
3.24E-34	0.336221525	0.276	0.055	4.28E-30	13	Snrnp25
5.31E-34	0.395544259	0.276	0.055	7.01E-30	13	Ddb1
5.32E-34	0.411231795	0.437	0.116	7.02E-30	13	Syncrip
7.86E-34	0.309433177	0.357	0.084	1.04E-29	13	Cct2
9.94E-34	0.255126423	0.402	0.1	1.31E-29	13	Itga4
1.00E-33	0.338613782	0.377	0.091	1.32E-29	13	Larp7
1.04E-33	0.39391575	0.256	0.049	1.37E-29	13	Noc2l
1.30E-33	0.351551746	0.251	0.047	1.71E-29	13	Nip7
1.53E-33	0.359918155	0.397	0.099	2.01E-29	13	Fundc2
1.86E-33	0.307538284	0.352	0.082	2.46E-29	13	Asap1
2.04E-33	0.428629849	0.623	0.199	2.69E-29	13	Hnrnpc
2.26E-33	0.420803924	0.286	0.06	2.98E-29	13	Srrt
2.37E-33	-2.046319377	0.618	0.744	3.13E-29	13	Tmsb10
2.71E-33	0.454062285	1	0.898	3.57E-29	13	Rps28
5.27E-33	0.461137382	0.497	0.146	6.95E-29	13	Ndufa12
6.11E-33	0.528358286	0.749	0.279	8.07E-29	13	Nap1l1
6.70E-33	0.487949267	0.98	0.738	8.83E-29	13	Rpl9
7.60E-33	0.399668046	0.261	0.052	1.00E-28	13	Sgol1
8.00E-33	0.405718805	0.281	0.058	1.06E-28	13	Atp8a1
8.66E-33	0.392938122	0.362	0.088	1.14E-28	13	Cct4
1.04E-32	0.333505155	0.302	0.066	1.37E-28	13	1500011K16Rik
1.05E-32	0.413782323	0.462	0.13	1.38E-28	13	Bcas2
1.14E-32	0.492628002	0.653	0.224	1.50E-28	13	Park7

1.43E-32	0.544207449	0.96	0.648	1.89E-28	13	Rps10
1.55E-32	0.373422014	0.276	0.057	2.05E-28	13	Kdm2b
1.63E-32	0.341676207	0.276	0.057	2.15E-28	13	Pop5
2.85E-32	0.327256828	0.302	0.066	3.75E-28	13	Paics
3.04E-32	0.560866454	0.899	0.424	4.02E-28	13	Gnb2l1
3.08E-32	0.330833035	0.477	0.134	4.06E-28	13	Ssna1
3.16E-32	0.291768223	0.302	0.066	4.16E-28	13	Pdhb
3.50E-32	0.411800959	0.271	0.056	4.61E-28	13	Nudcd2
3.75E-32	0.265775704	0.276	0.057	4.95E-28	13	Taf12
4.18E-32	0.5708004	0.92	0.513	5.51E-28	13	Rpl22
4.40E-32	0.533906507	0.839	0.344	5.80E-28	13	Gpx4
6.13E-32	0.298368855	0.422	0.112	8.08E-28	13	Cdc26
7.05E-32	0.358855635	0.392	0.101	9.30E-28	13	U2af2
8.04E-32	-0.70770368	0.99	0.998	1.06E-27	13	Malat1
9.68E-32	0.628331979	0.899	0.493	1.28E-27	13	Rpl22l1
9.99E-32	0.438359542	0.467	0.136	1.32E-27	13	Morf4l2
1.09E-31	0.36339505	0.477	0.137	1.44E-27	13	Sod1
1.14E-31	0.484810632	0.98	0.737	1.51E-27	13	Rps15
1.35E-31	0.462596174	0.98	0.802	1.78E-27	13	Rps16
2.03E-31	0.267626957	0.271	0.056	2.68E-27	13	Polr2m
2.16E-31	0.32539785	0.322	0.075	2.85E-27	13	Rbm17
2.22E-31	0.436560679	0.633	0.213	2.93E-27	13	Lsm4
2.36E-31	0.48148326	0.97	0.701	3.11E-27	13	Rpl31
2.38E-31	0.611439594	0.824	0.358	3.14E-27	13	Srrm2
2.71E-31	-1.27558387	0.452	0.672	3.58E-27	13	Arhgdib
2.92E-31	0.422364066	0.995	0.878	3.85E-27	13	Rps14
3.39E-31	0.460149022	0.372	0.097	4.47E-27	13	Srpk1
4.33E-31	0.361204074	0.432	0.12	5.71E-27	13	Rpl13a-ps1
4.39E-31	0.439077463	0.367	0.095	5.79E-27	13	Mcm5
4.66E-31	0.4815899	0.98	0.814	6.15E-27	13	Rpl23a
5.21E-31	0.485733901	0.985	0.714	6.88E-27	13	Rpl19

5.48E-31	0.300023746	0.482	0.139	7.23E-27	13	Mrpl14
6.99E-31	0.314769708	0.357	0.089	9.22E-27	13	Ak2
9.13E-31	0.377334442	0.543	0.169	1.20E-26	13	Dnmt1
9.89E-31	0.294757017	0.286	0.063	1.30E-26	13	Mrps26
1.10E-30	0.372095168	0.377	0.098	1.46E-26	13	Cct7
1.28E-30	0.303374202	0.286	0.063	1.68E-26	13	Zcchc6
1.50E-30	0.540469476	0.834	0.371	1.98E-26	13	Hist1h2ap
1.59E-30	0.4215869	0.593	0.196	2.10E-26	13	Hnrnpd
1.78E-30	-1.178046495	0.538	0.697	2.35E-26	13	Sh3bgrl3
2.07E-30	0.261574295	0.307	0.07	2.74E-26	13	Utp11l
2.21E-30	0.334449679	0.407	0.109	2.92E-26	13	Scaf11
2.39E-30	0.356566283	0.302	0.069	3.15E-26	13	Ebna1bp2
2.64E-30	0.293840767	0.342	0.084	3.49E-26	13	Cse1l
2.90E-30	0.468523653	0.673	0.243	3.82E-26	13	Alyref
3.61E-30	0.519145543	0.698	0.257	4.76E-26	13	Romo1
3.91E-30	0.546922416	0.814	0.351	5.16E-26	13	Ran
4.12E-30	0.282016827	0.472	0.136	5.43E-26	13	Chchd1
4.19E-30	0.374811708	0.286	0.064	5.53E-26	13	Sf3b3
4.45E-30	0.473643866	0.789	0.306	5.87E-26	13	Gm10036
4.52E-30	0.337444196	0.276	0.06	5.97E-26	13	Gins1
5.02E-30	0.586211082	0.94	0.558	6.62E-26	13	mt-Nd4
5.43E-30	0.438039724	0.578	0.193	7.16E-26	13	Ewsr1
5.48E-30	0.277714544	0.332	0.08	7.23E-26	13	Sdhd
6.19E-30	0.391601681	0.447	0.13	8.17E-26	13	Eny2
6.25E-30	0.387465183	0.553	0.177	8.24E-26	13	Thoc7
6.28E-30	0.375276869	0.447	0.13	8.28E-26	13	1810043H04Rik
8.05E-30	0.478876814	0.633	0.227	1.06E-25	13	Cks1b
8.13E-30	0.424359421	0.698	0.249	1.07E-25	13	Prrc2c
8.40E-30	0.261703217	0.266	0.057	1.11E-25	13	Mbd3
9.03E-30	0.265451537	0.347	0.086	1.19E-25	13	Sltm
9.97E-30	0.30660567	0.392	0.105	1.31E-25	13	Fbl

1.26E-29	0.555393715	0.859	0.413	1.67E-25	13	Eef2
2.18E-29	0.396366463	0.407	0.114	2.88E-25	13	Pfdn6
2.35E-29	0.424635704	0.427	0.123	3.10E-25	13	Alkbh5
2.45E-29	0.301665861	0.266	0.057	3.23E-25	13	Bcl7c
2.61E-29	0.308892271	0.261	0.056	3.45E-25	13	Gm10146
3.68E-29	0.509304228	0.392	0.109	4.85E-25	13	Pfdn1
3.80E-29	0.354437552	0.432	0.123	5.01E-25	13	Nudt21
4.23E-29	-1.608554918	0.377	0.622	5.57E-25	13	Ly6c2
4.42E-29	0.327133231	0.628	0.211	5.84E-25	13	Snrpd1
5.38E-29	0.287218351	0.422	0.118	7.10E-25	13	Zc3h15
5.96E-29	0.466512418	0.513	0.165	7.86E-25	13	Mrpl18
6.20E-29	0.3744299	0.523	0.166	8.18E-25	13	Slbp
6.59E-29	0.303295887	0.296	0.069	8.69E-25	13	Etf1
7.11E-29	0.500788084	0.965	0.709	9.38E-25	13	Rpl11
7.33E-29	0.336410681	0.347	0.089	9.67E-25	13	Mrps17
7.97E-29	0.337561328	0.357	0.093	1.05E-24	13	Wdr83os
8.94E-29	0.378747859	0.312	0.076	1.18E-24	13	Psmd2
9.03E-29	0.254265163	0.357	0.092	1.19E-24	13	Cdc37
1.01E-28	0.394182011	0.477	0.147	1.34E-24	13	Tomm22
1.07E-28	0.360942521	0.312	0.076	1.41E-24	13	Ptbp1
1.30E-28	0.265979632	0.251	0.053	1.72E-24	13	Upf1
1.31E-28	0.313121893	0.332	0.083	1.73E-24	13	Atp13a3
1.36E-28	0.374704056	0.623	0.211	1.80E-24	13	Atp5a1
1.42E-28	0.395455566	0.518	0.165	1.88E-24	13	Mrpl23
1.67E-28	0.434172369	0.553	0.186	2.20E-24	13	Llph
1.77E-28	0.4292891	0.724	0.272	2.33E-24	13	Eif4a1
1.96E-28	0.254661295	0.412	0.115	2.59E-24	13	Plekhj1
1.98E-28	0.314090397	0.322	0.08	2.61E-24	13	Rad23b
2.06E-28	0.369277443	0.392	0.109	2.71E-24	13	Acin1
2.20E-28	0.292945052	0.307	0.074	2.91E-24	13	Tmem147
2.21E-28	0.414064303	0.467	0.142	2.91E-24	13	Hnrnpr

2.27E-28	0.331929468	0.291	0.068	2.99E-24	13	Ppp2r4
2.91E-28	0.368963165	0.256	0.056	3.84E-24	13	Orc6
2.92E-28	0.434689589	0.698	0.264	3.85E-24	13	Bola2
4.32E-28	0.414996071	0.377	0.105	5.70E-24	13	Uhrf1
4.32E-28	0.350738332	0.472	0.145	5.70E-24	13	1110001J03Rik
4.49E-28	0.376280265	0.513	0.165	5.92E-24	13	Polr1d
5.12E-28	0.269457799	0.291	0.068	6.76E-24	13	Zmynd8
5.37E-28	0.331919039	0.352	0.093	7.09E-24	13	Cyb5a
6.04E-28	0.366550665	0.432	0.128	7.97E-24	13	Timm8b
6.05E-28	0.289030939	0.467	0.141	7.98E-24	13	Uqcrfs1
6.07E-28	0.584128333	0.829	0.399	8.00E-24	13	Chchd2
6.18E-28	0.365759341	0.407	0.117	8.15E-24	13	Dctpp1
6.47E-28	0.379895279	0.608	0.21	8.54E-24	13	Ndufc2
6.51E-28	0.349504243	0.432	0.128	8.58E-24	13	Mrpl20
6.51E-28	0.328413323	0.482	0.149	8.59E-24	13	Rpa3
7.28E-28	0.269433204	0.296	0.071	9.61E-24	13	Polr2e
7.32E-28	0.32930901	0.312	0.077	9.66E-24	13	Cdca7
8.78E-28	0.36679865	0.427	0.126	1.16E-23	13	Naa50
9.81E-28	0.274936686	0.322	0.08	1.29E-23	13	Zfp106
1.21E-27	-2.049718957	1	0.999	1.60E-23	13	S100a8
1.74E-27	0.535606011	0.849	0.422	2.30E-23	13	Rpl13-ps3
1.93E-27	0.506966269	0.96	0.589	2.54E-23	13	Rpl3
2.23E-27	0.364858689	0.281	0.067	2.94E-23	13	Dnajc2
2.60E-27	0.361841342	0.286	0.069	3.43E-23	13	Commd7
2.62E-27	0.395508294	0.271	0.063	3.45E-23	13	Ikzf5
2.99E-27	0.282647849	0.452	0.136	3.94E-23	13	Pcnp
3.19E-27	0.368775216	0.568	0.194	4.20E-23	13	Ndufa5
3.31E-27	0.530048718	0.975	0.695	4.36E-23	13	mt-Cytb
3.61E-27	0.312794727	0.332	0.086	4.77E-23	13	Herc1
3.68E-27	0.340412411	0.497	0.158	4.85E-23	13	Srsf1
3.91E-27	0.382555002	0.492	0.158	5.16E-23	13	Papola

4.13E-27	0.398176996	0.995	0.87	5.44E-23	13	Rpl18a
4.44E-27	0.380341547	0.472	0.148	5.86E-23	13	Rpn1
6.43E-27	0.321331992	0.563	0.189	8.48E-23	13	Dnajc8
6.54E-27	0.389427793	0.503	0.165	8.63E-23	13	Siva1
7.92E-27	0.337332716	0.457	0.141	1.04E-22	13	Cnbp
9.58E-27	0.294724984	0.372	0.103	1.26E-22	13	Eif3m
1.07E-26	0.438010757	0.332	0.089	1.41E-22	13	Hist1h1e
1.11E-26	0.367430846	0.467	0.147	1.46E-22	13	Dnaja1
1.14E-26	0.272278817	0.276	0.065	1.51E-22	13	Dph3
1.46E-26	0.293847782	0.482	0.152	1.92E-22	13	Tceb1
1.54E-26	0.279246832	0.357	0.097	2.04E-22	13	Cct5
1.70E-26	0.302804352	0.276	0.066	2.24E-22	13	Fbxo3
2.07E-26	0.360204196	0.503	0.165	2.73E-22	13	Uqcc2
2.48E-26	0.311224117	0.332	0.088	3.27E-22	13	Hmgn5
2.74E-26	0.289238674	0.382	0.108	3.61E-22	13	Snrpc
2.79E-26	0.481779205	0.317	0.084	3.68E-22	13	Ccne2
3.09E-26	0.347954463	0.492	0.16	4.08E-22	13	Pdcd5
3.68E-26	0.520820548	0.905	0.499	4.86E-22	13	Pabpc1
4.38E-26	0.288005382	0.271	0.064	5.78E-22	13	Lage3
4.48E-26	0.34489594	0.251	0.057	5.91E-22	13	Eif2a
5.66E-26	0.408362319	0.608	0.222	7.47E-22	13	Snrpd3
6.21E-26	0.527524165	0.854	0.419	8.19E-22	13	Cox17
8.42E-26	-1.68871034	0.186	0.511	1.11E-21	13	Cybb
8.48E-26	0.498839068	0.749	0.313	1.12E-21	13	Cks2
8.84E-26	0.379243993	0.673	0.252	1.17E-21	13	Ndufs6
9.22E-26	0.529346784	0.839	0.394	1.22E-21	13	Anp32b
9.58E-26	0.407503363	0.447	0.143	1.26E-21	13	Prdx6
1.20E-25	0.360381717	0.437	0.137	1.58E-21	13	Nmt1
1.21E-25	0.51854879	0.859	0.421	1.60E-21	13	Gm9493
1.40E-25	0.338549034	0.603	0.212	1.84E-21	13	Mrps14
1.59E-25	0.262431545	0.503	0.162	2.09E-21	13	Rp9

1.62E-25	0.453602471	0.975	0.72	2.14E-21	13	Rps25
1.65E-25	0.504776923	0.91	0.536	2.18E-21	13	Rpl7
1.71E-25	0.477741634	0.759	0.323	2.25E-21	13	Nucks1
1.79E-25	0.335923469	0.422	0.129	2.36E-21	13	Ythdf2
2.21E-25	0.462502409	0.879	0.417	2.92E-21	13	Rpl5
2.29E-25	-0.933466205	0.995	0.999	3.02E-21	13	Actb
2.44E-25	0.321120928	0.342	0.094	3.22E-21	13	R3hdm1
3.42E-25	0.31747687	0.548	0.187	4.51E-21	13	AI662270
3.74E-25	0.351154468	0.312	0.083	4.94E-21	13	Ndufa10
4.10E-25	0.412422619	0.658	0.25	5.41E-21	13	Snrpd2
4.30E-25	0.35116793	0.508	0.172	5.68E-21	13	Pcna
4.67E-25	0.278342502	0.291	0.074	6.17E-21	13	Chtop
4.75E-25	0.298192645	0.266	0.064	6.26E-21	13	Eif3d
4.83E-25	0.328997712	0.417	0.129	6.37E-21	13	Polr2j
5.53E-25	0.401636907	0.327	0.091	7.30E-21	13	Mcm6
5.58E-25	-1.276665077	0.508	0.649	7.36E-21	13	Prdx5
5.63E-25	0.346331697	0.382	0.113	7.43E-21	13	Cenpw
5.96E-25	0.26734017	0.332	0.09	7.86E-21	13	Eif3g
6.04E-25	0.444004775	0.809	0.359	7.97E-21	13	Srsf2
9.28E-25	0.339115734	0.307	0.081	1.22E-20	13	Emc6
1.17E-24	0.30893066	0.266	0.065	1.54E-20	13	Hectd1
1.20E-24	0.384683705	0.256	0.062	1.58E-20	13	Tmx1
1.25E-24	0.29764073	0.492	0.164	1.65E-20	13	Creg1
1.64E-24	0.496816007	0.995	0.855	2.16E-20	13	mt-Atp6
1.64E-24	0.499506012	0.889	0.451	2.17E-20	13	Rpl30
1.65E-24	0.313246764	0.538	0.188	2.17E-20	13	Caprin1
1.81E-24	0.252798596	0.362	0.104	2.39E-20	13	Zc3hav1
1.82E-24	0.378264219	0.276	0.07	2.41E-20	13	Ube2e3
2.47E-24	0.264634049	0.523	0.175	3.26E-20	13	Mrpl57
2.70E-24	0.320422998	0.307	0.082	3.56E-20	13	Pkig
2.74E-24	-2.084430173	0.995	0.994	3.62E-20	13	S100a9

2.81E-24	0.405111409	0.332	0.094	3.70E-20	13	Thrap3
2.89E-24	0.312694633	0.477	0.156	3.82E-20	13	Stra13
3.17E-24	-1.253070892	0.598	0.687	4.18E-20	13	Plac8
4.24E-24	0.266548592	0.286	0.073	5.59E-20	13	Maz
4.42E-24	0.301526573	0.256	0.062	5.83E-20	13	Actl6a
4.48E-24	0.315959174	0.332	0.093	5.91E-20	13	Sdha
6.12E-24	0.342269312	0.663	0.252	8.07E-20	13	Snrpb
6.51E-24	-1.411452201	0.362	0.589	8.58E-20	13	S100a11
7.46E-24	0.371067897	0.663	0.255	9.84E-20	13	Ndufb9
7.76E-24	0.473845915	0.779	0.354	1.02E-19	13	Tuba1b
8.79E-24	-1.418836425	0.261	0.536	1.16E-19	13	Coro1a
1.13E-23	0.266959667	0.467	0.153	1.48E-19	13	Ube2i
1.26E-23	0.288240941	0.261	0.065	1.66E-19	13	Tmem33
1.31E-23	-0.750989727	0.784	0.78	1.72E-19	13	Shfm1
1.76E-23	0.294138438	0.513	0.177	2.32E-19	13	Srsf6
2.12E-23	0.354775008	0.332	0.095	2.79E-19	13	Suz12
2.38E-23	0.314052225	0.995	0.975	3.13E-19	13	Rps29
4.22E-23	0.453249231	0.784	0.355	5.56E-19	13	Dek
4.57E-23	0.338724806	0.588	0.218	6.02E-19	13	Csnk1a1
7.02E-23	0.270939525	0.312	0.086	9.27E-19	13	Cdca3
7.32E-23	0.296606904	0.467	0.159	9.65E-19	13	Higd1a
8.15E-23	0.269553019	0.296	0.08	1.08E-18	13	Arl6ip4
8.56E-23	0.493436459	0.869	0.488	1.13E-18	13	Rpl7a
1.00E-22	0.3591029	0.508	0.182	1.32E-18	13	Sf3b5
1.11E-22	0.348534245	0.704	0.286	1.47E-18	13	Atp5g2
1.16E-22	0.38573707	0.608	0.237	1.53E-18	13	Rbm25
1.16E-22	0.318362062	0.588	0.218	1.53E-18	13	Anp32e
1.20E-22	-1.077251038	0.523	0.653	1.58E-18	13	Itm2b
1.29E-22	0.29964778	0.608	0.229	1.70E-18	13	Ube2s
1.46E-22	0.354384841	0.477	0.168	1.93E-18	13	Lsm3
1.70E-22	0.29050429	0.513	0.18	2.24E-18	13	Elavl1

1.99E-22	0.297349997	0.513	0.182	2.63E-18	13	Hnrnpl
2.29E-22	0.313620689	0.467	0.161	3.02E-18	13	Cbx1
2.50E-22	0.397776913	0.92	0.484	3.29E-18	13	Tubb5
2.81E-22	0.255059883	0.251	0.063	3.71E-18	13	Casc5
5.47E-22	0.254475508	0.327	0.095	7.22E-18	13	Usp34
6.02E-22	0.258367754	0.427	0.141	7.94E-18	13	Chd4
6.84E-22	0.309183355	0.377	0.119	9.02E-18	13	Dnajc9
7.03E-22	0.395795428	0.945	0.689	9.27E-18	13	Oaz1
8.20E-22	0.286312412	0.407	0.132	1.08E-17	13	Brd7
8.68E-22	0.391303913	0.98	0.681	1.15E-17	13	Hmgb1
9.81E-22	0.298957962	0.352	0.108	1.29E-17	13	0610012G03Rik
9.84E-22	0.273382284	0.271	0.073	1.30E-17	13	Eif4h
1.10E-21	0.420535294	0.759	0.338	1.46E-17	13	Rpl6l
1.27E-21	0.269162907	0.337	0.101	1.68E-17	13	U2surp
1.39E-21	0.436056539	0.925	0.663	1.83E-17	13	Rpl23a-ps3
1.47E-21	0.286545557	0.322	0.095	1.94E-17	13	Galnt1
1.50E-21	0.364510167	0.97	0.8	1.97E-17	13	Rpl34
1.57E-21	0.272298769	0.337	0.101	2.07E-17	13	Hjurp
1.77E-21	0.250978087	0.271	0.073	2.34E-17	13	Gm6483
1.78E-21	0.424097525	0.935	0.672	2.35E-17	13	Rpl27
2.01E-21	0.335980936	0.291	0.082	2.65E-17	13	Plk4
3.25E-21	0.261543047	0.523	0.191	4.28E-17	13	Atp5d
3.45E-21	0.427918142	0.779	0.37	4.55E-17	13	Minos1
3.92E-21	0.345775992	0.302	0.088	5.17E-17	13	Ccna2
3.97E-21	0.385679633	0.312	0.093	5.24E-17	13	Wdr76
6.13E-21	0.266707798	0.548	0.207	8.09E-17	13	Mki67
1.08E-20	0.257172081	0.357	0.112	1.42E-16	13	Ubxn1
1.20E-20	0.373253152	0.266	0.074	1.59E-16	13	Tspan32
1.41E-20	0.25472304	0.251	0.067	1.86E-16	13	Ipo7
1.51E-20	0.386377349	0.678	0.296	1.99E-16	13	Canx
1.96E-20	0.251296903	0.266	0.073	2.59E-16	13	Prrc2a

2.35E-20	0.387091643	0.698	0.305	3.10E-16	13	Snrpe	
3.37E-20	0.396282107	0.683	0.307	4.45E-16	13	Top2a	
4.54E-20	0.259049702	0.256	0.07	5.99E-16	13	Asxl2	
5.01E-20	0.403823623	0.95	0.637	6.61E-16	13	Gm10263	
5.37E-20	0.417784074	0.854	0.435	7.08E-16	13	Rpl9-ps6	
5.47E-20	0.311733176	0.302	0.091	7.22E-16	13	Aimp1	
6.04E-20	-0.893285472	0.658	0.687	7.97E-16	13	Actg1	
6.29E-20	0.300803086	0.307	0.093	8.30E-16	13	Ankrd13a	
6.93E-20	0.257812127	0.317	0.096	9.14E-16	13	Baz1b	
8.41E-20	0.305760638	0.327	0.101	1.11E-15	13	Fam104a	
8.48E-20	0.459452944	0.714	0.326	1.12E-15	13	Srsf3	
8.84E-20	0.320337641	0.985	0.869	1.17E-15	13	Rpl35a	
9.22E-20	0.26268799	0.472	0.173	1.22E-15	13	Trmt112	
1.53E-19	0.315543365	0.648	0.27	2.02E-15	13	Atp5b	
1.97E-19	0.30457682	0.558	0.218	2.59E-15	13	Smc2	
2.23E-19	0.300122065	0.367	0.123	2.94E-15	13	Usp1	
2.73E-19	0.31465606	0.724	0.308	3.60E-15	13	Hnrnpu	
2.85E-19	0.259683587	0.402	0.139	3.76E-15	13	Mrpl51	
2.92E-19	0.268814622	0.643	0.265	3.85E-15	13	Arglu1	
4.19E-19	0.344581289	0.99	0.799	5.53E-15	13	Rps24	
5.63E-19	0.326087547	0.673	0.289	7.42E-15	13	Sfpq	
7.53E-19	0.331055998	0.276	0.083	9.93E-15	13	Lsm12	
8.30E-19	-0.800990715	0.704	0.709	1.09E-14	13	Myl6	
9.18E-19	-1.977690977	1	0.929	1.21E-14	13	Camp	
1.15E-18	0.280539243	0.382	0.132	1.52E-14	13		2-Sep
1.56E-18	0.390973746	0.754	0.35	2.05E-14	13	Eif4g2	
1.71E-18	0.285431876	0.372	0.128	2.26E-14	13	Pole4	
2.22E-18	0.312771576	0.271	0.081	2.93E-14	13	Clasp2	
2.93E-18	0.339321049	0.588	0.241	3.87E-14	13	Ndufb2	
3.09E-18	0.406725575	0.889	0.563	4.07E-14	13	Cox8a	
3.18E-18	0.256692713	0.266	0.078	4.20E-14	13	Rbm6	

3.55E-18	0.394003965	0.462	0.177	4.69E-14	13	Lockd
4.16E-18	0.284477357	0.623	0.261	5.49E-14	13	Atp5o
5.67E-18	0.400735337	0.925	0.575	7.48E-14	13	Gm26917
6.82E-18	0.369755998	0.824	0.415	8.99E-14	13	Sumo2
7.09E-18	0.273390474	0.256	0.075	9.36E-14	13	Tpx2
7.64E-18	0.341839105	0.271	0.083	1.01E-13	13	Mapk1ip1l
7.97E-18	0.283654407	0.628	0.267	1.05E-13	13	mt-Nd4l
8.59E-18	-1.277967298	0.603	0.633	1.13E-13	13	Cst3
1.07E-17	0.366620983	1	0.953	1.42E-13	13	mt-Co3
1.51E-17	0.351606111	0.704	0.313	1.99E-13	13	Eif3f
1.53E-17	0.309543882	0.266	0.081	2.02E-13	13	Stxbp2
1.61E-17	0.265655199	0.377	0.133	2.12E-13	13	Hnrnpdl
2.55E-17	-0.537144654	0.884	0.834	3.37E-13	13	Pfn1
2.61E-17	0.349114166	0.94	0.684	3.45E-13	13	Rps13
3.01E-17	0.312297724	0.296	0.096	3.97E-13	13	Hells
3.26E-17	0.365783844	0.462	0.184	4.29E-13	13	Rrm2
3.38E-17	-1.584070771	0.372	0.537	4.46E-13	13	S100a6
3.47E-17	0.312059104	0.668	0.295	4.58E-13	13	Pcbp1
4.17E-17	-2.078255794	0.935	0.847	5.51E-13	13	Ngp
5.64E-17	0.257421716	0.382	0.139	7.44E-13	13	Rbm15
7.14E-17	0.266366036	0.367	0.132	9.42E-13	13	Psma5
1.13E-16	0.272427432	0.583	0.246	1.49E-12	13	Ndufb8
2.22E-16	-1.74996402	0.452	0.556	2.93E-12	13	Chil3
2.73E-16	0.275204296	0.402	0.153	3.61E-12	13	Kdelr2
7.85E-16	-1.196863728	0.231	0.451	1.04E-11	13	Arpc1b
9.86E-16	0.386687788	0.91	0.594	1.30E-11	13	Cox7c
1.00E-15	0.279459658	0.814	0.393	1.32E-11	13	Uqcrq
1.30E-15	0.318416533	0.744	0.365	1.72E-11	13	Btf3
2.84E-15	0.271958097	0.688	0.312	3.74E-11	13	Slc25a5
3.43E-15	-1.556705002	0.457	0.562	4.52E-11	13	Ifitm3
4.52E-15	-1.323770929	0.271	0.46	5.96E-11	13	Hp

4.56E-15	-1.140717977	0.246	0.451	6.01E-11	13	Gmfg
4.68E-15	0.307766827	0.839	0.455	6.17E-11	13	Rpl10-ps3
1.21E-14	0.306096537	0.819	0.44	1.59E-10	13	Naca
2.79E-14	0.289223045	0.276	0.097	3.68E-10	13	Vamp3
2.92E-14	0.343187186	0.925	0.622	3.85E-10	13	Uba52
3.77E-14	0.262928865	0.603	0.272	4.97E-10	13	Tra2b
4.50E-14	-1.145830265	0.266	0.457	5.94E-10	13	Alox5ap
5.60E-14	-1.130245018	0.312	0.483	7.38E-10	13	F630028O10Rik
6.00E-14	0.302995462	0.834	0.463	7.91E-10	13	Rpl27-ps3
8.75E-14	-1.610847544	0.146	0.367	1.15E-09	13	Ifitm6
1.82E-13	0.269943779	0.945	0.688	2.40E-09	13	Rpl10
2.75E-13	-1.325950131	0.161	0.37	3.62E-09	13	Lst1
3.50E-13	-0.642763267	0.849	0.768	4.62E-09	13	B2m
9.88E-13	0.344311581	0.844	0.503	1.30E-08	13	Cox6a1
1.30E-12	0.314463565	0.829	0.471	1.71E-08	13	Uqcrh
1.32E-12	0.369007203	0.935	0.646	1.75E-08	13	mt-Co2
1.38E-12	-1.438442962	0.276	0.434	1.82E-08	13	Anxa1
1.68E-12	0.291467439	0.819	0.442	2.22E-08	13	Uqcr10
2.08E-12	-1.988025333	0.533	0.559	2.75E-08	13	Lcn2
2.36E-12	-1.398271968	0.352	0.482	3.12E-08	13	Lgals1
3.11E-12	-1.207555992	0.131	0.334	4.11E-08	13	Cd53
3.51E-12	0.291017128	0.884	0.534	4.63E-08	13	Hspa8
4.14E-12	0.264685962	0.739	0.387	5.47E-08	13	Tomm7
4.59E-12	0.310794124	0.874	0.482	6.05E-08	13	Pcbp2
5.13E-12	-1.665024901	0.322	0.452	6.77E-08	13	Pglyrp1
6.68E-12	-1.463152767	0.668	0.636	8.82E-08	13	Crip1
9.41E-12	0.303770742	0.804	0.439	1.24E-07	13	Hnrnpa3
1.17E-11	-1.921713431	0.729	0.627	1.54E-07	13	Wfdc21
1.45E-11	-0.983351719	0.382	0.493	1.91E-07	13	Arpc2
2.00E-11	-1.164334343	0.497	0.545	2.63E-07	13	Psap
2.27E-11	-1.300000062	0.302	0.443	2.99E-07	13	Lgals3

3.86E-11	-1.067415405	0.146	0.338	5.09E-07	13	Clec12a
4.49E-11	-1.314358132	0.07	0.263	5.93E-07	13	Gm9844
4.84E-11	0.285642078	0.91	0.602	6.38E-07	13	Gm11808
9.65E-11	-0.964010897	0.342	0.468	1.27E-06	13	Rac2
6.07E-10	0.260580481	0.859	0.523	8.01E-06	13	Atp5k
7.83E-10	-1.022536969	0.121	0.298	1.03E-05	13	Pkm
1.06E-09	0.278746411	0.894	0.558	1.40E-05	13	Hnrnpa2b1
1.16E-09	-1.063329235	0.085	0.261	1.53E-05	13	Vim
1.18E-09	-1.160347587	0.09	0.263	1.55E-05	13	Cebpb
1.55E-09	-1.082190914	0.101	0.272	2.04E-05	13	Cotl1
3.04E-09	-1.224355895	0.161	0.322	4.01E-05	13	Ms4a6c
8.39E-09	-1.067701658	0.101	0.263	0.000110634	13	Lmo4
1.43E-08	-0.841925062	0.608	0.576	0.00018875	13	H2-D1
2.68E-08	-1.546681971	0.236	0.363	0.000353601	13	Ifi27l2a
2.82E-08	-0.940415674	0.211	0.353	0.000371879	13	Ptpn18
5.48E-08	-0.753338345	0.583	0.554	0.000722984	13	Calm1
1.03E-07	-0.883097471	0.653	0.585	0.00135736	13	Hmgn2
1.08E-07	-1.041488251	0.146	0.284	0.001420505	13	Lcp1
1.33E-07	-1.056356153	0.131	0.27	0.001748052	13	Clec4a2
1.43E-07	-1.692349705	0.593	0.54	0.001886789	13	Ltf
1.63E-07	-0.932062779	0.146	0.285	0.002150319	13	Myl12b
2.98E-07	-1.012045459	0.126	0.26	0.003925332	13	Anxa2
3.73E-07	-1.057237508	0.201	0.32	0.004918669	13	Lsp1
3.90E-07	-0.405724048	0.839	0.711	0.005149553	13	Cfl1
5.79E-07	-0.99073131	0.201	0.319	0.007638333	13	Ptprc
8.37E-07	-0.861807166	0.136	0.268	0.011040066	13	Ncf1
3.84E-06	-0.879169602	0.226	0.326	0.050695817	13	Celf2
4.53E-06	-0.898700726	0.286	0.368	0.059693889	13	Gsr
6.15E-06	-0.996472548	0.191	0.294	0.081082408	13	Mgst1
6.63E-06	-0.850722118	0.276	0.358	0.087475101	13	Fam49b
6.73E-06	-0.314507775	0.91	0.775	0.088836342	13	Serf2

7.47E-06	-0.871814627	0.291	0.368	0.098501568	13	Emp3
9.81E-06	-0.90765355	0.352	0.398	0.129411822	13	Dstn
1.16E-05	-1.124982135	0.176	0.277	0.153664278	13	Ccr2
1.91E-05	-0.725265916	0.518	0.489	0.252183527	13	Gnai2
2.32E-05	-0.750682908	0.372	0.418	0.306114915	13	Cdc42
3.70E-05	-0.829345022	0.286	0.355	0.48822139	13	Msrb1
5.46E-05	-0.777040178	0.367	0.399	0.720559486	13	Actr3
8.50E-05	-0.718443495	0.417	0.432	1	13	Tspo
8.88E-05	-0.807624797	0.322	0.369	1	13	Capza2
0.00010148	-0.328464516	0.884	0.771	1	13	H3f3a
0.00018128	-0.915682396	0.226	0.296	1	13	Sat1
0.00023722	-0.898698241	0.206	0.281	1	13	Slpi
0.00025323	-0.784066795	0.286	0.338	1	13	Clic1
0.00042551	-0.813858927	0.286	0.327	1	13	Lamtor4
0.00066892	-0.274276255	0.322	0.181	1	13	Samhd1
0.00096463	-1.202403824	0.216	0.274	1	13	Prtn3
0.00101901	-0.787254316	0.241	0.295	1	13	Laptm5
0.00147178	-0.254020166	0.422	0.243	1	13	Sec11c
0.00160933	-0.676521618	0.492	0.44	1	13	Actr2
0.00178532	-0.274509354	0.327	0.189	1	13	R3hdm4
0.00209414	-0.772893997	0.196	0.253	1	13	Psmb8
0.00245127	-0.479638842	0.633	0.539	1	13	Ubb
0.0029823	-0.74416011	0.236	0.28	1	13	Iqgap1
0.00319739	-0.253618707	0.402	0.24	1	13	Pgls
0.00359906	-0.252847756	0.402	0.239	1	13	Scand1
0.00362011	-0.696919213	0.226	0.276	1	13	Aldh2
0.00376379	-0.712500189	0.226	0.27	1	13	Gpi1
0.00382053	-0.723110765	0.432	0.402	1	13	Dbi
0.00426097	-0.720067873	0.211	0.257	1	13	Flna
0.00478977	-0.740345445	0.563	0.466	1	13	Ly6e
0.00562657	-0.284666677	0.397	0.237	1	13	Zfp36l2

0.00570629	-0.508879524	0.658	0.532	1	13	Mrpl33	
0.00644451	-0.867584936	0.211	0.251	1	13	Tmcc1	
0.00767072	-0.271174622	0.362	0.22	1	13	Krtcap2	
0	3.704401026	0.811	0.051	0	14	Ccl5	**NK cell**
0	2.867954177	0.849	0.068	0	14	AW112010	
0	1.894545332	0.378	0.004	0	14	Klre1	
1.73E-295	2.053616497	0.486	0.017	2.29E-291	14	Ms4a4b	
8.74E-274	1.662068667	0.319	0.005	1.15E-269	14	Gimap4	
3.80E-273	2.528164299	0.422	0.013	5.01E-269	14	Gzma	
2.45E-266	1.781701077	0.335	0.007	3.24E-262	14	Klrk1	
5.00E-194	1.856840933	0.514	0.036	6.59E-190	14	Klrd1	
6.46E-138	2.080505891	0.741	0.136	8.52E-134	14	Nkg7	
1.40E-54	1.272813378	0.416	0.087	1.84E-50	14	Ptprcap	
1.38E-51	1.324755407	0.514	0.139	1.82E-47	14	Id2	
6.83E-44	1.415572347	0.373	0.086	9.00E-40	14	Ccnd2	
2.06E-38	0.592842993	1	0.955	2.71E-34	14	Rps27	
1.09E-34	0.522065957	1	0.997	1.44E-30	14	Malat1	
2.45E-32	-1.203219805	0.654	0.913	3.23E-28	14	Lyz2	
3.61E-32	1.056815844	0.649	0.306	4.76E-28	14	Ptprc	
7.67E-30	1.008299282	0.254	0.056	1.01E-25	14	1-Sep	
1.31E-29	1.034316445	0.357	0.103	1.73E-25	14	Shisa5	
2.27E-29	0.617454793	0.968	0.804	2.99E-25	14	Rpl13a	
2.71E-29	0.647379451	0.962	0.841	3.58E-25	14	Rps27rt	
4.13E-29	0.556683314	0.984	0.733	5.45E-25	14	Tmsb10	
1.15E-26	-0.901679092	0.405	0.755	1.52E-22	14	Gpx1	
5.24E-26	0.956596168	0.643	0.35	6.91E-22	14	H2-K1	
2.68E-24	1.051146741	0.351	0.118	3.53E-20	14	Jak1	
6.61E-24	0.548300645	0.951	0.803	8.73E-20	14	Rps16	
5.61E-23	0.49630201	0.973	0.848	7.39E-19	14	Rpl13	
5.64E-23	0.965236811	0.438	0.179	7.43E-19	14	Hcst	
1.26E-21	0.600040016	0.908	0.744	1.66E-17	14	Rps15a	

1.16E-20	-1.007712629	0.4	0.692	1.53E-16	14	Plac8
3.28E-20	0.561624866	0.914	0.717	4.33E-16	14	Rplp1
1.58E-19	0.515753435	0.935	0.76	2.08E-15	14	Rpl36
2.02E-19	0.773398565	0.551	0.287	2.66E-15	14	Pdcd4
3.79E-19	0.401458641	0.995	0.937	5.00E-15	14	Rpl37a
3.57E-18	0.525742093	0.876	0.653	4.71E-14	14	Eef1a1
6.59E-18	0.364726805	0.995	0.954	8.70E-14	14	Rpl41
8.14E-18	0.50644942	0.946	0.815	1.07E-13	14	Rpl23a
9.39E-18	0.444717524	0.93	0.801	1.24E-13	14	Rps24
1.94E-17	0.387712344	0.989	0.878	2.56E-13	14	Rps14
2.00E-17	-1.076509863	0.249	0.568	2.63E-13	14	Ifitm3
2.73E-17	-0.825184444	0.346	0.654	3.60E-13	14	Prdx5
1.92E-16	-1.01914592	0.195	0.51	2.54E-12	14	Cybb
2.90E-16	0.482616156	0.957	0.846	3.83E-12	14	Rpl38
3.24E-16	0.432522219	0.941	0.833	4.27E-12	14	Rps18
3.29E-16	0.865153375	0.292	0.109	4.33E-12	14	Ets1
9.50E-16	-0.572585217	0.514	0.788	1.25E-11	14	Shfm1
2.16E-15	0.496402153	0.87	0.686	2.85E-11	14	Rps7
4.52E-15	0.45444195	0.903	0.781	5.96E-11	14	Rps5
1.06E-14	0.439863565	0.892	0.757	1.39E-10	14	Rps3
3.84E-14	0.40271929	0.957	0.865	5.06E-10	14	Rpl32
6.28E-14	-0.858205993	0.168	0.459	8.28E-10	14	Alox5ap
8.67E-14	-0.838508782	0.389	0.639	1.14E-09	14	Cst3
1.21E-13	0.396168106	0.951	0.806	1.60E-09	14	Rps23
1.32E-13	0.430071279	0.908	0.72	1.74E-09	14	Rps4x
1.62E-13	0.469180701	0.827	0.679	2.13E-09	14	Rpsa
2.45E-13	0.679214856	0.465	0.248	3.23E-09	14	Btg1
3.10E-13	0.464402562	0.811	0.626	4.09E-09	14	Uba52
3.89E-13	-0.937943094	0.141	0.404	5.13E-09	14	Dstn
4.08E-13	0.447119101	0.876	0.717	5.38E-09	14	Rpl27a
4.46E-13	0.443639219	0.849	0.675	5.88E-09	14	Rpl27

4.50E-13	0.4378195	0.876	0.726	5.93E-09	14	Cd52
5.26E-13	-1.11522098	0.405	0.637	6.94E-09	14	Wfdc21
1.08E-12	0.382532885	0.903	0.802	1.42E-08	14	Rpl34
1.74E-12	0.380528662	0.941	0.773	2.30E-08	14	Rplp2
2.03E-12	-0.911015228	0.319	0.559	2.68E-08	14	Chil3
2.82E-12	-0.664647044	0.314	0.589	3.72E-08	14	S100a11
3.36E-12	0.43890105	0.849	0.638	4.43E-08	14	Rpl36a
5.00E-12	0.329463712	0.978	0.9	6.59E-08	14	Rpl37
5.02E-12	-0.70102124	0.368	0.622	6.63E-08	14	Ly6c2
5.97E-12	0.35066874	0.978	0.871	7.87E-08	14	Rpl18a
7.99E-12	0.481627277	0.778	0.64	1.05E-07	14	Rpl18
8.33E-12	-0.56760005	0.67	0.825	1.10E-07	14	Ftl1
9.40E-12	0.472142707	0.762	0.57	1.24E-07	14	Rpl10a
1.25E-11	-0.963232641	0.13	0.371	1.64E-07	14	Lst1
1.52E-11	0.462002935	0.827	0.719	2.01E-07	14	Rpl19
1.68E-11	0.49404916	0.811	0.667	2.21E-07	14	Rpl23a-ps3
1.69E-11	0.432136745	0.859	0.687	2.23E-07	14	Rps13
1.93E-11	0.311207906	0.995	0.898	2.54E-07	14	Rps28
2.63E-11	-0.774528499	0.211	0.462	3.47E-07	14	Hp
2.67E-11	0.458100956	0.789	0.643	3.53E-07	14	Rpl8
3.95E-11	0.338327055	0.897	0.743	5.21E-07	14	Rps6
4.02E-11	0.339088795	0.865	0.768	5.30E-07	14	B2m
4.44E-11	0.35522076	0.941	0.79	5.86E-07	14	Rps11
4.85E-11	0.365503136	0.903	0.759	6.40E-07	14	Rpl17
6.87E-11	-1.13978552	1	0.999	9.06E-07	14	S100a8
8.82E-11	-1.068021017	0.73	0.853	1.16E-06	14	Ngp
2.05E-10	0.424067639	0.762	0.607	2.71E-06	14	Gm11808
2.17E-10	-1.150254161	0.984	0.995	2.86E-06	14	S100a9
2.17E-10	-0.781057234	0.124	0.347	2.87E-06	14	Ifitm2
2.49E-10	0.301341984	0.984	0.854	3.28E-06	14	Rpl35
3.29E-10	0.376122066	0.838	0.707	4.34E-06	14	Rplp0

4.80E-10	0.402867192	0.827	0.704	6.33E-06	14	Tpt1
5.15E-10	0.438213445	0.773	0.595	6.80E-06	14	Rpl3
5.20E-10	0.42565012	0.805	0.703	6.86E-06	14	Sub1
7.28E-10	-0.627980436	0.378	0.594	9.60E-06	14	Hmgn2
7.79E-10	-0.914685207	0.108	0.323	1.03E-05	14	Ms4a6c
9.67E-10	-1.061990526	0.914	0.932	1.27E-05	14	Camp
1.27E-09	-0.790844202	0.124	0.338	1.67E-05	14	Clec12a
1.28E-09	-0.772373345	0.335	0.549	1.68E-05	14	Psap
2.06E-09	0.570720948	0.557	0.399	2.71E-05	14	Rpl36-ps3
2.08E-09	0.348852742	0.897	0.749	2.74E-05	14	Gm10076
2.46E-09	0.382366275	0.773	0.654	3.25E-05	14	Rps10
2.58E-09	-1.005401761	0.162	0.366	3.40E-05	14	Ifitm6
7.98E-09	0.337140605	0.87	0.741	0.000105226	14	Rps15
8.61E-09	-0.787429538	0.243	0.444	0.000113571	14	Lgals3
1.22E-08	0.554042541	0.427	0.259	0.000160821	14	S100a10
1.41E-08	0.376378374	0.827	0.714	0.000186525	14	Rpl11
1.49E-08	-0.882628779	0.389	0.563	0.000196068	14	Lcn2
1.53E-08	-0.411836673	0.6	0.78	0.000202081	14	H3f3a
1.63E-08	0.334454569	0.708	0.573	0.000215409	14	H2-D1
1.66E-08	0.499867096	0.595	0.43	0.000218797	14	Gm9493
1.99E-08	-0.646928177	0.276	0.484	0.000262621	14	F630028O10Rik
2.20E-08	-0.767443956	0.108	0.302	0.000290453	14	Cd24a
2.33E-08	-0.474659245	0.454	0.654	0.000307683	14	Itm2b
2.88E-08	0.666734779	0.368	0.223	0.00038036	14	Nme1
3.17E-08	-0.68048813	0.092	0.28	0.000418356	14	Aldh2
3.42E-08	0.320596394	0.892	0.765	0.000451124	14	Rpl21
4.22E-08	0.472522476	0.6	0.435	0.000556463	14	Gm10073
6.77E-08	0.333511234	0.897	0.834	0.00089359	14	Pfn1
7.83E-08	0.28379876	0.87	0.769	0.001032863	14	Rps3a1
8.21E-08	0.412806905	0.627	0.47	0.001083198	14	Rpl27-ps3
1.19E-07	0.408653922	0.416	0.253	0.001563777	14	Gm9844

1.29E-07	-0.553038224	0.254	0.456	0.001698031	14	Atpif1
1.39E-07	0.448511506	0.568	0.431	0.001835509	14	Rpl13-ps3
1.42E-07	-0.649021843	0.178	0.369	0.001876072	14	Serp1
1.45E-07	0.572879061	0.341	0.204	0.00191841	14	Limd2
1.62E-07	-0.607222993	0.151	0.338	0.00213789	14	Notch2
1.82E-07	0.429614486	0.686	0.576	0.002395687	14	Rpl15
2.20E-07	0.381975855	0.627	0.465	0.002905437	14	Gm8730
2.90E-07	0.349521316	0.827	0.743	0.003819541	14	Rpl9
3.00E-07	0.298984009	0.843	0.681	0.003962204	14	Rpl14
3.37E-07	-0.683740201	0.124	0.296	0.004447417	14	Mgst1
3.77E-07	0.347349849	0.708	0.55	0.004971315	14	Rps2
4.44E-07	0.486417874	0.389	0.247	0.005853145	14	Psmb8
6.08E-07	0.333172106	0.735	0.604	0.008018544	14	Rps21
6.25E-07	0.254907386	0.897	0.773	0.008241589	14	Rpl39
6.29E-07	-0.614540078	0.195	0.371	0.00829447	14	Gsr
6.34E-07	0.369673284	0.627	0.48	0.008360613	14	Rpl12
6.52E-07	-0.844726293	0.195	0.364	0.008603158	14	Ifi27l2a
8.85E-07	-1.192148088	0.119	0.274	0.011678019	14	Mpo
8.94E-07	-0.613996719	0.259	0.434	0.011787017	14	Anxa1
9.10E-07	-0.907029822	0.411	0.545	0.012002599	14	Ltf
1.06E-06	-0.671344264	0.103	0.262	0.013995429	14	Lmo4
1.47E-06	0.403596008	0.627	0.502	0.01942587	14	Rpl22l1
1.54E-06	0.281104794	0.832	0.67	0.020258675	14	Gm2000
1.58E-06	-0.52886779	0.162	0.331	0.020897921	14	Lamtor4
1.77E-06	0.358523561	0.751	0.66	0.023354829	14	Rps20
1.86E-06	0.327734877	0.735	0.586	0.024508641	14	Wdr89
2.07E-06	0.533533554	0.281	0.16	0.027348492	14	Sepw1
2.53E-06	-1.086075464	0.13	0.277	0.03339859	14	Prtn3
2.63E-06	0.303713693	0.822	0.719	0.034633425	14	Rpl6
3.20E-06	0.254887	0.859	0.741	0.042274855	14	Rpl24
3.46E-06	0.327232325	0.757	0.643	0.045587134	14	Gm10263

3.98E-06	0.42117046	0.476	0.344	0.05247782	14	Ptpn18
4.16E-06	-0.579058153	0.2	0.358	0.054925353	14	Msrb1
4.91E-06	0.553252092	0.27	0.157	0.064745436	14	Itgal
5.48E-06	-0.661147516	0.124	0.27	0.072224461	14	Clec4a2
8.80E-06	0.257660958	0.789	0.655	0.116105547	14	Rps12
9.22E-06	0.466744827	0.524	0.424	0.121574432	14	Eef2
9.64E-06	-0.71956025	0.324	0.451	0.127113036	14	Pglyrp1
9.76E-06	0.496976579	0.314	0.198	0.128724465	14	Psme1
1.02E-05	-0.411429702	0.146	0.309	0.135152443	14	Taldo1
1.26E-05	-0.375130585	0.546	0.688	0.165746169	14	Cyba
1.27E-05	-0.317610236	0.135	0.283	0.167967591	14	Slpi
1.28E-05	-0.515684465	0.119	0.263	0.16839583	14	Aprt
1.28E-05	0.316767084	0.611	0.474	0.168503011	14	Lgals1
1.50E-05	-0.465152699	0.135	0.285	0.197998277	14	Anapc13
1.56E-05	0.538573699	0.33	0.217	0.205437987	14	Selplg
1.68E-05	-0.445025614	0.211	0.379	0.221815044	14	Tmem256
1.94E-05	-0.424209753	0.265	0.428	0.256147478	14	Tkt
1.97E-05	-0.581883795	0.13	0.267	0.259742587	14	Ncf1
2.31E-05	-0.561271229	0.178	0.326	0.305075787	14	Npc2
2.36E-05	0.340588815	0.578	0.502	0.311860215	14	Mbnl1
2.41E-05	-0.417321567	0.168	0.331	0.317300912	14	Cks2
2.49E-05	-0.310087025	0.67	0.783	0.328073778	14	Hmgb2
2.56E-05	-0.325334731	0.368	0.541	0.337167441	14	Mrpl33
2.69E-05	0.252723887	0.849	0.738	0.354477411	14	Rpl23
2.95E-05	-0.439670424	0.189	0.337	0.388589775	14	Irf2bp2
3.39E-05	-0.378820583	0.227	0.395	0.446998544	14	Smdt1
3.39E-05	-0.416450601	0.2	0.358	0.44754345	14	Atp6v0e
3.93E-05	0.400399942	0.6	0.466	0.517758106	14	Rps18-ps3
4.02E-05	-0.560904761	0.4	0.536	0.52999585	14	S100a6
4.70E-05	0.358948495	0.33	0.209	0.620608631	14	Ahnak
4.79E-05	-0.463513349	0.173	0.323	0.631825183	14	Gm10116

5.95E-05	0.357431806	0.557	0.435	0.784997257	14	Gnb2l1
8.38E-05	-0.373497517	0.189	0.331	1	14	Ap3s1
0.0001262	-0.577606748	0.141	0.261	1	14	Cebpb
0.00014819	-0.394219502	0.232	0.372	1	14	Capza2
0.00014912	0.446697127	0.292	0.198	1	14	Hmha1
0.00015512	-0.342807493	0.151	0.292	1	14	Sdcbp
0.00016537	-0.419958405	0.168	0.296	1	14	Pkm
0.00016678	0.41301706	0.384	0.281	1	14	Eef1g
0.00018617	-0.450752663	0.135	0.261	1	14	Gm10282
0.00021616	-0.385119548	0.146	0.273	1	14	Gpi1
0.00021652	0.32702318	0.562	0.462	1	14	Rpl30
0.00021655	0.253458185	0.811	0.692	1	14	Rpl10
0.00023015	-0.348001753	0.141	0.274	1	14	Aldoa
0.0002886	-0.299599776	0.227	0.374	1	14	Myeov2
0.00031049	-0.338910132	0.319	0.455	1	14	Uqcr11
0.00040799	0.420804438	0.47	0.402	1	14	Pfdn5
0.00050235	-0.34679751	0.319	0.452	1	14	Psma7
0.00050502	0.301025166	0.524	0.446	1	14	Rpl9-ps6
0.00050572	0.300528158	0.557	0.461	1	14	Rac2
0.00050643	0.258388693	0.643	0.542	1	14	Hspa8
0.00055036	-0.310404197	0.195	0.322	1	14	Top2a
0.00064148	-0.335256925	0.276	0.415	1	14	Atox1
0.00064614	-0.355924242	0.173	0.297	1	14	Ppp1cb
0.00077148	0.475651908	0.308	0.227	1	14	Sell
0.0008127	0.283207225	0.524	0.428	1	14	Rpl5
0.00091044	0.323494993	0.649	0.568	1	14	mt-Nd4
0.00106518	0.352305324	0.33	0.243	1	14	Fxyd5
0.00115494	-0.294578589	0.346	0.494	1	14	Gnai2
0.00120957	-0.297040144	0.173	0.29	1	14	Ndufv3
0.00127156	-0.31137765	0.178	0.297	1	14	Nap1l1
0.00142406	-0.272020769	0.389	0.511	1	14	Sec61b

0.0016568	-0.285371105	0.249	0.371	1	14	Dek	
0.00179645	-0.299250964	0.232	0.352	1	14	Vamp8	
0.0020138	-0.290134661	0.292	0.418	1	14	Gapdh	
0.00206554	-0.362729602	0.184	0.287	1	14	Cd44	
0.00215674	-0.474393915	0.162	0.253	1	14	Tmcc1	
0.00228756	-0.419750653	0.243	0.348	1	14	H2afy	
0.00236986	0.250730949	0.605	0.546	1	14	Rpl7	
0.00238393	0.430058618	0.308	0.233	1	14	Abhd2	
0.00280948	0.2577653	0.438	0.354	1	14	Gm42418	
0.00374175	0.301817923	0.27	0.192	1	14	Ywhaq	
0.00386862	0.403801424	0.368	0.3	1	14	Myl12a	
0.00468398	0.317684171	0.411	0.349	1	14	Rpl6l	
0.00476843	0.386058951	0.314	0.237	1	14	Pycard	
0.00480911	-0.311609192	0.162	0.259	1	14	Flna	
0.0050104	-0.33383826	0.232	0.338	1	14	Hnrnpa1	
0.00554081	0.350560692	0.276	0.208	1	14	Dad1	
0.00581459	-0.356817524	0.195	0.284	1	14	Myl12b	
0.00739335	-0.269178546	0.162	0.254	1	14	Ndufb3	
0	2.591873469	0.633	0.009	0	15	Ebf1	**B cell**
0	2.576918097	0.586	0.016	0	15	Vpreb3	
0	1.944254021	0.539	0.01	0	15	Cd79a	
7.50E-248	1.343925773	0.266	0.003	9.89E-244	15	Pou2af1	
2.06E-238	1.59877493	0.273	0.003	2.72E-234	15	Myl4	
1.66E-113	1.837911193	0.312	0.016	2.19E-109	15	Mzb1	
2.05E-113	1.650392673	0.359	0.022	2.70E-109	15	Cd79b	
2.83E-90	1.626583177	0.398	0.037	3.74E-86	15	Chchd10	
9.17E-40	1.110673437	0.273	0.038	1.21E-35	15	Pafah1b3	
1.88E-35	1.130032038	0.5	0.126	2.47E-31	15	Ly6d	
1.19E-21	0.990592992	0.336	0.091	1.57E-17	15	Ptprcap	
5.11E-17	0.943402238	0.586	0.29	6.74E-13	15	Cd24a	
7.50E-16	-0.815784974	0.578	0.82	9.89E-12	15	Tyrobp	

1.18E-15	-0.445377956	1	0.996	1.56E-11	15	Tmsb4x
2.77E-15	0.79310773	0.406	0.153	3.66E-11	15	Cd74
1.39E-14	0.889380555	0.352	0.13	1.84E-10	15	Dnajc7
2.08E-14	0.819843309	0.375	0.14	2.74E-10	15	Foxp1
2.49E-14	0.526226815	1	0.997	3.28E-10	15	Malat1
2.01E-12	-0.67275869	0.453	0.719	2.65E-08	15	Fcer1g
3.03E-11	0.644768488	0.953	0.84	3.99E-07	15	Ptma
1.91E-10	-0.712881314	0.828	0.907	2.52E-06	15	Lyz2
8.36E-10	0.886691808	0.766	0.689	1.10E-05	15	Hmgb1
8.76E-10	0.751745928	0.312	0.135	1.16E-05	15	Paip2
4.71E-09	0.336822827	0.992	0.956	6.21E-05	15	Rps27
4.94E-09	0.688344561	0.578	0.372	6.52E-05	15	H2afv
6.73E-09	0.355844564	0.938	0.842	8.88E-05	15	Rps27rt
3.07E-08	0.69219566	0.281	0.119	0.000405303	15	Mef2c
3.71E-08	0.708829757	0.266	0.115	0.000488847	15	Hmgn1
6.98E-08	-0.895292261	0.359	0.556	0.000920268	15	Chil3
1.67E-07	0.653096552	0.273	0.124	0.00220417	15	Dnajc9
1.70E-07	0.748114862	0.297	0.139	0.002240667	15	Pim1
2.23E-07	0.523268005	0.438	0.245	0.002939086	15	Rbm25
2.23E-07	0.66325689	0.367	0.201	0.002944583	15	Zfp706
3.58E-07	0.334569057	0.875	0.7	0.004722098	15	mt-Cytb
4.66E-07	-0.649378924	0.258	0.482	0.006147936	15	F630028O10Rik
7.15E-07	-0.712079804	0.258	0.458	0.00943799	15	Hp
8.75E-07	-0.867694295	0.375	0.562	0.011546545	15	Ifitm3
1.26E-06	-0.744723767	0.133	0.336	0.01665931	15	Clec12a
1.81E-06	0.282570119	0.922	0.774	0.023830777	15	Rpl39
2.25E-06	-0.756134144	0.25	0.442	0.029680489	15	Lgals3
2.61E-06	-0.528876929	0.492	0.648	0.034407159	15	Prdx5
3.09E-06	-0.545414549	0.414	0.585	0.040774902	15	S100a11
3.68E-06	-0.549455585	0.32	0.505	0.048573295	15	Cybb
5.35E-06	-0.346432474	0.664	0.782	0.070550111	15	Serf2

7.23E-06	0.723658072	0.367	0.227	0.095356313	15	Anp32e
8.54E-06	0.314295271	0.797	0.634	0.112586941	15	Xist
1.38E-05	0.561675083	0.32	0.178	0.181864773	15	Myb
1.41E-05	0.488375984	0.445	0.28	0.18627863	15	Luc7l2
1.56E-05	0.526019664	0.305	0.167	0.206132371	15	Cbx1
1.60E-05	-0.379625563	0.609	0.747	0.211154815	15	Gpx1
1.91E-05	0.490910569	0.258	0.131	0.251394347	15	Ctcf
2.56E-05	0.598100833	0.547	0.41	0.337706353	15	Stmn1
2.57E-05	-0.694130192	0.281	0.451	0.339084845	15	Pglyrp1
2.98E-05	0.358205162	0.859	0.773	0.393190996	15	H3f3a
3.17E-05	0.560155873	0.406	0.251	0.418501202	15	Btg1
3.63E-05	-0.453901311	0.477	0.617	0.478533014	15	Ly6c2
4.06E-05	0.304845528	0.727	0.567	0.534956295	15	mt-Nd4
4.23E-05	-0.337105924	0.688	0.782	0.557861251	15	Shfm1
5.59E-05	0.431502908	0.32	0.185	0.73742333	15	Pnisr
5.62E-05	-0.579166905	0.266	0.433	0.741613089	15	Anxa1
5.65E-05	0.648392697	0.367	0.238	0.744649437	15	Ube2s
5.87E-05	0.507175351	0.312	0.181	0.774285465	15	Erdr1
6.59E-05	0.495973289	0.469	0.333	0.869722435	15	Nucks1
6.60E-05	-0.731577875	0.508	0.633	0.870518729	15	Wfdc21
7.59E-05	-0.527268544	0.391	0.539	1	15	Mrpl33
7.80E-05	0.791860371	0.5	0.383	1	15	Hist1h2ap
7.97E-05	-0.324664082	0.75	0.822	1	15	Ftl1
8.80E-05	-0.554121409	0.305	0.454	1	15	Alox5ap
9.70E-05	-0.712755921	0.352	0.48	1	15	Lgals1
0.0001073	0.419018742	0.562	0.425	1	15	Rbm3
0.00011249	-0.367738687	1	0.999	1	15	Actb
0.00011251	0.4881586	0.359	0.224	1	15	Nme1
0.0001226	-0.49394202	0.164	0.322	1	15	Gm10116
0.00013627	0.609658616	0.445	0.316	1	15	Top2a
0.00014363	-0.529256195	0.211	0.369	1	15	Gsr

0.00014514	0.258568212	0.852	0.707	1	15	Rpl28
0.00015344	0.470042299	0.391	0.256	1	15	Ucp2
0.00017808	0.480056395	0.258	0.146	1	15	Phip
0.00019674	0.351804949	0.352	0.212	1	15	Pdap1
0.0002073	-0.538099913	0.25	0.399	1	15	Dstn
0.00021378	0.576422004	0.336	0.215	1	15	Mki67
0.00022164	-0.498803282	0.406	0.546	1	15	Psap
0.00023933	0.279562113	0.594	0.441	1	15	Ddx5
0.00026811	0.651600822	0.266	0.161	1	15	H2afx
0.00028506	-0.345806316	0.594	0.686	1	15	Cyba
0.00032985	0.287230856	0.719	0.588	1	15	Rpl4
0.00037274	0.352435905	0.281	0.165	1	15	Mtdh
0.00040083	0.550609947	0.359	0.243	1	15	Pgls
0.00049085	-0.71031787	0.234	0.363	1	15	Ifitm6
0.00050317	-0.579115776	0.133	0.26	1	15	Cebpb
0.00053066	0.506093635	0.266	0.163	1	15	Ppp1cc
0.00063053	-0.329642392	0.586	0.695	1	15	Sh3bgrl3
0.00063751	-0.529371301	0.133	0.258	1	15	Flna
0.00068172	0.310444882	0.391	0.26	1	15	Snrpd2
0.00072237	0.372288445	0.812	0.74	1	15	H3f3b
0.00089203	-0.3234751	0.562	0.686	1	15	Plac8
0.00096216	-0.529947235	0.242	0.368	1	15	Emp3
0.00100209	-0.611841049	0.43	0.544	1	15	Ltf
0.00104312	0.38182268	0.492	0.365	1	15	Tuba1b
0.00106265	0.31627421	0.57	0.449	1	15	Usmg5
0.00120809	0.358258531	0.258	0.156	1	15	Tra2a
0.00126104	0.35593752	0.328	0.213	1	15	Atrx
0.00129211	0.346625606	0.305	0.194	1	15	Smc4
0.00129785	-0.349335785	0.227	0.372	1	15	Myh9
0.00130001	0.318322226	0.766	0.704	1	15	Sub1
0.00133231	0.345829272	0.273	0.169	1	15	Ube2b

0.00137812	0.542321104	0.43	0.331	1	15	2810417H13Rik
0.00138345	-0.701992568	1	0.999	1	15	S100a8
0.00154358	0.343035082	0.461	0.339	1	15	Set
0.00157595	0.476766234	0.281	0.183	1	15	Lockd
0.001653	0.355802123	0.289	0.181	1	15	2310036O22Rik
0.00166688	-0.275012776	0.586	0.688	1	15	Actg1
0.00168817	0.278827991	0.375	0.25	1	15	Timm13
0.00169852	0.383147079	0.312	0.203	1	15	Ikzf1
0.00172384	-0.431967136	0.195	0.33	1	15	Ap3s1
0.00176293	0.516947827	0.344	0.237	1	15	Pycard
0.00188961	-0.326875805	0.602	0.666	1	15	Arhgdib
0.00195461	0.372689529	0.258	0.161	1	15	Rtf1
0.00202179	0.27695026	0.656	0.523	1	15	Rpl22
0.00204845	-0.39927462	0.312	0.447	1	15	Arpc1b
0.00215133	0.383721962	0.469	0.371	1	15	Srrm2
0.00216271	0.380479494	0.266	0.17	1	15	Adrbk1
0.00231752	0.311746474	0.398	0.279	1	15	Hnrnpab
0.00234806	0.293686244	0.555	0.429	1	15	Serinc3
0.00237098	0.387785742	0.391	0.282	1	15	Nsa2
0.00242536	-0.741656785	0.828	0.85	1	15	Ngp
0.00260084	0.32788292	0.43	0.315	1	15	Eef1b2
0.00273359	-0.282234911	0.617	0.711	1	15	Myl6
0.00276193	0.298005537	0.523	0.406	1	15	Rbm39
0.00277637	0.280639439	0.562	0.432	1	15	Rpl13-ps3
0.00283906	-0.37193332	0.312	0.434	1	15	Tspo
0.00301091	0.397913762	0.289	0.193	1	15	St13
0.00320953	0.528352744	0.617	0.559	1	15	Calm2
0.0033598	0.338141015	0.273	0.175	1	15	Slbp
0.00354852	-0.966364212	0.18	0.275	1	15	Prtn3
0.00373364	0.363634493	0.375	0.268	1	15	Romo1
0.00408663	-0.540408971	0.211	0.319	1	15	Ms4a6c

0.00422954	0.386835689	0.578	0.496	1	15	Tubb5	
0.00425655	-0.432554283	0.172	0.283	1	15	Myl12b	
0.00428179	0.268746492	0.406	0.297	1	15	Atp5g2	
0.00435421	-0.34161178	0.555	0.651	1	15	Itm2b	
0.00487921	0.310013063	0.305	0.206	1	15	Hnrnpd	
0.00501501	0.301921815	0.258	0.167	1	15	Rbbp4	
0.00547776	0.274945555	0.547	0.416	1	15	Hsp90ab1	
0.00583276	0.288486095	0.328	0.225	1	15	Psma2	
0.00586676	0.337215526	0.57	0.507	1	15	Cbx3	
0.0060025	0.254379473	0.422	0.312	1	15	Eif3k	
0.00603252	0.33311841	0.258	0.171	1	15	Cenpa	
0.00639235	-0.432482261	0.164	0.268	1	15	Cotl1	
0.00689936	-0.835029433	0.172	0.272	1	15	Mpo	
0.00699956	-0.376616813	0.547	0.634	1	15	Cst3	
0.00714262	0.386955478	0.258	0.171	1	15	U2af1	
0.00729056	0.337021545	0.43	0.336	1	15	Srsf3	
0.0083245	0.32225369	0.508	0.401	1	15	Rbx1	
0.00861108	0.292144387	0.477	0.374	1	15	Btf3	
0.00889578	-0.495753306	0.18	0.276	1	15	Ccr2	
0.00899742	0.346928732	0.469	0.361	1	15	Serp1	
0.00928026	-0.3495025	0.227	0.328	1	15	Lamtor4	
0.0095063	0.524357296	0.289	0.205	1	15	Birc5	
0	2.939350322	0.847	0.01	0	16	Cpa3	**Basophils**
0	2.34928309	0.605	0.005	0	16	Ms4a2	
0	1.763654053	0.323	0.002	0	16	Fcer1a	
4.94E-296	1.75261457	0.403	0.007	6.52E-292	16	Csrp3	
8.17E-288	4.687911158	0.823	0.05	1.08E-283	16	Prss34	
1.96E-282	5.256454258	0.887	0.063	2.59E-278	16	Mcpt8	
2.69E-244	1.92224622	0.492	0.017	3.54E-240	16	Gata2	
1.41E-138	2.20151892	0.532	0.042	1.86E-134	16	Ifitm1	
2.76E-127	1.336061282	0.347	0.017	3.64E-123	16	Edem3	

4.95E-98	1.35473123	0.339	0.022	6.53E-94	16	Ikzf2
7.98E-56	1.618553887	0.726	0.213	1.05E-51	16	Cd63
2.61E-53	1.446120163	0.621	0.146	3.44E-49	16	Ctsg
2.55E-43	1.118898022	0.282	0.036	3.37E-39	16	Ptprs
2.05E-37	1.1690288	0.395	0.08	2.70E-33	16	Nedd4
6.86E-37	1.333265348	0.298	0.047	9.04E-33	16	Spry2
1.39E-33	1.201167784	0.597	0.193	1.83E-29	16	Srgn
6.52E-33	1.229712454	0.435	0.107	8.60E-29	16	Runx1
5.10E-31	1.167547869	0.629	0.236	6.73E-27	16	Calr
1.78E-28	1.150515796	0.323	0.068	2.35E-24	16	Hdc
8.24E-28	0.685320678	0.702	0.262	1.09E-23	16	Mpo
2.28E-27	-1.500383643	0.718	0.91	3.00E-23	16	Lyz2
1.00E-25	0.599385363	0.992	0.846	1.32E-21	16	Rpl38
1.26E-25	1.082566793	0.395	0.107	1.67E-21	16	Gng12
2.34E-25	1.028624909	0.371	0.095	3.08E-21	16	Fam46a
2.48E-24	1.170709538	0.597	0.251	3.28E-20	16	Lmo4
7.68E-24	1.019362852	0.331	0.081	1.01E-19	16	Igf1r
4.18E-23	0.707072365	0.944	0.679	5.51E-19	16	Plac8
8.82E-22	1.012216473	0.347	0.096	1.16E-17	16	Pdia4
1.13E-21	-1.491438223	0.21	0.622	1.49E-17	16	Ly6c2
8.41E-20	0.958014813	0.266	0.064	1.11E-15	16	Nucb1
7.03E-18	0.924681254	0.387	0.133	9.28E-14	16	Csgalnact2
8.13E-17	-1.618364967	0.202	0.559	1.07E-12	16	Chil3
4.74E-15	0.550935145	0.823	0.471	6.25E-11	16	Lgals1
5.92E-15	1.032169576	0.306	0.1	7.80E-11	16	Hyou1
1.78E-14	-1.453504817	0.21	0.538	2.34E-10	16	S100a6
6.98E-14	0.859265435	0.444	0.199	9.20E-10	16	Ssr4
1.13E-13	0.786392027	0.589	0.322	1.49E-09	16	Ap3s1
1.15E-13	0.976888235	0.444	0.2	1.52E-09	16	Hspa5
1.26E-13	0.845742633	0.371	0.146	1.66E-09	16	Manf
1.74E-13	0.791628072	0.597	0.336	2.30E-09	16	Hsp90b1

2.90E-13	0.627779938	0.75	0.463	3.82E-09	16	Ly6e
4.81E-13	0.407147016	0.952	0.802	6.35E-09	16	Rps24
5.76E-13	0.991890536	0.306	0.11	7.60E-09	16	Ets1
5.86E-13	0.40600554	0.968	0.842	7.72E-09	16	Rps27rt
7.38E-13	0.375455056	0.984	0.791	9.74E-09	16	Rps11
9.08E-13	0.458809375	0.952	0.773	1.20E-08	16	Rpl39
1.14E-12	0.371292246	0.992	0.9	1.50E-08	16	Rpl37
2.20E-12	-1.123314802	0.21	0.507	2.90E-08	16	Cybb
5.17E-12	-0.788860304	0.435	0.689	6.81E-08	16	Cyba
1.07E-11	0.333201226	1	0.937	1.41E-07	16	Rpl37a
1.21E-11	0.320256052	0.992	0.956	1.59E-07	16	Rps27
1.53E-11	0.660966022	0.548	0.3	2.01E-07	16	Tsix
1.94E-11	-1.144114359	0.266	0.549	2.56E-07	16	Psap
2.10E-11	0.618711602	0.532	0.279	2.77E-07	16	Cmtm7
2.42E-11	0.538596342	0.274	0.093	3.19E-07	16	Ptprcap
2.74E-11	-0.778502883	0.524	0.734	3.62E-07	16	Cd52
3.82E-11	0.463653957	0.339	0.13	5.03E-07	16	Apoe
4.42E-11	0.428912924	0.911	0.691	5.83E-07	16	Rpl10
4.76E-11	-1.571954659	0.298	0.546	6.27E-07	16	Ltf
5.34E-11	0.409781927	0.976	0.807	7.04E-07	16	Rps23
1.05E-10	0.429296678	0.895	0.738	1.39E-06	16	Rpl23
2.13E-10	0.44377789	0.911	0.67	2.81E-06	16	Gm2000
3.24E-10	0.441453186	0.871	0.64	4.27E-06	16	Rpl36a
8.21E-10	0.384929827	0.984	0.833	1.08E-05	16	Rps8
8.27E-10	-1.462404662	0.879	0.932	1.09E-05	16	Camp
8.35E-10	0.692425728	0.427	0.221	1.10E-05	16	Krtcap2
1.47E-09	-0.940956285	0.194	0.46	1.94E-05	16	Hp
1.53E-09	-1.456837705	0.46	0.633	2.01E-05	16	Wfdc21
1.54E-09	0.347090278	0.984	0.856	2.03E-05	16	Rpl35
1.80E-09	0.701296967	0.403	0.204	2.37E-05	16	Fam107b
1.82E-09	-1.553579238	0.371	0.562	2.40E-05	16	Lcn2

2.01E-09	-1.473124244	1	0.999	2.66E-05	16	S100a8
2.32E-09	0.600102646	0.5	0.277	3.06E-05	16	Myl12b
2.65E-09	-0.672992211	0.403	0.65	3.49E-05	16	Prdx5
4.67E-09	0.670613727	0.347	0.158	6.16E-05	16	Ms4a3
4.83E-09	0.331981822	0.992	0.865	6.37E-05	16	Rpl32
5.38E-09	0.692668513	0.427	0.222	7.09E-05	16	Cxcr4
5.96E-09	-0.660598148	0.435	0.669	7.87E-05	16	Arhgdib
6.01E-09	0.466880109	0.782	0.634	7.93E-05	16	Xist
6.08E-09	0.736502361	0.331	0.154	8.01E-05	16	Cdk6
7.87E-09	-1.012124024	0.048	0.288	0.000103793	16	Cd44
1.04E-08	0.291597838	1	0.899	0.000137426	16	Rps28
1.16E-08	0.450100797	0.339	0.15	0.000153239	16	Nkg7
1.59E-08	-0.465999278	0.605	0.784	0.000209689	16	Shfm1
1.59E-08	0.652509069	0.282	0.119	0.000209923	16	Ankrd12
1.87E-08	-1.583453059	0.839	0.85	0.000246184	16	Ngp
4.64E-08	0.334972829	0.903	0.766	0.000611657	16	Rpl21
5.04E-08	0.2989321	0.968	0.895	0.000664829	16	Rps19
6.59E-08	0.618928433	0.306	0.141	0.000869759	16	Mat2a
1.04E-07	0.675399811	0.323	0.157	0.001372794	16	Rgs18
1.18E-07	-1.178409499	0.145	0.364	0.001558359	16	Ifitm6
1.28E-07	-0.821050114	0.169	0.401	0.001692394	16	Dstn
1.52E-07	0.317924261	0.944	0.817	0.002004183	16	Rpl23a
1.77E-07	0.637709161	0.347	0.175	0.002333109	16	Mpc2
1.84E-07	0.326191383	0.887	0.687	0.002421968	16	Rps7
2.36E-07	0.289344602	0.96	0.84	0.00311602	16	Ptma
2.50E-07	-1.501864946	1	0.994	0.003293985	16	S100a9
4.42E-07	0.623780245	0.258	0.116	0.005836727	16	Spn
4.67E-07	0.605227555	0.315	0.156	0.006164287	16	Sdf2l1
5.44E-07	0.384753666	0.815	0.641	0.007175132	16	Rpl18
6.38E-07	-1.061631715	0.403	0.562	0.008421408	16	Ifitm3
6.85E-07	0.527332739	0.403	0.226	0.009035052	16	Spcs2

6.89E-07	0.448766406	0.669	0.463	0.009088331	16	Rpl10-ps3
7.04E-07	0.326738014	0.879	0.682	0.009286834	16	Rpl14
8.16E-07	0.543784374	0.516	0.346	0.010760856	16	Ndufa1
8.23E-07	-0.699345887	0.403	0.585	0.0108529	16	S100a11
1.02E-06	0.514964061	0.298	0.146	0.013467339	16	Tax1bp1
1.09E-06	0.340894566	0.911	0.76	0.014334595	16	Rpl17
1.11E-06	0.329523065	0.919	0.72	0.014577021	16	Rps26
1.13E-06	-0.814643579	0.234	0.442	0.014897209	16	Lgals3
1.13E-06	0.425024626	0.669	0.471	0.014955894	16	Rps26-ps1
1.24E-06	0.651509642	0.266	0.126	0.016293471	16	Rabac1
1.43E-06	0.514811355	0.734	0.557	0.018920805	16	Calm2
1.59E-06	0.431540301	0.282	0.133	0.020919695	16	Vimp
1.80E-06	0.317899214	0.903	0.775	0.023794203	16	Rplp2
2.30E-06	0.510339369	0.629	0.463	0.030326527	16	Cd47
2.49E-06	0.280596345	0.911	0.803	0.032896811	16	Rpl34
2.60E-06	0.365947051	0.823	0.668	0.034289143	16	Rpl23a-ps3
2.62E-06	-0.383586308	0.613	0.778	0.03456261	16	H3f3a
3.22E-06	0.269126837	0.871	0.676	0.042525212	16	Rpl27
3.72E-06	0.331047388	0.855	0.701	0.04903691	16	mt-Cytb
3.97E-06	0.335501237	0.71	0.48	0.052354196	16	Rpl12
4.27E-06	0.550000227	0.339	0.184	0.056312973	16	Hcst
6.09E-06	-0.462442161	0.742	0.822	0.08032407	16	Ftl1
6.28E-06	0.493622845	0.411	0.242	0.082890489	16	Fxyd5
7.48E-06	0.256813903	0.968	0.88	0.098730392	16	Rps14
7.63E-06	0.274830979	0.927	0.806	0.100709053	16	Rpl13a
7.78E-06	0.67185989	0.29	0.155	0.102661735	16	Rpn1
8.26E-06	0.435959395	0.548	0.383	0.108937351	16	Tmem258
1.01E-05	0.480858796	0.331	0.185	0.132714969	16	Mrps33
1.19E-05	0.422074218	0.403	0.244	0.15732902	16	Anp32a
1.21E-05	0.27889446	0.863	0.707	0.159308561	16	Rpl28
1.23E-05	0.292050389	0.831	0.656	0.162755402	16	Eef1a1

1.35E-05	0.272411893	0.871	0.747	0.177429549	16	Rps15a
1.41E-05	0.459201238	0.306	0.164	0.186143831	16	Ssr3
2.00E-05	0.260128609	0.903	0.77	0.263340893	16	Rps3a1
2.10E-05	0.250924324	0.944	0.849	0.276421131	16	Rpl13
2.16E-05	0.325967765	0.766	0.587	0.285011512	16	Wdr89
2.93E-05	-0.699772807	0.145	0.32	0.386398701	16	Lsp1
3.27E-05	0.583683838	0.371	0.233	0.430989167	16	Metap2
3.56E-05	0.441081314	0.282	0.152	0.469108874	16	1110001J03Rik
3.99E-05	0.318252825	0.782	0.605	0.526968742	16	Rps21
4.34E-05	-0.808886561	0.097	0.253	0.572813975	16	Tmcc1
4.69E-05	0.423415681	0.323	0.185	0.619239863	16	Hp1bp3
4.94E-05	-1.075447558	0.21	0.362	0.651013525	16	Ifi27l2a
5.19E-05	0.433769921	0.347	0.205	0.684886745	16	Ppib
5.61E-05	0.268949867	0.911	0.744	0.739663094	16	Rps6
5.91E-05	0.411857856	0.347	0.207	0.779443395	16	Dad1
6.08E-05	0.41168598	0.306	0.171	0.801466086	16	Creg1
6.33E-05	0.425363009	0.266	0.144	0.835203277	16	Morf4l2
7.32E-05	0.325984487	0.637	0.471	0.965456977	16	Rpl27-ps3
7.65E-05	0.488248608	0.427	0.275	1	16	mt-Nd4l
7.98E-05	0.369441609	0.653	0.466	1	16	Rps18-ps3
9.44E-05	-0.458309045	0.177	0.359	1	16	Fam49b
9.80E-05	0.542586631	0.355	0.233	1	16	Dpm3
0.00010764	-0.559750252	0.548	0.644	1	16	Fth1
0.00011702	-0.851892547	0.129	0.277	1	16	Ccr2
0.00011766	-0.669694776	0.137	0.294	1	16	Mgst1
0.00011971	0.307528789	0.75	0.635	1	16	Sec61g
0.00013555	-0.448919612	0.363	0.531	1	16	Coro1a
0.0001636	-0.63587298	0.121	0.269	1	16	Clec4a2
0.00016668	0.250557603	0.79	0.628	1	16	Uba52
0.00018842	0.323649102	0.597	0.423	1	16	Eef2
0.00019125	-0.580772051	0.202	0.367	1	16	Lst1

0.00025087	-0.59367709	0.21	0.356	1	16	Msrb1
0.00029212	0.253132224	0.71	0.551	1	16	Rps2
0.00029528	-0.78732171	0.185	0.32	1	16	Ms4a6c
0.00032667	0.335626186	0.581	0.448	1	16	Alox5ap
0.00037981	0.303404371	0.581	0.431	1	16	Gm9493
0.00040222	0.301671862	0.363	0.218	1	16	Gm2a
0.00040637	-0.597718786	0.153	0.299	1	16	Cd24a
0.00050889	0.421884944	0.274	0.163	1	16	P4hb
0.00052039	-0.430427136	0.218	0.37	1	16	Capza2
0.00054795	-0.656966956	0.129	0.26	1	16	Gm9844
0.00059054	0.304404345	0.444	0.299	1	16	Erh
0.00063533	0.3386674	0.46	0.325	1	16	Cd53
0.00064072	0.436549528	0.29	0.179	1	16	Pdia3
0.00074624	-0.486115689	0.121	0.258	1	16	Flna
0.00075009	-0.41942096	0.355	0.492	1	16	Arpc2
0.00092204	0.342251083	0.266	0.157	1	16	Cd9
0.00092997	0.32122943	0.5	0.371	1	16	Tmem256
0.00096349	0.267740286	0.798	0.69	1	16	Rps13
0.00098938	0.285815863	0.548	0.424	1	16	Hint1
0.00100065	0.277427882	0.597	0.445	1	16	Rpl9-ps6
0.00107916	0.265465519	0.726	0.567	1	16	mt-Nd4
0.00116965	0.371234645	0.282	0.177	1	16	Hypk
0.00131353	0.287083318	0.516	0.37	1	16	Srsf2
0.00134539	0.366503729	0.444	0.324	1	16	Cks2
0.00147323	0.27988226	0.403	0.281	1	16	Gm10269
0.00148127	0.395346349	0.282	0.179	1	16	Myb
0.00154191	0.265869289	0.677	0.571	1	16	Rpl36al
0.00192154	0.253113271	0.629	0.505	1	16	Sec61b
0.00224789	-0.727525793	0.371	0.449	1	16	Pglyrp1
0.00240034	-0.497585834	0.339	0.451	1	16	Psma7
0.00278291	0.381783292	0.306	0.203	1	16	Ralbp1

0.00279029	0.323956625	0.331	0.225	1	16	Nme1	
0.00279234	0.596069055	0.274	0.184	1	16	Lockd	
0.00314146	-0.300257411	0.629	0.687	1	16	Actg1	
0.00345384	-0.532675117	0.234	0.347	1	16	H2afy	
0.00367275	-0.427023365	0.315	0.426	1	16	Tkt	
0.00384608	-0.420258095	0.202	0.321	1	16	Gm10116	
0.00440052	0.316271157	0.444	0.338	1	16	Ifitm2	
0.00479247	0.399055269	0.266	0.181	1	16	Gm6133	
0.00509967	0.352282617	0.355	0.255	1	16	Lsm6	
0.0051579	0.33936611	0.282	0.186	1	16	Fkbp2	
0.00526412	0.258400879	0.258	0.165	1	16	Tmem160	
0.00562756	0.331899817	0.274	0.182	1	16	Erdr1	
0.00667004	0.379574546	0.29	0.197	1	16	AI662270	
0.00689077	-0.418611192	0.21	0.318	1	16	Ptprc	
0.00706644	-0.370668788	0.177	0.289	1	16	Psmb1	
0.00739546	-0.407294226	0.266	0.374	1	16	Arpc5	
0.00749174	-0.280809583	0.645	0.707	1	16	Sub1	
0.00774308	-0.336348006	0.161	0.272	1	16	Dazap2	
0.00781021	0.256681751	0.661	0.577	1	16	Rpl15	
0.00815559	-0.340322956	0.218	0.324	1	16	Npc2	
0.00858797	0.333604714	0.274	0.19	1	16	Ddx3x	
0.00859091	0.289982045	0.597	0.524	1	16	Tma7	
0.00891283	-0.454180794	0.161	0.26	1	16	Gm10282	
0.00962091	0.315469051	0.274	0.182	1	16	Fli1	
0	3.092423691	0.758	0.036	0	17	H2-Eb1	**Dendritic cell**
4.24E-253	3.247386381	0.887	0.071	5.59E-249	17	H2-Aa	
1.58E-222	3.153878119	0.847	0.074	2.09E-218	17	H2-Ab1	
2.78E-170	3.379056513	0.952	0.143	3.67E-166	17	Cd74	
2.15E-125	2.08477316	0.435	0.03	2.83E-121	17	Cd209a	
4.10E-98	1.665582986	0.403	0.033	5.41E-94	17	H2-DMb1	
1.59E-42	1.19371092	0.355	0.057	2.10E-38	17	Pid1	

7.34E-37	1.448759657	0.298	0.048	9.68E-33	17	Tmem176b
8.99E-36	1.391552858	0.935	0.626	1.19E-31	17	Cst3
8.57E-34	1.273218807	0.435	0.106	1.13E-29	17	H2-DMa
1.79E-30	0.892078448	0.952	0.631	2.36E-26	17	Crip1
2.37E-28	1.185309541	0.565	0.199	3.13E-24	17	Ctss
4.02E-28	1.182557211	0.597	0.215	5.30E-24	17	Wfdc17
2.57E-27	1.00796488	0.871	0.553	3.39E-23	17	Ifitm3
3.96E-25	1.122093122	0.565	0.214	5.22E-21	17	Gm2a
3.48E-24	1.038131438	0.637	0.267	4.58E-20	17	Ccr2
3.72E-23	1.038314936	0.726	0.352	4.90E-19	17	Ifi27l2a
1.72E-22	0.967100253	0.661	0.31	2.26E-18	17	Ms4a6c
1.80E-22	-1.71826522	0.145	0.596	2.38E-18	17	Hmgn2
5.06E-20	-0.98242728	0.46	0.786	6.67E-16	17	Hmgb2
7.51E-19	0.62594651	0.935	0.792	9.90E-15	17	Rps11
8.27E-19	0.433990428	1	0.956	1.09E-14	17	Rps27
1.97E-18	1.046201973	0.339	0.103	2.60E-14	17	Marcks
2.47E-18	-2.181430686	0.177	0.566	3.26E-14	17	Lcn2
8.50E-18	0.77632558	0.355	0.107	1.12E-13	17	Rnase6
1.70E-17	0.650402913	0.96	0.735	2.25E-13	17	Tmsb10
4.04E-17	0.348334935	1	0.997	5.33E-13	17	Malat1
4.26E-17	0.467596411	0.976	0.871	5.61E-13	17	Rps9
1.70E-16	-1.592121504	0.202	0.559	2.24E-12	17	Chil3
2.32E-16	0.487509857	0.976	0.842	3.06E-12	17	Rps27rt
2.73E-16	0.487001143	0.992	0.918	3.60E-12	17	Fau
3.31E-16	0.735818641	0.621	0.31	4.36E-12	17	Lsp1
4.37E-16	0.867444753	0.347	0.114	5.77E-12	17	Ly86
1.91E-15	0.685211802	0.774	0.539	2.52E-11	17	Psap
1.17E-14	0.416746541	0.992	0.848	1.54E-10	17	Rpl13
2.35E-14	-1.95267416	1	0.999	3.10E-10	17	S100a8
3.09E-14	-0.913532607	0.323	0.651	4.08E-10	17	Prdx5
7.22E-14	-1.594242665	0.121	0.454	9.53E-10	17	Pglyrp1

1.34E-13	-1.824137815	0.371	0.635	1.77E-09	17	Wfdc21
3.76E-13	-2.055017689	0.685	0.853	4.97E-09	17	Ngp
6.84E-13	0.535383827	0.879	0.727	9.02E-09	17	Cd52
1.51E-12	-1.801733858	0.274	0.546	1.99E-08	17	Ltf
4.62E-12	-1.961576733	0.855	0.932	6.10E-08	17	Camp
6.43E-12	0.518994767	0.903	0.789	8.48E-08	17	Gm9843
1.46E-11	0.293724759	1	0.996	1.92E-07	17	Tmsb4x
1.71E-11	-0.551275728	0.661	0.823	2.26E-07	17	H2afz
3.79E-11	-0.965390072	0.145	0.46	5.00E-07	17	Hp
4.07E-11	0.898424733	0.298	0.117	5.36E-07	17	Mndal
4.39E-11	0.409821579	0.935	0.802	5.79E-07	17	Rpl34
5.36E-11	0.792027292	0.395	0.179	7.07E-07	17	Ccl6
5.75E-11	0.832456951	0.306	0.12	7.58E-07	17	Ifi30
6.14E-11	-1.988317851	0.984	0.995	8.11E-07	17	S100a9
7.22E-11	0.761483946	0.565	0.354	9.53E-07	17	H2-K1
2.09E-10	0.382075404	0.952	0.873	2.76E-06	17	Rpl18a
3.23E-10	-0.821231301	0.121	0.419	4.27E-06	17	Stmn1
6.91E-10	0.647526501	0.726	0.574	9.12E-06	17	H2-D1
8.26E-10	0.448625905	0.855	0.704	1.09E-05	17	Tpt1
1.29E-09	0.922162066	0.258	0.099	1.70E-05	17	Ms4a4c
1.66E-09	0.367579779	0.944	0.777	2.18E-05	17	Rpl26
1.66E-09	0.418954792	0.863	0.692	2.19E-05	17	Rpl10
2.01E-09	0.388695031	0.952	0.818	2.65E-05	17	Ftl1
2.02E-09	0.396941919	0.935	0.759	2.66E-05	17	Rpl17
2.08E-09	0.686864328	0.597	0.408	2.74E-05	17	Atox1
2.41E-09	0.711456717	0.411	0.208	3.18E-05	17	Ahnak
2.68E-09	0.672755171	0.5	0.29	3.53E-05	17	Laptm5
3.23E-09	-0.435713499	0.315	0.62	4.25E-05	17	Ly6c2
9.49E-09	0.421392069	0.831	0.64	0.000125117	17	Rpl18
1.13E-08	-0.863434053	0.137	0.401	0.000149384	17	Dstn
1.17E-08	0.418003476	0.847	0.689	0.000153743	17	Rps13

1.32E-08	1.100066697	0.29	0.13	0.000173581	17	Apoe
1.49E-08	-1.025147352	0.097	0.338	0.000196769	17	2810417H13Rik
1.73E-08	-1.125226335	0.145	0.39	0.000227721	17	Hist1h2ap
3.89E-08	0.445977895	0.831	0.77	0.000513257	17	B2m
4.45E-08	0.291823653	0.944	0.88	0.000586807	17	Rps14
6.24E-08	0.273191374	0.984	0.895	0.000823366	17	Rps19
6.89E-08	-1.000967646	0.048	0.262	0.000908905	17	Gm10282
6.96E-08	-0.728181335	0.081	0.323	0.000917635	17	Top2a
7.49E-08	0.719770871	0.282	0.129	0.000987874	17	Ifi203
7.71E-08	0.624996144	0.427	0.241	0.001016396	17	Fxyd5
1.07E-07	0.311910459	0.927	0.806	0.001412314	17	Rpl13a
1.09E-07	-0.667160837	0.21	0.469	0.001439577	17	Rac2
1.20E-07	-1.406530528	0.065	0.277	0.001585895	17	Prtn3
1.55E-07	0.663269272	0.363	0.192	0.00204891	17	Mpeg1
1.67E-07	-0.714172968	0.137	0.37	0.002197876	17	Gsr
2.88E-07	-0.728029026	0.073	0.295	0.003804166	17	Mgst1
3.68E-07	0.484359859	0.637	0.435	0.004850918	17	Lgals3
4.21E-07	0.525934963	0.411	0.223	0.005557034	17	S100a4
4.23E-07	0.356383969	0.798	0.657	0.005581442	17	Eef1a1
5.16E-07	0.319700385	0.919	0.765	0.006809621	17	Rpl21
5.21E-07	0.253521034	1	0.937	0.00687283	17	Rpl37a
6.31E-07	0.6918054	0.258	0.12	0.008322525	17	Ccl9
6.36E-07	-0.389066408	0.573	0.785	0.008395876	17	Shfm1
6.88E-07	0.264447776	0.992	0.899	0.009079035	17	Rps28
1.02E-06	0.290336849	0.935	0.775	0.01347024	17	Rplp2
1.02E-06	-0.851612364	0.161	0.373	0.013513809	17	Myh9
1.35E-06	-0.6953486	0.113	0.33	0.017747262	17	Cks2
1.44E-06	0.506099913	0.653	0.543	0.019015633	17	Hspa8
1.44E-06	0.336898143	0.919	0.718	0.019030856	17	Rpl6
1.69E-06	0.29395977	0.944	0.781	0.022346039	17	Rps5
1.82E-06	0.595162245	0.484	0.319	0.023972997	17	Npc2

2.25E-06	-0.627431223	0.153	0.38	0.029736077	17	H2afv
2.43E-06	-0.687398341	0.121	0.331	0.032032118	17	Ap3s1
3.61E-06	0.337768954	0.806	0.712	0.047554049	17	Fcer1g
5.14E-06	0.411242654	0.766	0.64	0.067837109	17	Fth1
5.17E-06	-0.492422653	0.492	0.668	0.068250599	17	Arhgdib
5.20E-06	0.647018655	0.266	0.136	0.068568802	17	Samd9l
5.76E-06	-0.795003019	0.234	0.433	0.076005972	17	Anxa1
7.80E-06	0.311595304	0.839	0.719	0.102900064	17	Rpl27a
9.33E-06	0.267600376	0.879	0.745	0.123057043	17	Rps6
9.36E-06	0.542819045	0.29	0.154	0.123518502	17	Ctsh
9.53E-06	0.263843524	0.887	0.707	0.125726663	17	Rplp0
1.14E-05	-0.589757219	0.274	0.482	0.149760883	17	F630028O10Rik
1.17E-05	-1.306653858	0.105	0.273	0.153893736	17	Mpo
1.22E-05	-0.477696919	0.242	0.474	0.160321299	17	Txn1
1.31E-05	0.301991748	0.895	0.806	0.173400619	17	Rps16
1.67E-05	0.69256611	0.274	0.149	0.220032496	17	Dock10
1.86E-05	-0.416891951	0.169	0.383	0.245519826	17	Lbr
1.87E-05	-0.289954371	0.952	0.955	0.247065155	17	mt-Co3
2.57E-05	0.379219722	0.75	0.597	0.338977519	17	Rpl3
3.23E-05	-0.503632214	0.298	0.512	0.426170587	17	Cbx3
3.45E-05	0.505606085	0.5	0.374	0.45517402	17	Btf3
3.87E-05	-0.483396516	0.113	0.296	0.510679218	17	Pkm
4.74E-05	0.686506894	0.419	0.292	0.625554815	17	Sat1
5.39E-05	-0.552814074	0.097	0.262	0.710421958	17	Aprt
5.47E-05	0.587699142	0.266	0.149	0.721742956	17	Trim30a
5.73E-05	0.562235625	0.379	0.248	0.755476222	17	Psmb8
6.75E-05	-0.709108675	0.121	0.282	0.889971225	17	Slpi
7.47E-05	0.430600582	0.46	0.313	0.985755601	17	Ptprc
8.36E-05	-0.524715901	0.105	0.272	1	17	Gpi1
9.25E-05	0.513999942	0.274	0.153	1	17	Arhgap30
0.00010675	0.253070496	0.839	0.672	1	17	Gm2000

0.00011831	0.399841544	0.589	0.466	1	17	Ly6e
0.00012817	-0.413004303	0.556	0.693	1	17	Hmgb1
0.00012884	0.309671765	0.879	0.77	1	17	Rps3a1
0.00012943	0.281072433	0.71	0.576	1	17	Rpl15
0.00014749	0.597672835	0.258	0.147	1	17	Cd48
0.00014923	0.422988887	0.306	0.182	1	17	Mrpl52
0.00016451	0.277312625	0.831	0.759	1	17	Rps3
0.00017493	-0.426247101	0.161	0.335	1	17	Lsm5
0.0001927	0.425476143	0.645	0.554	1	17	Calm1
0.00020501	0.259866903	0.758	0.645	1	17	Rpl8
0.00024886	-0.718696919	0.202	0.363	1	17	Ifitm6
0.0002895	-0.522200243	0.129	0.281	1	17	mt-Nd4l
0.00029772	0.270348345	0.637	0.525	1	17	Coro1a
0.0002994	0.299034716	0.79	0.644	1	17	Gm10263
0.00030219	-0.430786492	0.226	0.395	1	17	Gm8186
0.00034435	0.319698748	0.565	0.432	1	17	Rpl13-ps3
0.00035162	0.492885202	0.46	0.366	1	17	Capza2
0.00035356	0.511374406	0.444	0.338	1	17	Ifitm2
0.00036769	-0.423123135	0.419	0.585	1	17	S100a11
0.00038072	-0.379567422	0.121	0.273	1	17	Aldoa
0.00038425	0.382138107	0.565	0.465	1	17	Rpl10-ps3
0.0003899	0.291703424	0.823	0.744	1	17	Rpl9
0.0004281	0.279143314	0.653	0.53	1	17	S100a6
0.00044863	0.383207091	0.282	0.167	1	17	Pld4
0.00046326	-0.302398764	0.855	0.842	1	17	Ptma
0.00047109	0.392801935	0.589	0.502	1	17	Mbnl1
0.0004737	0.256835817	0.823	0.715	1	17	Rpl11
0.00050483	0.344064315	0.548	0.426	1	17	Prdx1
0.00056241	0.305062245	0.758	0.656	1	17	Rps10
0.00060677	0.545185661	0.258	0.161	1	17	Ctsz
0.0006824	-0.540943741	0.153	0.299	1	17	Cd24a

0.000702	0.420034519	0.371	0.252	1	17	Btg1
0.00082559	0.414747309	0.29	0.18	1	17	Kctd12
0.00086902	-0.429785702	0.218	0.369	1	17	Ran
0.00090531	-0.440203012	0.177	0.326	1	17	Scp2
0.00095606	0.252907378	0.742	0.741	1	17	H3f3b
0.00096592	-0.4071948	0.129	0.266	1	17	Ncf1
0.00099314	0.425876813	0.774	0.682	1	17	Plac8
0.00103166	-0.349323727	0.218	0.376	1	17	Srsf2
0.00108141	-0.665388409	0.306	0.48	1	17	Retnlg
0.00110282	-0.376835715	0.371	0.523	1	17	Snrpg
0.00129204	-0.30739652	0.258	0.427	1	17	Tkt
0.00131265	0.473640335	0.258	0.163	1	17	Sh3bgrl
0.00136541	0.355499613	0.565	0.473	1	17	Rpl27-ps3
0.00139407	-0.3918784	0.145	0.29	1	17	Ndufv3
0.00144999	0.460216423	0.427	0.331	1	17	Notch2
0.00148776	0.44391964	0.492	0.402	1	17	Pfdn5
0.00154484	-0.36742507	0.121	0.259	1	17	Alyref
0.00168126	-0.296180101	0.137	0.278	1	17	Lamtor2
0.00177022	-0.390152746	0.226	0.377	1	17	Tmem256
0.00177907	-0.415251313	0.226	0.37	1	17	Tuba1b
0.00185581	0.5052446	0.347	0.247	1	17	Capzb
0.00187412	-0.432724286	0.315	0.453	1	17	Atpif1
0.00190183	0.363402029	0.524	0.425	1	17	Eef2
0.00208679	-0.354561454	0.145	0.285	1	17	Tra2b
0.00218841	-0.280846103	0.411	0.571	1	17	Hnrnpa2b1
0.00220507	-0.371582698	0.242	0.387	1	17	1810037I17Rik
0.00225357	0.27173411	0.71	0.609	1	17	Gm11808
0.0025691	0.413750388	0.387	0.313	1	17	Eif3k
0.00287116	-0.351221303	0.218	0.356	1	17	Msrb1
0.0029055	0.259314735	0.677	0.574	1	17	Rpl10a
0.00294098	-0.331481866	0.266	0.417	1	17	Gapdh

0.00297917	0.2696864	0.532	0.417	1	17	Hsp90ab1	
0.00314335	-0.304823533	0.185	0.329	1	17	Lamtor4	
0.00326396	-0.334720011	0.194	0.339	1	17	Nucks1	
0.00356837	0.471153251	0.355	0.256	1	17	Gm9844	
0.0036051	-0.283648597	0.185	0.323	1	17	Hnrnpu	
0.00394047	-0.379925615	0.468	0.572	1	17	mt-Nd4	
0.00428916	0.403318498	0.274	0.183	1	17	Samhd1	
0.00432744	0.432071582	0.323	0.234	1	17	Zeb2	
0.00433786	-0.274561187	0.194	0.328	1	17	Hsp90aa1	
0.00451915	0.481167889	0.363	0.282	1	17	Gm10269	
0.00460602	-0.269497072	0.234	0.38	1	17	Eif5a	
0.0055033	-0.355030358	0.169	0.286	1	17	Ywhab	
0.0057311	0.41958511	0.339	0.254	1	17	Vim	
0.00605619	-0.344126333	0.21	0.336	1	17	Irf2bp2	
0.00613788	-0.402099021	0.161	0.277	1	17	Aldh2	
0.00657531	0.404916702	0.395	0.317	1	17	Gm10116	
0.00667447	0.329402097	0.419	0.333	1	17	Pomp	
0.00687825	-0.276912707	0.266	0.411	1	17	Anp32b	
0.00718243	0.372940866	0.371	0.283	1	17	Eef1g	
0.007693	-0.366288313	0.145	0.254	1	17	Ndufb2	
0.00794534	-0.347129369	0.524	0.622	1	17	mt-Nd1	
0.00801706	-0.289239846	0.137	0.253	1	17	Ndufb3	
0.00874004	-0.324046462	0.145	0.255	1	17	Timm13	
0.00919151	0.448462804	0.347	0.277	1	17	Atp6v1g1	
0.00922271	0.37255222	0.411	0.348	1	17	Ndufa6	
2.74E-17	0.485465639	0.366	0.09	3.62E-13	18	Ly6g	**Neutrophil**
1.86E-13	0.554674515	0.828	0.423	2.45E-09	18	Anxa1	
5.76E-13	0.476784983	0.505	0.178	7.60E-09	18	Ccl6	
1.19E-12	0.464794997	0.72	0.31	1.57E-08	18	Ptprc	
3.17E-12	0.442840482	0.892	0.536	4.18E-08	18	Ltf	
5.56E-12	0.564037264	0.645	0.286	7.34E-08	18	Mgst1	

6.12E-12	0.345011339	0.946	0.552	8.07E-08	18	Lcn2
3.02E-11	0.332497905	0.753	0.354	3.98E-07	18	Ifitm6
3.59E-11	0.281214336	0.989	0.625	4.73E-07	18	Wfdc21
4.39E-11	0.460286606	0.914	0.527	5.79E-07	18	S100a6
3.49E-10	0.428058641	0.333	0.112	4.60E-06	18	Mmp9
7.43E-10	0.356515193	0.828	0.442	9.80E-06	18	Pglyrp1
9.88E-10	0.407780573	0.86	0.554	1.30E-05	18	Ifitm3
3.33E-09	0.441593717	0.441	0.177	4.39E-05	18	Ckap4
3.43E-09	0.384250914	0.387	0.143	4.53E-05	18	Itgam
3.53E-09	0.471999739	0.344	0.123	4.65E-05	18	Pirb
5.52E-09	0.385649078	0.516	0.222	7.29E-05	18	S100a4
8.01E-09	0.333821994	1	0.994	0.000105689	18	S100a9
8.73E-09	0.335075096	0.989	0.847	0.000115108	18	Ngp
1.04E-08	0.383569136	0.269	0.087	0.000137518	18	Ssh2
1.08E-08	0.355752177	0.548	0.246	0.000142681	18	Tmcc1
1.39E-08	0.309994421	0.28	0.093	0.000182923	18	Rab8a
1.40E-08	0.510414469	0.28	0.097	0.00018457	18	Lrg1
2.19E-08	0.279250624	0.484	0.206	0.000288857	18	Fn1
2.77E-08	0.424497795	0.301	0.108	0.000364799	18	Dok3
2.85E-08	0.269409727	1	0.904	0.000376399	18	Lyz2
4.67E-08	0.347961807	0.505	0.225	0.000615731	18	Cpne3
4.95E-08	0.328073719	0.892	0.577	0.000652481	18	S100a11
5.67E-08	0.347183459	0.989	0.726	0.000747285	18	Cd52
8.10E-08	0.336667876	0.914	0.661	0.001068909	18	Arhgdib
9.06E-08	0.404502653	0.366	0.148	0.001195196	18	Rhog
9.27E-08	0.500169658	0.516	0.254	0.001222239	18	Cebpb
9.65E-08	0.30331725	0.548	0.252	0.001273056	18	Anxa2
9.81E-08	0.377825814	0.688	0.359	0.001294452	18	Lst1
1.45E-07	0.375607289	0.559	0.276	0.001910855	18	Rap1b
1.47E-07	0.414082631	0.28	0.102	0.001939583	18	Ms4a6b
2.31E-07	0.909174523	0.731	0.473	0.00305275	18	Retnlg

2.80E-07	0.382255408	0.323	0.128	0.003688079	18	Syne1
3.42E-07	0.373407644	0.505	0.241	0.004505024	18	Fxyd5
3.67E-07	0.382034026	0.559	0.275	0.0048375	18	Lcp1
4.24E-07	0.543969298	0.29	0.117	0.005587197	18	Mmp8
4.24E-07	0.385392199	0.398	0.177	0.005596429	18	Gda
4.38E-07	0.302752738	0.258	0.092	0.005775435	18	Megf9
4.89E-07	0.35304306	0.731	0.434	0.006454214	18	Lgals3
5.48E-07	0.394097808	0.796	0.497	0.007222424	18	Cybb
8.32E-07	0.30992209	0.548	0.27	0.010977596	18	Aldh2
1.17E-06	0.290420847	0.366	0.156	0.015488614	18	Cd9
1.78E-06	0.322846257	0.527	0.262	0.023506827	18	Clec4a2
2.07E-06	0.30382677	0.839	0.548	0.027244501	18	Chil3
2.76E-06	0.422985905	0.28	0.114	0.036406124	18	Rnf130
2.80E-06	0.304312621	0.559	0.29	0.036969169	18	Sat1
3.45E-06	0.304288671	0.29	0.12	0.045550339	18	Vcl
3.82E-06	0.292873436	0.548	0.283	0.050341388	18	Ndufv3
3.91E-06	0.389931537	0.473	0.237	0.05154109	18	Slfn2
3.99E-06	0.302792833	0.387	0.177	0.052652764	18	Zyx
4.15E-06	0.323315207	0.258	0.101	0.054803084	18	Gpr141
4.22E-06	0.27569131	0.258	0.1	0.055627185	18	Ltb4r1
5.10E-06	0.277458623	0.688	0.392	0.067300239	18	Dstn
5.58E-06	0.270637262	0.441	0.212	0.07361351	18	Srp14
5.91E-06	0.328166007	0.28	0.116	0.07800311	18	Neat1
7.29E-06	0.399095351	0.28	0.116	0.096189768	18	Ap1s2
7.29E-06	0.289962815	0.452	0.222	0.096200206	18	Ptpn6
8.48E-06	0.291509637	0.269	0.11	0.111853335	18	Il17ra
8.60E-06	0.329838241	0.333	0.15	0.113431283	18	Glrx
8.70E-06	0.315076984	0.269	0.111	0.114809006	18	Pygl
9.58E-06	0.271157862	0.43	0.21	0.126416643	18	Cd177
1.69E-05	0.318968595	0.419	0.211	0.223313008	18	C3
1.71E-05	0.276612041	0.452	0.231	0.225326979	18	Arpc4

1.99E-05	0.276172126	0.366	0.175	0.262388889	18	Mpc2
2.16E-05	0.336403518	0.269	0.117	0.285003053	18	Soat1
2.30E-05	-0.470401539	0.882	0.859	0.303746835	18	mt-Atp6
2.38E-05	0.325690595	0.29	0.132	0.313826325	18	Sephs2
3.08E-05	0.384659768	0.591	0.35	0.406929529	18	Msrb1
3.54E-05	0.293459775	0.495	0.275	0.466803308	18	Iqgap1
7.81E-05	0.264883744	0.312	0.147	1	18	Tpd52
7.84E-05	0.326516579	0.269	0.124	1	18	Ep300
8.66E-05	-0.357983227	1	0.955	1	18	Rpl41
0.00010203	0.267960828	0.409	0.214	1	18	Lims1
0.00011641	0.280709811	0.71	0.451	1	18	Hp
0.00012577	0.350387256	0.452	0.253	1	18	Flna
0.00015266	0.338201279	0.462	0.276	1	18	Slpi
0.00018311	0.272802811	0.258	0.121	1	18	Atp6v1b2
0.00021231	0.37715548	0.29	0.147	1	18	Lta4h
0.00022376	0.393547127	0.355	0.194	1	18	Eno1
0.00022408	0.29191152	0.301	0.153	1	18	Bnip3l
0.00022947	0.273325644	0.355	0.183	1	18	Rbms1
0.00028785	0.255278846	0.398	0.208	1	18	Mcemp1
0.00030044	0.359300519	0.462	0.261	1	18	Ncf1
0.00030489	0.270891831	0.333	0.174	1	18	Far1
0.0003947	0.254009427	0.312	0.158	1	18	Prkcd
0.00044185	0.276313696	0.301	0.156	1	18	Pak2
0.0004479	0.32363152	0.312	0.164	1	18	Napsa
0.00065433	-0.342628586	0.968	0.88	1	18	Rps14
0.00070339	0.267157685	0.312	0.163	1	18	Tecr
0.00071973	0.305262177	0.269	0.138	1	18	Gabarapl2
0.00091499	-0.359263568	0.882	0.806	1	18	Rps16
0.00097123	-0.38934619	0.903	0.835	1	18	Rps18
0.00113743	0.270495701	0.344	0.191	1	18	R3hdm4
0.00117492	-0.378387342	0.849	0.78	1	18	Rpl26

0.00120311	-0.331258099	0.968	0.896	1	18	Rps19	
0.00145586	0.25327785	0.269	0.142	1	18	Gpsm3	
0.00155286	-0.260623885	0.989	0.954	1	18	mt-Co3	
0.00157913	-0.382578099	0.753	0.753	1	18	Gm10076	
0.00158508	-0.391721389	0.903	0.775	1	18	Rpl39	
0.00186947	-0.373758549	0.731	0.722	1	18	Rpl19	
0.00243083	-0.40462414	0.688	0.662	1	18	Rps20	
0.0033296	-0.354277285	0.914	0.807	1	18	Rpl13a	
0.00385862	-0.354414635	0.935	0.834	1	18	Rps8	
0.00453551	-0.457845361	0.72	0.685	1	18	Rpl14	
0.00475953	0.268063121	0.28	0.164	1	18	Ube2l3	
0.00485873	-0.374792662	0.796	0.724	1	18	Rps4x	
0.00494713	-0.260112132	1	0.9	1	18	Rps28	
0.00502949	-0.348231268	0.849	0.784	1	18	Rps5	
0.00542991	-0.335714377	0.978	0.856	1	18	Rpl35	
0.00617994	-0.291863598	0.871	0.759	1	18	Rps3	
0.00635908	-0.383429198	0.796	0.703	1	18	mt-Cytb	
0.00729005	-0.296526913	0.785	0.762	1	18	Rpl17	
0.00763871	-0.374469892	0.72	0.69	1	18	Hmgb1	
0.00784156	-0.32398022	0.957	0.85	1	18	Rpl13	
0	2.503422127	0.753	0.012	0	19	Gypa	**Car enzyme HPC**
0	2.478705322	0.836	0.019	0	19	Hemgn	
0	2.100373061	0.589	0.003	0	19	Slc4a1	
5.41E-276	1.354215017	0.342	0.003	7.14E-272	19	Epb42	
4.44E-265	1.852842999	0.644	0.017	5.86E-261	19	Ctse	
5.39E-259	4.515615041	0.973	0.05	7.11E-255	19	Hbb-bt	
6.08E-245	5.015787377	1	0.058	8.02E-241	19	Hba-a2	
3.37E-197	1.127048264	0.329	0.005	4.45E-193	19	Sox6	
4.51E-197	2.480430106	0.959	0.064	5.95E-193	19	Car2	
4.96E-192	5.187250215	1	0.08	6.55E-188	19	Hba-a1	
5.64E-184	2.023247209	0.726	0.036	7.44E-180	19	Aqp1	

2.44E-181	1.126668941	0.315	0.005	3.22E-177	19	Sptb
6.74E-181	2.097882838	0.74	0.039	8.89E-177	19	Cpox
3.82E-175	5.011964269	1	0.089	5.05E-171	19	Hbb-bs
1.09E-166	1.306444402	0.411	0.011	1.44E-162	19	Nxpe2
4.66E-166	1.193121576	0.315	0.006	6.14E-162	19	Snca
1.27E-165	1.486517594	0.397	0.01	1.68E-161	19	Cldn13
5.28E-155	1.459745024	0.397	0.011	6.96E-151	19	Rhag
2.77E-148	1.640231314	0.562	0.026	3.66E-144	19	Smim1
1.09E-139	1.251753353	0.315	0.008	1.44E-135	19	Rhd
5.61E-129	1.115193773	0.274	0.006	7.39E-125	19	Btnl10
3.99E-128	1.367682317	0.384	0.013	5.27E-124	19	Hebp1
4.70E-127	2.009492315	0.753	0.061	6.20E-123	19	Blvrb
1.92E-118	1.143428011	0.26	0.006	2.54E-114	19	Rec114
7.63E-117	1.000677345	0.274	0.007	1.01E-112	19	Ank1
1.96E-111	1.192561411	0.274	0.007	2.59E-107	19	Spta1
2.13E-110	1.68144783	0.466	0.025	2.81E-106	19	Ubac1
1.81E-103	1.462703183	0.438	0.023	2.39E-99	19	Ermap
6.05E-101	1.114318914	0.288	0.009	7.99E-97	19	Acp5
7.79E-99	1.688005933	0.521	0.035	1.03E-94	19	Mgst3
4.17E-98	1.141716786	0.301	0.011	5.51E-94	19	Tspan33
1.28E-97	1.249789521	0.37	0.017	1.68E-93	19	Abcb10
1.01E-93	1.774378157	0.466	0.03	1.34E-89	19	Fn3krp
3.36E-83	1.735236394	0.521	0.042	4.43E-79	19	Slc25a37
2.86E-80	1.848414356	0.644	0.069	3.78E-76	19	Tfrc
1.35E-74	1.039988204	0.274	0.012	1.78E-70	19	Isg20
2.06E-72	1.205968545	0.356	0.022	2.71E-68	19	Klf1
6.21E-71	1.530787745	0.438	0.035	8.19E-67	19	Hmbs
2.05E-70	1.004714656	0.315	0.018	2.70E-66	19	Gstm5
1.40E-68	1.196231944	0.301	0.016	1.85E-64	19	Fech
3.96E-66	1.294496706	0.479	0.043	5.23E-62	19	Car1
4.73E-65	0.999832873	0.274	0.014	6.24E-61	19	Ptdss2

2.57E-63	1.814481963	0.781	0.139	3.39E-59	19	Prdx2
1.47E-60	1.723349112	0.726	0.12	1.94E-56	19	Glrx5
6.20E-60	1.978858418	0.959	0.289	8.18E-56	19	Cd24a
7.96E-60	1.678380959	0.863	0.179	1.05E-55	19	Tmem14c
4.62E-59	1.333320222	0.411	0.036	6.09E-55	19	Nfia
9.53E-59	0.986063258	0.315	0.021	1.26E-54	19	Zfpm1
3.65E-53	1.069737864	0.329	0.026	4.81E-49	19	Abcb4
2.77E-49	1.092909375	0.288	0.021	3.65E-45	19	Tfdp2
9.66E-47	1.267018172	0.589	0.094	1.27E-42	19	Bola3
1.02E-46	1.382539314	0.63	0.107	1.35E-42	19	Uhrf1
4.50E-46	0.921903072	0.26	0.019	5.94E-42	19	Gata1
4.23E-45	1.503296722	0.973	0.361	5.58E-41	19	Dek
1.83E-44	1.105444728	0.397	0.044	2.41E-40	19	Clcn3
5.23E-43	1.401514766	0.438	0.058	6.90E-39	19	Odc1
1.54E-42	0.968321348	0.301	0.027	2.03E-38	19	Alad
6.38E-42	-1.885970658	0.932	0.997	8.42E-38	19	Tmsb4x
1.31E-40	1.224747235	0.384	0.046	1.73E-36	19	Minpp1
5.68E-40	1.31454953	0.479	0.074	7.49E-36	19	Pabpc4
3.36E-39	0.88499824	0.274	0.024	4.43E-35	19	Urod
3.74E-39	1.442632333	0.959	0.445	4.93E-35	19	Atpif1
1.76E-36	0.948377719	0.342	0.04	2.33E-32	19	Gclc
6.21E-36	1.025116604	0.356	0.044	8.19E-32	19	Josd2
9.14E-36	1.427714472	0.781	0.23	1.21E-31	19	Metap2
1.22E-35	1.264891895	0.986	0.639	1.61E-31	19	Fth1
7.15E-35	1.459276142	0.712	0.186	9.44E-31	19	Rrm2
7.57E-34	1.009582425	0.26	0.026	9.99E-30	19	Stard10
1.09E-33	0.92221663	0.26	0.026	1.44E-29	19	Slc16a1
2.70E-33	0.942155924	0.288	0.031	3.56E-29	19	Hagh
6.72E-33	0.969253005	0.384	0.053	8.87E-29	19	2-Mar
7.83E-32	1.142791796	0.342	0.046	1.03E-27	19	Slc43a3
1.41E-31	1.1751458	0.438	0.075	1.86E-27	19	Hmgb3

2.24E-31	1.206083625	0.575	0.122	2.96E-27	19	Picalm
5.05E-31	1.087595419	0.411	0.066	6.66E-27	19	Acp1
8.75E-30	1.163056925	0.658	0.171	1.15E-25	19	Slbp
2.17E-29	1.090697533	0.493	0.096	2.86E-25	19	Tnfaip2
3.06E-28	1.138576963	0.63	0.158	4.04E-24	19	Lig1
2.90E-27	0.737801893	1	0.955	3.82E-23	19	Rpl41
3.85E-27	0.856505737	0.288	0.037	5.08E-23	19	Carhsp1
7.76E-27	1.105673881	0.342	0.053	1.02E-22	19	Wdhd1
8.31E-27	1.161479037	0.644	0.178	1.10E-22	19	Bsg
1.41E-25	0.829968073	0.288	0.039	1.85E-21	19	Yod1
1.68E-25	0.949346586	0.356	0.059	2.21E-21	19	Ncapg2
2.09E-25	1.029103005	0.384	0.069	2.76E-21	19	Fam117a
3.91E-25	1.032237775	0.521	0.122	5.16E-21	19	Dck
6.96E-25	1.026614125	0.849	0.312	9.19E-21	19	Top2a
8.22E-25	0.928540578	0.288	0.041	1.08E-20	19	Clic4
2.74E-24	1.108519127	0.438	0.092	3.61E-20	19	Hist1h1e
3.87E-24	0.985556657	0.37	0.067	5.10E-20	19	Prdx3
9.52E-24	0.865375719	0.397	0.075	1.26E-19	19	Svbp
2.62E-23	0.968848673	0.425	0.088	3.45E-19	19	Ccne2
8.25E-23	0.930750005	0.329	0.057	1.09E-18	19	Prkar2b
1.20E-22	0.973970046	0.685	0.214	1.59E-18	19	Ccnd3
1.58E-22	0.973584863	0.342	0.061	2.09E-18	19	Rad23a
1.91E-22	-2.956661764	0.274	0.745	2.51E-18	19	Tmsb10
3.08E-22	0.765033893	0.986	0.751	4.06E-18	19	Gm10076
4.30E-22	-2.002846205	0.781	0.907	5.67E-18	19	Lyz2
1.93E-21	0.811238906	0.315	0.054	2.55E-17	19	Myef2
3.47E-21	1.082076437	0.644	0.212	4.57E-17	19	Mki67
6.91E-21	0.794249544	0.301	0.051	9.11E-17	19	Dtl
9.12E-21	-1.877541678	0.233	0.736	1.20E-16	19	Cd52
1.32E-20	1.107976715	0.808	0.38	1.75E-16	19	Hist1h2ap
1.65E-20	1.107852004	0.575	0.178	2.17E-16	19	Pcna

2.01E-20	0.838351491	0.26	0.04	2.65E-16	19	Scoc
5.80E-20	0.935171947	0.425	0.101	7.65E-16	19	Mcm7
6.15E-20	0.845847166	0.945	0.55	8.12E-16	19	Rps2
1.07E-19	0.989344244	0.589	0.188	1.41E-15	19	Dut
1.33E-19	0.939955257	0.63	0.21	1.75E-15	19	Pdap1
1.93E-19	0.766915422	0.288	0.05	2.55E-15	19	Slc25a51
3.12E-19	0.922815548	0.384	0.086	4.12E-15	19	Prc1
4.27E-19	-1.82693931	0.192	0.69	5.64E-15	19	Cyba
5.72E-19	-1.447085218	0.479	0.819	7.55E-15	19	Tyrobp
1.14E-18	0.795496263	0.397	0.09	1.50E-14	19	Herc1
1.25E-18	0.825566367	0.315	0.061	1.65E-14	19	Ssbp3
1.74E-18	0.847710147	0.342	0.073	2.30E-14	19	Gclm
2.30E-18	1.134217893	0.466	0.128	3.03E-14	19	Hist1h2ae
2.48E-18	0.954461298	0.603	0.199	3.27E-14	19	Supt16
3.89E-18	0.933958766	0.822	0.403	5.13E-14	19	Anp32b
5.80E-18	-1.554990996	0.247	0.698	7.66E-14	19	Sh3bgrl3
6.38E-18	0.919340031	0.644	0.22	8.41E-14	19	Rpgrip1
8.60E-18	0.810869277	0.301	0.058	1.13E-13	19	Fam132a
1.07E-17	0.739099642	0.288	0.053	1.41E-13	19	Grina
1.51E-17	0.746588733	0.384	0.087	2.00E-13	19	Nudt4
1.57E-17	-1.590772647	0.288	0.719	2.07E-13	19	Fcer1g
2.87E-17	1.11269561	0.548	0.181	3.79E-13	19	Lockd
3.07E-17	0.728556199	0.973	0.72	4.05E-13	19	Rplp1
3.39E-17	0.688622397	0.959	0.721	4.47E-13	19	Rps26
6.03E-17	0.90779958	0.411	0.105	7.95E-13	19	Gps2
1.25E-16	0.682675789	0.356	0.08	1.65E-12	19	Ago2
3.64E-16	0.865277506	0.315	0.069	4.80E-12	19	Eid1
4.05E-16	0.935977836	0.521	0.161	5.34E-12	19	Hdgf
4.27E-16	0.747021859	0.959	0.708	5.64E-12	19	Rplp0
6.70E-16	-1.430254673	0.274	0.691	8.84E-12	19	Actg1
8.01E-16	0.837009867	0.384	0.099	1.06E-11	19	Atad2

8.53E-16	0.867592038	0.877	0.504	1.13E-11	19	Cbx3
1.66E-15	0.745845966	0.356	0.087	2.19E-11	19	Mrps36
1.98E-15	0.806437012	0.26	0.051	2.61E-11	19	Ap1b1
2.13E-15	0.793683809	0.74	0.322	2.81E-11	19	Cks2
2.39E-15	0.74202607	0.89	0.47	3.15E-11	19	Rps26-ps1
6.34E-15	0.815523885	0.795	0.381	8.36E-11	19	Eif2s2
1.66E-14	-1.757848437	0.151	0.587	2.19E-10	19	S100a11
2.28E-14	-1.717802868	0.219	0.619	3.00E-10	19	Ly6c2
2.68E-14	0.896781419	0.26	0.055	3.54E-10	19	Mcm2
4.23E-14	1.010875734	0.438	0.138	5.58E-10	19	Hist1h1b
5.43E-14	0.556310028	0.973	0.834	7.17E-10	19	Rps8
5.86E-14	0.450999394	0.986	0.954	7.73E-10	19	mt-Co3
7.49E-14	-1.339092759	0.301	0.669	9.89E-10	19	Arhgdib
1.03E-13	0.684433681	0.37	0.099	1.36E-09	19	Klf3
1.81E-13	0.805340956	0.507	0.179	2.39E-09	19	Tubb4b
2.12E-13	0.830453798	0.507	0.176	2.79E-09	19	Dnmt1
2.17E-13	0.619243545	0.411	0.117	2.86E-09	19	Zbtb7a
2.27E-13	0.748082483	0.795	0.436	3.00E-09	19	Gm10073
2.38E-13	0.724753383	0.274	0.062	3.13E-09	19	Mzt1
2.77E-13	0.773425042	0.822	0.465	3.65E-09	19	Gm8730
5.52E-13	0.841299085	0.534	0.203	7.28E-09	19	Birc5
7.36E-13	-1.593075949	0.11	0.532	9.71E-09	19	Coro1a
7.36E-13	0.758151968	0.37	0.106	9.71E-09	19	Fundc2
8.25E-13	-1.484717854	0.425	0.687	1.09E-08	19	Plac8
1.01E-12	0.636484732	0.918	0.656	1.33E-08	19	Rps12
1.28E-12	1.006099409	0.452	0.158	1.68E-08	19	Nusap1
1.53E-12	0.801882333	0.562	0.225	2.01E-08	19	Smc2
1.97E-12	0.624969901	0.342	0.093	2.60E-08	19	Mcm3
2.20E-12	0.862761718	0.37	0.111	2.90E-08	19	Khsrp
2.22E-12	0.742494544	0.795	0.453	2.93E-08	19	Rps12-ps3
3.47E-12	0.485553042	1	0.856	4.58E-08	19	Rpl35

4.43E-12	-1.503906406	0.288	0.636	5.84E-08	19	Cst3
5.73E-12	-0.755066623	0.74	0.837	7.55E-08	19	Pfn1
6.12E-12	0.673941843	0.479	0.169	8.07E-08	19	Srrm1
6.29E-12	0.666820935	0.274	0.067	8.30E-08	19	Zcchc6
6.99E-12	-1.78040186	0.123	0.506	9.22E-08	19	Cybb
9.12E-12	0.630148496	0.315	0.083	1.20E-07	19	Ehbp1l1
1.19E-11	0.61146017	0.288	0.073	1.56E-07	19	Asxl2
1.40E-11	0.727180238	0.384	0.123	1.84E-07	19	Cbx5
1.93E-11	0.677668245	0.37	0.114	2.55E-07	19	Ybx3
3.59E-11	0.563114542	0.315	0.087	4.73E-07	19	Pkig
4.43E-11	-1.002540998	1	0.999	5.84E-07	19	Actb
4.54E-11	-0.999103132	0.507	0.711	5.98E-07	19	Myl6
4.82E-11	0.730452809	0.315	0.091	6.36E-07	19	Tmem245
5.32E-11	0.641768836	0.329	0.095	7.02E-07	19	Mcm6
6.19E-11	-1.57431309	0.068	0.449	8.16E-07	19	Gmfg
8.04E-11	0.775338996	0.329	0.098	1.06E-06	19	Cyb5a
8.06E-11	0.718763069	0.26	0.067	1.06E-06	19	Hist1h4i
8.13E-11	0.608729446	0.37	0.115	1.07E-06	19	Ap2a2
8.40E-11	-0.835166371	0.63	0.794	1.11E-06	19	Ppia
8.42E-11	0.735594028	0.384	0.132	1.11E-06	19	Ccdc34
9.07E-11	0.711493905	0.397	0.132	1.20E-06	19	Mier1
1.28E-10	0.607500909	0.37	0.118	1.69E-06	19	Gtf2a2
1.36E-10	0.663407515	0.644	0.307	1.80E-06	19	Arf5
1.54E-10	0.707966487	0.274	0.074	2.03E-06	19	Ube2e3
1.57E-10	0.575740545	0.89	0.645	2.07E-06	19	Rpl8
1.70E-10	0.529809978	0.89	0.516	2.24E-06	19	Snrpg
1.75E-10	0.464593908	1	0.849	2.30E-06	19	Rpl13
1.89E-10	0.742920838	0.37	0.121	2.50E-06	19	Celf1
2.30E-10	-1.18356643	0.411	0.648	3.04E-06	19	Prdx5
2.40E-10	0.537263238	0.849	0.48	3.17E-06	19	Rpl12
2.52E-10	-1.378007869	0.137	0.494	3.32E-06	19	Arpc2

3.07E-10	0.459836579	0.959	0.774	4.05E-06	19	Rpl39
3.08E-10	0.579767245	0.877	0.605	4.06E-06	19	Rps21
3.18E-10	0.49499388	0.959	0.683	4.19E-06	19	Rpl14
3.33E-10	-1.001427513	0.315	0.653	4.39E-06	19	Itm2b
3.37E-10	0.576661498	0.329	0.099	4.44E-06	19	Galnt1
3.72E-10	0.658740548	0.466	0.174	4.91E-06	19	Srsf10
3.83E-10	0.445938288	1	0.834	5.05E-06	19	Rps18
3.89E-10	0.422666642	0.986	0.818	5.13E-06	19	H2afz
3.96E-10	0.503497366	0.945	0.742	5.22E-06	19	Gpx1
4.03E-10	-1.763353666	0.219	0.536	5.32E-06	19	S100a6
4.55E-10	0.700810539	0.342	0.109	6.00E-06	19	Zc3hav1
4.63E-10	0.70112577	0.356	0.119	6.11E-06	19	Wbp5
4.74E-10	0.984078257	0.342	0.117	6.26E-06	19	Wapl
5.74E-10	-1.453388264	0.096	0.448	7.57E-06	19	Arpc1b
5.84E-10	-1.43344002	0.123	0.482	7.71E-06	19	F630028O10Rik
6.99E-10	0.4684797	0.945	0.719	9.22E-06	19	Rpl6
1.17E-09	0.565238063	0.411	0.146	1.54E-05	19	Mrpl14
1.23E-09	0.563901458	0.452	0.17	1.62E-05	19	Cenpa
1.25E-09	0.624787931	0.342	0.112	1.65E-05	19	1700020I14Rik
1.45E-09	0.623807279	0.26	0.073	1.91E-05	19	Ppp2r4
1.45E-09	0.553271418	0.397	0.139	1.92E-05	19	Nol7
1.48E-09	-1.570915072	0.055	0.4	1.96E-05	19	Dstn
1.50E-09	-2.090352144	1	0.999	1.98E-05	19	S100a8
1.76E-09	0.508075612	0.863	0.493	2.32E-05	19	Tubb5
1.88E-09	0.599292922	0.301	0.092	2.48E-05	19	Ccna2
1.92E-09	0.673466564	0.384	0.137	2.53E-05	19	Bcas2
1.92E-09	-1.395434314	0.11	0.458	2.53E-05	19	Hp
1.98E-09	0.467071523	0.918	0.739	2.61E-05	19	Rpl23
2.64E-09	-1.617854754	0.11	0.442	3.49E-05	19	Lgals3
2.68E-09	-2.080142974	0.411	0.633	3.54E-05	19	Wfdc21
2.78E-09	0.640495746	0.521	0.226	3.66E-05	19	Csnk1a1

2.98E-09	-1.654580879	0.164	0.481	3.94E-05	19	Lgals1
2.99E-09	0.583479742	0.315	0.1	3.95E-05	19	Suz12
3.05E-09	0.648179455	0.384	0.141	4.03E-05	19	Lmo2
3.13E-09	0.52460076	0.904	0.634	4.12E-05	19	Sec61g
3.50E-09	0.552342495	0.562	0.253	4.62E-05	19	Alyref
3.65E-09	0.598910533	0.795	0.511	4.81E-05	19	Cox6a1
3.66E-09	0.638764333	0.301	0.094	4.83E-05	19	E2f8
4.31E-09	-0.735374889	0.671	0.782	5.69E-05	19	Shfm1
5.37E-09	-1.858094181	0.137	0.451	7.08E-05	19	Pglyrp1
5.44E-09	0.594344936	0.726	0.393	7.18E-05	19	mt-Atp8
5.78E-09	0.639065242	0.274	0.084	7.62E-05	19	Incenp
6.06E-09	0.652111571	0.658	0.356	8.00E-05	19	Gpx4
6.19E-09	0.532306311	0.89	0.681	8.17E-05	19	Rpsa
7.84E-09	0.531473982	0.37	0.13	0.000103395	19	Alkbh5
7.89E-09	0.60265519	0.329	0.112	0.000104037	19	Smc1a
8.59E-09	0.521297023	0.753	0.388	0.000113318	19	Gm8186
8.92E-09	0.587127372	0.493	0.205	0.000117645	19	Hnrnpd
9.41E-09	0.469129398	0.863	0.587	0.000124169	19	Wdr89
1.03E-08	-1.690405748	0.438	0.639	0.00013589	19	Crip1
1.18E-08	-1.688803299	0.315	0.555	0.000155046	19	Chil3
1.26E-08	0.785813455	0.329	0.116	0.000166775	19	Tipin
1.64E-08	0.412981301	0.932	0.694	0.000216179	19	Oaz1
1.68E-08	0.607900473	0.63	0.311	0.000221989	19	Atp5g1
1.81E-08	0.574704564	0.274	0.085	0.000238795	19	Psmd11
1.89E-08	0.683874782	0.301	0.102	0.000249177	19	Ppm1g
1.90E-08	-1.50621726	0.274	0.562	0.000250835	19	Ifitm3
2.00E-08	0.643167879	0.397	0.157	0.000263177	19	Rpa3
2.21E-08	0.458125949	0.918	0.688	0.00029186	19	Hmgb1
2.27E-08	0.378815627	0.959	0.807	0.000299173	19	Rpl13a
3.27E-08	0.645936391	0.26	0.081	0.000431967	19	Pkn2
3.56E-08	0.655667817	0.425	0.175	0.00047018	19	Smarca5

3.58E-08	0.504821374	0.452	0.185	0.000471961	19	Eif3j1
3.67E-08	0.374252134	0.945	0.858	0.000484265	19	mt-Atp6
3.76E-08	0.681252599	0.288	0.098	0.000495519	19	Wdr76
5.33E-08	0.409167142	0.918	0.641	0.000703153	19	Rpl36a
6.17E-08	0.511034173	0.288	0.096	0.000814407	19	Smap1
6.65E-08	0.619440478	0.452	0.202	0.000877024	19	Ewsr1
8.63E-08	0.5307524	0.74	0.429	0.00113777	19	Cox17
9.45E-08	0.472764468	0.836	0.522	0.001246288	19	Rpl22
9.69E-08	0.556289357	0.589	0.298	0.001278826	19	Sfpq
1.17E-07	0.562849243	0.274	0.091	0.001536942	19	Tnrc6a
1.20E-07	-2.146179029	1	0.994	0.001588291	19	S100a9
1.23E-07	0.462656498	0.781	0.444	0.001625856	19	Rpl9-ps6
1.24E-07	0.558734582	0.685	0.424	0.001641258	19	Serbp1
1.26E-07	0.380257726	0.918	0.779	0.001668352	19	Rpl26
1.32E-07	0.325838964	0.986	0.777	0.001747403	19	Hmgb2
1.84E-07	0.583393705	0.26	0.087	0.002425823	19	Plk4
1.91E-07	0.534376505	0.671	0.376	0.002521334	19	Lbr
1.96E-07	-1.279293387	0.315	0.546	0.00258817	19	Psap
2.06E-07	0.463171664	0.26	0.084	0.002722675	19	Itgb1
2.07E-07	-1.937718917	0.342	0.544	0.002728298	19	Ltf
2.08E-07	0.685953785	0.301	0.11	0.002748036	19	Cenpf
2.35E-07	0.344884355	0.932	0.806	0.003098839	19	Rps16
2.60E-07	-1.917444373	0.356	0.56	0.003427738	19	Lcn2
2.64E-07	0.443494062	0.26	0.085	0.003486706	19	Aplp2
2.95E-07	-1.001769382	0.342	0.58	0.003885048	19	H2-D1
3.13E-07	0.33495131	0.918	0.747	0.004134887	19	Rps15a
3.19E-07	0.680096323	0.616	0.37	0.004205761	19	Srsf2
3.40E-07	0.516442775	0.452	0.197	0.004481468	19	Dnajc8
3.51E-07	0.491759707	0.836	0.567	0.004635772	19	mt-Nd4
5.68E-07	-1.224162403	0.233	0.471	0.007495681	19	Ly6e
5.96E-07	-1.290459658	0.027	0.297	0.007855872	19	Laptm5

6.35E-07	0.619889008	0.26	0.092	0.008375379	19	Ccnb2
7.04E-07	0.357729593	0.932	0.776	0.009286368	19	Rplp2
7.20E-07	0.52273627	0.603	0.33	0.009497859	19	2810417H13Rik
8.33E-07	0.55590412	0.849	0.581	0.010987668	19	Eif1
9.28E-07	0.583348962	0.288	0.105	0.012234874	19	Golim4
9.37E-07	0.361820839	0.932	0.743	0.012359643	19	Rpl9
9.46E-07	-1.141614648	0.096	0.369	0.012478451	19	Emp3
9.54E-07	0.392513326	0.877	0.597	0.012579684	19	Rpl3
1.02E-06	-1.082215632	0.205	0.454	0.013412302	19	Alox5ap
1.15E-06	0.403064455	0.904	0.723	0.015231381	19	Rps4x
1.16E-06	0.536927957	0.288	0.107	0.015303301	19	Psip1
1.23E-06	0.50823876	0.301	0.115	0.016191793	19	Fam120a
1.25E-06	0.381497987	0.342	0.134	0.016541968	19	Ctbp1
1.49E-06	0.477805099	0.534	0.265	0.019623512	19	Ndufb9
1.57E-06	0.541557349	0.274	0.101	0.020733928	19	2700029M09Rik
1.63E-06	0.58903417	0.521	0.278	0.02153205	19	Hnrnpab
1.65E-06	0.519211293	0.534	0.281	0.021730459	19	Luc7l2
1.71E-06	-1.445871215	0.014	0.26	0.022573344	19	Gm9844
1.81E-06	0.536669545	0.301	0.119	0.023923481	19	Cenpw
1.88E-06	-1.205456555	0.123	0.368	0.024790125	19	Gsr
1.89E-06	-1.031382595	0.137	0.401	0.024952276	19	Actr3
2.07E-06	-1.27555859	0.192	0.432	0.027361389	19	Anxa1
2.13E-06	-1.2642343	0.014	0.259	0.02803116	19	Vim
2.21E-06	-0.697912029	0.603	0.707	0.029192322	19	Sub1
2.28E-06	-2.007177569	0.973	0.931	0.030011648	19	Camp
2.39E-06	0.389044065	0.849	0.655	0.031504951	19	Rps10
2.42E-06	0.372890665	0.89	0.673	0.031946955	19	Gm2000
2.47E-06	0.511603179	0.534	0.282	0.032622323	19	Tmpo
2.52E-06	0.495878209	0.808	0.618	0.0332679	19	mt-Nd1
2.68E-06	-2.13839434	0.849	0.849	0.035320598	19	Ngp
2.71E-06	-1.555197823	0.123	0.363	0.035762262	19	Ifitm6

3.12E-06	0.452122141	0.384	0.166	0.041168089	19	Srsf1
3.23E-06	0.467102769	0.74	0.479	0.042646713	19	Uqcrh
3.27E-06	-1.375004926	0.027	0.267	0.043141571	19	S100a10
3.72E-06	-1.198063683	0.123	0.367	0.049086204	19	Lst1
3.85E-06	0.38106663	0.493	0.236	0.050795281	19	Cks1b
3.89E-06	-1.128606075	0.123	0.366	0.051343057	19	Serp1
4.38E-06	0.609783535	0.301	0.125	0.057818147	19	Dnajc9
4.47E-06	0.32540203	0.918	0.707	0.05890266	19	Rpl28
4.57E-06	-1.200032365	0.082	0.325	0.060286978	19	Npc2
4.69E-06	0.348841023	0.918	0.743	0.061846	19	Rpl24
4.71E-06	0.336225722	0.89	0.767	0.062185742	19	Rpl21
4.93E-06	0.312729828	0.973	0.809	0.064966737	19	Rps23
4.93E-06	0.324563185	0.932	0.773	0.06496746	19	H3f3a
5.08E-06	0.36684896	0.658	0.364	0.067058901	19	Tuba1b
5.22E-06	0.495597629	0.26	0.097	0.068840137	19	N4bp2l2
5.38E-06	-1.421552398	0.041	0.277	0.07095041	19	Ccr2
5.98E-06	0.343642096	0.849	0.72	0.078849178	19	Rpl27a
6.42E-06	0.457469595	0.726	0.467	0.084672968	19	Rpl29
6.51E-06	0.372199029	0.589	0.315	0.085817479	19	Gm10116
6.95E-06	0.423872315	0.329	0.138	0.091677988	19	Snhg9
7.03E-06	0.47371306	0.562	0.314	0.092788849	19	Snrpe
7.55E-06	0.416895266	0.74	0.474	0.099610035	19	Rps27l
7.68E-06	0.691004301	0.301	0.128	0.101289331	19	Usp1
7.73E-06	-0.997550424	0.274	0.466	0.10201356	19	Rac2
8.42E-06	0.410791276	0.644	0.371	0.111026487	19	Tmem256
8.53E-06	-0.840206743	0.151	0.395	0.112528645	19	Ywhaz
9.45E-06	-1.130695989	0.068	0.295	0.124617462	19	Pkm
9.45E-06	0.476182842	0.589	0.335	0.124644059	19	Srsf3
9.62E-06	0.435958294	0.534	0.28	0.126921758	19	Tra2b
1.01E-05	-0.622542646	0.671	0.772	0.132853276	19	B2m
1.03E-05	-0.922476056	0.205	0.434	0.135392564	19	Tspo

1.07E-05	-0.987040579	0.123	0.358	0.141644806	19	Fam49b
1.11E-05	0.394838681	0.644	0.37	0.146911806	19	Srrm2
1.14E-05	0.50498135	0.329	0.144	0.15057109	19	Rbm15
1.15E-05	0.298332748	0.986	0.901	0.152089049	19	Rpl37
1.23E-05	0.419459437	0.301	0.124	0.162007457	19	Bclaf1
1.30E-05	-1.233656303	0.041	0.259	0.171857149	19	Anxa2
1.31E-05	0.543075715	0.452	0.232	0.172328301	19	Usp50
1.35E-05	0.372471474	0.356	0.157	0.1787049	19	Tardbp
1.37E-05	-1.140506003	0.123	0.343	0.181167173	19	Ifitm2
1.48E-05	0.527009316	0.479	0.251	0.195007982	19	Eif3a
1.49E-05	0.49344147	0.274	0.111	0.195927063	19	Gadd45gip1
1.52E-05	0.422166586	0.411	0.194	0.19996269	19	Smc4
1.53E-05	0.472175019	0.548	0.302	0.201269851	19	Hnrnpm
1.58E-05	0.422766269	0.411	0.197	0.208388795	19	Gm10020
1.60E-05	0.565765786	0.26	0.106	0.211291234	19	Adam10
1.67E-05	0.519391224	0.315	0.139	0.220193699	19	Dtymk
1.72E-05	0.527036115	0.26	0.103	0.227506608	19	Cmc2
1.73E-05	-0.812025325	0.164	0.406	0.228739744	19	Dbi
1.83E-05	-1.076946409	0.11	0.331	0.241675564	19	Cd53
1.90E-05	0.33668188	0.932	0.745	0.250973101	19	Rps6
1.93E-05	0.508273376	0.288	0.118	0.254725306	19	Cdc42ep3
1.99E-05	-0.810175041	0.274	0.48	0.262376745	19	Gng5
2.01E-05	0.518904656	0.425	0.212	0.26514593	19	Rad21
2.06E-05	0.367034711	0.37	0.167	0.271098238	19	Prpf4b
2.19E-05	-1.140033681	0.137	0.348	0.288722954	19	H2afy
2.38E-05	0.525599541	0.466	0.246	0.314119181	19	Rbm25
2.38E-05	0.406135909	0.384	0.175	0.314194877	19	Cltc
2.39E-05	-1.069583514	0.096	0.319	0.314939135	19	Lsp1
2.39E-05	-1.029785051	0.068	0.286	0.314983142	19	Cmtm7
2.43E-05	-1.120372246	0.055	0.268	0.32009172	19	Clec4a2
2.60E-05	0.67915979	0.329	0.153	0.342449678	19	Ube2c

2.74E-05	0.433835646	0.37	0.174	0.361483654	19	Siva1
2.81E-05	-1.041320564	0.055	0.269	0.370884894	19	Cotl1
2.90E-05	0.256879476	0.932	0.706	0.383011974	19	Rpl31
2.91E-05	0.495424694	0.288	0.123	0.384212594	19	Anapc5
2.92E-05	0.372287402	0.575	0.318	0.385172041	19	Hnrnpu
3.07E-05	-1.082622425	0.055	0.266	0.405236676	19	Ncf1
3.23E-05	-0.710251713	0.37	0.557	0.426177548	19	Calm1
3.32E-05	0.428410313	0.301	0.131	0.437616762	19	Ubap2l
3.51E-05	-1.030814679	0.082	0.289	0.46359877	19	Tagln2
3.56E-05	0.538414377	0.274	0.119	0.470051458	19	Ssrp1
3.61E-05	0.592468071	0.288	0.127	0.475593786	19	Zranb2
3.65E-05	-1.103834784	0.068	0.281	0.481270306	19	Slpi
3.86E-05	0.392584262	0.671	0.424	0.509722353	19	Eef2
3.94E-05	-1.08952268	0.082	0.293	0.520367634	19	Mgst1
4.67E-05	-0.919313228	0.11	0.325	0.616414578	19	Celf2
4.72E-05	-1.042509685	0.11	0.318	0.622521849	19	Ptprc
4.96E-05	0.363426468	0.699	0.448	0.654390481	19	Uqcr11
5.03E-05	-0.947510382	0.123	0.335	0.663358231	19	Clec12a
5.07E-05	-1.146009366	0.11	0.32	0.669181531	19	Ms4a6c
5.27E-05	0.508004335	0.301	0.137	0.695634656	19	Gmnn
5.36E-05	0.373410016	0.548	0.299	0.706475121	19	Erh
5.76E-05	-0.907661359	0.137	0.351	0.759583506	19	Ptpn18
6.23E-05	0.267674534	0.918	0.759	0.822423039	19	Rps3
6.25E-05	0.335680859	0.795	0.502	0.825012579	19	Rpl22l1
6.29E-05	0.444387724	0.329	0.155	0.829961094	19	Lmnb1
6.70E-05	0.430095316	0.479	0.262	0.884058383	19	Ndufs6
6.74E-05	-0.968144926	0.068	0.271	0.889356357	19	Gpi1
7.13E-05	0.447854853	0.288	0.125	0.940854779	19	Cnot6
7.45E-05	-1.098228475	0.137	0.335	0.983148212	19	Notch2
7.46E-05	0.275626329	0.918	0.719	0.984308127	19	Rps17
7.63E-05	-0.996796627	0.082	0.283	1	19	Rap1b

7.99E-05	-1.083880025	0.055	0.252	1	19	Tmcc1
8.06E-05	0.4335133	0.288	0.128	1	19	Eif4e
8.74E-05	-0.986509429	0.082	0.28	1	19	Iqgap1
9.24E-05	0.478708718	0.274	0.12	1	19	Abcf1
9.39E-05	-0.96481648	0.11	0.303	1	19	Tpm3
1.00E-04	0.269482171	0.301	0.132	1	19	Smchd1
0.00010496	0.324285116	0.795	0.576	1	19	Rpl15
0.00010971	-1.060833354	0.068	0.259	1	19	Cebpb
0.00011023	-0.861419224	0.274	0.451	1	19	Psma7
0.00011487	0.348903396	0.89	0.702	1	19	mt-Cytb
0.00011519	-0.988368934	0.068	0.258	1	19	Flna
0.00011944	0.297298591	0.836	0.66	1	19	Rps20
0.00012344	0.405896867	0.315	0.151	1	19	Comt
0.00012363	0.34112383	0.781	0.522	1	19	Tma7
0.00014219	0.449405714	0.411	0.221	1	19	Snrpd1
0.00014256	0.359508416	0.288	0.13	1	19	Dnajc19
0.00014339	0.296358263	0.89	0.726	1	19	Rps25
0.00015104	0.306219423	0.822	0.583	1	19	Gm26917
0.00015418	0.290060845	0.438	0.227	1	19	Anp32e
0.00016416	-0.778160367	0.164	0.37	1	19	Capza2
0.00016964	0.426746922	0.384	0.192	1	19	Klf13
0.00018297	0.355530318	0.507	0.29	1	19	Sarnp
0.0001898	-0.950099319	0.068	0.253	1	19	Psmb8
0.00019882	0.268300782	0.918	0.764	1	19	Rpl36
0.00020041	0.258974398	0.329	0.153	1	19	Bnip3l
0.00020879	0.362485751	0.493	0.268	1	19	Romo1
0.00021195	-0.975582946	0.096	0.277	1	19	Aldh2
0.00022026	-1.436280086	0.192	0.361	1	19	Ifi27l2a
0.00022959	-0.827079993	0.082	0.273	1	19	Aldoa
0.00022993	0.388209874	0.356	0.178	1	19	Luc7l3
0.0002354	0.337723412	0.301	0.14	1	19	Pim1

0.00023873	0.373384432	0.671	0.429	1	19	Serinc3
0.0002409	0.326868672	0.466	0.257	1	19	Atp5g3
0.00025325	-0.795448161	0.26	0.426	1	19	Tkt
0.00025495	-0.921789148	0.082	0.261	1	19	Aprt
0.00027007	0.305407231	0.699	0.457	1	19	Ndufa2
0.00027871	0.31893973	0.507	0.286	1	19	Sdcbp
0.00030778	0.273533665	0.288	0.131	1	19	Ctcf
0.0003078	-0.747030963	0.288	0.443	1	19	Actr2
0.00030816	0.414394992	0.356	0.19	1	19	Hnrnpl
0.00030952	0.457262552	0.548	0.334	1	19	Nucks1
0.00031499	-0.672664631	0.425	0.543	1	19	Ubb
0.00032032	0.402851763	0.329	0.165	1	19	Yme1l1
0.00032386	-0.926927828	0.11	0.283	1	19	Myl12b
0.00033109	0.343716567	0.63	0.432	1	19	Rpl13-ps3
0.00034296	-0.477538872	0.63	0.716	1	19	Cfl1
0.00034907	0.394585401	0.603	0.41	1	19	2010107E04Rik
0.00035339	0.253879214	0.918	0.771	1	19	Rps3a1
0.00035815	0.355500495	0.452	0.253	1	19	Ncl
0.00036424	0.359718272	0.301	0.146	1	19	Sod1
0.00037639	0.292863834	0.507	0.282	1	19	Eef1g
0.00039632	-0.922582778	0.11	0.281	1	19	Lcp1
0.00039702	-0.838168348	0.178	0.355	1	19	Msrb1
0.00041275	-0.855133698	0.082	0.26	1	19	Lmo4
0.00042485	0.316879355	0.356	0.181	1	19	2310036O22Rik
0.00043548	-0.94513567	0.123	0.296	1	19	Sat1
0.00044082	0.334436099	0.342	0.174	1	19	Mrpl18
0.00044227	0.419072719	0.301	0.152	1	19	Srsf11
0.00047968	0.422941055	0.63	0.426	1	19	Sumo2
0.00050429	0.409711639	0.466	0.28	1	19	Atp5b
0.00052321	0.382824019	0.356	0.187	1	19	Thoc7
0.00053032	0.311773098	0.411	0.219	1	19	Sf3b6

0.00057618	0.389453546	0.356	0.189	1	19	Elavl1
0.00060068	-1.339776063	0.11	0.275	1	19	Prtn3
0.00061854	0.29374967	0.384	0.204	1	19	Ndufa5
0.00071081	-0.807228071	0.164	0.328	1	19	Lamtor4
0.00071092	-0.717830313	0.26	0.419	1	19	Cdc42
0.00072152	0.314588694	0.699	0.451	1	19	Uqcr10
0.00072443	0.290439153	0.699	0.467	1	19	Rps18-ps3
0.00073147	0.25943169	0.356	0.184	1	19	Ptp4a2
0.00074304	0.324834035	0.315	0.161	1	19	Nhp2l1
0.00079404	0.271185871	0.534	0.319	1	19	Gm10036
0.00080168	0.329709251	0.466	0.275	1	19	Arglu1
0.00081806	-0.750607103	0.151	0.319	1	19	Capza1
0.00086972	-0.801794185	0.178	0.335	1	19	Irf2bp2
0.00091175	-0.662162284	0.164	0.339	1	19	Clic1
0.00092669	0.344658679	0.712	0.519	1	19	Atp5h
0.00098871	-0.593220999	0.479	0.561	1	19	Calm2
0.00102802	-0.888240254	0.219	0.371	1	19	Myh9
0.00105013	0.257822332	0.849	0.652	1	19	mt-Co2
0.00106752	-0.77470407	0.137	0.299	1	19	Cdk2ap2
0.00107804	-0.772639074	0.548	0.588	1	19	Hmgn2
0.00117976	0.276223051	0.479	0.289	1	19	D8Ertd738e
0.00121783	0.392277263	0.603	0.426	1	19	Hnrnpk
0.00124516	0.344674996	0.315	0.171	1	19	Rdx
0.0012969	0.428399592	0.329	0.182	1	19	Erdr1
0.00131246	-0.526111269	0.397	0.537	1	19	Mrpl33
0.00132897	-0.773456783	0.151	0.306	1	19	Taldo1
0.00134148	0.302403741	0.342	0.185	1	19	Srsf6
0.00137398	0.298466629	0.315	0.164	1	19	Paip1
0.00152619	0.264801122	0.397	0.222	1	19	Mrps14
0.00158693	0.376104054	0.575	0.38	1	19	Minos1
0.0016706	0.387556405	0.301	0.162	1	19	H2afx

0.00175941	-0.400076929	1	0.997	1	19	Malat1
0.001809	-0.827965496	0.219	0.36	1	19	H2-K1
0.00186423	-0.762851967	0.123	0.271	1	19	Abracl
0.00188849	0.376381861	0.301	0.163	1	19	Larp4b
0.00189908	0.259910666	0.301	0.155	1	19	Emg1
0.00191534	0.309010628	0.356	0.197	1	19	AI662270
0.00195838	0.281084524	0.425	0.25	1	19	Ndufb2
0.00204839	0.348820217	0.384	0.219	1	19	Top1
0.00211631	0.37122855	0.616	0.425	1	19	Rbm3
0.0022262	0.276476764	0.808	0.601	1	19	Cox7c
0.00254464	0.267819521	0.315	0.166	1	19	Higd1a
0.00256263	-0.581816243	0.384	0.505	1	19	Mbnl1
0.00258581	0.298671705	0.315	0.173	1	19	Uqcc2
0.00260435	-0.824898307	0.342	0.429	1	19	Prdx1
0.0027436	-0.820783918	0.151	0.285	1	19	Cd44
0.00277863	0.316243385	0.315	0.174	1	19	Stag2
0.00281601	0.393621164	0.548	0.364	1	19	Ran
0.00290465	0.282751456	0.288	0.15	1	19	Map1lc3b
0.00296245	-0.577007158	0.274	0.416	1	19	Gapdh
0.00328862	-0.705430529	0.192	0.325	1	19	Scp2
0.00353357	0.274693031	0.329	0.183	1	19	Mrpl52
0.00357559	0.300489563	0.466	0.291	1	19	Nap1l1
0.00382699	-0.679827149	0.11	0.252	1	19	Ostf1
0.00394175	0.26308814	0.63	0.449	1	19	Hnrnpa3
0.00560348	0.296884784	0.26	0.143	1	19	Mat2a
0.00655016	0.358954563	0.384	0.239	1	19	Ube2s
0.00669675	0.275740724	0.288	0.16	1	19	Wnk1
0.0072629	0.292905037	0.384	0.238	1	19	Eif5b
0.00745853	0.287678292	0.315	0.186	1	19	Skp1a
0.00832138	0.281220432	0.274	0.156	1	19	Dpy30
0.00928852	-0.46662445	0.425	0.508	1	19	Sec61b

0.00953458	-0.725797385	0.137	0.256	1	19	Btg1	
0.00959581	0.330544194	0.315	0.196	1	19	2410015M20Rik	
0	2.847547529	0.771	0.006	0	20	C1qc	**Macrophage**
0	2.441741056	0.629	0.003	0	20	C1qa	
0	2.409943012	0.686	0.004	0	20	C1qb	
2.92E-276	2.033223626	0.543	0.005	3.85E-272	20	Mrc1	
7.86E-272	2.010386719	0.429	0.003	1.04E-267	20	Fcna	
3.61E-250	1.694865919	0.286	0.001	4.76E-246	20	Cd5l	
7.06E-174	2.286361789	0.486	0.008	9.31E-170	20	Axl	
3.76E-158	3.384127364	0.8	0.027	4.96E-154	20	Vcam1	
4.26E-153	1.978552227	0.429	0.007	5.61E-149	20	Maf	
6.63E-131	2.246856622	0.629	0.019	8.75E-127	20	Sdc3	
1.40E-130	2.110448301	0.6	0.017	1.85E-126	20	Lgmn	
1.70E-128	2.008689137	0.457	0.01	2.24E-124	20	Pld3	
4.69E-104	2.028417519	0.429	0.011	6.18E-100	20	Mafb	
7.91E-95	1.521989522	0.257	0.004	1.04E-90	20	Cd163	
3.88E-83	2.740880002	0.571	0.027	5.11E-79	20	Hmox1	
7.73E-69	1.934485954	0.4	0.015	1.02E-64	20	Tcf7l2	
3.76E-57	1.488812876	0.314	0.011	4.96E-53	20	Ckb	
2.71E-52	2.659611448	0.686	0.065	3.57E-48	20	Slc40a1	
1.00E-33	1.763763771	0.371	0.028	1.32E-29	20	Lpl	
5.70E-32	2.057187919	0.657	0.096	7.52E-28	20	Sepp1	
7.50E-32	1.564462676	0.4	0.034	9.89E-28	20	Trf	
1.17E-29	2.076959682	0.657	0.104	1.55E-25	20	Marcks	
2.26E-27	1.431882638	0.257	0.016	2.98E-23	20	Fcgrt	
2.27E-25	2.138772435	0.686	0.131	2.99E-21	20	Apoe	
3.38E-22	1.622713191	0.6	0.11	4.46E-18	20	Ctsd	
4.87E-18	1.282377008	0.486	0.084	6.43E-14	20	Aplp2	
1.10E-17	1.075228351	0.314	0.037	1.45E-13	20	Lgals3bp	
2.50E-17	1.491612005	0.743	0.209	3.30E-13	20	Grn	
1.88E-14	1.100449666	0.343	0.052	2.49E-10	20	Ptprj	

1.84E-12	1.096103874	0.343	0.06	2.43E-08	20	Ccdc50
1.00E-11	1.634258364	0.629	0.21	1.32E-07	20	Ctsb
2.66E-11	1.009653333	0.4	0.083	3.51E-07	20	Cfp
3.93E-11	1.239372771	0.286	0.048	5.18E-07	20	Glul
4.75E-11	1.412757061	0.429	0.104	6.26E-07	20	Slc6a6
9.68E-09	0.945315067	0.343	0.081	0.000127665	20	Man2b1
1.66E-08	0.961797662	1	0.998	0.000218632	20	Malat1
2.39E-08	0.765822105	0.971	0.82	0.000315474	20	Ftl1
7.11E-08	1.044492433	0.314	0.076	0.000937594	20	Csf1r
1.09E-07	1.024437481	0.343	0.091	0.001433955	20	Actn1
1.26E-07	1.007760781	0.8	0.542	0.001664473	20	Psap
2.03E-07	0.81965985	0.886	0.641	0.002681099	20	Fth1
2.88E-07	1.084836066	0.314	0.082	0.003794235	20	Sirpa
6.97E-06	-0.839560908	0.714	0.852	0.09190608	20	Rpl13
1.09E-05	0.571381901	0.257	0.066	0.143172113	20	Lair1
1.51E-05	0.835769784	0.257	0.072	0.198934638	20	Nudcd3
2.45E-05	0.598491191	0.257	0.071	0.323554775	20	Fxr1
3.05E-05	0.871204035	0.571	0.293	0.402769379	20	Sat1
3.96E-05	0.855694661	0.257	0.077	0.522419257	20	Ctsa
4.05E-05	0.696326821	0.829	0.648	0.534669504	20	Itm2b
4.88E-05	1.047104096	0.257	0.077	0.64385428	20	Rnf149
7.88E-05	0.794714551	0.286	0.097	1	20	Kcnq1ot1
9.30E-05	0.702617952	0.257	0.079	1	20	Stat1
0.00010331	-0.704878655	0.743	0.868	1	20	Rpl32
0.00011572	0.727022534	0.6	0.314	1	20	Ptprc
0.00013854	0.800877632	0.4	0.157	1	20	Cd74
0.0001455	-1.161453462	0.114	0.436	1	20	Rpl13-ps3
0.00016629	0.718968987	0.257	0.084	1	20	Atp6ap1
0.00018777	0.604454711	0.686	0.442	1	20	Ddx5
0.00019394	0.728711282	0.343	0.136	1	20	Adipor1
0.00020611	0.82198011	0.486	0.235	1	20	Zeb2

0.00021257	-0.580901139	0.571	0.821	1	20	H2afz
0.00022494	-0.744131563	0.514	0.723	1	20	Rpl6
0.00023791	0.783847076	0.4	0.172	1	20	Creg1
0.00024636	-1.005859458	0.371	0.616	1	20	Ly6c2
0.00024967	-0.694985945	0.486	0.749	1	20	Rps6
0.00028393	-0.676131342	0.514	0.729	1	20	Rps25
0.00030108	0.898794415	0.286	0.103	1	20	Ctnnb1
0.00031219	-0.809140365	0.371	0.633	1	20	Uba52
0.00031619	0.632211266	0.286	0.103	1	20	Tuba1c
0.0003447	0.765575378	0.314	0.125	1	20	Tgfb1
0.00041737	0.749590692	0.343	0.141	1	20	Rab1a
0.00043436	0.63415323	0.257	0.09	1	20	AW112010
0.00044108	-0.707525345	0.571	0.746	1	20	Rpl24
0.0004867	-0.764962817	0.571	0.712	1	20	Rplp0
0.00049443	0.61337494	0.257	0.089	1	20	Rtn4
0.00062389	-0.7538282	0.343	0.613	1	20	Gm11808
0.00072486	-0.512636603	0.743	0.875	1	20	Rpl18a
0.00073222	-0.806826801	0.286	0.552	1	20	Cox6b1
0.00082324	0.599747635	0.429	0.205	1	20	Ctss
0.00085808	0.551110622	0.543	0.306	1	20	Canx
0.00085956	-0.969057516	0.2	0.471	1	20	Gm8730
0.00100783	-0.513142386	0.829	0.882	1	20	Rps14
0.00102077	-0.671050672	0.486	0.71	1	20	Rpl31
0.00113155	-0.994530864	0.057	0.327	1	20	Eif3f
0.00124349	-0.546157821	0.571	0.782	1	20	Rpl26
0.00135398	-0.903607851	0.114	0.387	1	20	Tmem258
0.00139468	0.652182121	0.571	0.357	1	20	H2-K1
0.00141808	-0.502554004	0.857	0.902	1	20	Rpl37
0.00145002	-0.654373743	0.571	0.726	1	20	Rps4x
0.00146639	-0.665039617	0.543	0.692	1	20	Rps7
0.00163727	0.643573746	0.514	0.287	1	20	Sdcbp

0.00177767	0.60012024	0.457	0.255	1	20	Alyref
0.00186522	0.73463953	0.286	0.121	1	20	Slc39a1
0.00188255	-0.548471744	0.629	0.754	1	20	Gm10076
0.00200757	0.566729049	0.371	0.176	1	20	Cltc
0.00205336	-0.516928602	0.686	0.843	1	20	Ptma
0.00222921	0.728755391	0.286	0.127	1	20	Ctsc
0.00230411	-0.469558554	0.829	0.897	1	20	Rps19
0.00231969	-0.770188314	0.371	0.577	1	20	Rpl10a
0.00233029	0.702302501	0.4	0.209	1	20	Lamp1
0.00235277	0.65748079	0.286	0.12	1	20	Map3k1
0.00248996	-0.586654329	0.514	0.725	1	20	Rps26
0.00259209	0.682470842	0.314	0.152	1	20	Comt
0.00276702	0.564520786	0.257	0.101	1	20	Hcls1
0.00285251	0.54621952	0.314	0.143	1	20	Ppp2ca
0.00291267	0.776295034	0.257	0.109	1	20	Dennd5a
0.00336563	0.251062972	0.829	0.631	1	20	Cst3
0.00351164	0.895474697	0.686	0.475	1	20	Retnlg
0.00395611	-0.578780014	0.514	0.697	1	20	Rpl10
0.00421615	0.404942619	0.257	0.104	1	20	Insig1
0.00434187	0.66425944	0.314	0.155	1	20	Akr1a1
0.00448867	-0.989674338	0.114	0.328	1	20	Ap3s1
0.00491005	-0.603681402	0.371	0.592	1	20	Rpl4
0.00509433	-0.480954064	0.771	0.836	1	20	Rps18
0.00525856	-0.910810144	0.114	0.327	1	20	Lamtor4
0.00558331	-0.606763401	0.571	0.671	1	20	Rpl23a-ps3
0.00590502	-0.842495775	0.114	0.341	1	20	Ifitm2
0.00613741	-0.757836933	0.257	0.471	1	20	Rps18-ps3
0.00613913	-0.705037164	0.371	0.589	1	20	Hmgn2
0.00623971	-0.510513906	0.6	0.718	1	20	Rpl11
0.00628179	-0.783849191	0.4	0.555	1	20	Rps2
0.00632161	0.553226842	0.257	0.112	1	20	Snx5

0.00640215	-0.513609885	0.229	0.521	1	20	Snrpg
0.0067329	-0.654065486	0.4	0.58	1	20	Rpl15
0.00715411	-0.442626539	0.714	0.805	1	20	Rps24
0.00718204	-0.678273021	0.543	0.685	1	20	Plac8
0.00809937	0.57359517	0.257	0.119	1	20	Tm9sf2
0.00832386	-0.512942562	0.514	0.71	1	20	Myl6
0.00838122	-0.478895189	0.686	0.785	1	20	Rps5
0.00851243	-0.555737513	0.4	0.601	1	20	Rpl3
0.00877925	0.509565163	0.343	0.182	1	20	Vsir
0.0096764	-0.822668134	0.114	0.309	1	20	Ppp1ca

Table 1. Markers defining each of the 20 clusters identifeid in **Fig. 1A**.
pct.1 represents the percent of the clutser expression a specific gene.
pct.2 represent the percent of cells outside that clu.ster that express the gene
